# Adversarial dynamical systems characterize when data-driven learning succeeds or fails

**DOI:** 10.1038/s41467-026-74220-8

**Published:** 2026-07-14

**Authors:** Matthew J. Colbrook, Igor Mezić, Alexei Stepanenko

**Affiliations:** 1 DAMTP, University of Cambridge, Cambridge, UK; 2 University of California, Santa Barbara, CA USA

**Keywords:** Computational science, Applied mathematics, Scientific data

## Abstract

Many systems resist analytical modeling, making data-driven inference of dynamics important. Yet data-driven methods can fail to converge or generalize, leaving open a central question: *When can system behavior be learned reliably from data, and when is such learning impossible?* We answer this question using adversarial dynamical systems to identify the boundary between accessible and inaccessible regimes. In Koopman operator learning, a leading framework for representing nonlinear dynamics through linear spectral objects, we design optimal data-driven spectral algorithms with convergence and certification guarantees under conditions arising broadly in physical systems. This yields a convergence theory for Koopman-operator approximations and resolves a longstanding open problem in Koopman spectral analysis. Conversely, by constructing adversarial systems, we prove matching impossibility results: without these conditions, no single-sequence limiting procedure can guarantee learning, regardless of data quality. These results sharply characterize when data-driven spectral learning can succeed and when it must fail. We validate the framework on oscillators, chaotic fluid flows and Arctic sea ice concentration forecasting. In the latter, we uncover hidden modes of Arctic sea ice decline, deliver long-range forecasts with geographic error bounds, and outperform state-of-the-art dynamical and deep learning models at substantially lower computational cost, enabling real-time deployment on standard CPUs.

## Abstract (web summary)

Machine learning can help predict complex systems from data, but its reliability is often unclear. Here, authors use adversarial dynamical systems to identify when learning succeeds or must fail, build guaranteed algorithms, and reveal hidden patterns of Arctic sea-ice loss, improving forecasts.

## Introduction

Models across science often involve systems that evolve over time, known as dynamical systems. These systems have long been used to understand, predict, and control complex behavior across physics, chemistry, biology, and medicine. Yet in many fields such as climate science, neuroscience, and robotics, systems are often too complex for direct analysis, or their governing equations are unknown. Machine learning (ML) has transformed the analysis of complex data^1^, with breakthroughs in protein structure prediction^2^ (see also the 2024 Nobel Prize in Chemistry^3^), image classification^4^, and drug/material discovery^5^. The emerging field of *data-driven dynamical systems* seeks to combine ML with time-series data to uncover underlying structure and principles without requiring explicit models^6–16^.

Current ML techniques often struggle to converge or generalize, with limited guarantees of their trustworthiness. This hinders their effectiveness in critical applications and poses a central challenge: *When can system behavior be learned reliably from data, and when is such learning impossible?* We address this with:

- **Adversarial dynamical systems:** We present examples in data-driven dynamical systems for which no sequence of learning algorithms-probabilistic or otherwise-can solve, even with unlimited data. By carefully altering a system’s behavior in a way that respects both its structure and the data, we design adversarial systems that block reliable learning. These are not rare edge cases: success is fundamentally limited to 50% and they arise in well-studied classes. Even for smooth systems on simple low-dimensional surfaces, tasks such as learning finite-dimensional linearized representations (e.g., via autoencoders) remain unsolvable.
  This parallels adversarial attacks in machine learning, where small perturbations expose vulnerabilities and drive the development of robust methods. Similarly, our adversarial systems reveal fundamental learning limits across broad classes of dynamical systems and offer principles for trustworthy algorithms. They may also shed light on phenomena such as hallucinations in large language models (LLMs), as discussed below.
- **Optimal algorithms with learning guarantees:** Building on insights from these adversarial systems (see challenges **(C1)** and **(C2)** below), we develop provably optimal algorithms with guaranteed convergence and error bounds under broad conditions. Unlike traditional approaches that rely on sequences of algorithms, our methods reach fundamental limits and enable reliable extrapolation, crucial for trustworthy AI. Our models are also trained on CPUs at a fraction of the cost of deep learning approaches, far exceeding the scales at which recent claims of efficiency have been made^17^.
- **A universal framework for classification:** We establish a rigorous yet practical mathematical foundation that clarifies when and why learning succeeds or fails. Matching lower and upper bounds on difficulty reveal the core challenges and offer a comprehensive classification of problem complexity.

These results offer a unified, rigorous, and practical basis for understanding when data-driven models can or cannot succeed, advancing the broader goal of trustworthy ML. They are applicable across fields from climate science and neuroscience to engineering and control (see “Discussion”), where reliable prediction and mechanistic insight are essential.

We apply this framework to Koopman operators, a major research focus that addresses nonlinearity by acting on an infinite-dimensional space of measurements rather than the system’s state. Introduced nearly a century ago by Koopman and von Neumann^18,19^, Koopman operators now play a central role in data-driven dynamical systems^7,12,20–23^. Their spectral properties (e.g., eigenfunctions and eigenvalues) decompose complex behavior into simpler components like trends, oscillations, or decay, allowing for the use of linear methods in prediction, estimation, and control. This enables explainable, robust, and cost-efficient ML. Notable successes include robot control^24^, climate analysis^25^, neural network training^26^, disease modeling^27^, brain analysis^28^, non-autonomous systems^29^, and interpretable AI^30^.

**Fig. 1:**
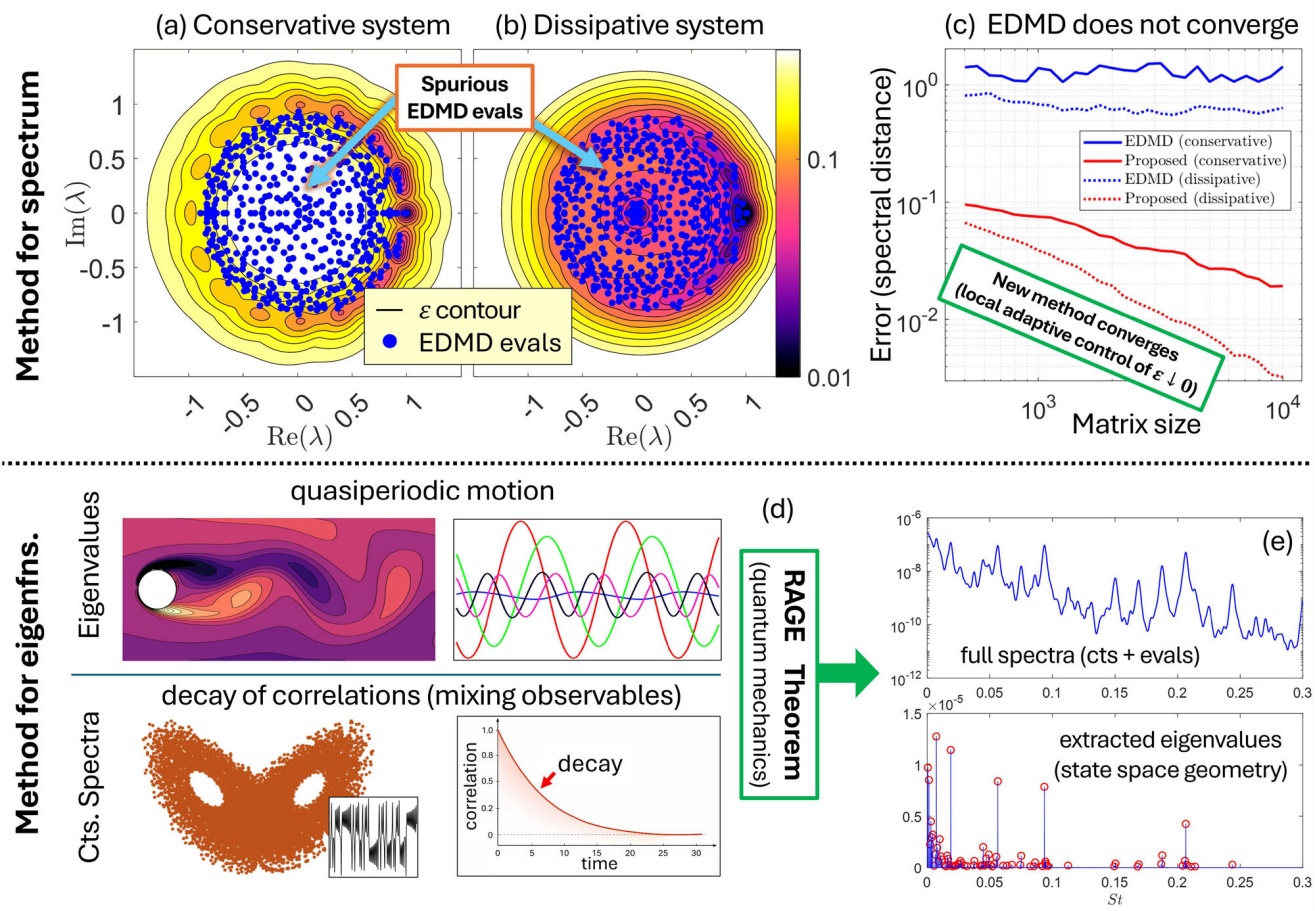
Convergent general-purpose methods for Koopman learning. Top: The method (Supplementary Algorithm 1) is benchmarked on the Duffing oscillator in conservative (**a**) and dissipative (**b**) regimes. The state-of-the-art EDMD method fails to converge (**c**), generating spurious eigenvalues (blue). In contrast, our approach converges reliably by adaptively computing temporally coherent observables *ϕ*_*ε*_ and associated scalars *λ* that approximately satisfy *ϕ*_*ε*_(*x*_*n*_) = *λ*^*n*^*ϕ*_*ε*_(*x*_0_) (*x*_*n*_ is the state of the system at time *n*) up to a controlled tolerance *ε*. The contours of *ε* show where approximate, near-eigenvalue behavior occurs, providing a more robust picture of dynamics. The tolerance *ε* is adjusted locally based on data availability and the number of observables used, yielding certified error bounds. The reported error corresponds to the maximum *ε* across outputs (averaged over 10 random realizations) and provably bounds the distance to the true spectrum (see Equation (16) of the Supplementary Information). Bottom: The method (Supplementary Algorithm 6) is applied to the *Re* = 19,000 cavity flow to extract Koopman eigenvalues in a regime exhibiting signatures of continuous spectrum. Observables are separated according to their long-time behavior (**d**): quasiperiodic components correspond to discrete eigenvalues, whereas mixing components spread across the continuous spectrum and progressively lose finite-dimensional representation. This distinction is reflected in the full spectral distribution and in the extracted eigenvalues shown in (**e**).

However, Koopman theory faces substantial practical challenges. Spectral approximation in infinite-dimensional settings is frequently non-convergent, even with perfect data^11,23,31^. The most widely used approach, Dynamic Mode Decomposition (DMD)^32^, and its variants, including extended DMD (EDMD)^33^, often generate spurious eigenvalues and fail to converge (Fig. 1, top). Koopman operators are typically non-self-adjoint (non-Hermitian) and may possess continuous rather than purely discrete spectra^7^, limiting the applicability of classical spectral approximation techniques^34,35^. Although recent advances^14,16,36,37^ address specific spectral properties under certain assumptions, they rely on multiple limiting procedures and do not provide a unified approach for convergence in all cases.

**Fig. 2:**
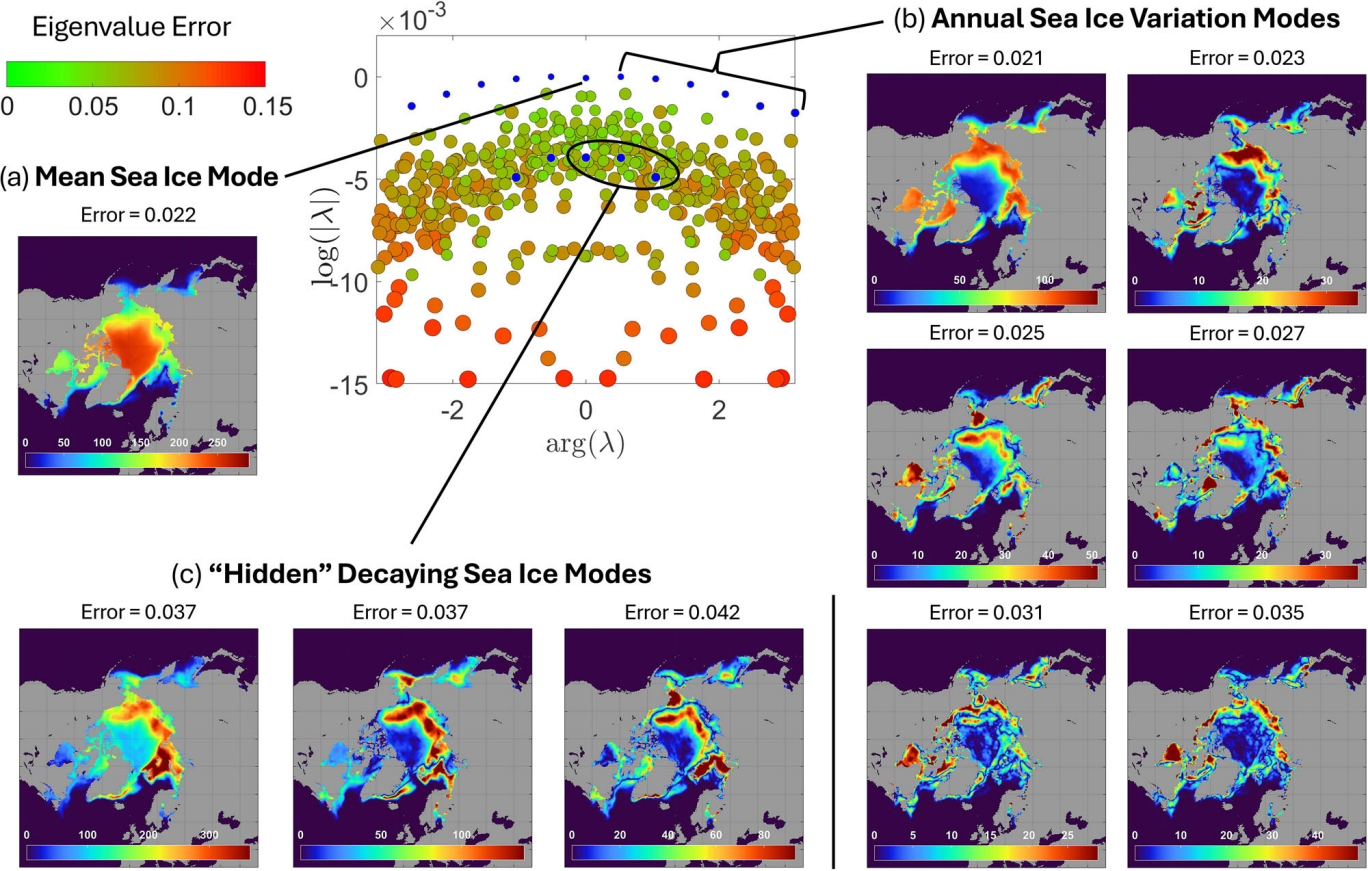
Verified eigenvalues and Koopman modes of Arctic sea ice (1979–2021) and detection of hidden modes for long-time forecasts. For each EDMD eigenvalue, our proposed method computes an associated error bound (colorbar). The displayed size of each eigenvalue is proportional to this error. While many EDMD eigenvalues are spurious, we identify 17 reliable ones (shown in blue) with small associated errors. The Koopman modes (the **g**_*j*_ in Eq. (6), where we plot their absolute value) are categorized into three groups: **a** the mean sea ice concentration, corresponding to a stationary mode with eigenvalue *λ* = 1; **b** annual variation in sea ice concentration, with $\lambda=\exp (m\pi i/6)$ for *m* = ± 1, ± 2, …, ± 5, 6, representing periodic variation across the months; and (**c**) “hidden” decaying modes with ∣*λ*∣ < 1 (revealed by our error bounds), representing long-term sea ice loss. The spatial structure of each mode indicates the geographic regions where these behaviors occur, and the corresponding Koopman eigenfunction time series shows a clear trend over the analysis interval. The hidden modes with nonzero $\arg (\lambda )$ can be interpreted as seasonal patterns (group (**b**)) modulated by the decaying mode with zero argument, providing insight into evolving Arctic sea ice dynamics.

**Fig. 3:**
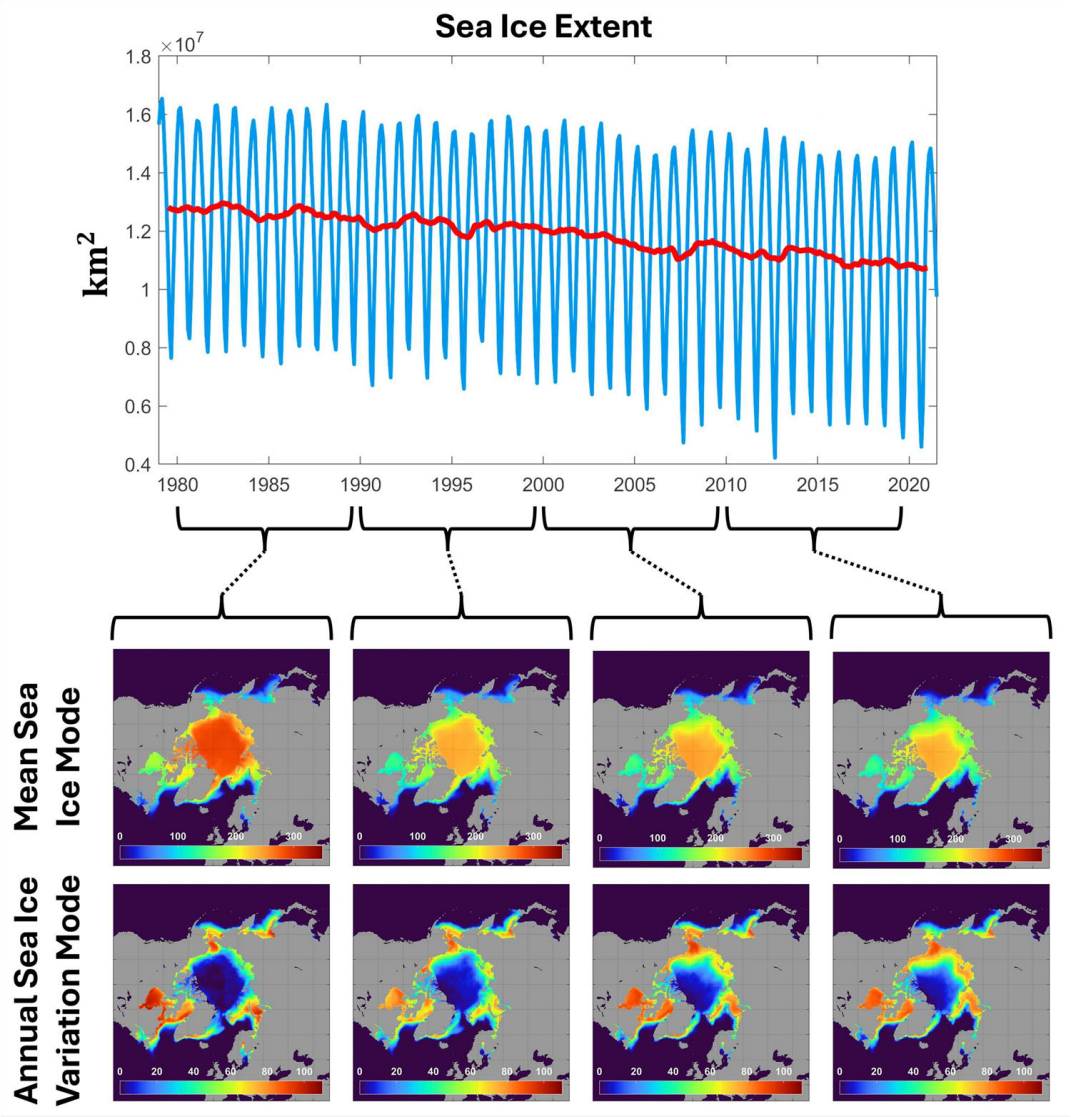
Sea ice decay and time-windowed Koopman modes. Top: Sea ice extent over the past several decades. The red curve shows the moving 12-month mean. Bottom: The absolute value of Koopman modes corresponding to the mean sea ice and annual variations over ten-year windows. The maximum error (relative residual) of these modes is 0.048. Mean and annual modes reveal declining sea ice, reduced winter extent, and amplified seasonal contrast in marginal seas, consistent with a slow decaying mode (Fig. 2).

**Fig. 4:**
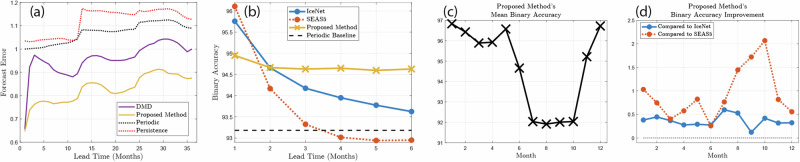
Comparison with benchmarks. **a** Forecast error for sea ice concentration at every grid point. Relative error of forecasted anomalies (relative to the periodic monthly climatological variance). We consider three-year forecasts initialized at each month from 2005 to 2015 and plot the average error for each lead time. The proposed method consistently outperforms DMD. **b** Mean binary accuracy (see text for definition) over the test years 2012–2020, shown for IceNet, SEAS5, and our proposed method that avoids spurious Koopman eigenvalues. Our proposed method achieves better accuracy for lead times greater than one month, with very little increase in errors at larger lead times. Moreover, this is achieved using orders of magnitude fewer trainable parameters and substantially less computational cost. **c** Mean binary accuracy of our proposed method over all lead times and years. **d** Improvements in binary accuracy over IceNet and SEAS5.

Koopman theory thus provides an ideal setting to explore our central question. We present a complete treatment of sharp algorithms and computational difficulty for Koopman spectra. In particular, we introduce general-purpose, provably convergent methods for learning Koopman spectral properties (Supplementary Algorithms 1–5, top panel of Fig. 1) that avoid spurious eigenvalues through explicit local minimization of spectral errors. We further adapt tools from quantum mechanics to separate spectral components with distinct physical signatures (Supplementary Algorithms 6–7, bottom panel of Fig. 1). These methods perform well across low- and high-dimensional systems, including challenging cases where the system’s behavior spans a continuous range of frequencies, rather than discrete periodic behavior. This includes practical applications such as forecasting Arctic sea ice (Figs. 2–4).

This final example is motivated by Arctic amplification, where near-surface Arctic temperatures are rising faster than the global average^38^. Sea ice loss has major impacts on polar bear habitats, Indigenous communities, shipping, and the Atlantic Meridional Overturning Circulation (AMOC). Concurrently, extreme weather events (e.g., wildfires, floods, heatwaves, and severe winters) have intensified in recent decades, affecting billions worldwide. The link between Arctic sea ice loss and Northern Hemisphere extreme weather remains an active area of research and debate^39–41^. While regional effects appear likely, identifying geographically significant regions and their influence is particularly challenging. Forecasting Arctic sea ice beyond two months remains a major challenge^42^.

Our algorithms uncover a family of hidden Koopman modes linked to sea ice decline and identify the associated geographic regions with error bounds. These patterns support accurate long-term predictions and reveal how different parts of the system are connected. By building data-driven decompositions from these and other validated modes, we achieve state-of-the-art Arctic sea ice forecasts (Fig. 4). Notably, such modes can influence tipping behavior, including patterns related to the AMOC^43,44^.

The adversarial dynamics we construct may also shed light on hallucinations in LLMs. LLMs generate trajectories (sentences) over a state space (words) via one-step-ahead prediction, a process linked to Koopman operators^26,45,46^. Our adversarial systems often have Koopman operators with a continuous spread of frequencies, characteristic of chaotic dynamics. This enables short-term accuracy but causes long-term unpredictability due to sensitivity to initial conditions, mirroring how small prompt changes in LLMs can cause divergent outputs. Our results may therefore help explain inherent limitations of autoregressive architectures in maintaining long-term accuracy.

## Results

### Multiple limits: bad, good, or insufficient data?

Learning systems from data requires two elements: quantities we can measure using sensors and their corresponding time series data. In ML, one often studies sequences of algorithms indexed by *n*, where *n* might represent the size of the dataset or the complexity of the model (for example, the width or depth of a neural network). A simple illustration is estimating the average energy *E* of an oscillating system based on measurements of its instantaneous energy *e*(*n*) at each time step *n*. If the system is ergodic (meaning that averages along a single long-term trajectory reflect the behavior of the entire system), then *E* can be estimated iteratively by:

1 $${\Gamma }_{n+1}=\frac{e(1)+\cdots+e(n+1)}{n+1}=\frac{n}{n+1}{\Gamma }_{n}+\frac{e(n+1)}{n+1}.$$

This running average *Γ*_*n*_ becomes more accurate as more data is collected. A classical result known as Birkhoff’s ergodic theorem^47^ guarantees that *Γ*_*n*_ converges to the true average energy *E* as *n* → *∞*, i.e., as we collect more data.

Learning often assumes that increasing the amount of data or model complexity will improve performance. However, our results show that for many key problems in dynamical systems, this assumption is false: no algorithm *Γ*_*n*_ can succeed by taking a single limit, regardless of how that limit is defined (e.g., sample size, model complexity, simulation length).

Instead, some problems only become solvable when multiple data limits are applied in a specific order. For example, computing long-term structures in a system, such as ergodic partitions^48^, requires not just averaging over time (as in Equation (1)) but also subsequently computing the averages for a growing number of measured quantities (instead of just the energy *e*). These two types of data increase, longer observations and richer measurements, typically cannot be combined or chosen adaptively from the data itself. This leads to a hierarchy of difficulty, where each level corresponds to the number and type of data limits needed for reliable learning.

### Dynamical setup

A dynamical system describes how a system changes over time. Suppose we track a collection of variables, denoted *x*, that represent the system’s state at a given moment. The set of all possible states is called the state space, written as ${{{\mathcal{X}}}}$, so that $x\in {{{\mathcal{X}}}}$. The system evolves in discrete time steps, meaning the next state *x*_*n*+1_ depends on the current state *x*_*n*_ via a rule:

2 $${x}_{n+1}=F({x}_{n}),\,\,n=0,1,2,\ldots .$$

Here, $F:{{{\mathcal{X}}}}\to {{{\mathcal{X}}}}$ is an unknown function that may come from sampling a continuous-time process.

To study a system, we measure its physical properties like temperature or velocity, represented by functions $g:{{{\mathcal{X}}}}\to {\mathbb{C}}$, called “observables”. One should think of *g*(*x*) as a quantity we can measure from state *x*. The Koopman operator, ${{{{\mathcal{K}}}}}_{F}$ (or ${{{\mathcal{K}}}}$), captures how these measurements evolve. It acts on an observable *g* by composing it with the system’s evolution:

3 $$[{{{\mathcal{K}}}}g](x)=[g\circ F](x)=g(F(x)),\,\,x\in {{{\mathcal{X}}}}.$$

This means ${{{\mathcal{K}}}}g$ gives the value of the observable one step into the future: $[{{{\mathcal{K}}}}g]({x}_{n})=g({x}_{n+1})$. The key property of ${{{\mathcal{K}}}}$ is linearity: for any observables *f* and *g* and scalars *α* and *β*, ${{{\mathcal{K}}}}(\alpha f+\beta g)=\alpha {{{\mathcal{K}}}}(f)+\beta {{{\mathcal{K}}}}(g)$. Linearity is powerful since it allows us to analyze the system through spectral properties of ${{{\mathcal{K}}}}$ (e.g., eigenvalues and eigenfunctions). The trade-off for this global linearization is that ${{{\mathcal{K}}}}$ acts on an infinite-dimensional space of observables. One can think of ${{{\mathcal{K}}}}$ as an infinite matrix, corresponding to the infinite number of observables *g*.

The goal is to learn the spectral properties of the Koopman operator from snapshot data, discrete sample pairs of the system’s behavior:

4 $$\left\{\left({x}^{(m)},{y}^{(m)}=F({x}^{(m)})\right):m=1,\ldots,M\right\}.$$

Here, each *y*^(*m*)^ represents the state one time step ahead of *x*^(*m*)^. Such data can arise from experiments or simulations, and observations of either long or short trajectories. We shall see examples of each of these throughout the paper.

### Separation of variables & spectra in nonlinear systems

Spectral properties of Koopman operators contain valuable information about the system. For example, a complex number $\lambda \in {\mathbb{C}}$ is called an almost eigenvalue if, for a tolerance *ε* > 0, there exists a normalized observable *ϕ*_*ε*_ with $\parallel {{{\mathcal{K}}}}{\phi }_{\varepsilon }-\lambda {\phi }_{\varepsilon }\parallel \le \varepsilon$. (Here, ∥ ⋅ ∥ measures an observable’s energy.) These observables, called approximate eigenfunctions, are physically relevant because, assuming $\parallel {{{\mathcal{K}}}}\parallel \le 1$, they exhibit approximate temporal coherence^12,49^:

5 $${\phi }_{\varepsilon }({x}_{n})={\lambda }^{n}{\phi }_{\varepsilon }({x}_{0})+{{{\mathcal{O}}}}(n\varepsilon )\,\,{{{\rm{as}}}}\,\,\varepsilon \downarrow 0\,\,{{{\rm{for}}}}\,\,n=1,2,\ldots .$$

That is, *λ* approximately describes the oscillatory behavior and decay (or growth) of the measurement *ϕ*_*ε*_(*x*) over time through its powers *λ*^*n*^. Smaller values of *ε* correspond to longer timescales where this approximation is valid. For *ε* = 0, *ϕ*_*ε*_ becomes an exact eigenfunction of ${{{\mathcal{K}}}}$ with eigenvalue *λ*.

Approximate eigenfunctions also encode key dynamical features of the system, such as the global stability of equilibria. The contours (or level sets) of these functions highlight key structures in the system’s dynamics, such as regions that behave independently over long times, pathways along which the system evolves, and surfaces where states settle into long-term behavior at the same rate^20,50^. The approximate point spectrum of ${{{\mathcal{K}}}}$, denoted ${{{{\rm{Sp}}}}}_{{{{\rm{ap}}}}}({{{\mathcal{K}}}})$, is the set of all scalars *λ* for which *ε* can be made arbitrarily small, and forms the most fundamental spectral property of Koopman operators.

Just as a matrix is diagonalized by its eigenvalues and eigenvectors, a nonlinear system can be “diagonalized” by its Koopman spectra. Koopman eigenfunctions act as fundamental components of behavior, revealing persistent patterns (like oscillations or trends) in the system. For a vector of observations ${{{\bf{g}}}}\in {{\mathbb{C}}}^{N}$, the Koopman mode associated with an eigenvalue is the projection of **g** onto the corresponding eigenspace. Under suitable conditions, this yields a spectral expansion for the time evolution of the observables^7^:

6 $${{{\bf{g}}}}({x}_{n})={\sum }_{j=1}^{\infty }{\lambda }_{j}^{n}{\phi }_{j}({x}_{0}){{{{\bf{g}}}}}_{j}.$$

Here, ${{{{\bf{g}}}}}_{j}\in {{\mathbb{C}}}^{N}$ is the *j*th Koopman mode, associated with eigenvalue *λ*_*j*_ and eigenfunction *ϕ*_*j*_, which one may think of as an expansion coefficient. This decomposition is conceptually and operationally similar to separation of variables: the eigenvalues describe time evolution through the powers ${\lambda }_{j}^{n}$, while the Koopman modes capture how each pattern is expressed in space and the regions where this dynamical behavior occurs.

When the observable **g** is real-valued, the eigenvalues, eigenfunctions, and Koopman modes in Equation (6) appear in complex-conjugate pairs. To illustrate how this affects time evolution, consider one such pair: ${\lambda }_{1}^{n}{\phi }_{1}({x}_{0}){{{{\bf{g}}}}}_{1}+{\overline{{\lambda }_{1}}}^{n}\overline{{\phi }_{1}({x}_{0})}\overline{{{{{\bf{g}}}}}_{1}}.$ Writing *λ*_1_ = *re*^*iθ*^ and *ϕ*_1_(*x*_0_)**g**_1_ = **R***e*^*i***Θ**^, the pair becomes

7 $${\lambda }_{1}^{n}{\phi }_{1}({x}_{0}){{{{\bf{g}}}}}_{1}+{\overline{{\lambda }_{1}}}^{n}\overline{{\phi }_{1}({x}_{0})}\overline{{{{{\bf{g}}}}}_{1}}=2{{{\bf{R}}}}{r}^{n}\cos (n\theta+{{{\boldsymbol{\Theta }}}}).$$

This expression reveals key dynamical features: *r*^*n*^ controls exponential growth (*r* > 1), decay (*r* < 1), or neutral evolution (*r* = 1); the angle *θ* sets the oscillation frequency; and the Koopman mode (e.g., through **R**) encodes spatial structure.

Methods for learning ${{{{\rm{Sp}}}}}_{{{{\rm{ap}}}}}({{{\mathcal{K}}}})$ and spectral expansions from data face significant challenges, including spurious eigenvalues (Fig. 1), missing critical spectral components, and numerical instabilities. Recent advances mitigate these issues^37^, but there is a critical need to develop a deeper theoretical understanding of the conditions under which such spectral computations are feasible. Equally important is identifying scenarios where these computations are fundamentally impossible. We shall see that addressing these questions guides the development of robust and reliable methods.

We first present three illustrative examples that demonstrate the reliability, advantages, and broad applicability of our general-purpose learning algorithms: (i) a classic oscillator where previous methods diverge but ours converges, (ii) a fluid flow with continuous spectrum where we extract meaningful eigenvalues, and (iii) an Arctic sea ice dataset where we uncover physically interpretable modes with error bounds and achieve state-of-the-art forecasts. We then examine the underlying theoretical foundations, showing that our algorithms optimally match fundamental limits revealed by adversarial dynamical systems. Indeed, analyzing the reasons behind these fundamental limitations enabled us to pinpoint the essential algorithmic properties required for reliable convergence. Finally, we provide a unified classification of the complexity of these learning problems, clarifying precisely when learning is possible and when it is fundamentally impossible.

### Overcoming lack of convergence in current methods

We begin with a simple low-dimensional system. Figure 1 (top) examines the classical Duffing oscillator in two distinct regimes: the undamped case (conservative, panel (a)) and the damped case (dissipative, panel (b)).

EDMD, widely regarded as the state-of-the-art, constructs a finite *N* × *N* matrix approximation of the Koopman operator by projecting onto *N* trial functions (a ‘dictionary’ of observables). We use a common and effective choice of dictionary: radial basis functions centered via *k*-means clustering^33^. In contrast to computing eigenvalues of an approximation, our method (Supplementary Algorithm 1) searches for approximate eigenfunctions where *ε* in Eq. (5) is locally minimized, with guaranteed convergence to the spectrum as *N* increases. The number of observables *N* grows with the number of snapshots *M*. Details of the experimental setup are in the “Methods”.

Panel (c) shows that EDMD does not converge as the matrix dimension *N* increases, instead generating numerous spurious eigenvalues due to truncation from the infinite-dimensional Koopman operator to a finite observable subspace. In contrast, our algorithm (using the same data and dictionary) converges reliably across dynamical regimes. Importantly, even without access to ground truth, it provides a principled error bound and enables validation of the chosen observable dictionary, a critical practical consideration given that computations necessarily involve finitely many observables. In the “Methods”, we further demonstrate the same behavior across a broad range of examples.

### Eigenvalue extraction in a chaotic flow

Figure 1 (bottom) shows the application of our algorithms to *Re* = 19,000 cavity flow, a regime exhibiting signatures of both discrete and continuous Koopman spectral components. In such cases, the Koopman representation involves an integral over a continuous spectrum in addition to the discrete sum in Eq. (6)^7,51^. Discrete Koopman eigenvalues typically signal quasiperiodic motion, whereas broadband spectral content is indicative of chaotic dynamics^52^. The combined spectral structure reflects the underlying geometry of the attractor in state space. Systems displaying both discrete and continuous spectral components are often described as skew-periodic^53^, with one component evolving periodically and the other exhibiting phase-modulated chaotic behavior.

Our algorithm (see “Methods”) successfully extracts eigenvalues and separates spectral components using a two-limit approach: adaptively increasing a time lag for autocorrelations to detect spectrally localized observables and expanding projections onto increasing finite-dimensional subspaces. This is grounded in linking quasiperiodic dynamics and the corresponding eigenvalues (the so-called RAGE theorem^54^, see Eq. (14)). This foundation ensures that the separation of the continuous spectrum and eigenvalues is both mathematically rigorous and practically optimal.

Physically, the evolution of Koopman spectra with increasing Reynolds number illustrates a transition, in which a chaotic state arises after one or two bifurcations (sudden qualitative changes in the system’s behavior) from a stable steady flow. The Koopman spectrum offers a powerful tool for analyzing bifurcations, quantifying energy in both quasiperiodic and continuous components, and links to the geometry of the state space and the flow domain (Supplementary Figs. 6–8).

### Physical modes for Arctic sea ice concentration

We consider monthly Arctic sea ice concentration satellite data from 1979–2021. The data are defined on a 432 × 432 grid of 625 km^2^ cells. Details on data collection and processing are provided in the “Methods”. As discussed in the introduction, Arctic sea ice is a critical component of the climate system, notorious for its complex dynamics. In this setting, Koopman eigenvalues reveal the timescales of changes in sea ice cover, while the corresponding modes indicate the spatial pattern of these changes. Notably, Koopman methods require no model: measurements of the concentration suffice to compute the eigenvalues and modes.

We first analyze the entire dataset and approximate Koopman eigenvalues using EDMD (Fig. 2). Similar to Fig. 1, our method provides error bounds for eigenpairs, shown in the plot. While many EDMD eigenvalues are spurious, several (shown in blue) have small errors and correspond to key dynamics: the annual mean sea ice (*λ* = 1); yearly growth-melt cycle captured by a fundamental monthly mode ($\lambda \approx \exp (\pi i/6)$); and decaying modes (∣*λ*∣ < 1). The small errors of the hidden modes indicate strong coherency and forecasting power (see Eq. (5)).

The decaying modes, “hidden” behind a sea of spurious modes but nonetheless revealed by our error bounds, are particularly interesting as they reflect dissipative dynamics. The corresponding Koopman eigenfunction time series (computed separately over 1980–1999 and 2000–2019 to assess robustness) suggests a connection to long-term sea-ice decline associated with climate change (Supplementary Fig. 9). This connection is likely nuanced: dissipative Koopman eigenfunctions exhibit reduction (as well as oscillation) as time increases, whereas observed sea-ice loss rates over recent decades are known to be non-monotonic. Since the full sea-ice state is expressed as a sum of modes, the near-monotonic behavior of *ϕ*_decay_ does not contradict non-monotonic trend patterns in Fig. 3.

Hidden modes with nonzero $\arg (\lambda )$ can be interpreted as seasonal patterns modulated by this long-term trend. In other words, these represent seasonal oscillations that gradually decay over decades. Similar behavior has also been reported for Koopman eigenfunctions computed from sea-surface temperature data^29^. Our modes capture both the oscillation and the long-term decay. If *ϕ*_decay_ is the decaying eigenfunction with zero complex argument and *ϕ*_var_ an annual variation mode with argument *π*/6, then other decaying eigenfunctions are approximately given by ${\phi }_{{{{\rm{decay}}}}}\times {[{\phi }_{{{{\rm{var}}}}}]}^{j}$. This structure arises from the multiplicative property of Koopman eigenfunctions: if ${{{\mathcal{K}}}}{g}_{1}={\lambda }_{1}{g}_{1}$ and ${{{\mathcal{K}}}}{g}_{2}={\lambda }_{2}{g}_{2}$, then ${{{\mathcal{K}}}}({g}_{1}{g}_{2})={\lambda }_{1}{\lambda }_{2}{g}_{1}{g}_{2}$, which follows from Eq. (3). For example, the canonical correlations between these decaying modes and the underlying modulated monthly dynamics are 0.9807 and 0.9798 for modes with arguments *π*/6 and *π*/3, respectively. In the climate science literature, such seasonally modulated product modes are sometimes referred to as “combination modes”^25,55^.

The mean decay time, $-1/\log (| \lambda | )$, for these modes is 233 months, consistent with decay times observed in the Antarctic region^56^. Notably, this decay rate was not observed in the Arctic region using DMD methods in ref. ^56^, which we attribute to the challenges of extracting reliable eigenpairs in the absence of error bounds. Several studies indicate nonlinear trends in the decline of sea ice^57^, which further advocates the use of Koopman operator techniques to disentangle the complex nonlinear dynamics.

We compute Koopman modes (**g**_*j*_ in Eq. (6) where **g** is the vector of sea ice concentrations), which capture spatial and temporal patterns in sea ice concentration not easily discernible by conventional methods. Although the modes share units with the input data, they may take values above 100% due to the non-orthogonal expansion in Equation (6). Each mode highlights geographic regions where sea ice exhibits oscillatory, growing, or decaying behavior, as indicated by its corresponding eigenvalue. For example, modes with one-year oscillations reflect the seasonal cycle, eigenvalues near one capture the mean state, and multi-year or slowly varying modes indicate long-term trends, with the associated modes highlighting the regions where these changes occur.

The hidden decaying mode is concentrated in the Barents and Kara Seas, implying that the decrease in sea ice concentration over these timescales is localized to those regions. This is consistent with observations in ref. ^56^. Indeed, there are links between sea ice reduction in these regions and extreme weather, such as severe winters in central Eurasia^58^. Importantly, our method provides error estimates, giving confidence that these identified modes are real and not artifacts (unlike many spurious modes given by EDMD).

These hidden modes also capture seasonally-modulated reemergence of correlations (Supplementary Fig. 10), revealing ‘memory’ in the climate system, whereby sea ice anomalies occurring during the growth season reemerge in the following melt season despite a loss of correlation in the intervening winter months^59^. For studies of this phenomenon using kernel methods, see refs. ^60,61^. Such long-lived modes and their geographic foci are critical, as they could influence large-scale climate patterns (e.g., ocean circulation changes) and have practical implications for shipping routes and climate resilience. Further work is needed to perform data-driven prediction of changes due to tipping points such as the greater mixing between the Barents Sea and North Atlantic^62^. Modes in sea ice can significantly influence the onset or prevention of tipping behavior of AMOC patterns^43,44^.

Sea ice decay is commonly assessed by sea ice extent, the area covered by grid cells with sea ice concentration exceeding 15%, shown in Fig. 3. We compute the Koopman modes corresponding to the mean sea ice concentration and the annual variation over ten-year periods (1980–1989, 1990–1999, 2000–2009, 2010–2019) and plot their absolute values. The mean mode reveals a clear decline in overall sea ice and reduced winter extent. Annual modes show a geographic shift in seasonal variability, with marginal seas (particularly the Beaufort, Kara, and nearby coastal regions) exhibiting increased amplitude, indicating stronger seasonal contrast in these regions. The overall decline in concentration suggests a slow decaying mode, consistent with the spectrum in Fig. 2.

### Arctic sea ice forecasting

We now address the problem of forecasting, focusing first on the challenging task of reconstructing sea ice concentration at active grid points. Forecasts are initialized monthly from 2005 to 2015, each with a 3-year horizon. Training data consists of observations from 1979 up to the month preceding initialization, yielding 132 distinct forecast trajectories. To obtain accurate forecasts, we truncate the Koopman mode decomposition using spectral approximations and error bounds computed by our algorithm (Eq. (20) in “Methods”). We compare our method to EDMD with delay embedding, which has previously shown strong performance in Arctic sea ice forecasting^56^. We also benchmark against two baselines: the monthly climatology at each grid point (referred to as the periodic baseline) and the monthly persistence model. Forecast errors are measured relative to the climatological variance of the periodic baseline (see “Methods”) and averaged over all forecast trajectories. Figure 4a shows the results. Our approach outperforms EDMD, particularly at long lead times, consistent with its explicit minimization of *ε* in Eq. (5).

We next consider a binary classification problem and compare our approach to IceNet^63^, a deep learning model that provides state-of-the-art six-month sea ice forecasts. A point is classified as open water if the sea ice concentration is below 15%, the standard threshold for defining the ice edge. Following the setup in ref. ^63^, we evaluate monthly forecasts from 2012 to 2020, with lead times from 1 to 6 months (chosen to align with the lead times for which IceNet produces forecasts). Binary accuracy is defined as the percentage of predicted classes matching the observations. We also compare against SEAS5^64^, a leading dynamical model from the European Center for Medium-Range Weather Forecasts. Figure 4b shows the mean binary accuracy across lead times. Beyond one-month forecasts, the proposed verified Koopman model consistently outperforms both benchmarks, with significantly greater accuracy as lead time increases. This robustness arises from the method’s direct minimization of *ε*, yielding longer coherency and predictive timescales (see Eq. (5)).

This improvement is achieved at a fraction of the computational cost and with significantly fewer parameters than deep learning approaches. IceNet uses 4.4 × 10^7^ trainable weights and requires over a day to train on a NVIDIA Quadro P4000 GPU, while the Koopman approach relies on an observable space of at most 510 dimensions (260100 parameters) and is trained on a laptop in under a second.

We found no clear relationship between prediction accuracy and El Niño events. Figure 4c, d presents average accuracy by calendar month and the improvement achieved by our method. Accuracy declines markedly during summer, consistent with the well-known “spring predictability barrier,” which affects forecasting models owing to the influence of melt-season ice thickness. These months also exhibit the largest performance gains relative to SEAS5. Finally, even as *ε↓*0 with increasing data, predictability remains fundamentally constrained by atmospheric chaos and observational noise^65^.

These practical advantages motivate a return to our central question: *When can system behavior be learned reliably from data, and when is such learning impossible?* To address this, we present our theoretical results, with full proofs provided in the Supplementary Information.

### Adversarial systems reveal challenges

We first construct adversarial dynamical systems that expose two fundamental challenges in computing Koopman spectra. These establish lower bounds on problem complexity. Even with infinite data, no algorithm can guarantee learning certain system behaviors, providing an explicit demonstration of inherent limits in data-driven dynamical system learning.

While it is sometimes possible to design algorithms that work for a single known system, this does not reflect the goals of general-purpose learning or complexity classification. Instead, our focus is on identifying the minimal assumptions under which an algorithm can reliably learn across a broad class of systems, a core aim in both computational complexity and ML. To illustrate, let ${\Omega }_{{\mathbb{D}}}$ be the class of systems that are continuous, measure-preserving, and invertible on the unit disk in two dimensions. (A system is measure-preserving if it preserves a volume on the state space ${{{\mathcal{X}}}}$ during its evolution, e.g., an idealized frictionless system. Such systems are ubiquitous, including classical Hamiltonian systems^66^, physical systems in equilibrium^67^, and the post-transient behavior of many systems^7^.) Let Ω_[0, 1]_ be the class of smooth, invertible *F* on the interval [0, 1] with uniform bounds on their derivatives (not necessarily measure-preserving).

Our results (Theorems 2.6 and 2.9 of the Supplementary Information) show that for either of these classes (denoted collectively as Ω) no deterministic algorithms *Γ*_*n*_ exist that, using snapshot data, converge to ${{{{\rm{Sp}}}}}_{{{{\rm{ap}}}}}({{{{\mathcal{K}}}}}_{F})$ for all *F* in the class Ω as *n* → *∞*. Furthermore, for any probabilistic learning algorithms, the probability of convergence cannot exceed 50%. These impossibility results are universal, applying to any type of algorithm and regardless of what *n* represents. Hence, for any algorithm, simply increasing the number of data points *M* → *∞* will not lead to convergence, as this would correspond to an instance of the sequence *Γ*_*n*_. These constructive results reveal fundamental challenges that occur across data-driven dynamical systems:

- **(C1)**: For systems in ${\Omega }_{{\mathbb{D}}}$, the challenge lies in determining when enough data has been collected to approximate the action of ${{{\mathcal{K}}}}$ on a given observable, e.g., by approximating the averages in Eq. (1). The convergence rate is problem dependent^68^: no universal rate exists^69^.
- **(C2)**: For systems in Ω_[0, 1]_, the difficulty stems from the non-normality of ${{{\mathcal{K}}}}$ (non-orthogonality of its eigenfunctions). This is a well-known challenge in spectral approximation more generally^70^, and manifests itself in the Koopman context as the difficulty in distinguishing data corresponding to transient dynamics from post-transient dynamics.

Moreover, these challenges:

- cover randomized algorithms, e.g., random trajectory sampling, or training with probability distributions over data, as in stochastic gradient descent and other ML methods;
- hold whatever the distribution of data;
- hold for any type of computer, e.g., digital computation (Turing machines) or exact arithmetic (BSS machines);
- hold even if we consider smoother *F* and allow our algorithms to sample the derivatives of *F* as well as Eq. (4).

The system classes for which we construct adversaries include widely studied examples such as measure-preserving flows and smooth interval exchange maps. The mechanisms can also be embedded in higher dimensions and other state spaces: the impossibility result holds for any class of systems satisfying **(C1)** or **(C2)**. These mechanisms are not limited to Koopman spectral estimation and reflect general challenges in data-driven dynamical systems.

### A universal algorithm with learning guarantees

Learning from the above results, we now show that Koopman spectra can be computed from trajectory data, provided two key conditions are satisfied. Together with the other algorithms discussed below, these show upper bounds on problem complexity. This result also resolves the fundamental open problem of data-driven computation of ${{{{\rm{Sp}}}}}_{{{{\rm{ap}}}}}({{{{\mathcal{K}}}}}_{F})$.

To address challenge **(C1)**, we assume the system is measure-preserving (this can be relaxed in many dissipative cases). To address **(C2)**, we require some control over the smoothness of the dynamical map *F*. Specifically, we assume *F* has a known modulus of continuity *α*. This function controls the distance between *F*(*x*) and *F*(*y*) by the distance between states *x* and *y*. Although such a function always exists, the impossibility result above shows that without knowledge of *α*, one cannot compute Koopman spectra in a single limit.

Let ${\Omega }_{{{{\mathcal{X}}}}}^{\alpha,m}$ be the class of systems satisfying both conditions. For such systems, we have developed deterministic learning algorithms *Γ*_*n*_ that reliably approximate the system’s dynamics using snapshot data (Theorem 2.3 and Algorithm 1 of the Supplementary Information). These algorithms converge to ${{{{\rm{Sp}}}}}_{{{{\rm{ap}}}}}({{{{\mathcal{K}}}}}_{F})$ for all *F* in the class ${\Omega }_{{{{\mathcal{X}}}}}^{\alpha,m}$ as *n* → *∞*. They also provide explicit error bounds that verify the accuracy of the approximation.

Our analysis of **(C1)** and **(C2)** directly yields a provably convergent algorithm, in contrast to EDMD, which does not converge in general. The central idea (see “Methods”) is to use the modulus of continuity to adaptively select the dictionary size *N* as a function of the available data *M*, within an averaging framework similar to Eq. (1). Unlike EDMD, we do not compute eigenvalues of a finite-dimensional matrix. Instead, the adaptive procedure constructs a correlation matrix from which we evaluate the error metric shown in Fig. 1. We then identify local minimizers of this metric and, using the measure-preserving structure of the system (or resolvent bounds, e.g., in dissipative settings), relate it to the distance between a point $z\in {\mathbb{C}}$ and the spectrum. This distance is computed together with an associated approximate eigenfunction satisfying the coherency condition Eq. (5), with *ε* equal to the estimated spectral distance.

Figure 1 demonstrates the convergence of this algorithm applied to the Duffing oscillator (see also the further systems in the “Methods”). Moreover, the error bounds achieved by the algorithm enabled improved sea ice forecasts in Fig. 4.

### To infinity and beyond

Surprisingly, spectral properties can still be learned in the presence of **(C1)** and **(C2)** by adjusting the approach. Instead of requiring a single data limit as *n* → *∞*, we consider separate successive limits for key parameters. Each is tied to a different type of data, such as increasing dataset size, measurement resolution, or dictionary complexity.

For instance, for EDMD (see “Methods”) without the modulus of continuity, one must first take the number of data samples *M* to infinity to approximate correlations and only then the number of observables *N* to infinity. (Even then, EDMD may fail to converge. Convergence of EDMD eigenvalues can be established along a subsequence, provided the weak limit of the associated eigenfunctions is non-zero^31^. Verifying this condition, however, entails multiple limiting procedures in the sense of the SCI described below.) The convergence rate of time-averaged quantities such as correlations depends sensitively on the system’s mixing properties and the regularity of the observable. While polynomial rates hold for certain strongly mixing systems^68,71^, Birkhoff’s theorem guarantees only convergence without any uniform rate and time averages can converge arbitrarily slowly^69^,Chapter 2. This non-uniformity prevents the formulation of a universal convergence rate or a general-purpose *N*(*M*) strategy, unless we can control **(C1)**.

To further understand how assumptions on a system’s structure influence our ability to learn from data, we establish results under three scenarios (see Theorem 2.3 of the Supplementary Information):

– First, let ${\Omega }_{{{{\mathcal{X}}}}}^{\alpha }$ denote systems whose dynamics have a known smoothness quantified by a modulus of continuity *α*. For these systems, there exist learning algorithms that depend on two parameters, *n*_1_ and *n*_2_, and reliably use snapshot data to approximate the dynamics. These algorithms converge in two successive data limits (*n*_1_ → *∞* and then *n*_2_ → *∞*) to the spectrum ${{{{\rm{Sp}}}}}_{{{{\rm{ap}}}}}({{{{\mathcal{K}}}}}_{F})$ for all systems *F* in ${\Omega }_{{{{\mathcal{X}}}}}^{\alpha }$.

– Second, let ${\Omega }_{{{{\mathcal{X}}}}}^{m}$ denote measure-preserving systems (this assumption can be relaxed using resolvent bounds) with continuous dynamics. Algorithms again exist that converge to ${{{{\rm{Sp}}}}}_{{{{\rm{ap}}}}}({{{{\mathcal{K}}}}}_{F})$, but require two successive data limits for all systems in this class. The necessity of these successive limits arises naturally: in Eqs. (8) and (9), challenge **(C1)** implies that convergence rates of the underlying sums cannot be uniformly controlled^69^, a phenomenon well known in mixing systems, among others. One must therefore first take the large-data limit (*M* → *∞*), followed by the dictionary limit (*N* → *∞*); in general, these limits cannot be combined or reversed. Our analysis further shows that, for any algorithm (not only EDMD), no universal rule linking *M* and *N* guarantees convergence when both grow simultaneously.

– Finally, consider the most general scenario, ${\Omega }_{{{{\mathcal{X}}}}}$, consisting of all continuous systems without further assumptions. While the continuity of *F* can be relaxed, it is often assumed because discontinuities can lead to pathologies^47^. Remarkably, reliable algorithms still exist, but they require three successive data limits to converge to the spectrum ${{{{\rm{Sp}}}}}_{{{{\rm{ap}}}}}({{{{\mathcal{K}}}}}_{F})$ for all *F* in ${\Omega }_{{{{\mathcal{X}}}}}$: increasing the number of snapshots (*M* → *∞*), expanding the dimensionality of the subspaces (*N* → *∞*), and refining coherence estimates into spectra (taking the regularization parameter *ε↓*0 in Eq. (5)). Without assumptions on the underlying system, no simpler two-step algorithm can achieve guaranteed convergence (Theorem 2.15 of the Supplementary Information). **(C1)** and **(C2)** each cost a limit, illustrating the intrinsic difficulty of learning general dynamical systems.

### Unified complexity classifications of learning

The Solvability Complexity Index (SCI)^72,73^ formalizes how many limits are needed to learn properties from data. For the full class ${\Omega }_{{{{\mathcal{X}}}}}$, computing ${{{{\rm{Sp}}}}}_{{{{\rm{ap}}}}}({{{{\mathcal{K}}}}}_{F})$ requires three separate limits, which cannot be reduced or combined: no method can succeed with only two. For structured subclasses like ${\Omega }_{{{{\mathcal{X}}}}}^{\alpha,m}$, ${\Omega }_{{{{\mathcal{X}}}}}^{\alpha }$, or ${\Omega }_{{{{\mathcal{X}}}}}^{m}$, the above shows that fewer limits suffice.

**Fig. 5:**
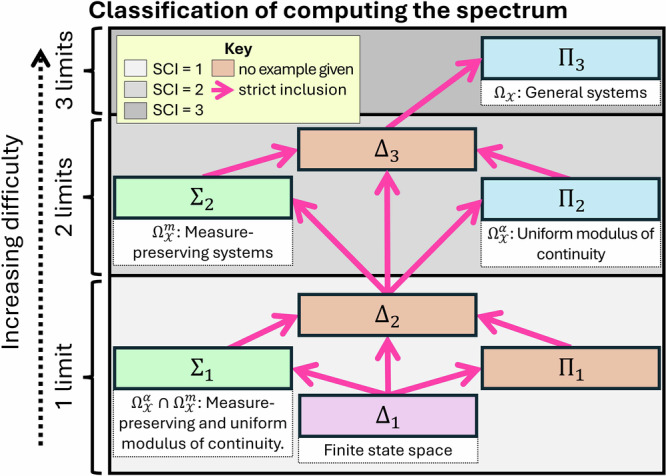
Classifications for learning Koopman spectra from trajectory data. Each SCI level indicates that solving the problem requires more complex, layered procedures. Each `limit' reflects an extra data refinement. Our results establish both upper bounds (via convergent algorithms) and lower bounds (through adversarial dynamical systems) on the computational complexity of these problems. The class Δ_*m*+1_ comprises problems with SCI≤*m*. The Σ and Π classes characterize how verification occurs in the final limit when learning Koopman operator spectra: Σ corresponds to convergence from within, and Π to convergence from above. For finite state spaces, the problem lies in Δ_1_, since the Koopman operator reduces to a finite-dimensional matrix whose spectrum can be computed by a single convergent algorithm with explicit rates.

This framework provides a systematic way to assess the complexity of data-driven problems. Applied to Koopman operators, Fig. 5 summarizes the difficulty of learning their spectra based on our results. Each classification includes both an upper bound (a convergent algorithm), and a lower bound, established by constructing adversarial dynamical systems to prove that fewer limits are insufficient. We can also relate existing algorithms to the SCI hierarchy by summarizing convergence results from the Koopman literature and their corresponding implicit upper bounds in Table 1. Each algorithm relies on specific system assumptions and, in some cases, uses more data limits than necessary for convergence.

**Table 1:** Convergence results for Koopman operators in the SCI hierarchy

Algorithm	Comments/Assumptions	Spectral Problem’s Corresponding SCI Upper Bound			
		${{{\mathcal{K}}}}g$	Spectrum	Spectral Measure (if m.p.)	Spectral Type (if m.p.)
Extended DMD^33^	general *L*^2^ spaces	SCI≤2^*^	N/C	N/C	n/a
Residual DMD^37^	general *L*^2^ spaces	SCI≤2^*^	SCI≤3^*^	SCI≤2^*^	varies, see ref. ^97^
					e.g., a.c. density: SCI≤2^*^
Measure-preserving EDMD^98^	m.p. systems	SCI≤1	N/C	SCI≤2^*^ (general)	n/a
				SCI≤1 (delay-embedding)	
Hankel DMD^99^	m.p. ergodic systems	SCI≤2^*^	N/C	N/C	n/a
Christoffel–Darboux kernel^36^	m.p. ergodic systems	SCI≤3	n/a	SCI≤2	e.g., a.c. density: SCI≤2
Generator EDMD^100^	cts.-time, samples ∇ *F*	SCI≤2	N/C	SCI≤2	n/a
	(otherwise additional limit)				
Compactification^16^	cts.-time, m.p. ergodic systems	SCI≤4	N/C	SCI≤4	n/a
Resolvent compactification^49^	cts.-time, m.p. ergodic systems	SCI≤5	N/C	SCI≤5	n/a
Diffusion maps^9^	cts.-time, m.p. ergodic systems	SCI≤3	n/a	n/a	n/a

This table summarizes convergence results from the Koopman literature, interpreted through the lens of the Solvability Complexity Index (SCI). “N/C” denotes non-convergence without additional strong assumptions (e.g., requiring observables to lie within a finite-dimensional invariant subspace), “n/a” indicates the method is not applicable to the spectral problem, and “m.p.” stands for measure-preserving systems. A superscript ^*^ indicates the SCI bound improves by one if errors in approximations of correlations (see Eqs. (8) and (9)) are controlled, e.g., through known variational bounds on *F*. This reduction depends on system properties^68^. The column ${{{\mathcal{K}}}}g$ refers to approximating ${{{\mathcal{K}}}}$’s action on observables *g*. Note that computing the spectral measure (distribution of the system’s behavior across different frequencies) may not yield the spectrum due to spurious eigenvalues. Upper bounds typically assume access to a dictionary with projections converging strongly to the identity; this construction is either specified (e.g., the entries “compactification methods” and “diffusion maps” have the distinct advantage of learning a well-conditioned dictionary) or required as an additional input by the user (e.g., EDMD and generator EDMD) which may or may not increase the SCI. In our upper bounds in Fig. 5, we construct a dictionary. N.B. These results are upper bounds on the SCI. Many of these bounds are not sharp, meaning they overestimate the number of limits required.

Beyond Table 1, Ulam’s method is widely used to approximate eigenvalues of transfer operators, which describe the evolution of probability densities and are dual to Koopman operators. It typically requires two successive limits: one for Monte Carlo approximation and another for increasing matrix size^74^, but convergence is not always reliable^75^, Section 2.6. Adding a third limit via noise smoothing can improve convergence^75^, though adaptive noise selection sometimes reduces this back to two. Similar SCI classifications apply to other data-driven dimensionality reduction methods^76^. For multiple limits in control theory, see ref. ^77^,Theorem 3.

These examples illustrate that multiple-limit phenomena are central to many data-driven methods in dynamical systems. While methods provide SCI upper bounds, a key challenge is determining whether these bounds are optimal. This raises fundamental questions: Can convergence be achieved with fewer limits? If not, what assumptions make it easier? To address these, we have derived lower bounds that show how system properties and the quantities being computed shape optimal algorithm design. When upper and lower bounds match, the algorithm is provably optimal for the problem.

In short, we now have a characterization of when data-driven spectral learning can succeed and when it cannot.

### Learning eigenpairs and latent spaces is hard

As a final problem, we consider the complexity of determining the spectral type, i.e., eigenvalues versus continuous components (bottom panel of Fig. 1), of measure-preserving systems. Spectral types distinguish between recurrent patterns (periodic or quasiperiodic oscillations) and more chaotic or mixing behavior, based on how the system spreads energy over frequencies^78^ page 45. They play a key role in applications such as fluid mechanics^79^, anomalous transport^80^, and analysis of trajectory invariants and exponents^81^, and are particularly important in reduced-order modeling^7,82^. We discussed their role in the above cavity flow problem.

Identifying eigenvalues and eigenfunctions of Koopman operators (*ε* = 0 in Eq. (5)) reveals coordinates in which nonlinear dynamics evolve linearly, enabling the expansion in Eq. (6). Non-unit eigenvalues (*λ* ≠ 1) encode time-varying structure. Let Ω_*p*_ denote the class of smooth, invertible, measure-preserving systems on the torus with uniformly bounded derivatives. This class lies within ${\Omega }_{{{{\mathcal{X}}}}}^{\alpha,m}$; consequently, our universal one-limit algorithm applies and provably computes the full spectrum-without requiring any a priori distinction between discrete and continuous components.

Strikingly, even for this well-structured class, no single-limit learning algorithm (deterministic or probabilistic with success probability exceeding 50%) based on trajectory data can determine whether the Koopman operator ${{{{\mathcal{K}}}}}_{F}$ admits a non-unit eigenvalue for *F* in Ω_*p*_. Likewise, no such algorithm can converge to the set of eigenvalues (Theorem 2.12 of the Supplementary Information).

**Fig. 6:**
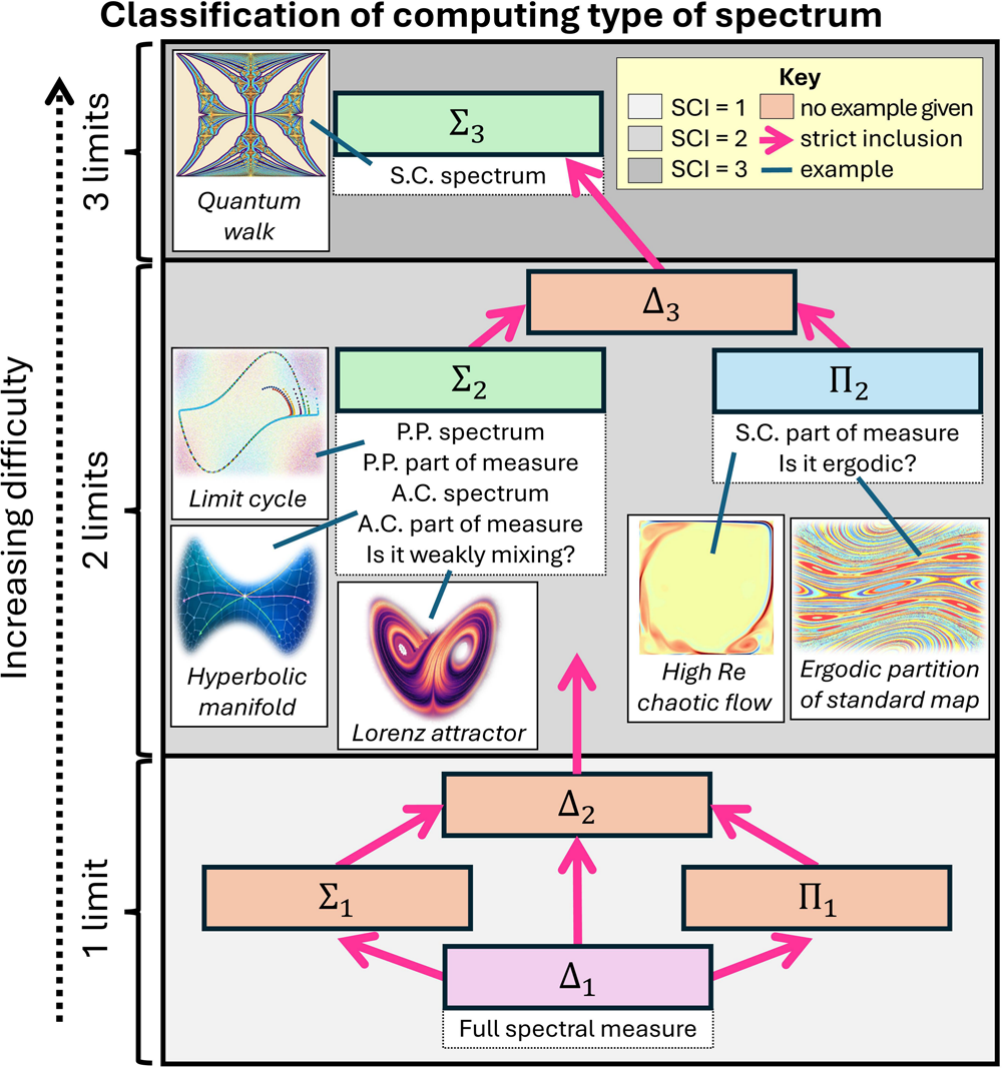
Classifications for learning spectral types (eigenvalues (p.p.), absolutely continuous (a.c.) and singular continuous (s.c.)) of Koopman operators for measure-preserving invertible systems from trajectory data. Each classification comprises an upper bound (convergent algorithms) and a lower bound (established by constructing adversarial dynamical systems). The SCI is the number of data limits needed to solve a problem. For each learning objective, we provide a representative system to emphasize that the classification fundamentally depends on the nature of the underlying dynamics. These examples illustrate how different dynamical behaviors influence the complexity of learning spectral types from data. The various classes Δ_*m*_, Σ_*m*_, and Π_*m*_ are described in Fig. 5.

However, both problems become computable through two interdependent data limits (Supplementary Algorithms 6 and 7): adaptively adjusting the time lag in autocorrelation estimates according to data size, and increasing projections onto finite-dimensional subspaces defined by a dictionary. We demonstrated this strategy on a complex fluid flow (see also “Methods”). This two-limit procedure is provably optimal: no single-limit algorithm can resolve these questions. The corresponding SCI classifications are summarized in Fig. 6.

These results help explain the challenges in finding finite-dimensional representations, such as autoencoders or latent spaces, in which the dynamics appear linear^30^. In particular, it is fundamentally impossible to determine or verify the existence of Koopman eigenvalues using only a single limit. A constructive alternative is to compute approximate eigenfunctions associated with ${{{{\rm{Sp}}}}}_{{{{\rm{ap}}}}}({{{{\mathcal{K}}}}}_{F})$. This problem admits a single-limit algorithm with verification (Fig. 5), providing a sharp and practically implementable route for spectral analysis. We applied this to the Arctic sea ice dataset above.

### Discussion

We developed a powerful technique, the construction of adversarial dynamical systems (see § Adversarial systems and Fig. 9), to establish impossibility results in data-driven dynamical systems. We identified conditions (**(C1),** **(C2)**) under which no sequence of randomized algorithms (e.g., based on randomly sampled trajectories) can succeed with probability greater than 50%. These failures are not rare pathological cases, but reflect fundamental barriers intrinsic to the problem. These barriers, in turn, guided the development of a suite of algorithms (Supplementary Algorithms 1–7) that achieve optimal performance and are provably convergent. The resulting methods produce trustworthy, verifiable outputs and include what we term multi-data-limit methods (see Figs. 5, 6). By linking dynamical systems theory with the foundations of computation, we establish a computational complexity framework for data-driven dynamical systems. This framework clarifies the inherent limitations of using finite data to analyze complex dynamics while simultaneously identifying algorithmic possibilities with broad applicability. It provides a synthesis in ML between foundational theory and concrete application.

We demonstrated the power of our framework by solving a long-standing problem: computing Koopman spectra from data without spurious eigenvalues or missing components. For example, we show that there exists a constructive computational procedure for learning ${{{{\rm{Sp}}}}}_{{{{\rm{ap}}}}}({{{{\mathcal{K}}}}}_{F})$ from data for general continuous systems. Our algorithms succeed where existing methods (e.g., EDMD) struggle, recovering accurate spectra in both low- and high-dimensional systems, including cases with continuous spectra and a real-world application to Arctic sea ice.

Arctic amplification has accelerated sea ice loss, with major consequences for ecosystems, local communities, and extreme weather. A key challenge is identifying the geographic patterns driving these changes. Our algorithms uncover hidden Koopman modes linked to sea ice decline (Fig. 2), offering dynamic and spatial insight supported by verification. These decaying modes are physically meaningful and may guide future measurements. Using spectral approximations and error bounds to truncate spurious components, we built forecast models that achieve state-of-the-art accuracy at a fraction of the cost of deep learning models (Fig. 4 and surrounding discussion). Applications include optimizing shipping routes, reducing environmental risks, and informing early-warning systems. Understanding sea ice loss is also vital given potential links to extreme events such as wildfires, floods, heatwaves, and cold spells. The Koopman framework further enables systematic comparisons across climate models and supports the construction of interpretable response formulas and reaction coordinates near critical transitions, with broad implications for climatology^83^.

Our approach extends beyond Koopman operators, provided appropriate domain-specific “sudden change” lemmas can be established (see “Methods”). In each case, an analog of the “ball of learnability” applies. This perspective is relevant to a range of methods, including SINDy^10^, neural ODEs^84^, Fourier neural operators^85^, LSTMs^86^, and PDE-net^87^, as well as to other areas of scientific computing with ML. For example, recent work has shown that linear elliptic PDEs can be learned from input-output pairs^88^, analogous to snapshot-based learning. Whether similar approaches extend to hyperbolic or nonlinear PDEs remains open, but our proof techniques may offer insight into this challenge.

This paper initiates a broader effort to explore the limits of robust learning and to develop a theory of necessary and sufficient conditions. Several promising directions remain. First, we have assumed access to full-state observations in Eq. (4), whereas many applications involve only partial measurements of the state *x*. Extending our upper and lower bounds to such settings is a natural next step. Second, we focused on discrete-time systems, reflecting how data is typically collected. It would be valuable to study continuous-time dynamics and the sampling conditions needed for reliable learning. Our results should carry over under generic time discretizations, but a formal analysis remains open. Third, Koopman-based methods have shown promise in control problems across domains such as power grids^89^, robotics^24^, fluid dynamics^90^, chemistry^91^, and biology^92^, where the linearity of ${{{\mathcal{K}}}}$ enables tractable control design^93^. Our development of provably convergent, error-bounded algorithms for Koopman spectral properties opens the door to significant advances in nonlinear control. Fourth, it is natural to seek lower bounds complementing the upper bounds in Table 1 for problems that do not rely on spectral computations. These may have different lower bounds than those we established, but we anticipate that the adversarial dynamical systems framework can be extended to cover them.

ML in data-driven dynamical systems is rapidly expanding, and this momentum shows no signs of slowing. Across nearly every area, key challenges are being reexamined through big data and deep learning. With this surge of interest and innovation, it is crucial for the community to grasp not only what is possible but also what is fundamentally impossible. This prevents the pursuit of unattainable algorithms or methods, safeguards against potentially catastrophic errors, and reveals the conditions under which learning is feasible: upper and lower bounds inform and sharpen one another. This process led us to our convergent algorithms. Such classifications are essential if we are to fully harness the power of ML in dynamical systems.

## Methods

### Computing Koopman modes and EDMD

For a dictionary of observables ${\{{g}_{j}\}}_{j=1}^{N}$ and snapshot pairs (*x*^(*m*)^, *y*^(*m*)^) in Eq. (4), EDMD approximates two correlation matrices *G*_*ij*_ = 〈*g*_*j*_, *g*_*i*_〉 and ${A}_{ij}=\langle {{{\mathcal{K}}}}{g}_{j},{g}_{i}\rangle$ (*i*, *j* = 1, …, *N*) from data averages using ${{{\mathcal{K}}}}{g}_{j}({x}^{(m)})={g}_{j}({y}^{(m)})$, analogously to Eq. (1):

8 $${G}_{ij}\approx {\widehat{G}}_{ij}=\frac{1}{M}{\sum }_{m=1}^{M}{g}_{j}({x}^{(m)})\overline{{g}_{i}({x}^{(m)})},\,\,i,j=1,\ldots,N,$$

9 $${A}_{ij}\approx {\widehat{A}}_{ij}=\frac{1}{M}{\sum }_{m=1}^{M}{g}_{j}({y}^{(m)})\overline{{g}_{i}({x}^{(m)})},\,\,i,j=1,\ldots,N.$$

(Here, the *L*^2^ inner product 〈 ⋅ , ⋅ 〉 is a generalized dot product that measures how strongly two observables are correlated.) The Koopman approximation is the *N* × *N* matrix ${\widehat{G}}^{-1}\widehat{A}$ and EDMD computes its eigenvalues.

We compute the Koopman modes **g**_*j*_ appearing in Eq. (6) as follows. Given a collection of *n* approximate eigenfunctions (computed, e.g., by EDMD or our algorithms), let $\Psi \in {{\mathbb{C}}}^{M\times n}$ denote the matrix corresponding to their time series across the training data *x*^(*m*)^ (*m* = 1, …, *M*) in Eq. (4). For a vector of observations ${{{\bf{g}}}}\in {{\mathbb{C}}}^{N}$, let ${{{\bf{O}}}}\in {{\mathbb{C}}}^{M\times N}$ be their values over the same training data. The Koopman modes **g**_*j*_ are computed by

10 $${\left({{{{\bf{g}}}}}_{1}\,{{{{\bf{g}}}}}_{2}\cdots {{{{\bf{g}}}}}_{n}\right)}^{\top }={\Psi }^{{{\dagger}} }{{{\bf{O}}}},$$

where † denotes the Moore–Penrose inverse (solution of the least squares problem) and ⊤ the matrix transpose.

In Figs. 2 and 3, we have plotted the absolute values of Koopman modes since these describe spatial presence of the dynamics described by eigenvalues (as explained in the discussion surrounding Eq. (6)). The error bars for these Koopman modes and eigenvalues correspond to $\parallel {{{\mathcal{K}}}}{\phi }_{\varepsilon }-\lambda {\phi }_{\varepsilon }\parallel$ (computed using the function in Eq. (12)) for the corresponding approximate eigenfunction *ϕ*_*ε*_ (normalized so ∥*ϕ*_*ε*_∥ = 1). These provide an error bound for coherency in Eq. (5).

### Upper bounds: a suite of convergent algorithms

There are several algorithms, each corresponding to different classes of dynamical systems in Fig. 5, and full pseudocode is provided in the Supplementary Information. Recall from the Duffing oscillator example that we consider a dictionary of observables ${\{{g}_{j}\}}_{j=1}^{N}$ and that we have access to the snapshot data in Eq. (4).

We assume the state space is a compact metric space $({{{\mathcal{X}}}},{d}_{{{{\mathcal{X}}}}})$ and consider observables *g* in ${L}^{2}({{{\mathcal{X}}}},\omega )$, the space of functions whose squared values can be averaged (integrated) over ${{{\mathcal{X}}}}$ using a probability measure *ω*, which defines how different parts of the state space are weighted. We also assume that *F* is nonsingular with respect to *ω* (if *ω*(*E*) = 0 then *ω*({*x*: *F*(*x*) ∈ *E*}) = 0) and ${{{{\mathcal{K}}}}}_{F}$ is bounded. This is the standard setting in Koopman analysis, often referred to as the spectral study of the system. Notably, our methods apply to any compact metric space ${{{\mathcal{X}}}}$ and probability measure *ω*. To assess how well algorithms capture the spectrum of the Koopman operator, we use the Hausdorff metric, which measures how far two spectral sets are from each other by quantifying the greatest distance one must travel from a point in one set to reach the other. This metric ensures that the computed spectra converge accurately, avoiding errors such as including false eigenvalues or missing important spectral regions.

**Fig. 7:**
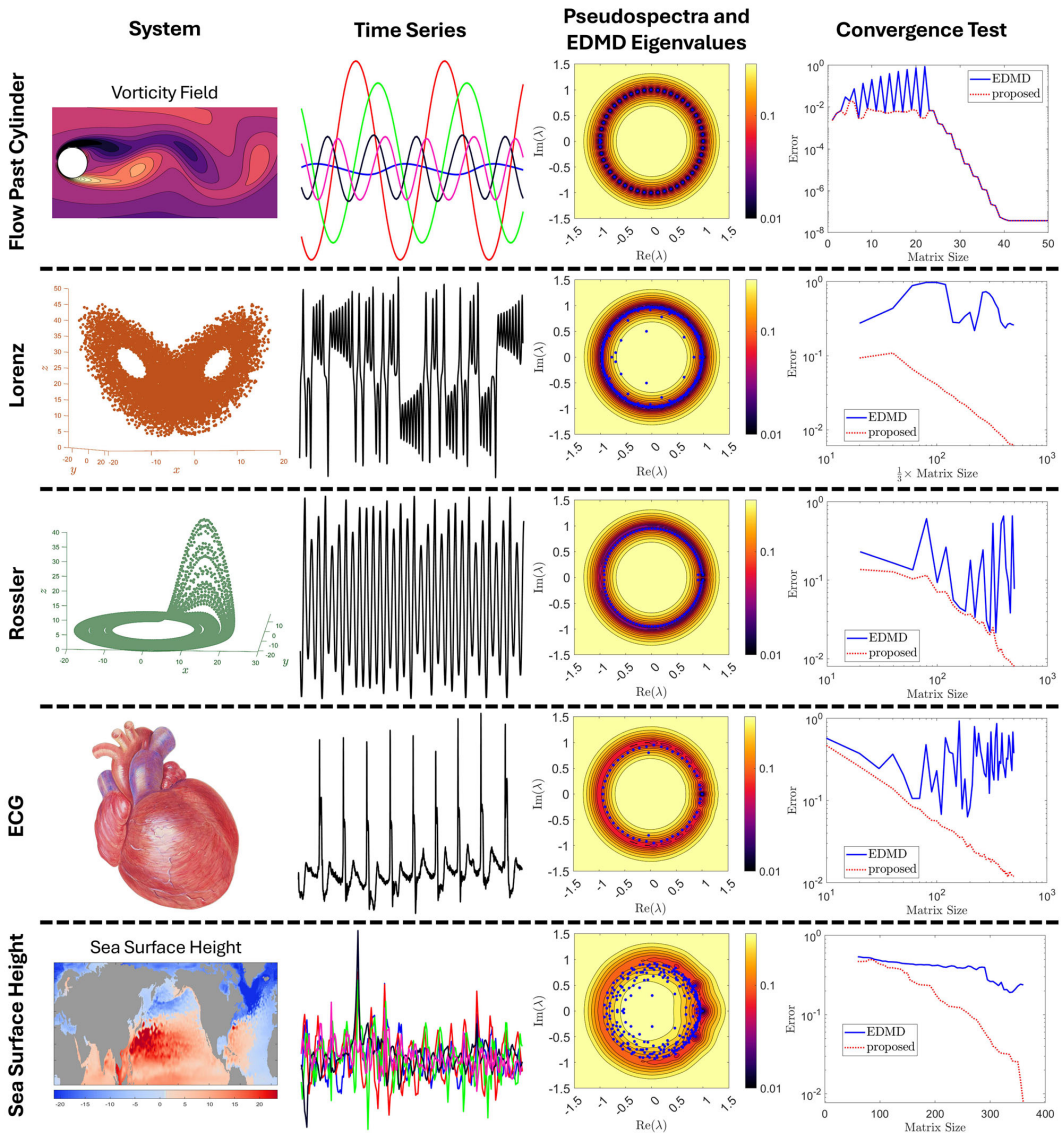
Spectral analysis of a range of analytic and real-world dynamical systems using our proposed algorithms. Each row corresponds to a different system: (i) periodic flow past a cylinder at *Re* = 100, (ii) Lorenz system, (iii) Rössler system, (iv) electrocardiogram (ECG) data (Image: *Heart* by H. G. Wetselaar, Leiden University Libraries / Europeana, Public Domain), and (v) monthly mean sea surface height in the Northern Hemisphere (1950-present). Columns show: the system or dataset; a representative time series of observables; the computed level curves of *ε* for *ε*-approximate eigenfunctions (color scale) termed “pseudospectra” overlaid with EDMD eigenvalues (blue dots); and a convergence comparison between EDMD (blue) and our method (red, using Supplementary Algorithm 1). The pseudospectrum shows where approximate, near-eigenvalue behavior occurs, providing a more robust picture of dynamics. In this final column, the error metric is the same as in Fig. 1. Except for the periodic flow, EDMD yields spurious eigenvalues and lacks convergence. In contrast, our approach produces qualitatively accurate and convergent spectral approximations. Notably, the level curves of *ε* reveal distinct spectral structures across systems: continuous spectra for chaotic systems (Lorenz, Rössler), spectral clustering near *λ* = 1 for ECG, and non-normal features and seasonal modes around $\lambda \approx \exp (2\pi i/12)$ in sea surface height data. Further experimental details are provided in the Supplementary Information.

We first describe the algorithm for ${\Omega }_{{{{\mathcal{X}}}}}^{\alpha,m}$ (Supplementary Algorithm 1). Traditional methods for analyzing the dynamics of complex systems (e.g., EDMD) often rely on estimating the Koopman operator by computing eigenvalues of finite-dimensional matrices. However, these approximations can be unreliable and fail to converge, as shown in Figs. 1, 7. Our approach avoids this pitfall by taking a different route. Instead of directly computing eigenvalues of an approximate matrix, we use trajectory data from the system to construct three matrices that capture the essential correlations between observables and their evolution. These matrices approximate inner products involving the Koopman operator ${{{{\mathcal{K}}}}}_{F}$ and its adjoint ${{{{\mathcal{K}}}}}_{F}^{*}$ (which encodes how observables change in reverse time).

We form a correlation matrix based on how different observables behave across the dataset:

11 $${\widehat{L}}_{ij}=\frac{1}{M}{\sum }_{m=1}^{M}{g}_{j}({y}^{(m)})\overline{{g}_{i}({y}^{(m)})},\,\,i,j=1,\ldots,N,$$

which estimates how the time-evolved observables overlap. It approximates $\langle {{{\mathcal{K}}}}{g}_{j},{{{\mathcal{K}}}}{g}_{i}\rangle$ in an analogous fashion to how Eqs. (8) and (9) approximate 〈*g*_*j*_, *g*_*i*_〉 and $\langle {{{\mathcal{K}}}}{g}_{j},{g}_{i}\rangle$, respectively. Using these matrices, we define a function

12 $${h}_{N}(z,F)=\sqrt{{\sigma }_{\inf }(\hat{L}-\overline{z}\hat{A}-z{\hat{A}}{*}+| z{| }^{2}\hat{G})}.$$

Here, ${\sigma }_{\inf }$ denotes the smallest singular value of a matrix. This method has a key advantage: If *F* has a known modulus of continuity, we adaptively select *N* based on the number of data points *M* (amounting to controlling errors in the approximations of the correlations) to ensure that

13 $${\lim}_{N\to \infty }{h}_{N}(z,F)={\sigma }_{\inf }({{{{\mathcal{K}}}}}_{F}-zI),$$

with convergence from above. Here, *I* denotes the identity operator. Moreover, this specific construction guarantees convergence, in contrast to approaches using ${\sigma }_{\inf }(\widehat{A}-z\widehat{G})$, which lack this property. It establishes the rigorous error bounds demonstrated, for example, in Figs. 1 and 7.

We evaluate this function over an initial grid of *z*-points that is adaptively refined with increasing *N* (a good choice is $\{z\in \frac{1}{n}{\mathbb{Z}}+\frac{i}{n}{\mathbb{Z}}:| z| \le n\}$). The computed function *h*_*N*_(*z*, *F*) yields an upper bound *d*_*N*,*z*_ on ${{{\rm{dist}}}}(z,{{{{\rm{Sp}}}}}_{{{{\rm{ap}}}}}({{{{\mathcal{K}}}}}_{F}))$, provided the non-normality of ${{{{\mathcal{K}}}}}_{F}$ is suitably controlled (e.g., if the system is measure-preserving, see also Eq. (16) of the Supplementary Information for the general resolvent-bounded case). We then find local minimizers of *h*_*N*_(*z*, *F*) in a radius *d*_*N*,*z*_ neighborhood of each grid point. These minimizers provide approximations to the spectrum near each point. The union of these local approximations is guaranteed to converge to the true spectrum as *N* → *∞*. By computing the singular vectors corresponding to the smallest singular values, we simultaneously obtain approximate eigenfunctions *ϕ*_*ε*_, which satisfy the condition $\parallel {{{{\mathcal{K}}}}}_{F}{\phi }_{\varepsilon }-\lambda {\phi }_{\varepsilon }\parallel \le \varepsilon$ (with $\varepsilon={\sigma }_{\inf }({{{{\mathcal{K}}}}}_{F}-\lambda I)$ for a spectral parameter *λ*) and hence the coherency bound in Eq. (5). In addition to providing error bounds, the algorithm is local, trivially parallelizable, and stable.

For the class ${\Omega }_{{{{\mathcal{X}}}}}^{\alpha }$, we cannot directly convert *h*_*N*_(*z*, *F*) into the distance bound *d*_*N*,*z*_ as described above. This limitation reflects the challenge **(C2)** and requires an additional limiting process to achieve the conversion (Supplementary Algorithm 3). Similarly, for the class ${\Omega }_{{{{\mathcal{X}}}}}^{m}$, we are unable to adaptively choose *N* based on the number of data points *M*, due to the challenge **(C1)**. As a result, we must evaluate *h*_*N*_(*z*, *F*) using another limiting procedure (Supplementary Algorithm 4). In the most general case, ${\Omega }_{{{{\mathcal{X}}}}}$, both challenges arise simultaneously, necessitating three successive limits (Supplementary Algorithm 5). This phenomenon of several successive limits occurs in all algorithms for Koopman operators that provably converge. In particular, the above argument using the matrices *L* is a generalization of the ResDMD algorithm^37^.

To separate eigenvalues from continuous spectra, we use a mathematical result known as the RAGE theorem. It states for unitary ${{{\mathcal{K}}}}$ that we can isolate the contribution of eigenfunctions by averaging time-evolved observables after projecting onto an increasing sequence of finite-dimensional subspaces. Specifically, if $\{{{{{\mathcal{P}}}}}_{n}\}$ are finite-rank orthogonal projections converging to the identity, then for any observable *g*, the projection ${{{{\mathcal{P}}}}}_{{{{\rm{pp}}}}}$ onto the closure of the space spanned by eigenfunctions of ${{{\mathcal{K}}}}$ satisfies:

14 $$\parallel {{{{\mathcal{P}}}}}_{{{{\rm{pp}}}}}g{\parallel }^{2}={{{\rm{lim}}}}_{n\to \infty }{{{\rm{lim}}}}_{L\to \infty }\frac{1}{2L+1}{\sum }_{\ell=-L}^{L}{\left\Vert {{{{\mathcal{P}}}}}_{n}{{{{\mathcal{K}}}}}^{\ell }g\right\Vert }^{2}.$$

This formula separates observables into parts that are quasiperiodic over time and parts corresponding to continuous spectra (decay of correlations). This provides a principled way to identify and extract the parts of an observable associated with eigenfunctions. The procedure is implemented in Supplementary Algorithms 6 and 7.

Equation (14) involves two consecutive limits: one over the increasing rank of the projections or number of observables (*n* → *∞*) and another over the time window length (*L* → *∞*). This method enables accurate spectral computations using only finite data, without requiring an explicit approximation of the Koopman operator (which must be formed for EDMD). We also point out that this technique is substantially different from partitions of the full spectral measure (distribution of the system’s behavior across different frequencies) over intervals, which have previously been computed using the ergodic theorem, e.g.,^36^. Moreover, the RAGE theorem does not require the system to be ergodic.

### Duffing oscillator

The Duffing oscillator is the system of equations:

15 $$\frac{dx}{dt}=y,\,\,\frac{dy}{dt}=-\gamma y+x(1-{x}^{2}).$$

The state ${{{\bf{x}}}}=(x,y)\in {{\mathbb{R}}}^{2}$ evolves in a two-dimensional state space. To analyze this system in discrete time, we use a time step of *Δt* = 0.3. We consider two cases based on the parameter *γ*. Conservative case (*γ* = 0): This is a Hamiltonian system: it conserves energy and, by Liouville’s theorem, preserves phase-space area. Dissipative case (*γ* = 0.3): In this scenario, the system has energy dissipation with two stable spirals at ( ± 1, 0) and a saddle point at the origin. These two cases highlight distinct dynamical behaviors. In the Hamiltonian case, trajectories exhibit long-term behavior without dissipation, while in the dissipative case, trajectories eventually converge to stable attractors. EDMD struggles to reliably approximate the Koopman operator spectrum in both cases, illustrating its non-convergence under varying dynamical conditions.

We first generate an initial dataset by uniformly sampling 10^4^ initial conditions **x**_0_ = (*x*_0_, *y*_0_) in the square [−2, 2]^2^ and recording trajectories of 5 time steps for each sample. To construct a dictionary of observables, we apply *k*-means clustering to this initial dataset and use the resulting cluster centers ${\{{c}_{j}\}}_{j=1}^{N}$ to define *N* radial basis functions:

16 $${g}_{j}({{{\bf{x}}}})=\exp (-2\parallel {{{\bf{x}}}}-{c}_{j}{\parallel }_{{l}^{2}}/\sigma ),\,\,j=1,\ldots,N.$$

The trajectory length 5 is chosen to reduce the condition number of the resulting basis (Supplementary Fig. 4) and *k*-means clustering is a standard way to ensure that the centers are well distributed, preventing over-concentration and improving the representativeness and conditioning of the dictionary. The scaling parameter *σ* is set to the average *l*^2^-norm of the snapshot data after it is shifted to mean zero, which we have found empirically to work well across a range of examples. Other dictionary choices are certainly possible. The only requirement is that, as we use more basis functions, the projections acting on any fixed observable converge to that observable.

Once the dictionary is constructed, we further resample *M* snapshots of the system. This corresponds to Monte Carlo integration, where the snapshots approximate the correlations required in our analysis. Supplementary Fig. 5 illustrates the convergence of this approach as the number of snapshots increases, as well as the convergence of *h*_*N*_(*z*, *F*) with increasing *N*.

The level curves of *ε* in Fig. 1 were computed using *N* = 500 basis functions and *M* = 50000 snapshots, and Supplementary Algorithm 2. To compute the spectrum, we used the above adaptive procedure (Algorithm 1 in the S.I). We repeated experiments 10 times with different random seeds for the trajectory data to ensure robustness.

### Further examples comparing EDMD and our algorithms

Our algorithms are further applied to a variety of analytic and real-world systems, as shown in Fig. 7, using trajectory data with qualitatively different characteristics. Further details about each system and experiment are provided in the Supplementary Information, and code for all examples is publicly available. The examples span a broad range of dynamics, including periodic flow past a cylinder and canonical chaotic systems such as the Lorenz and Rössler attractors, which are two of the simplest systems that exhibit chaotic motion. To demonstrate applicability to more realistic scenarios, we also include data collected from an electrocardiogram (ECG), and monthly mean sea surface height in the Northern Hemisphere from 1950 to the present. Data sources are listed in the Supplementary Information. In all cases, our algorithms and EDMD use the same data and the same dictionary to ensure a fair comparison.

In each case, we computed both the pseudospectra (sublevel sets of *ε* for approximate eigenfunctions) using Supplementary Algorithm 2 and the EDMD eigenvalues (blue dots). Except for the periodic flow case, EDMD produces spurious eigenvalues, as it did for the Duffing oscillator in Fig. 1. Figure 7 demonstrates this phenomenon over a range of dictionary choices, data collections, and types of dynamical systems.

The pseudospectra exhibit different structures. For the cylinder flow, the multiplicative structure of the eigenvalues (see the discussion of combination modes in the Arctic sea ice example) is clearly visible in the first row. The Lorenz and Rössler systems display continuous spectra concentrated on the unit circle. The ECG data yields a tight spectral cluster near *λ* = 1 and near the frequency of the recorded heartbeat. The pseudospectra of the sea surface height data show strong non-normal features (the pseudospectra differ from spectral distances), capturing transient dynamics and nonstationary trends in data, along with dominant spectral regions corresponding to seasonal variations around $\lambda \approx \exp (2\pi i/12)$.

In all cases, our spectral approximation algorithm (Supplementary Algorithm 1) converges as expected, whereas EDMD does not converge (except for the periodic flow case).

### High Reynolds number fluid flows

As an example of detecting eigenfunctions using an optimal two-limit procedure (Supplementary Algorithm 6), we consider two-dimensional lid-driven cavity flow^94^. This system involves the motion of an incompressible, viscous fluid at a high Reynolds number (*Re*), which characterizes the dominance of inertial forces over viscous forces, leading to complex flow patterns. This setup provides a challenging example for identifying eigenfunctions of the underlying dynamics.

The domain is the cavity *B* = [−1, 1]^2^, with stationary solid boundaries on all sides except the top. The top boundary moves with a regularized velocity profile ${u}_{{{{\rm{top}}}}}={(1-{x}^{2})}^{2}$. This standard boundary condition ensures both continuity and incompressibility, even at the corners of the top boundary. Using the streamfunction *ψ*, the incompressible Navier–Stokes equations for this flow can be reformulated as:

17 $$\frac{\partial }{\partial t}{\nabla }^{2}\psi+\frac{\partial \psi }{\partial y}\frac{\partial }{\partial x}{\nabla }^{2}\psi -\frac{\partial \psi }{\partial x}\frac{\partial }{\partial y}{\nabla }^{2}\psi=\frac{1}{Re}{\nabla }^{4}\psi,$$

18 $$\psi {| }_{\partial B}=0,\,\frac{\partial \psi }{\partial n}(y\,=\,-1)=\frac{\partial \psi }{\partial n}(x\,=\,\pm 1)=0,\,\frac{\partial \psi }{\partial n}(y\,=\,1)={u}_{{{{\rm{top}}}}}.$$

These equations have a unique solution, and the flow dynamics converge to a universal attractor as time progresses^95^. As discussed in the Results, the spectral structure of the Koopman operator tells us about the geometry of this attractor. To compute *ψ*, we use a Chebyshev spectral collocation method with an adaptive grid resolution^96^, which depends on *Re*, ensuring accurate computation for a range of flow conditions. For our analysis, we utilize *M* = 20000 snapshots of the flow sampled at time intervals of 0.1s to capture its dynamics, after an initial burn-in time to ensure data is collected from the attractor.

We apply Eq. (14) to analyze the mean-subtracted total kinetic energy as our observable *g*. To construct the projections ${{{{\mathcal{P}}}}}_{n}$, we use time-delay embedding, with *n* time delays. This approach captures the temporal structure of the system by embedding the observable in a higher-dimensional space. Specifically, the terms $\parallel {{{{\mathcal{P}}}}}_{n}{{{{\mathcal{K}}}}}^{\ell }g{\parallel }^{2}$ are calculated as follows: First, the Koopman operator ${{{{\mathcal{K}}}}}^{\ell }$ is applied to the trajectory data of *g*, which acts by shifting the time series by *ℓ* steps. Then, the Moore–Penrose inverse is used to apply the projection ${{{{\mathcal{P}}}}}_{n}$, ensuring consistency with the chosen time-delay embedding. Finally, the squared norm is computed by averaging as in Eq. (9), which allows us to extract long-term statistical averages from the trajectory data.

**Fig. 8:**
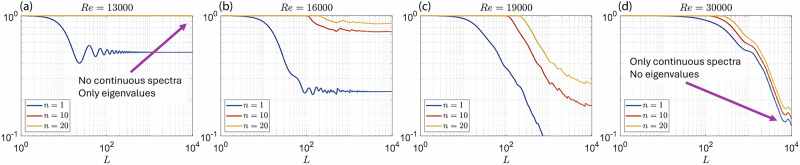
Application of Eq. (14) to the kinetic energy of a lid-driven cavity flow. The plots show $\frac{1}{2L+1}{\sum }_{\ell=-L}^{L}\parallel {{{{\mathcal{P}}}}}_{n}{{{{\mathcal{K}}}}}^{\ell }g{\parallel }^{2}$ (for normalized *g*), which in the double limit ${\lim}_{n\to \infty }{\lim}_{L\to \infty }$ converge to the fraction of *g* made up of eigenfunctions (Supplementary Algorithm 6). This double limit procedure is used to prove upper bounds. As we move from left to right, the Reynolds number of the flow increases, and the spectral type becomes more dominated by continuous spectra. The structure of the spectrum reveals a sequence of bifurcations described in the main text.

Figure 8 shows the results for various choices of *Re*. We observe the double limit ${\lim}_{n\to \infty }\mathop{\lim}_{L\to \infty }$ at play and the structure of the spectrum reveals a sequence of bifurcations as the Reynolds number increases. For *Re*≤10,000, the flow converges to a steady laminar solution, corresponding to a fixed point in the state space. Just above *Re* = 10,000, this steady solution becomes unstable, and the system transitions to a time-periodic flow, which remains stable up to *Re* = 15,000. The fundamental frequency of the periodic flow decreases with increasing Reynolds number. At *Re*≥15,000, a second bifurcation occurs, and the flow becomes quasiperiodic. The basic frequencies of the quasiperiodic motion also decrease with Reynolds number, until around *Re* = 18,000, where a third bifurcation is observed: the portion of kinetic energy associated with the continuous spectrum rises sharply to a few percent. This continuous component continues to grow, and by *Re*≥22,000, the state space dynamics exhibit no quasiperiodic structure: the Koopman spectrum consists solely of continuous components, indicating fully chaotic dynamics. Our results are consistent with the findings of^51,94^, where the authors computed spectral measures for this system.

The bottom of Fig. 1 illustrates the extraction of the eigenvalues for *Re* = 19000. The top part of panel (e) shows the smoothed spectral measure (distribution of the kinetic energy across different frequencies) with a second-order smoothing kernel (taken from^51^) and smoothing parameter *ϵ* = 10^−3^. The bottom part of panel (e) displays the extracted eigenvalues, computed using Eq. (14) (Supplementary Algorithm 6). These are plotted against the Strouhal number, a dimensionless quantity that measures how often vortices are shed from a body relative to the speed of the flow and the size of the body.

### Arctic sea ice

Satellites have measured sea ice for decades using passive microwave sensors, which are then used to compute sea ice concentration using retrieval algorithms. We used data from the European Organisation for the Exploitation of Meteorological Satellites’ (EUMETSAT) Ocean and Sea Ice Satellite Application Facilities (OSI-SAF) data record, comprising retrieval algorithms OSI-450 (1979–2015) and OSI-430-b (2016 onwards). These algorithms have been shown to be more accurate than other retrieval algorithms. Some portions of the data surrounding the North Pole are missing, and we use bilinear interpolation to fill these gaps. There are also missing data for three months in the 1980s due to satellite malfunctions, and we use linear interpolation to fill these gaps.

To construct our approximation of the Koopman operator, we consider observables ${{{\bf{x}}}}\in {{\mathbb{R}}}^{97877}$ representing sea ice concentration at each sea grid point. We then apply time-delay embedding: with *τ* − 1 delays, the augmented system state at time *n* is given by (we use ( ⋅ ) to indicate time dependence instead of a subscript since **x** is a vector):

19 $$\widehat{{{{\bf{x}}}}}(n)=\left(\begin{array}{r}{{{\bf{x}}}}(n)\\ {{{\bf{x}}}}(n-1)\\ \vdots \\ {{{\bf{x}}}}(n-\tau+1)\end{array}\right)\in {{\mathbb{R}}}^{97877\cdot \tau }.$$

That is, we include the sea ice concentration at the current and *τ* − 1 previous time steps. We first choose *τ* = 6 to help capture semiannual patterns, though other choices also yield good performance. As our dictionary, we use Gaussian radial basis functions of the form $\exp (-\frac{100}{81}\parallel \widehat{{{{\bf{x}}}}}-{\widehat{{{{\bf{x}}}}}}^{(m)}{\parallel }_{{l}^{2}}^{2}/{\sigma }^{2}),$ where the centers ${\widehat{{{{\bf{x}}}}}}^{(m)}$ correspond to the *M* snapshot data of the augmented system state (in contrast to the Duffing oscillator where we used *k*-means to choose centers). As in the Duffing oscillator example, the scaling parameter *σ* is set to the average *l*^2^-norm of the snapshot data after centering it to have zero mean. For the three-year forecast in the left panel of Fig. 4, we use a Lorentzian radial-basis-function dictionary; for the binary accuracy problem, we use a poly-exponential dictionary with up to 60 adaptively chosen time delays.

A key strength of our algorithm is its rigorous error bounds (e.g., Figs. 1, 7), which enable direct evaluation of dictionary performance by tracking the error without needing held-out data or forecasts. This allows us to verify that our chosen dictionary yields small errors when approximating the Koopman spectrum. Future work will explore integrating neural network embeddings^30^ with our error-bound framework.

To compute the periodic benchmark in Fig. 4, let *I*_*k*_ denote the time indices for month *k* ( = 1, 2, …, 12) in the training data when we make a forecast. For forecasting month *k*, **x**_per_ is the average of **x** over the times in *I*_*k*_. The difference **x** − **x**_per_ then represents the sea ice anomaly. Error metrics are computed over active grid points defined separately for each calendar month. This region expands in winter and contracts in summer according to the maximum observed sea ice extent for that month, following^63^. We define *σ*^2^ as the average value of $\parallel {{{\bf{x}}}}-{{{{\bf{x}}}}}_{{{{\rm{per}}}}}{\parallel }_{{l}^{2}}^{2}$, evaluated over the active grid points, at a lead time of one month and averaged over the period 2005–2015. A one-month lead time is used to avoid the trivial zero-error baseline at initialization. Letting **x** be the true sea ice concentration and **x**_rec_ the forecast, we define the relative error shown in Fig. 4 (a) as ${{{\rm{Error}}}}=\parallel {{{\bf{x}}}}-{{{{\bf{x}}}}}_{{{{\rm{rec}}}}}{\parallel }_{{l}^{2}}^{2}/{\sigma }^{2},$ where the *l*^2^ norm is computed over the active grid points (and averaged over the initializations in the years 2005–2015). This normalization removes the trivially predictable seasonal cycle, enabling a more meaningful assessment of forecast skill. We also include a monthly persistence model as a baseline for comparison, in which the sea ice concentration is forecast by repeating its value from the previous year for the same calendar month.

To compute forecasts, we get rid of spurious modes in the decomposition in Eq. (6) (where **g** is the vector of sea ice concentrations **x**). The evolution is predicted forward in time for **x** using approximate eigenfunctions ${\phi }_{\varepsilon }^{(j)}$ for errors *ε* below a threshold *ε*_0_:

20 $${{{\bf{x}}}}(n)=\mathop{\sum }_{\varepsilon \le {\varepsilon }_{0}}{\lambda }_{j}^{n}{\phi }_{\varepsilon }^{(j)}(0){{{{\bf{g}}}}}_{j}.$$

Here, the parameter *j* ranges from 1 to *M* (corresponding to the space generated by the radial basis functions). The errors associated with the approximate eigenfunctions *ϕ*_*ε*_ are ordered by *ε*, and we identify the “elbow” in the error curve to determine a principled truncation point (Supplementary Fig. 12). The Koopman modes **g**_*j*_ correspond to the vector **x** of observables. After this model has been built, forecasts are produced by increasing *n*. The DMD forecast in Fig. 4b is computed in the same manner, but now does not get rid of spurious modes and uses the augmented state space $\widehat{{{{\bf{x}}}}}$ as the dictionary of observables. IceNet and SEAS5 data in Fig. 4 are taken directly from^63^.

### Lower bounds: the method of adversarial systems

We establish each lower bound (impossibility result) using simple examples of state spaces ${{{\mathcal{X}}}}$ (e.g., disk, interval, or torus) where *ω* represents the standard Lebesgue measure. The techniques are general and can be extended to other state spaces ${{{\mathcal{X}}}}$ and function spaces. Snapshot data is subject to noise and finite precision. To establish robust results, we use a measurement device ${{{{\mathcal{T}}}}}_{F}$ that allows arbitrarily accurate sampling:

21 $${{{{\mathcal{T}}}}}_{F}=\left\{{\widehat{y}}_{j,n}\in {{{\mathcal{X}}}}:{d}_{{{{\mathcal{X}}}}}(F({\widehat{x}}_{j}),{\widehat{y}}_{j,n})\le {2}^{-n},n\in {\mathbb{N}}\right\},$$

where ${\{{\widehat{x}}_{j}\}}_{j=1}^{\infty }$ is a dense subset of the metric space $({{{\mathcal{X}}}},{d}_{{{{\mathcal{X}}}}})$, corresponding physically to “measurement points”. This formulation assumes that measurements can approximate the mapping *F* with arbitrarily high precision. This is a strong assumption, and hence it allows us to derive correspondingly strong impossibility results, highlighting fundamental limits even under idealized conditions. Limitations under idealized conditions imply that they hold under more realistic ones. (It can also be significantly relaxed for our upper bounds.)

To establish the lower bounds, we construct families of adversarial dynamical systems. These systems are carefully designed to embed sudden changes in the spectral properties of the Koopman operator ${{{{\mathcal{K}}}}}_{F}$ directly into the dynamics, while remaining consistent with the observed trajectory data.

**Fig. 9:**
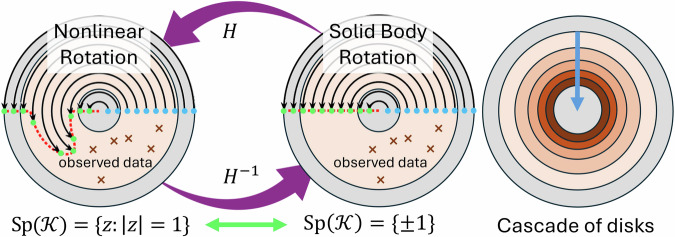
Proof idea of the impossibility result for ${\Omega }_{{\mathbb{D}}}$. At each stage, we modify the system consistently with the observed data ("x''), ensuring it is related to a rotation, thereby drastically altering the spectrum (see green arrow). This alteration is executed such that the cascade of dynamical systems converges to an underlying limit, providing the adversarial family of systems.

Figure 9 illustrates this idea for the class ${\Omega }_{{\mathbb{D}}}$. The construction uses a homeomorphism (a continuous, reversible transformation that stretches or bends a space without tearing, gluing, or creating holes) to deform the map while preserving the sampled data. This sudden change lemma (Lemma 2.7 in the Supplementary Information) alters the spectral behavior of the Koopman operator in a controlled way. By applying this construction recursively on a cascade of nested disks, we effectively force any proposed algorithm to fail to converge. Each adversarial construction is tailored to a specific sudden change lemma, with full details provided in the Supplementary Information. This strategy bridges computational techniques with classical ergodic theory, offering a framework for analyzing dynamical systems. Moreover, the method is flexible and can be adapted to tackle a broader range of problems beyond those considered in this study.

To prove a learning problem cannot be solved in one limit (i.e., it has SCI > 1), we assume, for contradiction, that a convergent sequence of algorithms exists. We then construct an adversarial family that causes the algorithm to fail, ensuring convergence occurs with probability no greater than 50%. This involves tricking the algorithm into oscillating between two different outputs as more trajectory data is collected (see the green arrow in Fig. 9). To prove that a problem cannot be solved in two limits (i.e., SCI > 2), we embed complex combinatorial problems into the system’s dynamics. These problems, involving sets of numbers with an inherent complexity in their description, are embedded into the dynamics, lifting the lower bound from combinatorics to dynamics.

## Supplementary information

Supplementary Information

Transparent Peer Review file

## Data Availability

The observational sea ice concentration data are provided by OSI-SAF. The results of IceNet and SEAS5 in Fig. 4 are reported in ref. ^63^. All other datasets are produced by the code listed below.

## Code availability

Code for the examples of this paper can be found at https://github.com/MColbrook/Adversarial-Dynamical-Systems.
