# Supplementary material for: Adversarial dynamical systems characterize when data-driven learning succeeds or fails
Source: Nat Commun. 2026 Jul 14;17:5397. doi: 10.1038/s41467-026-74220-8 (PMC13369498; doi:10.1038/s41467-026-74220-8)
Supplement: Supplementary file 1 — Supplementary Information [file 41467_2026_74220_MOESM1_ESM.pdf]

Supplementary Information: Adversarial dynamical systems characterize when data-driven learning succeeds or fails

Matthew J. Colbrook[1], Igor Mezić[2], Alexei Stepanenko[1]

[1] DAMTP, University of Cambridge, Cambridge, CB3 0WA, UK.

[2] University of California, Santa Barbara, CA 93106, USA.

Contents

1 Background: A general framework **1**
1.1 Koopman operators – linearising nonlinear systems . . . 2
1.1.1 Koopman spectrum . . . 2
1.2 The Solvability Complexity Index – classifying the difficulty of problems . . . 3
1.2.1 Computational problems and general algorithms . . . 3
1.2.2 Towers of algorithms . . . 4
1.2.3 Inexact input . . . 5
1.2.4 Refinements that capture error control . . . 5
1.2.5 Randomized algorithms . . . 7
1.3 The setup of this paper – a perfect measurement device (which implies stronger results) . . . 9

2 Theorems that tell us what is possible and what is not possible **9**
2.1 A general computational result – upper bounds or "possibility results" . . . 9
2.2 Measure-preserving maps on the unit disk . . . 12
2.3 Maps on the unit interval . . . 16
2.4 Computing the spectral type is impossible in one limit . . . 20
2.5 Spectra of discrete-space systems . . . 22

3 Pseudocode for algorithms **24**
3.1 Algorithms for the approximate point spectrum and pseudospectrum . . . 24
3.2 Algorithms for spectral type and pure point spectrum . . . 25

4 Further experimental diagnostics **27**
4.1 Further analysis for Duffing oscillator . . . 27
4.2 Further analysis for cavity flow . . . 28
4.3 Further analysis for Arctic sea ice . . . 29
4.4 Other dynamical systems . . . 31
4.4.1 Flow past a cylinder . . . 31
4.4.2 Lorenz system . . . 33
4.4.3 Rössler system . . . 33
4.4.4 Electrocardiogram (ECG) . . . 34
4.4.5 Northern Hemisphere sea surface height . . . 34

1 Background: A general framework

This section provides further details for constructing our adversarial dynamical systems and classifying problems. We introduce the Solvability Complexity Index (SCI), which, as discussed in the main text, provides a general framework across data-driven dynamical system problems, and consider Koopman operators and their spectral properties. We end this section by discussing the setup for the theorems.

1.1 Koopman operators – linearising nonlinear systems

Throughout, we consider discrete-time dynamical systems:

$$x_{n+1} = F(x_n), \qquad n = 0, 1, 2, \ldots. \tag{1}$$

Here, $x \in \mathcal{X}$ denotes the state of the system, and the metric space $(\mathcal{X}, d_{\mathcal{X}})$ denotes the state space. Often, $\mathcal{X} \subset \mathbb{R}^d$, though this is not required in what follows. The function $F : \mathcal{X} \to \mathcal{X}$ governs the evolution of the dynamical system and is generally nonlinear. (Here, we mean that F is linear if $\mathcal{X}$ is a vector space and F is a linear map. Otherwise, we call F nonlinear.) We lift the system (1) into a (typically infinite-dimensional) vector space of observable functions using a Koopman operator to deal with the nonlinearity. A Koopman operator [41, 42] is defined on a Banach space $\mathcal{J}$ of functions $g : \mathcal{X} \to \mathbb{C}$, where the functions $g \in \mathcal{J}$ are referred to as *observables* and measure the state of the system. Koopman operators allow us to study the evolution of observables in $\mathcal{J}$ through a linear framework. The Koopman operator is defined via the composition formula:

$$[\mathcal{K}g](x) = [g \circ F](x) = g(F(x)), \qquad g \in \mathcal{D}(\mathcal{K}), \tag{2}$$

where $\mathcal{D}(\mathcal{K}) \subset \mathcal{J}$ is a suitable domain. This definition means that $[\mathcal{K}g](x_n) = g(F(x_n)) = g(x_{n+1})$ represents the measurement of the state one time-step ahead of $g(x_n)$, and hence that $\mathcal{K}$ effectively captures the dynamic progression of the system.

The critical property of the Koopman operator $\mathcal{K}$ is its *linearity*. This linearity holds irrespective of whether the map F in Equation (1) is linear or nonlinear. Consequently, the spectral properties of $\mathcal{K}$ become a powerful tool in analyzing the dynamical system's behavior. The Koopman operator is not defined uniquely by the dynamical system in (1), but fundamentally depends on the space $\mathcal{J}$. Throughout this paper, we focus on the choice

$$\mathcal{J} = \mathcal{D}(\mathcal{K}) = L^2(\mathcal{X}, \omega) \quad \text{with inner product} \quad \langle g_1, g_2 \rangle = \int_{\mathcal{X}} g_1(x)\overline{g_2(x)}\, \mathrm{d}\omega(x) \quad \text{and norm} \quad \|g\| = \sqrt{\langle g, g \rangle},$$

for some positive measure ω. We do not assume that this measure is invariant. For Hamiltonian systems, a common choice of ω is the standard Lebesgue measure, for which the Koopman operator is unitary on $L^2(\mathcal{X}, \omega)$. For other systems, we can select ω according to the region where we wish to study the dynamics, for example, by using a Gaussian density. In many applications, ω corresponds to an unknown ergodic measure on an attractor.

In going from a pointwise definition in (2) to the space $L^2(\mathcal{X}, \omega)$, care is needed since $L^2(\mathcal{X}, \omega)$ consists of equivalence classes of functions. We assume that the map F is nonsingular with respect to ω, meaning that

$$\omega(E) = 0 \quad \text{implies that} \quad \omega(\{x : F(x) \in E\}) = 0.$$

This condition ensures that the Koopman operator is well-defined since $g_1(x) = g_2(x)$ for ω-almost every x implies that $g_1(F(x)) = g_2(F(x))$ for ω-almost every x. The pushforward measure is defined as $F\#\omega(E) = \omega(F^{-1}(E))$, and the fact that F is nonsingular with respect to ω is equivalent to saying that $F\#\omega$ is absolutely continuous with respect to ω. We assume that $\mathcal{K}$ is a bounded linear operator on the Hilbert space $L^2(\mathcal{X}, \omega)$. This assumption is equivalent to saying that the Radon–Nikodym derivative $dF\#\omega/d\omega$ lies in $L^\infty(\mathcal{X}, \omega)$. The above Hilbert space setting is standard in the Koopman literature, though our results can be extended to other function spaces such as those studied in [50]. Once $(\mathcal{X}, \omega)$ are specified, we let $\mathcal{K}_F$ denote the corresponding Koopman operator on the corresponding Hilbert space $L^2(\mathcal{X}, \omega)$.

Since $\mathcal{K}_F$ acts on an *infinite-dimensional* function space, we have exchanged the nonlinearity in (1) for an infinite-dimensional linear system. This means that the spectral properties of $\mathcal{K}_F$ can be significantly more complex than those of a finite matrix, making them more challenging to compute. A message of this paper is that, in most cases, unless strong assumptions are made regarding the system, the spectral properties of $\mathcal{K}_F$ are impossible to compute in a single limit, even if we had a perfect measurement device to sample trajectories of the dynamical system.

1.1.1 Koopman spectrum

If $g \in L^2(\mathcal{X}, \omega)$ is an *eigenfunction* of $\mathcal{K}_F$ with *eigenvalue* λ, then g exhibits perfect coherence[1] since

$$g(x_n) = [\mathcal{K}_F^n g](x_0) = \lambda^n g(x_0) \quad \forall n \in \mathbb{N}. \tag{3}$$

The oscillation and decay/growth of the observable g are dictated by the complex argument and absolute value of the eigenvalue λ, respectively. In infinite dimensions, the appropriate generalization of the set of eigenvalues of $\mathcal{K}_F$ is the *spectrum*:

$$\mathrm{Sp}(\mathcal{K}_F) = \left\{ z \in \mathbb{C} : (\mathcal{K}_F - zI)^{-1} \text{ does not exist as a bounded operator} \right\} \subset \mathbb{C}.$$

Here, I denotes the identity operator. In contrast to finite matrices, the spectrum $\mathrm{Sp}(\mathcal{K}_F)$ may contain points that are not eigenvalues. This phenomenon occurs because there are more ways for $(\mathcal{K}_F - zI)^{-1}$ to not be a linear bounded operator

[1] Coherence here is meant in the sense of an observation for which all the points in state space exhibit the same, (complex) exponential, time-dependence.

in infinite dimensions than in finite dimensions. For example, the standard Lorenz system on the Lorenz attractor has a Koopman operator with no eigenvalues except $\lambda = 1$ [45]. The *approximate point spectrum* is

$$\mathrm{Sp}_{\mathrm{ap}}(\mathcal{K}_F) = \left\{\lambda \in \mathbb{C} : \exists \{g_n\}_{n\in\mathbb{N}} \subset L^2(\mathcal{X}, \omega) \text{ such that } \|g_n\| = 1, \lim_{n\to\infty} \|(\mathcal{K}_F - \lambda I)g_n\| = 0\right\} \subset \mathrm{Sp}(\mathcal{K}_F) \subset \mathbb{C}$$

and for $\epsilon > 0$, the *approximate point pseudospectrum* is

$$\mathrm{Sp}_{\mathrm{ap},\epsilon}(\mathcal{K}_F) = \left\{\lambda \in \mathbb{C} : \exists \{g_n\}_{n\in\mathbb{N}} \subset L^2(\mathcal{X}, \omega) \text{ such that } \|g_n\| = 1, \lim_{n\to\infty} \|(\mathcal{K}_F - \lambda I)g_n\| \leq \epsilon\right\} \subset \mathrm{Sp}_\epsilon(\mathcal{K}_F) \subset \mathbb{C}.$$

An observable g with $\|g\| = 1$ and $\|(\mathcal{K}_F - \lambda I)g\| \leq \epsilon$ for $\lambda \in \mathbb{C}$ is known as an ϵ-approximate eigenfunction. Such observables satisfy

$$\|\mathcal{K}_F^n g - \lambda^n g\| = \mathcal{O}(n\epsilon) \text{ as } \epsilon \downarrow 0 \quad \forall n \in \mathbb{N}.$$

In other words, λ describes an *approximate* coherent oscillation and decay/growth of the observable g with time. The approximate eigenfunctions and $\mathrm{Sp}_{\mathrm{ap}}(\mathcal{K}_F)$ encode information about the underlying dynamical system [51]. For example, the level sets of certain eigenfunctions determine ergodic partitions [48, 13], invariant manifolds [49], isostables [47], and the global stability of equilibria [46].

In this paper, we will focus on the computation of $\mathrm{Sp}_{\mathrm{ap}}(\mathcal{K}_F)$. We anticipate that further foundational results can be proven on the computation of other spectral properties of Koopman operators, such as spectral type. For example, see Theorem 2.12 regarding the detection of non-trivial eigenfunctions.

Two special classes of Koopman operators are defined as follows:

- Measure-preserving systems: The dynamical system preserves ω if and only if $\mathcal{K}_F$ is an isometry, that is $\mathcal{K}_F^*\mathcal{K}_F = I$.
- Measure-preserving invertible systems: The dynamical system preserves ω and is invertible modulo ω-null sets [30, Chapter 7] if and only if $\mathcal{K}_F$ is unitary, that is $\mathcal{K}_F^*\mathcal{K}_F = \mathcal{K}_F\mathcal{K}_F^* = I$.

The Wold–von Neumann decomposition [53, Theorem I.1.1] states that any isometry on a Hilbert space is unitarily equivalent to the direct sum of a unitary operator and a direct sum of unilateral shifts. Hence, if $\mathcal{K}_F$ is an isometry but not unitary, then the spectrum of $\mathcal{K}_F$ is the unit disk and $\mathrm{Sp}_{\mathrm{ap}}(\mathcal{K}_F) = \mathbb{T} = \{z \in \mathbb{C} : |z| = 1\}$. If $\mathcal{K}_F$ is unitary, then the spectrum of $\mathcal{K}_F$ is equal to $\mathrm{Sp}_{\mathrm{ap}}(\mathcal{K}_F)$ and is a subset of $\mathbb{T}$.

1.2 The Solvability Complexity Index – classifying the difficulty of problems

We now outline the fundamentals of the Solvability Complexity Index Hierarchy. This tool allows us to precisely classify the difficulty of computational problems and prove that algorithms are optimal, realizing the boundaries of what is possible. We give the definitions of the hierarchy before specializing to the computational setup of this paper, where we give a precise formulation of a *perfect measuring device*, which we use to prove our lower bounds (impossibility results).

1.2.1 Computational problems and general algorithms

Before classifying the difficulty of computational problems, we must precisely define a computational problem. Precision here is essential since altering the information an algorithm is permitted to use can significantly affect the difficulty of a problem or even whether the problem can be solved at all. The following definition of a computational problem is deliberately general, designed to encompass all types of problems encountered in computational mathematics. For example, as well as spectral problems, the SCI hierarchy has been applied to other areas of mathematics, including PDEs [20, 6], the limits of AI, and Smale's 18th problem [23], and optimization [5].

Definition 1.1 (Computational problem)**.** The basic objects of a computational problem are:

- A *primary set*, Ω, that describes the input class;
- A *metric space* $(\mathcal{M}, d)$;
- A *problem function* $\Xi : \Omega \to \mathcal{M}$;
- An *evaluation set*, Λ, of functions on Ω.

The problem function Ξ is the object we want to compute, with the notion of convergence captured by the metric space $(\mathcal{M}, d)$. The evaluation set Λ describes the information that algorithms can read. We require that Λ separates elements of Ω to the degree of separation achieved by Ξ:

$$\text{if } A, B \in \Omega \text{ with } \Xi(A) \neq \Xi(B), \text{ then } \exists f \in \Lambda \text{ with } f(A) \neq f(B). \tag{4}$$

In other words, any $\Xi(A) \in \mathcal{M}$ is uniquely determined by the set of evaluations $\{f(A) : f \in \Lambda\}$ (otherwise it is impossible to recover Ξ from Λ). We refer to the collection $\{\Xi, \Omega, \mathcal{M}, \Lambda\}$ as a *computational problem.*

Example 1.2. In the above setting of dynamical systems, we fix the metric space $(\mathcal{X}, d_{\mathcal{X}})$ and the measure ω. The primary set Ω could be a class of functions $F : \mathcal{X} \to \mathcal{X}$, each of which induces a dynamical system with bounded $\mathcal{K}_F$ on $L^2(\mathcal{X}, \omega)$. The problem function could describe a spectral property of $\mathcal{K}_F$. For example, we could consider the computation of the approximate point spectrum with $\Xi(F) = \mathrm{Sp}_{\mathrm{ap}}(\mathcal{K}_F)$. Since $\mathcal{K}_F$ is bounded, $\Xi(F)$ is a compact, non-empty subset of $\mathbb{C}$ [28, Proposition VII.6.7]. Hence, we let $(\mathcal{M}, d)$ be the Hausdorff metric space, $(\mathcal{M}_{\mathrm{H}}, d_{\mathrm{H}})$, which is the collection of non-empty compact subsets of $\mathbb{C}$ equipped with the Hausdorff metric:

$$d_{\mathrm{H}}(X, Y) = \max\left\{\sup_{x\in X}\inf_{y\in Y}|x-y|, \sup_{y\in Y}\inf_{x\in X}|x-y|\right\}, \quad X, Y \in \mathcal{M}_{\mathrm{H}}.$$

Convergence to the spectrum in this metric means our algorithms converge without spectral pollution (persistent spurious eigenvalues) or spectral invisibility (missing parts of the spectrum). As our evaluation set, we could consider maps $f_x(F) = F(x)$ for $x \in \mathcal{X}$ or a subset of such x. For example, if $\mathcal{X} \subset \mathbb{R}^d$, then f_x is a real vector-valued function. ■

With the definition of a computational problem $\{\Xi, \Omega, \mathcal{M}, \Lambda\}$ established, we now define what we mean by an algorithm. An algorithm is a function $\Gamma : \Omega \to \mathcal{M}$ that, unlike the problem function Ξ, utilizes the evaluation set Λ in some manner. The specifics of how Λ is used (or even which sets of Λ are permitted) depend on the computational model. We adopt a general definition for proving lower bounds (impossibility results). This approach not only yields stronger results but also significantly simplifies the proofs. Specifically, we aim to establish lower bounds that are valid in *any model of computation*.

Definition 1.3 (General algorithm)**.** A general algorithm for a computational problem $\{\Xi, \Omega, \mathcal{M}, \Lambda\}$ is a map $\Gamma : \Omega \to \mathcal{M}$ with the following property. For any $A \in \Omega$, there exists a non-empty finite subset of evaluations $\Lambda_\Gamma(A) \subset \Lambda$ such that if $B \in \Omega$ with $f(A) = f(B)$ for every $f \in \Lambda_\Gamma(A)$, then $\Lambda_\Gamma(A) = \Lambda_\Gamma(B)$ and $\Gamma(A) = \Gamma(B)$.

Definition 1.3 outlines the most fundamental properties of any reasonable *deterministic* computational device. It says that Γ can only use a finite amount of information, though it can adaptively choose this information as it processes the input. Moreover, the output of Γ depends consistently on the information it has accessed. Specifically, if Γ sees the same information for two different inputs, it must behave identically for those two inputs. A general algorithm has no restrictions on the operations allowed. It is more powerful than a Turing machine [66] or BSS machine[2] [11] and serves two main purposes:

(i) *A focus on what really matters:* Definition 1.3 significantly simplifies the process of proving lower bounds. The non-computability results we present stem from the intrinsic non-computability of the problems themselves, not from the type of operations allowed being too restrictive. Specifically, the limitation lies in the algorithmic input Λ being inadequate for solving the problem.

(ii) *Classifications with the strongest possible lower and upper bounds:* The generality of Definition 1.3 implies that a lower bound established for general algorithms also applies to any computational model. Furthermore, the algorithms we provide can be executed using only arithmetic operations, both in the Turing and BSS models. Therefore, we derive the strongest possible lower and upper bounds simultaneously.

1.2.2 Towers of algorithms

Having established precise definitions for a computational problem and a general algorithm, we now introduce the concept of a tower of algorithms. This captures the observation in the main text that algorithms for data-driven Koopmanism depend on several parameters that must be taken to successive limits to ensure convergence.

Definition 1.4 (Tower of algorithms)**.** Let $k \in \mathbb{N}$. A tower of algorithms of height k for a computational problem $\{\Xi, \Omega, \mathcal{M}, \Lambda\}$ is a collection of functions

$$\Gamma_{n_k}, \Gamma_{n_k,n_{k-1}}, \ldots, \Gamma_{n_k,\ldots,n_1} : \Omega \to \mathcal{M}, \quad n_k, \ldots, n_1 \in \mathbb{N},$$

where $\{\Gamma_{n_k,\ldots,n_1}\}$ are general algorithms (Definition 1.3) and for every $A \in \Omega$, the following convergence holds in $(\mathcal{M}, d)$:

$$\Xi(A) = \lim_{n_k\to\infty}\Gamma_{n_k}(A), \quad \Gamma_{n_k}(A) = \lim_{n_{k-1}\to\infty}\Gamma_{n_k,n_{k-1}}(A), \quad \ldots \quad \Gamma_{n_k,\ldots,n_2}(A) = \lim_{n_1\to\infty}\Gamma_{n_k,\ldots,n_1}(A).$$

We shall use the term "tower" even if $k = 1$.

When we prove upper bounds (i.e., provide algorithms that solve a problem), we can specify the type of tower by imposing conditions on the functions $\{\Gamma_{n_k,\ldots,n_1}\}$ at the lowest level. In essence, the type is the toolbox allowed:

- A *general tower*, denoted by $\alpha = G$, refers to Definition 1.4 with no further restrictions.

[2] One should think of a Blum–Shub–Smale (BSS) machine as akin to an algorithm that deals with exact arithmetic. They are a model of computation designed to work over any ring or field, most notably over the real numbers. This distinguishes them from Turing machines, which are based on discrete values. A BSS machine extends the concept of computation to include computation with real numbers and other continuous data.

- An *arithmetic tower*, denoted by $\alpha = A$, refers to Definition 1.4 where each $\Gamma_{n_k,\ldots,n_1}(A)$ can be computed using Λ and finitely many arithmetic operations and comparisons. More precisely, if Λ is countable, each output $\Gamma_{n_k,\ldots,n_1}(A)$ is a finite string of numbers (or encoding) that can be identified with an element in $\mathcal{M}$, and the following function is recursive: $(n_k,\ldots,n_1,\{f(A)\}_{f\in\Lambda}) \mapsto \Gamma_{n_k,\ldots,n_1}(A)$.

We can now define the Solvability Complexity Index (SCI).

Definition 1.5 (Solvability Complexity Index)**.** A computational problem $\{\Xi,\Omega,\mathcal{M},\Lambda\}$ has Solvability Complexity Index $k \in \mathbb{N}$ with respect to type α, written $\mathrm{SCI}(\Xi,\Omega,\mathcal{M},\Lambda)_\alpha = k$, if k is the smallest integer for which there exists a tower of algorithms of type α and height k that solves the problem. If no such tower exists, then $\mathrm{SCI}(\Xi,\Omega,\mathcal{M},\Lambda)_\alpha = \infty$. If there exists an algorithm Γ of type α with $\Xi = \Gamma$, then $\mathrm{SCI}(\Xi,\Omega,\mathcal{M},\Lambda)_\alpha = 0$.

The SCI induces the SCI hierarchy as follows.

Definition 1.6 (SCI hierarchy)**.** Consider a collection $\mathcal{C}$ of computational problems and let $\mathcal{T}_\alpha$ be the collection of all towers of algorithms of type α. We define the following subclasses of $\mathcal{C}$:

$$\begin{aligned}
\Delta_0^\alpha &= \{\{\Xi,\Omega,\mathcal{M},\Lambda\} \in \mathcal{C} : \mathrm{SCI}(\{\Xi,\Omega,\mathcal{M},\Lambda\})_\alpha = 0\},\\
\Delta_1^\alpha &= \{\{\Xi,\Omega,\mathcal{M},\Lambda\} \in \mathcal{C} : \exists\{\Gamma_n\}_{n=1}^\infty \in \mathcal{T}_\alpha \text{ s.t. } \forall A \in \Omega, d(\Gamma_n(A),\Xi(A)) \leq 2^{-n}\},\\
\Delta_{m+1}^\alpha &= \{\{\Xi,\Omega,\mathcal{M},\Lambda\} \in \mathcal{C} : \mathrm{SCI}(\{\Xi,\Omega,\mathcal{M},\Lambda\})_\alpha \leq m\}, \quad \text{for } m \in \mathbb{N}.
\end{aligned}$$

In summary, a Δ_{m+1}^α problem can be computed in m successive limits, and a Δ_1^α problem can be computed in one limit with complete error control. The 2^{-n} in the definition of Δ_1^α is arbitrary; replacing it with any sequence converging to zero that is computable from Λ does not alter the definition.

1.2.3 Inexact input

So far, we have only discussed algorithms with exact input from the evaluation set Λ. However, in practice, we may only have access to input of a certain accuracy. Suppose we are given a computational problem $\{\Xi,\Omega,\mathcal{M},\Lambda\}$ with evaluation set $\Lambda = \{f_j : \Omega \to \mathcal{M}_\Lambda\}_{j\in\mathcal{I}}$, for some index set $\mathcal{I}$ and metric space $(\mathcal{M}_\Lambda, d_\Lambda)$. Obtaining f_j may be a computational task in its own right. For instance, $f_j(A)$ could be the number $\cos(\sin(1))$ or an inner product that is approximated using quadrature. Alternatively, it may be the case that we can only measure $f_j(A)$ to a certain accuracy due to effects such as noise. In the context of Koopman operators, any physical measurement device will have a non-zero measurement error.

We may view the problem of obtaining $f_j(A)$ as a problem in the SCI hierarchy. A Δ_1-classification corresponds to access to $f_{j,n} : \Omega \to \mathcal{M}_\Lambda$ such that

$$d_\Lambda(f_{j,n}(A), f_j(A)) \leq 2^{-n} \quad \forall A \in \Omega. \tag{5}$$

We want algorithms that can handle all possible choices of such inexact input. We can make this precise by replacing the class Ω by the class of suitable evaluation functions $\{f_{j,n_1}(A)\}_{j,n_1\in\mathcal{I}\times\mathbb{N}}$ that satisfy Equation (5). This viewpoint is well-defined since Equation (4) holds. The following definition captures the notion of algorithms being robust to noise in input data.

Definition 1.7 (Computational problems with Δ_1-information)**.** Given a computational problem $\{\Xi,\Omega,\mathcal{M},\Lambda\}$, the corresponding computational problem with Δ_1-information is denoted by $\{\Xi,\Omega,\mathcal{M},\Lambda\}^{\Delta_1} = \{\tilde{\Xi},\tilde{\Omega},\mathcal{M},\tilde{\Lambda}\}$, and defined as follows:

- The primary set $\tilde{\Omega}$ is the class of tuples $\tilde{A} = \{f_{j,n_1}(A) : j \in \mathcal{I}, n_1 \in \mathbb{N}\}$, where $A \in \Omega$, $\{f_j\}_{j\in\mathcal{I}}$ and Equation (5) holds;
- The problem function is $\tilde{\Xi}(\tilde{A}) = \Xi(A)$, which is well-defined by Equation (4);
- The evaluation set is $\tilde{\Lambda} = \{\tilde{f}_{j,n_1}\}_{j,n_1\in\mathcal{I}\times\mathbb{N}}$, where $\tilde{f}_{j,n_1}(\tilde{A}) = f_{j,n_1}(A)$.

The SCI hierarchy given Δ_1-information is then defined in an obvious manner.

1.2.4 Refinements that capture error control

When performing numerical computations, particularly in many spectral applications, determining the accuracy of the results is essential. The importance of error bounds extends beyond science and engineering and holds in pure mathematics, especially when using spectral problems in computer-assisted proofs. For instance, even when a discretization method for computing spectra through eigenvalues converges, typically, only a subset of the numerically computed eigenvalues is reliable. Note that such a problem is not computable in the classical Turing sense but instead verifiable. Most infinite-dimensional spectral problems do not lie in Δ_1 [18], but many lie in the following refinements that capture error control [26, 25]. Classifying when error bounds can or cannot be obtained is a fundamental challenge in dealing with infinite-dimensional spectral problems.

Sufficient structure in $(\mathcal{M}, d)$ enables two types of verification or error control: convergence from above and below. In this paper, there are two metric spaces that we use for computational problems $\{\Xi,\Omega,\mathcal{M},\Lambda\}$:

- If $\mathcal{M} = \{0,1\}$ with the discrete topology, we call the problem a decision problem and denote this space by $\mathcal{M}_{\text{dec}}$. For an input $A \in \Omega$, we interpret the output $\Xi(A) = 1$ as "Yes" and the output $\Xi(A) = 0$ as "No".
- The Hausdorff metric space, $(\mathcal{M}_{\text{H}}, d_{\text{H}})$, is suitable for computing spectra of bounded operators. It is the collection of non-empty compact subsets of $\mathbb{C}$ equipped with the Hausdorff metric:

$$d_{\text{H}}(X,Y) = \max\left\{\sup_{x\in X}\inf_{y\in Y}|x-y|, \sup_{y\in Y}\inf_{x\in X}|x-y|\right\}, \quad X,Y \in \mathcal{M}_{\text{H}}. \tag{6}$$

  As noted above, we are interested in the Hausdorff metric since convergence to the spectrum in this metric means that our algorithms converge without spectral pollution or spectral invisibility.

We now define notions of error control for these two metric spaces. When $\mathcal{M}$ is a totally ordered set with relation $\leq$, such as $\mathbb{R}$ or $\mathbb{N}$, convergence from above or below is straightforward to define.

Definition 1.8 (Σ and Π classes for totally ordered sets)**.** Consider a collection $\mathcal{C}$ of computational problems and let $\mathcal{T}_\alpha$ be the collection of all towers of algorithms of type α. Suppose $\mathcal{M}$ is a totally ordered set. Set $\Sigma_0^\alpha = \Pi_0^\alpha = \Delta_0^\alpha$ and for $m \in \mathbb{N}$, define

$$\Sigma_m^\alpha = \left\{\{\Xi,\Omega,\mathcal{M},\Lambda\} \in \Delta_{m+1}^\alpha : \exists \ \{\Gamma_{n_m,\dots,n_1}\} \in \mathcal{T}_\alpha \text{ s.t. } \Gamma_{n_m}(A) \uparrow \Xi(A) \forall A \in \Omega\right\},$$
$$\Pi_m^\alpha = \left\{\{\Xi,\Omega,\mathcal{M},\Lambda\} \in \Delta_{m+1}^\alpha : \exists \ \{\Gamma_{n_m,\dots,n_1}\} \in \mathcal{T}_\alpha \text{ s.t. } \Gamma_{n_m}(A) \downarrow \Xi(A) \forall A \in \Omega\right\},$$

where $\uparrow$ and $\downarrow$ denote convergence from below and above, respectively. In other words, we have convergence from below or above in the final limit of the tower of algorithms.

The following two examples discuss $(\mathcal{M}, d) = \mathbb{R}$ and $\mathcal{M}_{\text{dec}}$, respectively.

Example 1.9 (Spectral radius of normal operators)**.** Let Ω be the class of bounded normal operators on $l^2(\mathbb{N})$, $\Lambda = \{A \mapsto \langle Ae_j, e_i\rangle : i,j \in \mathbb{N}\}$, and consider the spectral radius problem function $\Xi(A) = \sup_{z\in\text{Sp}(A)}|z|$. For normal operators, $\sup_{z\in\text{Sp}(A)}|z| = \|A\|$. Hence, we let $\Gamma_n(A)$ be an approximation of $\|\mathcal{P}_n A \mathcal{P}_n^*\|$ to accuracy $1/n$ from below, where $\mathcal{P}_n$ is the orthogonal projection onto $\text{span}\{e_1,\dots,e_n\}$. $\Gamma_n(A) \uparrow \|A\|$ and hence $\{\Xi,\Omega,\mathbb{R},\Lambda\} \in \Sigma_1^A$. This classification means that for any finite n, we obtain a lower bound for the value $\Xi(A)$. However, we may not know how close $\Gamma_n(A)$ is to $\|A\|$ and one can also show that $\{\Xi,\Omega,\mathbb{R},\Lambda\} \notin \Delta_1^G$. To see why, suppose for a contradiction that $\{\Xi,\Omega,\mathbb{R},\Lambda\} \in \Delta_1^G$. Hence, there exists a general algorithm Γ such that $|\Gamma(A) - \|A\|| \leq 1$ for all $A \in \Omega$. We may choose $A = \text{diag}(0,\dots,0,3,3,\dots)$ such that the number of zeros in the diagonal of A ensures that $\Gamma(A) = \Gamma(0)$ (this follows from the consistency requirement in the definition of a general algorithm). But then $1 \geq |\Gamma(A) - 3| = |\Gamma(0) - 3| \geq 3 - |\Gamma(0) - 0| \geq 2$, a contradiction. The point is that we cannot compute an upper bound on $\|A\|$ from a finite amount of information, and hence, we cannot get full error control in the metric space $\mathbb{R}$. ■

Example 1.10 (Is the spectral radius strictly larger than one?)**.** Consider the setup of Example 1.9, but now let Ξ be the decision problem, 'Is $\sup_{z\in\text{Sp}(A)}|z| > 1$?' Let $\Gamma_n(A)$ be as before, then if $\Xi(A) = 1$ (yes), $\Gamma_n(A) > 1$ for some n, otherwise $\Gamma_n(A) \leq 1$ for all n. Note that we have used the fact that $\{\Gamma_n\}$ is a Σ_1^A-tower for the problem in Example 1.9. It follows that

$$\tilde{\Gamma}_n(A) = \begin{cases} 1, & \text{if } \Gamma_n(A) > 1, \\ 0, & \text{otherwise.} \end{cases}$$

provides a Σ_1^A-tower for $\{\Xi,\Omega,\mathcal{M}_{\text{dec}},\Lambda\}$. Again, one can show that $\{\Xi,\Omega,\mathcal{M}_{\text{dec}},\Lambda\} \notin \Delta_1^G$. If we changed the decision problem to 'Is $\sup_{z\in\text{Sp}(A)}|z| \leq 1$?', we would obtain a Π_1^A classification instead. ■

More generally, the classes Σ_1^A and Π_1^A in Definition 1.8 allow *verification*. For example, suppose that we have a problem function $\Xi : \Omega \to \mathbb{R}$ and we wish to verify a theorem $\Xi(A) < 0$ for some $A \in \Omega$. If there exists a Π_1^A-tower $\{\Gamma_n\}$ for the problem and the theorem is true for the given A, then $\Gamma_n(A) < 0$ for sufficiently large n. We can compute $\Gamma_n(A)$ for various n and as soon as $\Gamma_n(A) < 0$, we know that $\Xi(A) \leq \Gamma_n(A) < 0$ and have verified the theorem. Note, however, that we cannot use a Π_1^A-tower to negate such a theorem (but can, instead, use a Σ_1^A-tower if it exists). We can only verify one way using a Π_1^A-tower. Similar remarks hold for Σ_1^A.

While Definition 1.8 is straightforward, it does not carry over to the more complicated Hausdorff metric. To define convergence of $\Gamma_n(A)$ to $\Xi(A)$ in the Hausdorff metric "from below", a first attempt may be to require that $\Gamma_n(A) \subset \Xi(A)$. However, this is severely restrictive. For example, when computing $\text{Sp}(A) \subset \mathbb{C}$, we can rarely ensure that a point z is exactly in $\text{Sp}(A)$. Nevertheless, we can often ensure that z is close to $\text{Sp}(A)$ and measure how close. Hence, it is natural to relax the condition $\Gamma_n(A) \subset \Xi(A)$ to $\sup_{z\in\Gamma_n(A)} \text{dist}(z, \Xi(A)) \leq 2^{-n}$. The exact form of the sequence $\{2^{-n}\}$ does not matter. What matters is that we can control the proximity of $\Gamma_n(A)$ to being contained within $\Xi(A)$ using a *known* sequence that converges to zero as $n \to \infty$. Based on this discussion, the following provides the generalization of Definition 1.8.

Definition 1.11 (Σ and Π classes for Hausdorff metric)**.** Consider a collection $\mathcal{C}$ of computational problems and let $\mathcal{T}_\alpha$ be the collection of all towers of algorithms of type α. Suppose that $(\mathcal{M}, d)$ is the Hausdorff metric. We set $\Sigma_0^\alpha = \Pi_0^\alpha = \Delta_0^\alpha$ and for $m \in \mathbb{N}$, we define

$$\Sigma_m^\alpha = \Big\{ \{\Xi, \Omega, \mathcal{M}, \Lambda\} \in \Delta_{m+1}^\alpha : \exists \{\Gamma_{n_m,\dots,n_1}\} \in \mathcal{T}_\alpha, \{X_{n_m}(A)\} \subset \mathcal{M} \text{ s.t. } \forall A \in \Omega$$
$$\Gamma_{n_m}(A) \subset X_{n_m}(A), \lim_{n_m \to \infty} \Gamma_{n_m}(A) = \Xi(A), d(X_{n_m}(A), \Xi(A)) \leq 2^{-n_m} \Big\},$$
$$\Pi_m^\alpha = \Big\{ \{\Xi, \Omega, \mathcal{M}, \Lambda\} \in \Delta_{m+1}^\alpha : \exists \{\Gamma_{n_m,\dots,n_1}\} \in \mathcal{T}_\alpha, \{X_{n_m}(A)\} \subset \mathcal{M} \text{ s.t. } \forall A \in \Omega$$
$$\Xi(A) \subset X_{n_m}(A), \lim_{n_m \to \infty} \Gamma_{n_m}(A) = \Xi(A), d(X_{n_m}(A), \Gamma_{n_m}(A)) \leq 2^{-n_m} \Big\}.$$

These classes capture convergence from below or above, up to a small error parameter 2^{-n}. It is precisely the classes Σ_1^α and Π_1^α that allow computations with verification, used, for example, in computer-assisted proofs. For example, to build a Σ_1^α algorithm in the case of the Hausdorff metric, it is enough to construct a convergent tower $\{\Gamma_n\}$ such that $\Gamma_n(A) \subset \Xi(A) + B_{E_n}(0)$ with some computable E_n that converges to zero.

1.2.5 Randomized algorithms

We also consider sequences of probabilistic general algorithms. Randomized algorithms are commonly used in machine learning and optimization. For example, in the context of Koopman operators, Monte Carlo methods that randomly sample the snapshots are common. We consider the following definition.

Definition 1.12 (Coin model)**.** Let $\mathcal{C} = \{0, 1\}^{\mathbb{N}}$, which we interpret as the set of possible outcomes of a countably infinite number of coin flips. We equip $\mathcal{C}$ with the product topology, where each flip's outcome set, $\{0, 1\}$, is given the discrete topology. Let p_j denote the projection map $p_j(a) = a_j$ for $a \in \mathcal{C}$ and $j \in \mathbb{N}$. We define $\mathbb{P}$ as the standard probability measure corresponding to an unbiased coin. That is, for any $n \in \mathbb{N}$ and any $a_1, \dots, a_n \in \{0, 1\}$, we have $\mathbb{P}(\{b \in \mathcal{C} : b_j = a_j, j = 1, \dots, n\}) = 2^{-n}$.

When an algorithm is given an input A from an input class Ω, we want it to be able to act on both the outcome of coin flips and A. This motivates the following definition of a probabilistic computational problem.

Definition 1.13 (Probabilistic computational problem)**.** Let $\{\Xi, \Omega, \mathcal{M}, \Lambda\}$ be a computational problem, as defined in Definition 1.1. The corresponding probabilistic computational problem is $\{\Xi^{\mathbb{P}}, \Omega^{\mathbb{P}}, \mathcal{M}^{\mathbb{P}}, \Lambda^{\mathbb{P}}\}$, where:

- $\Omega^{\mathbb{P}} = \Omega \times \mathcal{C} = \{(A, a) : A \in \Omega, a \in \mathcal{C}\}$;
- $\Xi^{\mathbb{P}}(A, a) = \Xi(A)$ for $(A, a) \in \Omega^{\mathbb{P}}$;
- $\mathcal{M}^{\mathbb{P}} = \mathcal{M} \cup \{\text{NH}\}$, where NH is added as an isolated point to $(\mathcal{M}, d)$;
- $\Lambda^{\mathbb{P}} = \{f : f(A, a) = f(A), f \in \Lambda\} \cup \{p_j : p_j(A, a) = p_j(a), j \in \mathbb{N}\}$.

The set $\Omega^{\mathbb{P}}$ is interpreted as an infinite sequence of coin flips for each input $A \in \Omega$. The evaluation set $\Lambda^{\mathbb{P}}$ consists of the original evaluation set and the ability to read the outcomes of the coin flips. The point NH added to $\mathcal{M}$ is interpreted as "non-halting", meaning that the algorithm never finishes computing to produce an output.

Example 1.14 (Why we include NH)**.** Suppose an algorithm repeatedly flips an unbiased coin. After each flip, if it sees "heads", it outputs the answer 1 and halts. Otherwise, it flips the coin again. With probability 1, this algorithm will halt and output 1. However, there is an outcome – an infinite sequence of tails – upon which the algorithm does not halt. ■

Definition 1.15 (Probabilistic general algorithm)**.** Given a computational problem $\{\Xi, \Omega, \mathcal{M}, \Lambda\}$, a probabilistic general algorithm is a map $\Gamma : \Omega^{\mathbb{P}} \to \mathcal{M} \cup \{\text{NH}\}$ with the following property. For any $(A, a) \in \Omega^{\mathbb{P}}$, there exists a non-empty subset of evaluations $\Lambda_\Gamma(A, a) \subset \Lambda^{\mathbb{P}}$ such that:

- If $(B, b) \in \Omega^{\mathbb{P}}$ with $f(A, a) = f(B, b)$ for every $f \in \Lambda_\Gamma(A, a)$, then $\Lambda_\Gamma(A, a) = \Lambda_\Gamma(B, b)$ and $\Gamma(A, a) = \Gamma(B, b)$;
- If $\Gamma(A, a) \neq \text{NH}$, then $\Lambda_\Gamma(A, a)$ is finite.

We will refer to a sequence of probabilistic general algorithms as an SPGA.

One can easily show that if Γ is a probabilistic general algorithm for a computational problem $\{\Xi, \Omega, \mathcal{M}, \Lambda\}$, then for any fixed $A \in \Omega$, the map $a \mapsto \Gamma(A, a)$ is measurable (and hence a random variable). Hence, we obtain an interpretation of a probabilistic general algorithm as a randomized algorithm. For $A \in \Omega$, we consider the possible outputs $\{\Gamma(A, a) : a \in \mathcal{C}\}$

with the probability measure $\mathbb{P}$. For example, given a sequence of probabilistic general algorithms (SPGA) $\{\Gamma_n\}$, we drop $a \in \mathcal{C}$ from the notation when there is no ambiguity and define

$$\mathbb{P}\left(\lim_{n\to\infty} \Gamma_n(A) = \Xi(A)\right) = \mathbb{P}\left(\left\{a \in \mathcal{C} : \lim_{n\to\infty} \Gamma_n(A,a) = \Xi^{\mathbb{P}}(A,a)\right\}\right).$$

The conditions of a probabilistic general algorithm hold for any standard probabilistic machine that flips coins (e.g., probabilistic Turing [3, Ch. 7] or BSS [11, Ch. 17] machines).[3] The critical point is that we have replaced consistency in the output of deterministic algorithms with consistency in the probability law of randomized algorithms.

Example 1.16 (Consistency)**.** Let Γ be a probabilistic general algorithm for a computational problem $\{\Xi, \Omega, \mathcal{M}, \Lambda\}$ and fix $A \in \Omega$. Let $S \subset \Lambda^{\mathbb{P}}$ and $B \in \Omega$ be such that $f(A) = f(B)$ for all $f \in S \cap \Lambda$. Then the consistency requirement in Definition 1.15 implies that

$$\{a \in \mathcal{C} : \Lambda_\Gamma(A,a) \subset S\} = \{a \in \mathcal{C} : \Lambda_\Gamma(B,a) \subset S\}.$$

Moreover, for any a in this set, we must have $\Gamma(B,a) = \Gamma(A,a)$ ■

Example 1.17 (Random guess for decision problems)**.** Suppose $\{\Xi, \Omega, \mathcal{M}, \Lambda\}$ is a decision problem with $\mathcal{M} = \mathcal{M}_{\mathrm{dec}} = \{0,1\}$. We can define a probabilistic general algorithm by setting $\Gamma(A,a) = p_1(a)$. If we set $\Gamma_n = \Gamma$, we find that $\mathbb{P}(\lim_{n\to\infty} \Gamma_n(A) = \Xi(A)) = 1/2$. However, if we instead set $\Gamma_n(A,a) = p_n(a)$, then $\mathbb{P}(\Gamma_n(A)$ does not converge in $\mathcal{M}_{\mathrm{dec}}) = 1$. ■

Example 1.18 (Independent sequence of probabilistic algorithms)**.** For certain problems, we may want to consider an SPGA $\{\Gamma_n\}$, where the coin flips of Γ_j and Γ_k are independent for $j \neq k$. This can be easily achieved by expressing the set of positive integers as a disjoint union of countably infinite sets, $\mathbb{N} = \cup_{j=1}^{\infty} T_j$. We then restrict Λ_{Γ_j} to be a subset of $\Lambda \cup \{p_k : k \in T_j\}$. Hence, we see that the definition of an SPGA captures computations that allow for independence or dependence between the different Γ_n's. ■

Example 1.19 (Random sampling of trajectories)**.** Suppose we sample snapshots randomly from $\mathcal{X}$ according to a probability distribution. Any random sampling produced by a digital computer and subsequent use of the data to produce an output is an example of a probabilistic general algorithm. Hence, the lower bounds we prove in this paper are universal. ■

A very useful property of probabilistic general algorithms is the following covering lemma. This tool enables us to reason similarly to deterministic algorithms with only an arbitrarily small loss in probability.

Lemma 1.20 (Finite ϵ-covering lemma)**.** *Let Γ be a probabilistic general algorithm for a computational problem $\{\Xi, \Omega, \mathcal{M}, \Lambda\}$ and fix $A \in \Omega$. For any $\epsilon > 0$, there exists a finite subset $S \subset \Lambda^{\mathbb{P}}$ such that*

$$\mathbb{P}(\Lambda_\Gamma(A,a) \subset S \text{ and } \Gamma(A,a) \neq \mathrm{NH}) \geq \mathbb{P}(\Gamma(A,a) \neq \mathrm{NH}) - \epsilon.$$

Proof. For any $S \subset \Lambda^{\mathbb{P}}$, define the set

$$U_S = \{a \in \mathcal{C} : \Lambda_\Gamma(A,a) \subset S, \Gamma(A,a) \neq \mathrm{NH}\}.$$

If $a \in U_S$, then there exists an open neighborhood of a such that any b in this neighborhood satisfies $\Lambda_\Gamma(A,a) = \Lambda_\Gamma(A,b)$. It follows that U_S is open. If $a \in \mathcal{C}$ has $\Gamma(A,a) \neq \mathrm{NH}$, then $\Lambda_\Gamma(A,a)$ is finite. It follows that

$$\{a \in \mathcal{C} : \Gamma(A,a) \neq \mathrm{NH}\} = \bigcup_{S \subset \Lambda^{\mathbb{P}}, S \text{ finite}} U_S.$$

Let U denote this set. Since U is a union of open sets, it is open. We shall prove that there exists a countable subcover of U.

Let $V \subset \mathcal{C}$ be the set of eventually constant coin flips. That is, the set of all $a \in \mathcal{C}$ such that the sequence $\{p_j(a)\}_{j=1}^{\infty}$ is eventually constant. The set V is countable and dense in $\mathcal{C}$. Since U is open, $V \cap U = \{v_1, v_2, \ldots\}$ is dense in U. For each $j \in \mathbb{N}$ and $n \in \mathbb{N}$, consider the open ball $D_{1/n}(v_j)$. If there exists a finite set $S \subset \Lambda^{\mathbb{P}}$ with $D_{1/n}(v_j) \subset U_S$, let $U_{j,n} = U_S$ for a choice of such an S. Otherwise, let $U_{j,n} = \emptyset$. Let $a \in U$. There exists a finite set $S \subset \Lambda^{\mathbb{P}}$ and $\delta > 0$ such that $D_\delta(a) \subset U_S$. We may choose $j, n \in \mathbb{N}$ such that $a \in D_{1/n}(v_j) \subset D_\delta(a)$. Hence, $U_{j,n} \neq \emptyset$ and $a \in U_{j,n}$. Since $a \in U$ was arbitrary, $U = \cup_{j,n=1}^{\infty} U_{j,n}$. If $S_1 \subset S_2$, then $U_{S_1} \subset U_{S_2}$. It follows that there exists an increasing sequence of finite sets $S_1 \subset S_2 \subset S_3 \subset \cdots \subset \Lambda^{\mathbb{P}}$ with

$$\{a \in \mathcal{C} : \Gamma(A,a) \neq \mathrm{NH}\} = \bigcup_{n=1}^{\infty} U_{S_n}.$$

Hence, $\mathbb{P}(\Gamma(A,a) \neq \mathrm{NH}) = \lim_{n\to\infty} \mathbb{P}(U_{S_n})$ and the lemma follows. □

[3]One could also consider other, even continuous, probability distributions. In the case of BSS machines, machines that can pick numbers uniformly at random in $[0,1]$ are no more computationally powerful [11, Section 17.5]. Hence, we do not consider such scenarios, which are also unrealistic in practice.

We can now make sense of probabilistic classes in the SCI hierarchy.

Definition 1.21 (Probabilistic classes in the SCI hierarchy)**.** A computational problem $\{\Xi, \Omega, \mathcal{M}, \Lambda\}$ does not belong to $\Delta_1^{\mathbb{P},1/2}$ if for any SPGA $\{\Gamma_n\}$,

$$\inf_{A\in\Omega} \mathbb{P}\left(d(\Gamma_n(A), \Xi(A)) \leq 2^{-n} \text{ for all } n \in \mathbb{N}\right) \leq 1/2.$$

A computational problem $\{\Xi, \Omega, \mathcal{M}, \Lambda\}$ does not belong to $\Delta_2^{\mathbb{P},1/2}$ if for any SPGA $\{\Gamma_n\}$,

$$\inf_{A\in\Omega} \mathbb{P}\left(\lim_{n\to\infty} \Gamma_n(A) = \Xi(A)\right) \leq 1/2.$$

Since a sequence of general algorithms is an SPGA, if $\{\Xi, \Omega, \mathcal{M}, \Lambda\} \notin \Delta_1^{\mathbb{P},1/2}$, then $\{\Xi, \Omega, \mathcal{M}, \Lambda\} \notin \Delta_1^G$. Similarly, if $\{\Xi, \Omega, \mathcal{M}, \Lambda\} \notin \Delta_2^{\mathbb{P},1/2}$, then $\{\Xi, \Omega, \mathcal{M}, \Lambda\} \notin \Delta_2^G$.

1.3 The setup of this paper – a perfect measurement device (which implies stronger results)

In this paper, we consider computational problems where:

- Ω is a class of dynamical systems, or F, on some state space $\mathcal{X}$ as in (1);
- Λ, the evaluation set, will be pointwise evaluations of the function F:

$$\Lambda = \Lambda_{\mathcal{X}} = \{F \mapsto F(\hat{x}_j) : j = 1, 2, \ldots\},$$

  where $\{\hat{x}_j\}_{j=1}^{\infty}$ is a dense subset of $(\mathcal{X}, d_{\mathcal{X}})$.
- The metric space $(\mathcal{M}, d)$ will be either $\mathcal{M}_{\text{dec}}$, in the case of decision problems, or $\mathcal{M}_{\text{H}}$, when we compute spectral sets.

In general, the choice of evaluation set matters [15]. The above setup agrees with the usual "snapshot" setting of data-driven Koopmanism, where one is given access to a finite collection of pairs

$$\left\{\left(x^{(m)}, y^{(m)} = F(x^{(m)})\right) : m = 1, \ldots, M\right\}.$$

However, our lower bounds become *stronger*. Specifically, this strengthening occurs when we prove lower bounds for a computational problem $\{\Xi, \Omega, \mathcal{M}, \Lambda_{\mathcal{X}}\}^{\Delta_1}$, which we remind the reader, corresponds to allowing arbitrary precision of the evaluation set according to Definition 1.7. Hence, our lower bound holds even if we allow algorithms arbitrarily many point samples of F to arbitrary precision. When proving lower bounds, we will always deal with the case that ω is the natural Lebesgue measure on $\mathcal{X} \subset \mathbb{R}^d$, though many of our results can be easily extended to other measures.

Remark 1.22 (Simplified notation)**.** To simplify notation, we drop the superscript Δ_1 from the notation throughout the results below. We also drop the superscript α from the computational classes, e.g., writing Σ_1. Our lower bounds are proven for $\alpha = G$ (general algorithms), and our upper bounds can generally be made to work with $\alpha = A$ (arithmetic algorithms). Hence, we obtain the strongest possible results regarding computational models. ■

2 Theorems that tell us what is possible and what is not possible

We now provide statements of our theorems. These theorems precisely state the results written in the main text, corresponding to classifications in the SCI hierarchy. Whenever we prove an upper bound in the SCI hierarchy (i.e., construct a convergent tower of algorithms), we shall reference the corresponding pseudocode given in Section 3.

2.1 A general computational result – upper bounds or "possibility results"

In this section, we let $(\mathcal{X}, d_{\mathcal{X}})$ be a compact metric space and ω a finite Borel measure on $(\mathcal{X}, d_{\mathcal{X}})$. Under these assumptions, there are two key properties of $L^2(\mathcal{X}, \omega)$ that we shall make use of:

- $L^2(\mathcal{X}, \omega)$ is a separable Hilbert space [17, Proposition 3.4.5];
- The space of continuous functions on $\mathcal{X}$, denoted $C(\mathcal{X})$, is dense in $L^2(\mathcal{X}, \omega)$ [33, Proposition 7.9].

In particular, by the Gram–Schmidt process, there exists an orthonormal basis $\{g_1, g_2, \ldots\} \subset C(\mathcal{X})$ of $L^2(\mathcal{X}, \omega)$.

Definition 2.1. Let $\alpha : \mathbb{R}_{\geq 0} \to \mathbb{R}_{\geq 0}$ be an increasing continuous function with $\alpha(0) = 0$. We say that a continuous function $F : \mathcal{X} \to \mathcal{X}$ has a modulus of continuity α if

$$d_{\mathcal{X}}(F(x), F(y)) \leq \alpha(d_{\mathcal{X}}(x, y)) \quad \forall x, y \in \mathcal{X}. \tag{7}$$

Since $\mathcal{X}$ is compact, any continuous function $F : \mathcal{X} \to \mathcal{X}$ is uniformly continuous and hence has a choice of α so that Equation (7) holds. However, there is no α such that Equation (7) holds universally for all continuous functions. We set

$$\begin{aligned}
\Omega_{\mathcal{X}} &= \{F : \mathcal{X} \to \mathcal{X} \text{ such that } F \text{ is continuous and non-singular, } \mathcal{K}_F \text{ is bounded}\}, \\
\Omega_{\mathcal{X}}^{\alpha} &= \{F : \mathcal{X} \to \mathcal{X} \text{ such that } F \text{ has a modulus of continuity } \alpha \text{ and is non-singular, } \mathcal{K}_F \text{ is bounded}\}, \\
\Omega_{\mathcal{X}}^{m} &= \{F : \mathcal{X} \to \mathcal{X} \text{ such that } F \text{ is measure-preserving}\}, \\
\Omega_{\mathcal{X}}^{\alpha,m} &= \Omega_{\mathcal{X}}^{\alpha} \cap \Omega_{\mathcal{X}}^{m}.
\end{aligned}$$

We consider the problem functions $\Xi_{\mathrm{Sp}_{\mathrm{ap}}}(F) = \mathrm{Sp}_{\mathrm{ap}}(\mathcal{K}_F)$ and $\Xi_{\mathrm{Sp}_{\mathrm{ap},\epsilon}}(F) = \mathrm{Sp}_{\mathrm{ap},\epsilon}(\mathcal{K}_F)$ for any $\epsilon > 0$.

Example 2.2 (EDMD does not work for $\Omega_{\mathcal{X}}^{\alpha,m}$, from [52]). Let $\mathcal{X} = \mathbb{T}$ (the unit circle), equipped with the usual measure ω, and consider the doubling map $F(x) = x^2$. To apply the algorithm EDMD, we use the Fourier basis $\psi_j(x) = x^j/\sqrt{2\pi}$, $j \in \mathbb{Z}$. Note that $\mathcal{K}_F\psi_j = \psi_{2j}$ and $\mathrm{Sp}(\mathcal{K}_F) = \{z \in \mathbb{C} : |z| \leq 1\}$ ($\mathcal{K}_F$ is an isometry whose range is a strict subspace of $L^2(\mathcal{X}, \omega)$). We may split the space $L^2(\mathcal{X}, \omega)$ into invariant subspaces as follows. Let $n \in \mathbb{N}$ be odd, then $\mathcal{K}_F$ acts as a unilateral shift on $\mathrm{span}\{\psi_{n2^k} : k = 0, 1, \ldots\}$ and $\mathrm{span}\{\psi_{-n2^k} : k = 0, 1, \ldots\}$. Hence, $\mathcal{K}_F$ acts as a direct sum of unilateral shifts. If we use a finite number of Fourier basis functions as our dictionary, the large data limit of EDMD is a direct sum of finite sections of unilateral shifts. These finite matrices have spectrum $\{0\}$, and hence, we completely miss regions of the spectrum. ■

The above example shows that methods such as EDMD do not converge, even for the class $\Omega_{\mathcal{X}}^{\alpha,m}$. This kind of argument can also be extended to systems in $\Omega_{\mathcal{X}}^{\alpha,m}$ whose Koopman operator is not just an isometry, but also unitary (e.g., Arnold's cat map). Nevertheless, part of the following theorem says we can use a different algorithm to ensure convergence.

Theorem 2.3. *Given the above setup, for $\epsilon > 0$, we have the following classifications:*

$$\begin{aligned}
\{\Xi_{\mathrm{Sp}_{\mathrm{ap},\epsilon}}, \Omega_{\mathcal{X}}, \mathcal{M}_{\mathrm{H}}, \Lambda_{\mathcal{X}}\} &\in \Sigma_2, & \{\Xi_{\mathrm{Sp}_{\mathrm{ap}}}, \Omega_{\mathcal{X}}, \mathcal{M}_{\mathrm{H}}, \Lambda_{\mathcal{X}}\} &\in \Pi_3 \\
\{\Xi_{\mathrm{Sp}_{\mathrm{ap},\epsilon}}, \Omega_{\mathcal{X}}^{\alpha}, \mathcal{M}_{\mathrm{H}}, \Lambda_{\mathcal{X}}\} &\in \Sigma_1, & \{\Xi_{\mathrm{Sp}_{\mathrm{ap}}}, \Omega_{\mathcal{X}}^{\alpha}, \mathcal{M}_{\mathrm{H}}, \Lambda_{\mathcal{X}}\} &\in \Pi_2 \\
\{\Xi_{\mathrm{Sp}_{\mathrm{ap},\epsilon}}, \Omega_{\mathcal{X}}^{m}, \mathcal{M}_{\mathrm{H}}, \Lambda_{\mathcal{X}}\} &\in \Sigma_2, & \{\Xi_{\mathrm{Sp}_{\mathrm{ap}}}, \Omega_{\mathcal{X}}^{m}, \mathcal{M}_{\mathrm{H}}, \Lambda_{\mathcal{X}}\} &\in \Sigma_2 \\
\{\Xi_{\mathrm{Sp}_{\mathrm{ap},\epsilon}}, \Omega_{\mathcal{X}}^{\alpha,m}, \mathcal{M}_{\mathrm{H}}, \Lambda_{\mathcal{X}}\} &\in \Sigma_1, & \{\Xi_{\mathrm{Sp}_{\mathrm{ap}}}, \Omega_{\mathcal{X}}^{\alpha,m}, \mathcal{M}_{\mathrm{H}}, \Lambda_{\mathcal{X}}\} &\in \Sigma_1.
\end{aligned}$$

Proof. **Step 1:** Classifications for $\Omega_{\mathcal{X}}^{\alpha}$. Given $F \in \Omega_{\mathcal{X}}^{\alpha}$, we consider the *infinite* matrices A and L acting on $l^2(\mathbb{N})$ with

$$A_{i,j} = \langle \mathcal{K}_F g_j, g_i \rangle = \int_{\mathcal{X}} g_j(F(x))\overline{g_i(x)}\,\mathrm{d}\omega(x), \quad L_{i,j} = \langle \mathcal{K}_F^* \mathcal{K}_F g_j, g_i \rangle = \langle \mathcal{K}_F g_j, \mathcal{K}_F g_i \rangle = \int_{\mathcal{X}} g_j(F(x))\overline{g_i(F(x))}\,\mathrm{d}\omega(x), \quad i, j \in \mathbb{N}.$$

We first claim that if $F \in \Omega_{\mathcal{X}}^{\alpha}$, then given any $i, j \in \mathbb{N}$ and $\delta > 0$, there exists a general algorithm using Δ_1-information that computes an approximation of $A_{i,j}$ and $L_{i,j}$ within an error bounded by δ. We show this for $A_{i,j}$, and the case of $L_{i,j}$ is similar.

Since $\mathcal{X}$ is a compact metric space, given any $\eta > 0$, there exists a finite subset $\{x_{1,\eta}, \ldots, x_{N_\eta,\eta}\} \subset \mathcal{X}$ and continuous functions $\rho_{p,\eta} : \mathcal{X} \to [0, 1]$ such that

$$\sum_{p=1}^{N_\eta} \rho_{p,\eta}(x) = 1 \quad \forall x \in \mathcal{X}, \quad \mathrm{supp}(\rho_{p,\eta}) \subset \{x \in \mathcal{X} : d_{\mathcal{X}}(x, x_{p,\eta}) < \eta\} \text{ for } p = 1, \ldots, N_\eta.$$

By slightly changing the points $\{x_{j,\eta}\}$ if necessary, we may assume that $\{x_{1,\eta}, \ldots, x_{N_\eta,\eta}\} \subset \{\hat{x}_j\}_{j=1}^{\infty}$. Let $y_{p,\eta}$ be an approximation of $F(x_{p,\eta})$ to accuracy η, which can be computed from the given Δ_1-information. We then approximate the integral $A_{i,j}$ by

$$\Gamma_\eta(F) = \sum_{p=1}^{N_\eta} \int_{\mathcal{X}} \rho_{p,\eta}(x) g_j(y_{p,\eta}) \overline{g_i(x)}\,\mathrm{d}\omega(x).$$

To bound the error in this approximation, note that if $x \in \mathrm{supp}(\rho_{p,\eta})$, then $d_{\mathcal{X}}(x, x_{p,\eta}) < \eta$. Recall that α is a modulus of continuity for F. Let α_j be a modulus of continuity for g_j, then

$$\begin{aligned}
|g_j(F(x)) - g_j(y_{p,\eta})| &\leq |g_j(F(x)) - g_j(F(x_{p,\eta}))| + |g_j(F(x_{p,\eta})) - g_j(y_{p,\eta})| \\
&\leq \alpha_j(d_{\mathcal{X}}(F(x), F(x_{p,\eta}))) + \alpha_j(d_{\mathcal{X}}(F(x_{p,\eta}), y_{p,\eta})) \leq \alpha_j(\alpha(\eta)) + \alpha_j(\eta).
\end{aligned}$$

It follows that

$$|A_{i,j} - \Gamma_\eta(F)| \leq \int_{\mathcal{X}} \sum_{p=1}^{N_\eta} \rho_{p,\eta}(x)|g_j(F(x)) - g_j(y_{p,\eta})||g_i(x)| \,\mathrm{d}\omega(x) \leq [\alpha_j(\alpha(\eta)) + \alpha_j(\eta)] \int_{\mathcal{X}} |g_i(x)| \,\mathrm{d}\omega(x).$$

We can make this bound smaller than a given $\delta > 0$ by choosing η sufficiently small.

For a given $n \in \mathbb{N}$, let $\mathcal{P}_n$ be the orthogonal projection onto the span of the first n canonical basis vectors of $l^2(\mathbb{N})$. We define the function

$$h_n(z, F) = \sigma_{\inf}((A - zI)\mathcal{P}_n^*), \quad \text{where} \quad \sigma_{\inf}(T) = \inf\{\|Tx\| : \|x\| = 1\},$$

and we view $(A - zI)\mathcal{P}_n^*$ as an operator from the range of $\mathcal{P}_n$ to $l^2(\mathbb{N})$. Since $A^*A = L$, we can rewrite h_n as

$$h_n(z, F) = \sqrt{\sigma_{\inf}(\mathcal{P}_n(A - zI)^*(A - zI)\mathcal{P}_n^*)} = \sqrt{\sigma_{\inf}(\mathcal{P}_n[L - \overline{z}A - zA^* + |z|^2 I]\mathcal{P}_n^*)}.$$

In particular, the operator $\mathcal{P}_n[L - \overline{z}A - zA^* + |z|^2 I]\mathcal{P}_n^*$ is built from a finite matrix truncation of A and L. It follows that we may compute $h_n(z, F)$ to any desired accuracy using finitely many evaluations of F to a given precision. By Dini's theorem, $h_n(z, F)$ converges locally uniformly to the function $\sigma_{\inf}(A - zI)$. We may now apply the general construction of [25] to see that $\{\Xi_{\mathrm{Sp}_{\mathrm{ap},\epsilon}}, \Omega_{\mathcal{X}}^\alpha, \mathcal{M}_{\mathrm{H}}, \Lambda_{\mathcal{X}}\} \in \Sigma_1$. Namely, we let $\tilde{h}_n$ be an approximation of h_n computed to accuracy $1/n$ and set

$$\gamma_n(F) = \left\{z \in \frac{1}{n}(\mathbb{Z} + i\mathbb{Z}) \cap B_n(0) : \tilde{h}_n(z, F) + \frac{1}{n} < \epsilon\right\} \subset \mathrm{Sp}_{\mathrm{ap},\epsilon}(\mathcal{K}_F),$$

which converges to $\mathrm{Sp}_{\mathrm{ap},\epsilon}(\mathcal{K}_F)$ as $n \to \infty$. The method is summarized in Supplementary Algorithm 2. Note that $\mathrm{Sp}_{\mathrm{ap}}(\mathcal{K}_F) \subset \mathrm{Sp}_{\mathrm{ap},\epsilon}(\mathcal{K}_F)$ and that $\lim_{\epsilon \downarrow 0} \mathrm{Sp}_{\mathrm{ap},\epsilon}(\mathcal{K}_F) = \mathrm{Sp}_{\mathrm{ap}}(\mathcal{K}_F)$. It follows from the classification for $\Xi_{\mathrm{Sp}_{\mathrm{ap},\epsilon}}$ that $\{\Xi_{\mathrm{Sp}_{\mathrm{ap}}}, \Omega_{\mathcal{X}}^\alpha, \mathcal{M}_{\mathrm{H}}, \Lambda_{\mathcal{X}}\} \in \Pi_2$. This method is summarized in Supplementary Algorithm 3.

Step 2: Classifications for $\Omega_{\mathcal{X}}$. The proof is similar to the $\Omega_{\mathcal{X}}^\alpha$ case, but we do not assume access to α, a modulus of continuity for F. It follows that we can compute any matrix element $A_{i,j}$ or $L_{i,j}$ in one limit without error control. In particular, we let $h_{n_2,n_1}(z, F)$ be functions that we compute with $\lim_{n_1 \to \infty} h_{n_2,n_1}(z, F) = h_{n_2}(z, F)$. However, the set

$$\left\{z \in \frac{1}{n_2}(\mathbb{Z} + i\mathbb{Z}) \cap B_{n_2}(0) : h_{n_2,n_1}(z, F) < \epsilon\right\}$$

need not converge as $n_1 \to \infty$, since the convergence $\lim_{n_1 \to \infty} h_{n_2,n_1}(z, F) = h_{n_2}(z, F)$ need not be monotonic. To fix this, we define $\Gamma_{n_2,n_1}(F)$ as follows. Let $z \in \frac{1}{n_2}(\mathbb{Z} + i\mathbb{Z}) \cap B_{n_2}(0)$ and consider the separated intervals

$$I_{n_2}^1(\epsilon) = [0, \epsilon - 1/n_2], \quad I_{n_2}^2(\epsilon) = [\epsilon + 1/(2n_2), \infty).$$

Given $h_{n_2,j}(z, F)$ for $j = 1, \ldots, n_1$, let k be the largest such j with $h_{n_2,j}(z, F) \in I_{n_2}^1(\epsilon) \cup I_{n_2}^2(\epsilon)$. If such a k exists with $h_{n_2,k}(z, F) \in I_{n_2}^1(\epsilon)$, then $z \in \Gamma_{n_2,n_1}(F)$. Otherwise, $z \notin \Gamma_{n_2,n_1}(F)$. Since the sequence $h_{n_2,j}(z, F)$ cannot visit both intervals $I_{n_2}^1(\epsilon)$ and $I_{n_2}^2(\epsilon)$ infinitely often as $n_1 \to \infty$, it follows that the limit $\lim_{n_1 \to \infty} \Gamma_{n_2,n_1}(F) = \Gamma_{n_2}(F)$ exists. Moreover,

$$\left\{z \in \frac{1}{n_2}(\mathbb{Z} + i\mathbb{Z}) \cap B_{n_2}(0) : h_{n_2}(z, F) < \epsilon - \frac{1}{2n_2}\right\} \subset \Gamma_{n_2}(F) \subset \left\{z \in \frac{1}{n_2}(\mathbb{Z} + i\mathbb{Z}) \cap B_{n_2}(0) : h_{n_2}(z, F) < \epsilon\right\} \subset \mathrm{Sp}_{\mathrm{ap},\epsilon}(\mathcal{K}_F).$$

Hence, $\{\Gamma_{n_2,n_1}\}$ is a Σ_2-tower for $\{\Xi_{\mathrm{Sp}_{\mathrm{ap},\epsilon}}, \Omega_{\mathcal{X}}, \mathcal{M}_{\mathrm{H}}, \Lambda_{\mathcal{X}}\}$. The method is summarized in Supplementary Algorithm 4. Again by taking $\epsilon \downarrow 0$, we see that $\{\Xi_{\mathrm{Sp}_{\mathrm{ap}}}, \Omega_{\mathcal{X}}, \mathcal{M}_{\mathrm{H}}, \Lambda_{\mathcal{X}}\} \in \Pi_3$. This method is summarized in Supplementary Algorithm 5.

Step 3: Classifications for $\Omega_{\mathcal{X}}^m$. Since $\Omega_{\mathcal{X}}^m \subset \Omega_{\mathcal{X}}$, we need only prove the classification for the problem function $\Xi_{\mathrm{Sp}_{\mathrm{ap}}}$. Note that if $F \in \Omega_{\mathcal{X}}^m$, then $\mathcal{K}_F$ is an isometry. It follows from the Wold–von Neumann decomposition [53, Theorem I.1.1] that $\mathcal{K}_F$ can be written as a direct sum of copies of the unilateral shift and a unitary operator. In particular,

$$\sigma_{\inf}(\mathcal{K}_F - zI) = \mathrm{dist}(z, \mathrm{Sp}_{\mathrm{ap}}(\mathcal{K}_F)), \quad \mathrm{Sp}_{\mathrm{ap},\epsilon}(\mathcal{K}_F) = \mathrm{Sp}_{\mathrm{ap}}(\mathcal{K}_F) + B_\epsilon(0).$$

We can convert the Σ_2 tower for $\Xi_{\mathrm{Sp}_{\mathrm{ap},\epsilon}}$ to a Σ_2 tower for $\Xi_{\mathrm{Sp}_{\mathrm{ap}}}$ following the same argument for self-adjoint operators in [7].

Step 4: Classifications for $\Omega_{\mathcal{X}}^{\alpha,m}$. Again, if $F \in \Omega_{\mathcal{X}}^m$, then

$$\sigma_{\inf}(\mathcal{K}_F - zI) = \mathrm{dist}(z, \mathrm{Sp}_{\mathrm{ap}}(\mathcal{K}_F)), \quad \mathrm{Sp}_{\mathrm{ap},\epsilon}(\mathcal{K}_F) = \mathrm{Sp}_{\mathrm{ap}}(\mathcal{K}_F) + B_\epsilon(0).$$

We can argue as in step 1 for $\Xi_{\mathrm{Sp}_{\mathrm{ap},\epsilon}}$. We can convert the Σ_1 tower for $\Xi_{\mathrm{Sp}_{\mathrm{ap},\epsilon}}$ to a Σ_1 tower for $\Xi_{\mathrm{Sp}_{\mathrm{ap}}}$ as in [25]. The method is summarized in Supplementary Algorithm 1. □

Remark 2.4. It is remarkable that there is a constructive computational procedure for $\{\Xi_{\mathrm{Sp}_{\mathrm{ap}}}, \Omega_{\mathcal{X}}, \mathcal{M}_{\mathrm{H}}, \Lambda_{\mathcal{X}}\}$. The procedure involves three successive limits. The first limit is the large data limit, collecting more snapshots of the dynamical system. The second limit is the large subspace limit, observing the action of the Koopman operator on more observables. The final limit is the computation of spectra through pseudospectra, which is also how the classical computational spectral problem was solved [36]. This phenomenon of several successive limits occurs in all algorithms for Koopman operators that provably converge. In particular, the above argument using the matrices L is a generalization of the ResDMD algorithm [27, 24]. We shall see below that several successive limits are necessary unless we can control the large data limit (e.g., using a modulus of continuity) and the pseudospectra limit (e.g., by assuming that the system is measure-preserving so that $\sigma_{\inf}(\mathcal{K}_F - zI) = \mathrm{dist}(z, \mathrm{Sp}_{\mathrm{ap}}(\mathcal{K}_F))$, see Definition 3.1 for a generalization). ■

Remark 2.5. In the above proof, we used a basis constructed for $\mathcal{X}$ and assumed that certain system-independent integrals with respect to the measure ω could be computed. Once $\mathcal{X}$ and ω are fixed, this still defines a general algorithm. To obtain an arithmetic algorithm, the basis construction must depend on the state space and measure. Practical examples of this are discussed in Section 4.4. Methods for learning a well-conditioned dictionary include, for example, those in [29, 34, 9]. Proving the SCI of basis construction lies beyond the scope of this paper, but represents an interesting direction for future work. ■

2.2 Measure-preserving maps on the unit disk

In Theorem 2.3, we saw that the approximate point spectrum of Koopman operators associated with measure-preserving dynamical systems can be computed in one limit if we have a bound on the modulus of continuity of F, and two limits otherwise. We now show that this classification is sharp by proving a lower bound. We consider invertible, measure-preserving dynamical systems on the closed unit disk $\mathcal{X} = \mathrm{Cl}(\mathbb{D}) \subset \mathbb{R}^2$, equipped with the Euclidean metric and standard Lebesgue measure. We set

$$\Omega_{\mathbb{D}} = \{F : \mathrm{Cl}(\mathbb{D}) \to \mathrm{Cl}(\mathbb{D}) \text{ such that } F \text{ is continuous, measure-preserving and invertible}\}$$

and also consider maps with a priori known Lipschitz constant,

$$\Omega_{\mathbb{D}}^{L} = \{F \in \Omega_{\mathbb{D}} : \mathrm{Lip}(F) \leq L\},$$

where $\mathrm{Lip}(F)$ is the (optimal) Lipschitz constant if F is Lipschitz and $+\infty$ otherwise.

Theorem 2.6. *Given the above setup, we have the following classifications for the spectrum:*

$$\Delta_2^{\mathbb{P},1/2} \not\ni \{\Xi_{\mathrm{Sp}}, \Omega_{\mathbb{D}}, \mathcal{M}_{\mathrm{H}}, \Lambda_{\mathrm{Cl}(\mathbb{D})}\} \in \Sigma_2, \qquad \Delta_1^{\mathbb{P},1/2} \not\ni \{\Xi_{\mathrm{Sp}}, \Omega_{\mathbb{D}}^{L}, \mathcal{M}_{\mathrm{H}}, \Lambda_{\mathrm{Cl}(\mathbb{D})}\} \in \Sigma_1.$$

The same classifications hold for the pseudospectrum Sp_ϵ *(and the approximate point spectrum and approximate point pseudospectrum). In other words, to compute the spectral sets in one limit, we must be able to bound the variability of* F*.*

Let $F_0 \in \Omega_{\mathbb{D}}$ be the map $(r, \theta) \mapsto (r, \theta + \pi)$, where we use polar coordinates. Using the eigenfunctions of the Dirichlet Laplacian, we see that $\mathrm{Sp}(\mathcal{K}_{F_0}) = \{\pm 1\}$. To prove Theorem 2.6, we will need the following technical lemma regarding F_0.

Lemma 2.7. *Let* $\mathcal{A} = \{x \in \mathbb{R}^2 : 0 < R \leq |x| \leq r < 1\}$ *be an annulus and* $X = \{x_1, \dots, x_N\}$ *and* $Y = \{y_1, \dots, y_N\}$ *two sets of* N *distinct points in* $\mathcal{A}$ *with* $X \cap Y = \emptyset$*. Then there exists a measure-preserving homeomorphism* H *that acts as the identity on* $\mathrm{Cl}(\mathbb{D}) \backslash \mathcal{A}$ *such that* $H^{-1} \circ F_0 \circ H(x_j) = y_j$ *for* $j = 1, \dots, N$*.*

Proof. It suffices to show there exists a measure-preserving homeomorphism H that acts as the identity on $\mathrm{Cl}(\mathbb{D}) \backslash \mathcal{A}$ with

$$H(x_j) = (r_j, 0), \quad H(y_j) = (r_j, \pi), \quad j = 1, \dots, N,$$

where we employ polar coordinates and $r > r_1 > \cdots > r_N > R$. Consider a family $\mathcal{G}$ of nested, smooth, closed simple curves filling $\mathrm{Cl}(\mathbb{D})$ such that $\mathcal{G}$ consists of concentric circles on $\mathrm{Cl}(\mathbb{D}) \backslash \mathcal{A}$ and otherwise all curves enclose $R \cdot \overline{\mathbb{D}}$. We can construct such a family $\mathcal{G}$ so that x_j and y_j lie on the same curve in $\mathcal{G}$ for all j. Let $\mathfrak{c}$ be a Jordan arc from $\partial\mathbb{D}$ to the origin, such that $\mathfrak{c}$ meets each curve in $\mathcal{G}$ at a single point. Assume that $\mathfrak{c}$ passes through x_j for all j, starting with x_1 then passing through $x_2, \dots, x_N$ consecutively on the way to the origin (after reordering the pairs (x_j, y_j) if necessary). Without loss of generality, $\mathcal{G}$ may be represented implicitly by a smooth positive function on $\mathrm{Cl}(\mathbb{D})$ via the formula $f(x) = a$, $0 < a < 1$ with $f(x_1) > \cdots > f(x_N)$. By [12, Theorem 1], there exists a measure-preserving homeomorphism H, acting as the identity on $\mathrm{Cl}(\mathbb{D}) \backslash \mathcal{A}$, which maps each family of curves $\mathcal{G}$ to a family of nested concentric circles in $\mathrm{Cl}(\mathbb{D})$ about the origin. Furthermore, the theorem ensures that H maps the chord $\mathfrak{c}$ onto the radius of $\mathrm{Cl}(\mathbb{D})$ corresponding to zero angular coordinates. It follows that $H(x_j) = (r_j, 0)$ and $|H(y_j)| = r_j$ for all j.

It remains to prove that we can choose f so that the angular coordinate of $H(y_j)$ is π. We employ the formula for H provided in [12, Equations 3 and 4],

$$|H(x)| = f(x), \qquad \theta = \frac{1}{f(x)} \int_{T(x)}^{x} \frac{\mathrm{d}s}{f_n} = \frac{1}{f(x)} \int_{T(x)}^{x} \frac{\mathrm{d}s}{\sqrt{[\partial_1 f(s)]^2 + [\partial_2 f(s)]^2}}, \tag{8}$$

where $T(x)$ is the point of intersection with $\mathfrak{c}$ of the curve of $\mathcal{G}$ through x, f_n is the directional derivative in the outer normal direction, and the integral is evaluated along the curve of $\mathcal{G}$ with s measured in the sense for which the interior is on the left. Let θ_j denote the angular coordinate of $H(y_j)$. By adjusting f only in a small neighborhood of the curve passing through x_j and y_j (avoiding the curves passing through the other points) and making f_n small or large along the path of integration from $T(y_j) = x_j$ to y_j, we can ensure that $\theta_j = \pi$. □

Proof of Theorem 2.6. We split the proof into three parts: upper bounds, and proving lower bounds for $\Omega_{\mathbb{D}}$ and $\Omega_{\mathbb{D}}^L$.

Step 1: Upper bounds. To prove the upper bounds, we must alter the proof of Theorem 2.3 to arithmetic algorithms. We let $\{g_n\}_{n=1}^{\infty}$ be an orthonormal basis of $L^2(\mathbb{D})$ made up of the eigenfunctions of the Dirichlet Laplacian. These functions can be explicitly expressed in terms of Fourier basis functions in the angle coordinate and Bessel functions of the first kind in the radial coordinate. Let $F \in \Omega_{\mathbb{D}}^L$ and consider the two types of integrals

$$A_{i,j} = \langle \mathcal{K}_F g_j, g_i \rangle = \int_{\mathbb{D}} g_j(F(x))\overline{g_i(x)}\,\mathrm{d}x, \quad L_{i,j} = \langle \mathcal{K}_F^* \mathcal{K}_F g_j, g_i \rangle = \langle \mathcal{K}_F g_j, \mathcal{K}_F g_i \rangle = \int_{\mathbb{D}} g_j(F(x))\overline{g_i(F(x))}\,\mathrm{d}x, \quad i,j \in \mathbb{N}.$$

We can bound the modulus of continuity and modulus of the products $g_j(F(x))\overline{g_i(x)}$ and $g_j(F(x))\overline{g_i(F(x))}$. It follows that the integrals can be computed to any desired accuracy using quadrature, for example, Riemann sums. (In practice, other choices may be better.) Similarly, if $F \in \Omega_{\mathbb{D}}$, we can construct arithmetic algorithms that converge to these inner products without error control. The rest of the proofs of upper bounds follow those of the upper bounds in Theorem 2.3.

Step 2: $\{\Xi_{\mathrm{Sp}}, \Omega_{\mathbb{D}}, \mathcal{M}_{\mathrm{H}}, \Lambda_{\mathrm{Cl}(\mathbb{D})}\} \notin \Delta_2^{\mathbb{P},1/2}$. Suppose for a contradiction that $\{\Gamma_n\}$ is an SPGA for $\{\Xi_{\mathrm{Sp}}, \Omega_{\mathbb{D}}, \mathcal{M}_{\mathrm{H}}, \Lambda_{\mathrm{Cl}(\mathbb{D})}\}$ such that

$$\inf_{F \in \Omega_{\mathbb{D}}} \mathbb{P}\left(\lim_{n\to\infty} \Gamma_n(\tilde{F}) = \mathrm{Sp}(\mathcal{K}_F)\right) = \epsilon > 1/2. \tag{9}$$

Here, $\tilde{F}$ represents inexact input for F. That is, an admissible $\tilde{F}$ is of the form $\{f_{j,n}(F) : j, n \in \mathbb{N}\}$, where $\|f_{j,n}(F) - F(\hat{x}_j)\| \leq 2^{-n}$. We will obtain a contradiction by constructing an $F \in \Omega_{\mathbb{D}}$ and corresponding $\tilde{F}$ so that Equation (9) cannot hold. Throughout the proof, we work in polar coordinates $x = (r, \theta)$ in $\mathrm{Cl}(\mathbb{D})$. We also fix a smooth surjective bump function $\phi : [0,1] \to [0,1]$ with $\mathrm{supp}(\phi) \subset [1/4, 3/4]$. All of the elements of $\Omega_{\mathbb{D}}$ we consider act as the identity on $\{0\} \cup \partial\mathbb{D}$, and hence we may remove any $\hat{x}_j \in \{0\} \cup \partial\mathbb{D}$ from the evaluation set $\Lambda_{\mathrm{Cl}(\mathbb{D})}$ without loss of generality. We construct F from a sequence of functions $\{F_k\}_{k=1}^{\infty} \subset \Omega_{\mathbb{D}}$ that are defined inductively as follows.

Base case: For $k = 1$, we begin by defining the perturbed rotation

$$Z_1(r, \theta) = (r, \theta + \pi + \phi(r)).$$

A simple argument using Jacobians shows that Z_1 is measure-preserving. It is also smooth and invertible. Let r_0 be any point in $[0,1]$ with $\phi(r_0)/\pi \notin \mathbb{Q}$ and $f_n : [0,1] \to [0,1]$ be a non-vanishing continuous function supported in $I_n = [\max\{r_0 - 1/n, 0\}, \min\{r_0 + 1/n, 1\}]$. For $j \in \mathbb{Z}$, set $f_{n,j}(r,\theta) = c_n f_n(r) e^{ij\theta}$, where c_n is a normalization constant so that $\|f_{n,j}\|_{L^2} = 1$. Then

$$\left\| \mathcal{K}_{Z_1} f_{n,j} - e^{ij(\pi + \phi(r_0))} f_{n,j} \right\|_{L^2}^2 = |c_n|^2 \int_0^1 \int_0^{2\pi} f_n(r)^2 \left| e^{ij\phi(r)} - e^{ij\phi(r_0)} \right|^2 r \,\mathrm{d}\theta\,\mathrm{d}r \leq \sup_{r \in I_n} \left| e^{ij\phi(r)} - e^{ij\phi(r_0)} \right|^2.$$

This bound converges to zero as $n \to \infty$ and hence $e^{ij\pi(1+\phi(r_0)/\pi)} \in \mathrm{Sp}(\mathcal{K}_{Z_1})$. Since $j \in \mathbb{Z}$ was arbitrary and $\phi(r_0)/\pi \notin \mathbb{Q}$, $\mathrm{Sp}(\mathcal{K}_{Z_1}) = \mathbb{T}$.

Let $\tilde{Z}_1 = \{f_{j,n}(Z_1) : j, n \in \mathbb{N}\}$ be a set of Δ_1-information for Z_1 such that all of the points $f_{j,n}(Z_1)$ are distinct, $\tilde{Z}_1 \cap \{\hat{x}_j : j \in \mathbb{N}\} = \emptyset$, and $\|f_{j,n}(Z_1) - Z_1(\hat{x}_j)\| \leq 2^{-(n+1)}$. The assumed convergence in Equation (9) implies that there exists $n_1 \in \mathbb{N}$ such that

$$\mathbb{P}\left(\mathrm{dist}(i, \Gamma_{n_1}(\tilde{Z}_1)) \leq 1\right) \geq \frac{1}{2}\left(\epsilon + \frac{1}{2}\right) = \frac{\epsilon}{2} + \frac{1}{4} > 1/2.$$

Let E_1 be the event $\mathrm{dist}(i, \Gamma_{n_1}(\tilde{Z}_1)) \leq 1$. Let E_2 be the event that $\Gamma_{n_1}(\tilde{Z}_1) \neq \mathrm{NH}$. Note that $E_1 \subset E_2$. Given $\eta > 0$, we may apply the covering lemma (Lemma 1.20) to deduce the existence of a finite subset $S \subset \Lambda_{\mathrm{Cl}(\mathbb{D})}^{\mathbb{P}}$ such that

$$\mathbb{P}\left(\{a \in \mathcal{C} : \Lambda_{\Gamma_{n_1}}(\tilde{Z}_1, a) \subset S\} \cap E_2\right) \geq \mathbb{P}(E_2) - \eta.$$

It follows that

$$\mathbb{P}(\{\Lambda_{\Gamma_{n_1}}(\tilde{Z}_1,a)\subset S\}\cap E_1)=\mathbb{P}(E_1)-\mathbb{P}(E_1\backslash\{\Lambda_{\Gamma_{n_1}}(\tilde{Z}_1,a)\subset S\})\geq\mathbb{P}(E_1)-\mathbb{P}(E_2\backslash\{\Lambda_{\Gamma_{n_1}}(\tilde{Z}_1,a)\subset S\})\geq\mathbb{P}(E_1)-\eta.$$

We choose η sufficiently small so that $\mathbb{P}(E_1)-\eta\geq\epsilon/4+3/8$. Hence, there exists a finite index set $\mathcal{I}_1\subset\mathbb{N}^2$ so that

$$\mathbb{P}\left(\mathrm{dist}(i,\Gamma_{n_1}(\tilde{Z}_1))\leq 1\text{ and }C_1(\tilde{Z}_1)\right)\geq\frac{\epsilon}{4}+\frac{3}{8}>1/2,$$

where $C_1(\tilde{F})$ is the event that Γ_{n_1} only samples from $\{f_{j,n}(F):(j,n)\in\mathcal{I}_1\}\subset\tilde{F}$ and outputs $\Gamma_{n_1}(\tilde{F})\neq\mathrm{NH}$. Let J_1 be the set of j for which there exists n with $(j,n)\in\mathcal{I}_1$ and for each such j, let $p_1(j)$ be the maximal such n. Define the sets

$$X_1=\{\hat{x}_j:j\in J_1\}\quad\text{and}\quad Y_1=\{f_{j,p_1(j)}(Z_1):j\in J_1\}.$$

Our assumptions on $\tilde{Z}_1$ imply that X_1 and Y_1 are sets of distinct points with $X_1\cap Y_1=\emptyset$.

Let $\mathcal{A}_1=\{R_1\leq r\leq r_1\}$ be a closed annulus in $\mathbb{D}\backslash\{0\}$, whose interior contains $X_1\cup\{f_{j,n}(Z_1):(j,n)\in\mathcal{I}_1\}$. Using Lemma 2.7, there is a measure-preserving homeomorphism H_1 that acts as the identity on $\mathrm{Cl}(\mathbb{D})\backslash\mathcal{A}_1$ such that $[H_1^{-1}\circ F_0\circ H_1](\hat{x}_j)=f_{j,p_1(j)}(Z_1)$ for $j\in J_1$. We define $F_1=H_1^{-1}\circ F_0\circ H_1$ so that $F_1(\hat{x}_j)=f_{j,p_1(j)}(Z_1)$. If $(j,n)\in\mathcal{I}_1$, then

$$\begin{aligned}\|f_{j,n}(Z_1)-F_1(\hat{x}_j)\|&\leq\|f_{j,n}(Z_1)-Z_1(\hat{x}_j)\|+\|Z_1(\hat{x}_j)-F_1(\hat{x}_j)\|\\&\leq 2^{-(n+1)}+2^{-(p_1(j)+1)}\leq 2^{-n}.\end{aligned}$$

In particular, we may extend $\{f_{j,n}(Z_1):(j,n)\in\mathcal{I}_1\}$ to admissible Δ_1-information for F_1, $\tilde{F}_1$, by setting $f_{j,n}(F_1)=F_1(\hat{x}_j)$ for $(j,n)\notin\mathcal{I}_1$. By consistency of probabilistic general algorithms,

$$\mathbb{P}\left(\mathrm{dist}(i,\Gamma_{n_1}(\tilde{F}_1))\leq 1\text{ and }C_1(\tilde{F}_1)\right)=\mathbb{P}\left(\mathrm{dist}(i,\Gamma_{n_1}(\tilde{Z}_1))\leq 1\text{ and }C_1(\tilde{Z}_1)\right)\geq\frac{\epsilon}{4}+\frac{3}{8}>1/2.$$

This completes the base case.

Inductive step: For the inductive step, suppose that $F_k\in\Omega_{\mathbb{D}}$, $\tilde{F}_k=\{f_{j,n}(F_k):j,n\in\mathbb{N}\}$, $n_k\in\mathbb{N}$ and $R_k\in(0,1)$ have been defined. To define the next function F_{k+1} and set of Δ_1-information $\tilde{F}_{k+1}$, we first choose a positive $r_{k+1}<\min\{(k+1)^{-1},R_k\}$ and define the following function in $\Omega_{\mathbb{D}}$:

$$Z_{k+1}(r,\theta)=\begin{cases}(r,\theta+\pi+\phi(r/r_{k+1})), & \text{if } r\leq r_{k+1},\\ F_k(r,\theta), & \text{otherwise.}\end{cases}$$

The argument for determining $\mathrm{Sp}(\mathcal{K}_{Z_1})$ extends to show that $\mathrm{Sp}(\mathcal{K}_{Z_{k+1}})=\mathbb{T}$.

Let $\tilde{Z}_{k+1}=\{f_{j,n}(Z_{k+1}):j,n\in\mathbb{N}\}$ be a set of Δ_1-information for Z_{k+1} with the following properties. If $|\hat{x}_j|\geq r_{k+1}$, then $f_{j,n}(Z_{k+1})=f_{j,n}(F_k)$. If $|\hat{x}_j|<r_{k+1}$, then $|f_{j,n}(Z_{k+1})|<r_{k+1}$, $\|f_{j,n}(Z_{k+1})-Z_{k+1}(\hat{x}_j)\|\leq 2^{-(n+1)}$, and the points $\{f_{j,n}(Z_{k+1}):|\hat{x}_j|<r_{k+1}\}$ are distinct with $\{f_{j,n}(Z_{k+1}):|\hat{x}_j|<r_{k+1}\}\cap\{\hat{x}_j:j\in\mathbb{N}\}=\emptyset$. Arguing as above, there exists $n_{k+1}\in\mathbb{N}$ with $n_{k+1}>n_k$, a finite set $\mathcal{I}_{k+1}\subset\mathbb{N}^2$ such that the following two conditions hold. First, letting J_{k+1} be the set of j for which there exists n with $(j,n)\in\mathcal{I}_{k+1}$, it holds that $|\hat{x}_j|<r_{k+1}$ whenever $j\in J_{k+1}$. Second,

$$\mathbb{P}\left(\mathrm{dist}(i,\Gamma_{n_{k+1}}(\tilde{Z}_{k+1}))\leq 1\text{ and }C_{k+1}(\tilde{Z}_{k+1})\right)\geq\frac{\epsilon}{4}+\frac{3}{8}>1/2,$$

where $C_{k+1}(\tilde{F})$ is the event that $\Gamma_{n_{k+1}}$ only samples from $\{f_{j,n}(F):(j,n)\in\mathcal{I}_{k+1}\}\cup\{f_{j,n}(F):|\hat{x}_j|\geq r_{k+1}\}\subset\tilde{F}$ and outputs $\Gamma_{n_{k+1}}(\tilde{F})\neq\mathrm{NH}$. For each $j\in J_{k+1}$, let $p_{k+1}(j)$ be the maximal n such that $(j,n)\in\mathcal{I}_{k+1}$. We define the two sets

$$X_{k+1}=\{\hat{x}_j:j\in J_{k+1}\}\quad\text{and}\quad Y_{k+1}=\{f_{j,p_{k+1}(j)}(Z_{k+1}):j\in J_{k+1}\}.$$

Similar to the base case, our assumptions on $\tilde{Z}_{k+1}$ imply that X_{k+1} and Y_{k+1} are sets of distinct points with $X_{k+1}\cap Y_{k+1}=\emptyset$.

Let $\mathcal{A}_{k+1}=\{R_{k+1}\leq r\leq r_{k+1}\}$ for some $0<R_{k+1}<r_{k+1}$ be a closed annulus in $\mathbb{D}\backslash\{0\}$, whose interior contains $X_{k+1}\cup\{f_{j,n}(Z_{k+1}):(j,n)\in\mathcal{I}_{k+1}\}$. Using Lemma 2.7, there is a measure-preserving homeomorphism H_{k+1} that acts as the identity on $\mathrm{Cl}(\mathbb{D})\backslash\mathcal{A}_{k+1}$ such that $[H_{k+1}^{-1}\circ F_0\circ H_{k+1}](\hat{x}_j)=f_{j,p_{k+1}(j)}(Z_{k+1})$ for $j\in J_{k+1}$. We then define

$$F_{k+1}(r,\theta)=\begin{cases}[H_{k+1}^{-1}\circ F_0\circ H_{k+1}](r,\theta), & \text{if } r\leq r_{k+1},\\ F_k(r,\theta), & \text{otherwise,}\end{cases}$$

so that $F_{k+1}(\hat{x}_j)=f_{j,p_{k+1}(j)}(Z_{k+1})$ for $j\in J_{k+1}$. If $(j,n)\in\mathcal{I}_{k+1}$, then

$$\|f_{j,n}(Z_{k+1})-F_{k+1}(\hat{x}_j)\|\leq\|f_{j,n}(Z_{k+1})-Z_{k+1}(\hat{x}_j)\|+\|Z_{k+1}(\hat{x}_j)-F_{k+1}(\hat{x}_j)\|\leq 2^{-(n+1)}+2^{-(p_{k+1}(j)+1)}\leq 2^{-n}.$$

In particular, we may extend $\{f_{j,n}(Z_{k+1}) : (j,n) \in \mathcal{I}_{k+1}\} \cup \{f_{j,n}(F_k) : |\hat{x}_j| \geq r_{k+1}\}$ to admissible Δ_1-information for F_{k+1}, $\tilde{F}_{k+1}$, by setting $f_{j,n}(F_{k+1}) = F_{k+1}(\hat{x}_j)$ if $|\hat{x}_j| < r_{k+1}$ and $(j,n) \notin \mathcal{I}_{k+1}$. By consistency of probabilistic general algorithms,

$$\mathbb{P}\left(\mathrm{dist}(i, \Gamma_{n_{k+1}}(\tilde{F}_{k+1})) \leq 1 \text{ and } C_{k+1}(\tilde{F}_{k+1})\right) = \mathbb{P}\left(\mathrm{dist}(i, \Gamma_{n_{k+1}}(\tilde{Z}_{k+1})) \leq 1 \text{ and } C_{k+1}(\tilde{Z}_{k+1})\right) \geq \frac{\epsilon}{4} + \frac{3}{8} > 1/2.$$

This completes the inductive step.

Limit argument: We now let

$$F = \lim_{k\to\infty} F_k, \qquad H = \lim_{k\to\infty} H_k \circ H_{k-1} \circ \cdots \circ H_1,$$

and define the Δ_1-information $\tilde{F}$ by $f_{j,n}(F) = f_{j,n}(F_k)$ if $|\hat{x}_j| \geq r_{k+1}$ for $k = 1, 2, \ldots$. Since $f_{j,n}(F_{k+1}) = f_{j,n}(F_k)$ if $|\hat{x}_j| \geq r_{k+1}$ and $F_{k+1}(x) = F_k(x)$ for $|x| \geq r_{k+1}$, $\tilde{F}$ is well-defined and admissible. By consistency of probabilistic general algorithms, for any $k \in \mathbb{N}$,

$$\mathbb{P}\left(\mathrm{dist}(i, \Gamma_{n_k}(\tilde{F})) \leq 1 \text{ and } C_k(\tilde{F})\right) = \mathbb{P}\left(\mathrm{dist}(i, \Gamma_{n_k}(\tilde{Z}_k)) \leq 1 \text{ and } C_k(\tilde{Z}_k)\right) > 1/2.$$

Let T_k be the random variable $\mathrm{dist}(i, \Gamma_{n_k}(\tilde{F}))$, then

$$\mathbb{P}(T_k \leq 1) \geq \mathbb{P}\left(\mathrm{dist}(i, \Gamma_{n_k}(\tilde{F})) \leq 1 \text{ and } C_k(\tilde{F})\right) > 1/2 \quad \forall k \in \mathbb{N}.$$

Let A_m be the event $\cap_{k=m}^{\infty}\{T_k > 1 \text{ and } \Gamma_{n_k}(\tilde{F}) \neq \mathrm{NH}\}$, and note that

$$\mathbb{P}(A_k) \leq 1 - \mathbb{P}(T_k \leq 1) < 1/2. \tag{10}$$

The function H is a measure-preserving homeomorphism and $F = H^{-1} \circ F_0 \circ H$. Hence, $\mathcal{K}_H$ is unitary and $\mathcal{K}_F = \mathcal{K}_H^* \mathcal{K}_{F_0} \mathcal{K}_H$ so that $\mathrm{Sp}(\mathcal{K}_F) = \mathrm{Sp}(\mathcal{K}_{F_0}) = \{\pm 1\}$. In particular, $\mathrm{dist}(i, \mathrm{Sp}(\mathcal{K}_F)) > 1$, so by our initial assumption in Equation (9),

$$\mathbb{P}\left(\cup_{m=1}^{\infty} A_m\right) \geq \mathbb{P}\left(\lim_{n\to\infty} \Gamma_n(\tilde{F}) = \mathrm{Sp}(\mathcal{K}_F)\right) \geq \epsilon > 1/2.$$

Since $A_1 \subset A_2 \subset A_3 \subset \cdots$, there exists A_M with $\mathbb{P}(A_M) > 1/2$, which contradicts the bound in Equation (10).

Step 3: $\{\Xi_{\mathrm{Sp}}, \Omega_{\mathbb{D}}^L, \mathcal{M}_{\mathrm{H}}, \Lambda_{\mathrm{Cl}(\mathbb{D})}\} \notin \Delta_1^{\mathbb{P},1/2}$. We prove the slightly stronger result for exact input. Suppose for a contradiction that $\{\Gamma_n\}$ is an SPGA for $\{\Xi_{\mathrm{Sp}}, \Omega_{\mathbb{D}}^L, \mathcal{M}_{\mathrm{H}}, \Lambda_{\mathrm{Cl}(\mathbb{D})}\}$ such that

$$\inf_{F \in \Omega_{\mathbb{D}}^L} \mathbb{P}\left(d_{\mathrm{H}}(\Gamma_n(F), \mathrm{Sp}(\mathcal{K}_F)) \leq 2^{-n} \text{ for all } n \in \mathbb{N}\right) = \epsilon > 1/2. \tag{11}$$

Let F_1 be the identity map. We may argue as above to show that there exists a finite set $X \subset \mathcal{X} = \mathrm{Cl}(\mathbb{D})$ such that

$$\mathbb{P}\left(d_{\mathrm{H}}(\Gamma_1(F_1), \{1\}) \leq 2^{-1} \text{ and } C(F_1)\right) > 1/2,$$

where $C(F)$ is the event that Γ_1 samples F within the fixed finite set of points X to output $\Gamma_1(F)$. As before, let $\phi : [0,1] \to [0,1]$ be a smooth surjective bump function with $\mathrm{supp}(\phi) \subset [1/4, 3/4]$. There exists $r_0 \in [0,1]$ and $\delta_0 > 0$ such that with

$$F_2(r, \theta) = (r, \theta + \delta_0 \phi(r/r_0)),$$

we have $F_2 \in \Omega_{\mathbb{D}}^L$, $F_1 = F_2$ on X, and hence

$$\mathbb{P}\left(d_{\mathrm{H}}(\Gamma_1(F_2), \{1\}) \leq 2^{-1} \text{ and } C(F_2)\right) = \mathbb{P}\left(d_{\mathrm{H}}(\Gamma_1(F_1), \{1\}) \leq 2^{-1} \text{ and } C(F_1)\right) > 1/2.$$

Given $\delta > 0$, we may also assume that $\mathbb{P}(C(F_1)) = \mathbb{P}(C(F_2)) \geq 1 - \delta$.

We run two independent instances of Γ_1 for the two inputs F_1 and F_2. Let $T_1 = \Gamma_1(F_1)$ and $T_2 = \Gamma_1(F_2)$ be the corresponding outputs, which are random variables. Let B_j be the event $d_{\mathrm{H}}(\Gamma_1(F_j), \mathrm{Sp}(\mathcal{K}_{F_j})) \leq 2^{-1}$ for $j = 1, 2$, and let $E = C(F_1) \cap C(F_2)$. If E occurs, then the laws of T_1 and T_2 are the same. Moreover, $\mathrm{Sp}(\mathcal{K}_{F_1}) = \{1\}$, whereas $\mathrm{Sp}(\mathcal{K}_{F_2}) = \mathbb{T}$. Hence,

$$\mathbb{P}(B_1 \cap E) + \mathbb{P}(B_2 \cap E) = \mathbb{P}(\{d_{\mathrm{H}}(T_1, \{1\}) \leq 2^{-1}\} \cap E) + \mathbb{P}(\{d_{\mathrm{H}}(T_1, \mathbb{T}) \leq 2^{-1}\} \cap E).$$

Since $d_{\mathrm{H}}(\{1\}, \mathbb{T}) > 1$, it follows from the triangle inequality that

$$\mathbb{P}(\{d_{\mathrm{H}}(T_1, \{1\}) \leq 2^{-1}\} \cap E) + \mathbb{P}(\{d_{\mathrm{H}}(T_1, \mathbb{T}) \leq 2^{-1}\} \cap E) \leq 1.$$

Note that $\mathbb{P}(E) \geq 1 - 2\delta$. Upon combining with the assumed convergence in Equation (11), we see that

$$1 < 2\epsilon \leq \mathbb{P}(B_1) + \mathbb{P}(B_2) \leq \mathbb{P}(B_1 \cap E) + \mathbb{P}(B_2 \cap E) + 2\mathbb{P}(E^c) \leq 1 + 4\delta.$$

This is a contradiction for sufficiently small δ. □

Remark 2.8. We can alter the argument in step 2 of the above proof to smooth functions F on the punctured disk $\mathrm{Cl}(\mathbb{D}) \backslash \{0\}$. ■

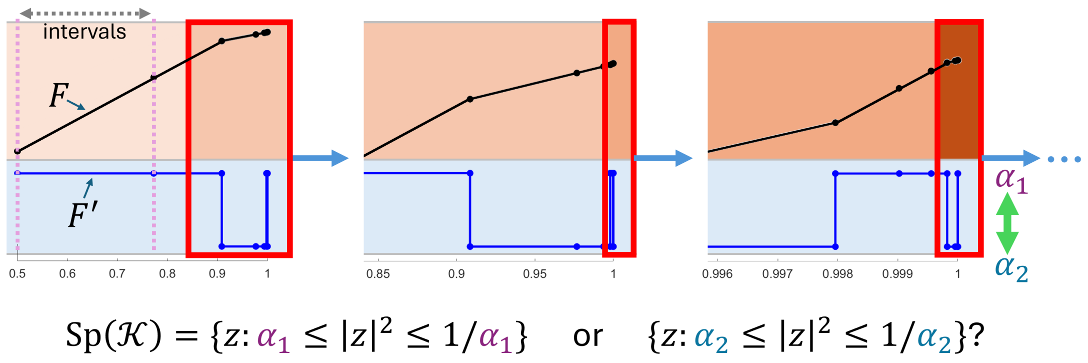

Supplementary Figure 1: The idea of the proof of the lower bound in Theorem 2.9. The plots show successive zoomed-in views of F as we move from left to right. We consider (smoothed) interval exchange maps, whose derivatives switch an infinite number of times as we approach the interval endpoints, causing the approximated spectrum to alternate between different annuli.

2.3 Maps on the unit interval

We now complement Theorem 2.6 and show that, even for smooth, invertible systems, where we can bound the variability of F, the spectrum cannot be computed in one limit if we drop the measure-preserving assumption from Theorem 2.3. We consider $\mathcal{X} = [0,1]$ equipped with the Euclidean metric and Lebesgue measure.

Let $c = \{c_n\}_{n=0}^{\infty}$ be a strictly increasing sequence in $[1/2, 1)$ with $c_0 = 1/2$ and $\lim_{n\to\infty} c_n = 1$. We set $c_{-n} = 1 - c_n$ for $n \in \mathbb{N}$ and define the intervals

$$I_n = [c_n, c_{n+1}), \quad n \in \mathbb{Z}.$$

We consider a continuous bijection $F : [0,1] \to [0,1]$ with $F(0) = 0$ and $F(1) = 1$ such that

$$F(c_n) = c_{n+1}, \quad F(I_n) = I_{n+1}.$$

In other words, F acts as a bijection between I_n and I_{n+1}. To study the spectrum of $\mathcal{K}_F$, we define the ratios

$$a_n = \frac{|I_{n+1}|}{|I_n|}, \quad n \in \mathbb{Z}.$$

We assume that F and F^{-1} are smooth on each subinterval I_n and for $\delta > 0$, we define

$$\Omega^{\delta}_{[0,1]} = \left\{F : [0,1] \to [0,1] \text{ such that the above holds for a sequence } c = \{c_n\} \text{ with } \max\{\|F'\|_{L^\infty}, \|(F^{-1})'\|_{L^\infty}\} \le 1+\delta\right\}.$$

(The 1 in the $1+\delta$ here comes from the fact that F is a bijection on the whole interval $[0,1]$.) Note that if the ratios a_n are bounded and bounded below away from zero, then the piecewise affine function F_c (uniquely) defined by acting as an affine function on each I_n is a particular case for which

$$\max\{\|F_c'\|_{L^\infty}, \|(F_c^{-1})'\|_{L^\infty}\} \le \max\left\{\sup_{n\in\mathbb{N}} a_n, \sup_{n\in\mathbb{N}} a_n^{-1}\right\}.$$

The map F_c is an example of an affine interval exchange map [43], which are well-studied in dynamical systems theory.

Theorem 2.9. *Given the above setup and $\delta > 0$, we have the following classifications for the approximate point spectrum*

$$\Delta_2^{\mathbb{P},1/2} \not\ni \{\Xi_{\text{Sp}_{\text{ap}}}, \Omega^{\delta}_{[0,1]}, \mathcal{M}_{\text{H}}, \Lambda_{[0,1]}\} \in \Pi_2.$$

The same classifications hold when restricting to smooth functions in $\Omega^{\delta}_{[0,1]}$, or to piecewise affine functions in $\Omega^{\delta}_{[0,1]}$.

Remark 2.10. Since $\Omega^{\delta}_{[0,1]} \subset \Omega^{\alpha}_{\mathcal{X}}$ for $\alpha(x) = (1+\delta)x$, Theorem 2.3 and Theorem 2.9 demonstrate that computing Sp_{ap} is strictly harder than computing $\text{Sp}_{\text{ap},\epsilon}$ for this class of Koopman operators. ■

We need the following technical lemma to prove Theorem 2.9 and the idea is summarized in Supplementary Figure 1. The first part of this lemma states that any $F \in \Omega^{\delta}_{[0,1]}$ induces a bounded Koopman operator $\mathcal{K}_F$ on $L^2([0,1])$. The second part of the lemma gives conditions for the spectrum to be contained in (or equal to) certain annuli.

Lemma 2.11. *If $F \in \Omega^{\delta}_{[0,1]}$, then $\|\mathcal{K}_F\| \le \sqrt{1+\delta}$. Furthermore, suppose that $\lim_{n\to\infty} a_n = \alpha \in (0,1]$ and that*

$$(1-\delta_F)a_n \le \frac{\mathrm{d}F}{\mathrm{d}x}(x) \le (1+\delta_F)a_n \qquad \textit{and} \quad (1-\delta_F)/a_{n-1} \le \frac{\mathrm{d}F^{-1}}{\mathrm{d}x}(x) \le (1+\delta_F)/a_{n-1} \quad \forall x \in I_n$$

for some $\delta_F \ge 0$. Then

$$\mathrm{Sp}(\mathcal{K}_F) \subset \left\{z \in \mathbb{C} : \sqrt{\alpha/(1+\delta_F)} \le |z| \le \sqrt{(1+\delta_F)/\alpha}\right\}.$$

If $\alpha < 1$, then

$$\left\{z \in \mathbb{C} : \sqrt{\alpha} \le |z| \le 1/\sqrt{\alpha}\right\} \subset \mathrm{Sp}_{\mathrm{ap}}(\mathcal{K}_F).$$

and hence $\mathrm{Sp}(\mathcal{K}_{F_c}) = \mathrm{Sp}_{\mathrm{ap}}(\mathcal{K}_{F_c}) = \{z \in \mathbb{C} : \sqrt{\alpha} \le |z| \le 1/\sqrt{\alpha}\}$.

Proof. For ease of notation, let $b_n = \sup_{t\in I_n} |(F^{-1})'(t)|$. If $g \in L^2([0,1])$ with $\|g\| = 1$, then

$$\|\mathcal{K}g\|^2 = \sum_{n\in\mathbb{Z}} \int_{I_n} |g(F(x))|^2 \,\mathrm{d}x \le \sum_{n\in\mathbb{Z}} \int_{I_{n+1}} |g(x)|^2 b_{n+1} \,\mathrm{d}x \le (1+\delta) \sum_{n\in\mathbb{Z}} \int_{I_{n+1}} |g(x)|^2 \,\mathrm{d}x = (1+\delta)\|g\|^2.$$

This bound proves the first part of the lemma, i.e., that $\|\mathcal{K}_F\| \le \sqrt{1+\delta}$.

Suppose in addition that $\lim_{n\to\infty} a_n = \alpha \in (0,1]$ and that

$$(1-\delta_F)a_n \le \frac{\mathrm{d}F}{\mathrm{d}x}(x) \le (1+\delta_F)a_n \qquad \text{and} \quad (1-\delta_F)/a_{n-1} \le \frac{\mathrm{d}F^{-1}}{\mathrm{d}x}(x) \le (1+\delta_F)/a_{n-1} \quad \forall x \in I_n$$

for some $\delta_F \ge 0$. We may apply the above argument $l \in \mathbb{N}$ times to see that

$$\|\mathcal{K}^l g\|^{1/l} \le \left[\sum_{n\in\mathbb{Z}} \int_{I_{n+l}} |g(x)|^2 \,\mathrm{d}x \prod_{j=1}^{l} b_{n+j}\right]^{1/(2l)} \le \sqrt{\sup_{n\in\mathbb{Z}} \prod_{j=1}^{l} b_{n+j}^{1/l}} \le \sqrt{\sup_{n\in\mathbb{Z}} \frac{1}{l}\sum_{j=1}^{l} b_{n+j}},$$

where we have used the generalized AM-GM inequality for the last inequality. For any $L \in \mathbb{N}$,

$$\limsup_{l\to\infty} \sup_{n\in\mathbb{Z}} \frac{1}{l}\sum_{j=1}^{l} b_{n+j} \le \limsup_{l\to\infty} \frac{1}{l}\sum_{j=1}^{l} \sup_{|n|>L} b_n = \sup_{|n|>L} b_n.$$

Taking $L \to \infty$, we have

$$\limsup_{l\to\infty} \sup_{n\in\mathbb{Z}} \frac{1}{l}\sum_{j=1}^{l} b_{n+j} \le \limsup_{|l|\to\infty} b_l \le (1+\delta_F)\max\left\{\lim_{n\to-\infty} 1/a_n, \lim_{n\to+\infty} 1/a_n\right\} \le \frac{(1+\delta_F)}{\alpha}.$$

It follows that $\lim_{l\to\infty} \|\mathcal{K}^l\|^{1/l} \le \sqrt{(1+\delta_F)/\alpha}$. Gelfand's formula for the spectral radius implies that if $z \in \mathrm{Sp}(\mathcal{K}_F)$, then $|z| \le \sqrt{(1+\delta_F)/\alpha}$. We can argue in the same manner, using the fact that $\lim_{n\to-\infty} a_n = 1/\alpha \in [1,\infty)$, to see that $\lim_{l\to\infty} \|\mathcal{K}^{-l}\|^{1/l} \le \sqrt{(1+\delta_F)/\alpha}$ and hence that

$$\inf_{z\in\mathrm{Sp}(\mathcal{K}_F)} |z| = \frac{1}{\sup_{z\in\mathrm{Sp}(\mathcal{K}_{F^{-1}})} |z|} \ge \frac{1}{\sqrt{(1+\delta_F)/\alpha}} = \sqrt{\alpha/(1+\delta_F)}.$$

It follows that

$$\mathrm{Sp}(\mathcal{K}_F) \subset \left\{z \in \mathbb{C} : \sqrt{\alpha/(1+\delta_F)} \le |z| \le \sqrt{(1+\delta_F)/\alpha}\right\}.$$

To prove the inclusion for the approximate point spectrum, suppose that $\alpha < 1$, let $z \in \mathbb{C}$ with $\sqrt{\alpha} < |z| < 1/\sqrt{\alpha}$ and set

$$g = \sum_{n\in\mathbb{Z}} z^n \chi_{I_n}.$$

Given $\eta > 0$ with $\sqrt{\alpha} < \sqrt{\eta} < |z| < 1/\sqrt{\eta} < 1/\sqrt{\alpha}$, let $\rho = \max\{|z|\sqrt{\eta}, \sqrt{\eta}/|z|\} < 1$. For sufficiently large n we have $a_n \le \eta$. It follows that there exists a constant $C > 0$ such that if $n \ge 0$, then

$$|z|^{2n}|I_n| = |I_0||z|^{2n} \prod_{j=0}^{n-1} a_j \le C(|z|\sqrt{\eta})^{2n} \le C\rho^{2n},$$

and if $n > 1$ then

$$|z|^{-2n}|I_{-n}| = |z|^{-2n}|I_{n-1}| = |I_0||z|^{-2n}\prod_{j=0}^{n-2} a_j \le C(\sqrt{\eta}/|z|)^{2n} \le C\rho^{2n}.$$

It follows that

$$\|g\|^2 = \sum_{n\in\mathbb{Z}} |z|^{2n}|I_n| \le 2C\sum_{n=0}^{\infty}\rho^{2n} < \infty.$$

Since F acts as a bijection between I_n and I_{n+1}, $F^{-1}(I_{n+1}) = I_n$ and hence $\mathcal{K}\chi_{I_n} = \chi_{I_{n-1}}$. It follows that $\mathcal{K}g = zg$ and hence z is an eigenvalue. Since z with $\sqrt{\alpha} < |z| < 1/\sqrt{\alpha}$ was arbitrary, the closed annulus $\{z \in \mathbb{C} : \sqrt{\alpha} \le |z| \le 1/\sqrt{\alpha}\}$ lies in $\mathrm{Sp}_{\mathrm{ap}}(\mathcal{K}_F)$. The final statement follows from the fact that we can take $\delta_{F_c} = 0$. $\square$

Proof of Theorem 2.9. The upper bound immediately follows from Theorem 2.3, so we only need to prove the lower bounds. We prove this for piecewise affine functions with exact input (which implies the result for Δ_1-information), and the proof is almost identical when restricting to smooth functions in $\Omega^{\delta}_{[0,1]}$.

Suppose, for a contradiction, that $\{\Gamma_n\}$ is an SPGA for $\{\Xi_{\mathrm{Sp_{ap}}}, \Omega^{\delta}_{[0,1]}, \mathcal{M}_{\mathrm{H}}, \Lambda_{[0,1]}\}$ with

$$\inf_{F\in\Omega^{\delta}_{[0,1]}} \mathbb{P}\left(\lim_{n\to\infty}\Gamma_n(F) = \mathrm{Sp}_{\mathrm{ap}}(\mathcal{K}_F)\right) = \epsilon > 1/2. \tag{12}$$

We consider $F = F_c$ defined by acting as an affine function on each subinterval I_n, and choose the sequence $c = \{c_n\}_{n=0}^{\infty}$ to contradict Equation (12). The probabilistic part of the proof is similar to step 2 of the proof of Theorem 2.6. Let $r_1 \in (0,1)$ be such that $r_1^{-2} < 1+\delta$. Set

$$r_2 = (1-r_1)^2 + r_1 > r_1,$$

so that $(1-r_2) = r_1(1-r_1)$. We may choose $\tau > 0$ such that

$$(1+2\tau)\sqrt{r_1} < (1-2\tau)\sqrt{r_2}.$$

The numbers r_1, r_2, and τ are fixed throughout the proof. We use a superscript (j) to denote choices of the sequences $\{c_n\}$ and corresponding objects such as the intervals $I_n^{(j)}$.

We begin with the sequence $c^{(1)} = \{c_n^{(1)}\}_{n=0}^{\infty}$ chosen so that

$$|I_n^{(1)}| = \beta^{(1)} r_1^n, \quad n = 0, 1, 2, \ldots.$$

Here, the constant $\beta^{(1)}$ is chosen such that

$$\sum_{n=-\infty}^{\infty}|I_n^{(1)}| = 2\sum_{n=0}^{\infty}|I_n^{(1)}| = \frac{2\beta^{(1)}}{1-r_1} = 1.$$

Since $\max\{r_1, r_1^{-1}\} = r_1^{-1} < 1+\delta$, $F_{c^{(1)}} \in \Omega^{\delta}_{[0,1]}$. The intervals $I_n^{(1)}$ have constant ratio $a_n^{(1)} = r_1$ and hence Lemma 2.11 implies that

$$\mathrm{Sp}(\mathcal{K}_{F_{c^{(1)}}}) = \mathrm{Sp}_{\mathrm{ap}}(\mathcal{K}_{F_{c^{(1)}}}) = \left\{z \in \mathbb{C} : \sqrt{r_1} \le |z| \le 1/\sqrt{r_1}\right\}.$$

Applying the covering lemma (Lemma 1.20), it follows that there exists $n_1, N_1 \in \mathbb{N}$ such that

$$\mathbb{P}\left(\inf\{|z| : z \in \Gamma_{n_1}(F_{c^{(1)}})\} \le (1+\tau)\sqrt{r_1},\, \Lambda_{\Gamma_{n_1}}(F_{c^{(1)}}) \subset \cup_{j=-N_1}^{N_1-1} I_j^{(1)}\right) > 1/2,$$

where the notation $\Lambda_\Gamma(F)$ means that Γ samples F only within the set $\Lambda_\Gamma(F) \subset \mathcal{X}$ before producing its output. Moreover, we use the convention that $\inf\{|z| : z \in \Gamma_{n_1}(F_{c^{(1)}})\} \le (1+\tau)\sqrt{r_1}$ necessarily implies that $\Gamma_{n_1}(F_{c^{(1)}}) \neq \mathrm{NH}$.

Next, we define the sequence $c^{(2)}$ implicitly by

$$|I_n^{(2)}| = \begin{cases} |I_n^{(1)}|, & \text{if } 0 \le n \le N_1, \\ \beta^{(2)} r_2^{n-(N_1+1)}, & \text{if } n > N_1. \end{cases}$$

Here, the factor $\beta^{(2)}$ is chosen so that

$$\frac{\beta^{(2)}}{1-r_2} = \sum_{n=N_1+1}^{\infty}|I_n^{(2)}| = \sum_{n=N_1+1}^{\infty}|I_n^{(1)}| = |I_{N_1}^{(1)}|\sum_{n=1}^{\infty} r_1^n = |I_{N_1}^{(1)}|\frac{r_1}{1-r_1}.$$

In particular,

$$\frac{|I^{(2)}_{N_1+1}|}{|I^{(2)}_{N_1}|} = \frac{\beta^{(2)}}{|I^{(1)}_{N_1}|} = \frac{r_1(1-r_2)}{1-r_1} = r_1^2 \leq 1 < 1+\delta.$$

Similarly, the reciprocal of this ratio is $r_1^{-2} < 1+\delta$. Hence, $F_{c^{(2)}} \in \Omega^{\delta}_{[0,1]}$ and Lemma 2.11 implies that

$$\mathrm{Sp}(\mathcal{K}_{F_{c^{(2)}}}) = \mathrm{Sp}_{\mathrm{ap}}(\mathcal{K}_{F_{c^{(2)}}}) = \{z \in \mathbb{C} : \sqrt{r_2} \leq |z| \leq 1/\sqrt{r_2}\}.$$

Applying the covering lemma (Lemma 1.20), it follows that there exists $n_2, N_2 \in \mathbb{N}$ such that $n_2 > n_1$, $N_2 > N_1$, and

$$\mathbb{P}\left(\inf\{|z| : z \in \Gamma_{n_2}(F_{c^{(2)}})\} \geq (1-\tau)\sqrt{r_2},\, \Lambda_{\Gamma_{n_2}}(F_{c^{(2)}}) \subset \cup_{j=-N_2}^{N_2-1} I^{(2)}_j\right) > 1/2.$$

Again, we use the convention that $\inf\{|z| : z \in \Gamma_{n_2}(F_{c^{(2)}})\} \geq (1-\tau)\sqrt{r_2}$ necessarily implies that $\Gamma_{n_2}(F_{c^{(2)}}) \neq \mathrm{NH}$.

We continue this process inductively. Let $\hat{r}_j = r_2$ if j is even and $\hat{r}_j = r_1$ if j is odd. Suppose that the intervals $I^{(k-1)}_n$ have been defined for $n \in \mathbb{Z}$ and $n_{k-1}, N_{k-1} \in \mathbb{N}$ are such that

$$\mathbb{P}\left(\inf\{|z| : z \in \Gamma_{n_{k-1}}(F_{c^{(k-1)}})\} \begin{Bmatrix} \leq (1+\tau)\sqrt{r_1}, & \text{if } k \text{ is even} \\ \geq (1-\tau)\sqrt{r_2}, & \text{if } k \text{ is odd} \end{Bmatrix} \text{ and } \Lambda_{\Gamma_{n_{k-1}}}(F_{c^{(k-1)}}) \subset \cup_{j=-N_{k-1}}^{N_{k-1}-1} I^{(k-1)}_j\right) > 1/2.$$

We define the sequence $c^{(k)}$ implicitly by

$$|I^{(k)}_n| = \begin{cases} |I^{(k-1)}_n|, & \text{if } 0 \leq n \leq N_{k-1}, \\ \beta^{(k)} \hat{r}_k^{\,n-(N_{k-1}+1)}, & \text{if } n > N_{k-1}, \end{cases}$$

Here, the factor $\beta^{(k)}$ is chosen so that

$$\frac{\beta^{(k)}}{1-\hat{r}_k} = \sum_{n=N_{k-1}+1}^{\infty} |I^{(k)}_n| = \sum_{n=N_{k-1}+1}^{\infty} |I^{(k-1)}_n| = |I^{(k-1)}_{N_{k-1}}| \sum_{n=1}^{\infty} \hat{r}_{k-1}^n = |I^{(k-1)}_{N_{k-1}}| \frac{\hat{r}_{k-1}}{1-\hat{r}_{k-1}}.$$

In particular, a case by case analysis shows that

$$\frac{|I^{(k)}_{N_{k-1}+1}|}{|I^{(k)}_{N_{k-1}}|} = \frac{\beta^{(k)}}{|I^{(k-1)}_{N_{k-1}}|} = \frac{\hat{r}_{k-1}(1-\hat{r}_k)}{1-\hat{r}_{k-1}} \leq 1+\delta, \quad \frac{1-\hat{r}_{k-1}}{\hat{r}_{k-1}(1-\hat{r}_k)} \leq 1+\delta.$$

Hence, $F_{c^{(k)}} \in \Omega^{\delta}_{[0,1]}$. Using Lemma 2.11, we select $n_k, N_k \in \mathbb{N}$ so that

$$\mathbb{P}\left(\inf\{|z| : z \in \Gamma_{n_k}(F_{c^{(k)}})\} \begin{Bmatrix} \leq (1+\tau)\sqrt{r_1}, & \text{if } k+1 \text{ is even} \\ \geq (1-\tau)\sqrt{r_2}, & \text{if } k+1 \text{ is odd} \end{Bmatrix} \text{ and } \Lambda_{\Gamma_{n_k}}(F_{c^{(k)}}) \subset \cup_{j=-N_k}^{N_k-1} I^{(k)}_j\right) > 1/2. \tag{13}$$

and this completes the inductive step.

We can now define the sequence $c = \{c_n\}$ by taking the limit $c_n = \lim_{j\to\infty} c^{(j)}_n$. For any fixed n, $c^{(j)}_n$ is constant for large j and hence $F_c \in \Omega^{\delta}_{[0,1]}$. For each $k \in \mathbb{N}$, let B_k be the event

$$\inf\{|z| : z \in \Gamma_{n_k}(F_c)\} \begin{cases} \leq (1+\tau)\sqrt{r_1}, & \text{if } k+1 \text{ is even}, \\ \geq (1-\tau)\sqrt{r_2}, & \text{if } k+1 \text{ is odd}. \end{cases}$$

Due to Equation (13), and the consistency of probabilistic general algorithms (see the discussion in Example 1.16), $\mathbb{P}(B_k) > 1/2$ for all k. From the bound $(1+2\tau)\sqrt{r_1} < (1-2\tau)\sqrt{r_2}$, we see that either $\inf\{|z| : z \in \mathrm{Sp}_{\mathrm{ap}}(F_c)\} > (1+\tau)\sqrt{r_1}$ or $\inf\{|z| : z \in \mathrm{Sp}_{\mathrm{ap}}(F_c)\} < (1-\tau)\sqrt{r_2}$ (or both). Assume without loss of generality that the former holds and let $A_m = \cap_{k=m}^{\infty} B^c_{2k+1}$. Note that $\mathbb{P}(A_m) \leq 1 - \mathbb{P}(B_{2m+1}) < 1/2$. However, from the assumed lower bound of the probability of convergence in Equation (12),

$$\mathbb{P}\left(\cup_{m=1}^{\infty} A_m\right) \geq \mathbb{P}\left(\lim_{n\to\infty} \Gamma_n(F_c) = \mathrm{Sp}_{\mathrm{ap}}(\mathcal{K}_{F_c})\right) \geq \epsilon > 1/2.$$

Since $A_1 \subset A_2 \subset A_3 \subset \cdots$, there must exist some A_M with $\mathbb{P}(A_M) > 1/2$, the required contradiction. □

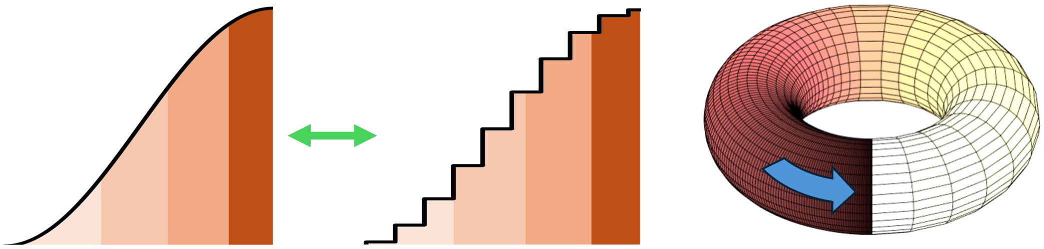

Supplementary Figure 2: The idea of the proof of Theorem 2.12's lower bound. We examine skew products on tori, where the spectral type depends on the existence of neighborhoods with constant cross-sections. The spectral change comes from a skew product constructed through iterations of locally piecewise constant and smooth approximations.

2.4 Computing the spectral type is impossible in one limit

We now consider the problem of computing the pure point spectrum of the Koopman operator and determining if there are any eigenvalues except 1. This last problem is important since one usually wishes to know if there is coherency in the sense of Equation (3) for $\lambda \neq 1$. Our results also show that one cannot determine whether the spectral measure away from $\lambda = 1$ is continuous. We consider the torus $\mathcal{X} = [0,2\pi]_{\mathrm{per}} \times [0,2\pi]_{\mathrm{per}} = [0,2\pi]_{\mathrm{per}}^2$, equipped with the standard, normalized, Lebesgue measure. We set

$$\Omega_p = \left\{F : [0,2\pi]_{\mathrm{per}}^2 \to [0,2\pi]_{\mathrm{per}}^2 \text{ s.t. } F \text{ is smooth, measure-preserving, invertible}, \mathrm{Lip}(F) \leq 2, \mathrm{Lip}(F^{-1}) \leq 2\right\}.$$

In other words, Ω_p includes both assumptions needed to compute spectra in one limit in Theorem 2.3. We consider two problems. The first is computing the pure point spectrum (closure of the set of eigenvalues), i.e., the problem function:

$$\Xi_p : \Omega_p \ni F \mapsto \mathrm{Sp}_{\mathrm{pp}}(\mathcal{K}_F) = \mathrm{Cl}\left(\{\lambda \in \mathbb{C} : \lambda \text{ is an eigenvalue of } \mathcal{K}_F\}\right) \in \mathcal{M}_{\mathrm{H}}.$$

The second is the following decision problem:

$$\Xi_p^{\mathrm{dec}} : \Omega_p \ni F \mapsto \left\{\begin{matrix} 0, & \text{if there are no eigenvalues except 1} \\ 1, & \text{otherwise} \end{matrix}\right\} \in \mathcal{M}_{\mathrm{dec}}.$$

The following theorem precisely classifies these two computational problems.

Theorem 2.12. *Given the above setup, we have the following classifications for non-trivial eigenvalues*

$$\Delta_2^{\mathbb{P},1/2} \not\ni \{\Xi_p, \Omega_p, \mathcal{M}_{\mathrm{H}}, \Lambda_{[0,2\pi]_{\mathrm{per}}^2}\} \in \Sigma_2, \qquad \Delta_2^{\mathbb{P},1/2} \not\ni \{\Xi_p^{\mathrm{dec}}, \Omega_p, \mathcal{M}_{\mathrm{dec}}, \Lambda_{[0,2\pi]_{\mathrm{per}}^2}\} \in \Sigma_2.$$

The following technical lemma is used in the proof of Theorem 2.12 and the idea is summarized in Supplementary Figure 2.

Lemma 2.13. *Let $F_f \in \Omega_p$ be the skew product map $F_f(x,y) = (x, f(x)+y)$, where $f : [0,2\pi]_{\mathrm{per}} \to [0,2\pi]_{\mathrm{per}}$ is a smooth function that is non-decreasing on $[0,2\pi)$ with $f(0) = 0$. Then:*

(a) If f is a bijection with $f'(x) > 0$ for all $x \in [0,2\pi)$, $\mathrm{Sp}_{\mathrm{pp}}(\mathcal{K}_{F_f}) = \{1\}$.

(b) If there exists an open interval $(a,b) \subset [0,2\pi)$ upon which $f(x) = c$ is constant, then $\{e^{ijc} : j \in \mathbb{Z}\} \subset \mathrm{Sp}_{\mathrm{pp}}(\mathcal{K}_{F_f})$.

Proof. For (a), suppose that f is a bijection with $f'(x) > 0$ for all $x \in [0,2\pi)$. Consider the smooth invertible map $\phi(x,y) = (f^{-1}(x), y)$. Then $(\phi^{-1} \circ F_f \circ \phi)(x,y) = (x, x+y)$ and $\mathcal{K}_{\phi^{-1}\circ F_f \circ \phi} = \mathcal{K}_\phi \mathcal{K}_{F_f}[\mathcal{K}_\phi]^{-1}$. Hence, $\mathcal{K}_{F_f}$ is similar to the Koopman operator of the skew product map $(x,y) \mapsto (x, x+y)$. Similar operators have the same eigenvalues, so the statement in (a) now follows. For (b), suppose that there exists an open interval $(a,b) \subset [0,2\pi)$ upon which $f(x) = c$ is constant. Let $g_j(x,y) = \chi_{(a,b)}(x)e^{ijy}$, where χ_S denotes the indicator function of a set S. Then $\mathcal{K}_{F_f} g_j = e^{ijc} g_j$ and the result follows. □

We also need the following discrete-time RAGE theorem [32, Proof of Theorem A.2]. The continuous-time RAGE theorem [1, Theorem 2.6], named after Ruelle [61], Amrein and Georgescu [2], and Enss [31], is a classical dynamical characterization of the continuous spectrum of self-adjoint operators, and is a valuable tool in the study of Schrödinger operators.

Theorem 2.14. *Let A be a unitary operator acting on a separable Hilbert space $\mathcal{H}$, and $\{\mathcal{P}_n\}_{n\in\mathbb{N}}$ a sequence of increasing finite-rank orthogonal projections such that $\mathcal{P}_n^*\mathcal{P}_n$ converges strongly to the identity. (We view $\mathcal{P}_n$ as an operator from $\mathcal{H}$ to its range, hence the need for $\mathcal{P}_n^*$.) Let $\mathcal{P}_{\rm c}$ and $\mathcal{P}_{\rm pp}$ denote the orthogonal projections onto $\mathcal{H}_{\rm c}$ (continuous part) and $\mathcal{H}_{\rm pp}$ (pure point part), respectively. Then for any $g \in \mathcal{H}$,*

$$\|\mathcal{P}_{\rm c}g\|^2 = \lim_{n\to\infty}\lim_{L\to\infty}\frac{1}{2L+1}\sum_{\ell=-L}^{L}\left\|(I-\mathcal{P}_n^*\mathcal{P}_n)A^\ell g\right\|^2, \qquad \|\mathcal{P}_{\rm pp}g\|^2 = \lim_{n\to\infty}\lim_{L\to\infty}\frac{1}{2L+1}\sum_{\ell=-L}^{L}\left\|\mathcal{P}_n A^\ell g\right\|^2.$$

Proof of Theorem 2.12. We first prove the upper bounds and then prove the lower bounds.

Step 1: Upper bounds. Let $\{e_j\}_{j=1}^\infty$ be an orthonormal basis of $L^2(\mathcal{X},\omega)$ constructed using a tensor product of Fourier bases. Using the arguments in Step 1 of the proof of Theorem 2.3 and those of [19], for any $z \notin \mathbb{T}$ and any $g \in L^2(\mathcal{X},\omega)$ that is a finite linear combination of the $\{e_j\}_{j=1}^\infty$, we can compute $(\mathcal{K}_F - zI)^{-1}g$ with error control using the given Δ_1-information. The proof follows the arguments in [19, Theorem 2.1]. It follows from the unitary version of [18, Theorem 5.1.1] that $\{\Xi_p, \Omega_p, \mathcal{M}_{\rm H}, \Lambda_{[0,2\pi]^2_{\rm per}}\} \in \Sigma_2$. To deal with $\Xi_p^{\rm dec}$, let $\mathcal{P}_n$ be the orthogonal projection onto ${\rm span}\{e_1,\ldots,e_n\}$. Let g be a finite linear combination of the $\{e_j\}_{j=1}^\infty$. For $n \in \mathbb{N}$ and $\ell \in \mathbb{Z}$, using the unitary version of [18, Theorem 4.3.3 & Remark 4.3.4] (which uses Theorem 2.14), we may compute $W_{n_2,n_1}(g)$ (see Supplementary Algorithm 6) such that

$$\lim_{n_1\to\infty} W_{n_2,n_1}(g) = W_{n_2}(g), \quad \lim_{n_2\to\infty} W_{n_2}(g) = \mu_g^{\rm (pp)}(\mathbb{T}\backslash\{1\}),$$

where $\mu_g^{\rm (pp)}$ is the pure point part of the spectral measure of $\mathcal{K}_F$ with respect to g and the convergence as $n_2 \to \infty$ is from below. Let $\{g_k\}_{k=1}^\infty$ be a set of such g that form a dense subset of $L^2(\mathcal{X},\omega)$. We then set

$$a_{n_2,n_1}(F) = \max_{1\le k\le n_2} W_{n_2,n_1}(g_k).$$

Define the two separated intervals $I_1 = [0, 1/4]$ and $I_2 = [1/2, \infty)$. As $n_1 \to \infty$, $a_{n_2,n_1}(F)$ converges to $a_{n_2}(F) = \max_{1\le k\le n_2} W_{n_2}(g_k)$ and hence cannot visit both I_1 and I_2 infinitely often. For a given n_1, we set $\Gamma_{n_2,n_1}(F) = 0$ if the largest $l = 1,\ldots,n_1$ with $a_{n_2,l} \in I_1 \cup I_2$ has $a_{n_2,l} \in I_1$. If no such l exists, or $a_{n_2,l} \in I_2$, we set $\Gamma_{n_2,n_1}(F) = 1$. If $\Xi_p^{\rm dec}(F) = 0$, then $\mu_{g_k}^{\rm (pp)}(\mathbb{T}\backslash\{1\}) = 0$ for all k and hence $a_{n_2}(F) = 0$ for all n_2. It follows that $\lim_{n_1\to\infty}\Gamma_{n_2,n_1}(F) = 0$. If $\Xi_p^{\rm dec}(F) = 1$, then there exists g_k with $\mu_{g_k}^{\rm (pp)}(\mathbb{T}\backslash\{1\}) > 1/2$ and hence $a_{n_2}(F) > 1/2$ for sufficiently large n_2. It follows that $\lim_{n_1\to\infty}\Gamma_{n_2,n_1}(F) = 1$ for sufficiently large n_2. Moreover, since $a_{n_2,n_1}(F)$ are increasing in n_2, $\Gamma_{n_2}(F)$ is increasing in n_2. It follows that $\{\Xi_p^{\rm dec}, \Omega_p, \mathcal{M}_{\rm dec}, \Lambda_{[0,2\pi]^2_{\rm per}}\} \in \Sigma_2$. The process is summarized in Supplementary Algorithm 7.

Step 2: Lower bounds. Throughout the proof, we write $(x, y) \in [0, 2\pi]^2_{\rm per}$ to denote the state. We prove the lower bound for the subclass of maps $F_q \in \Omega_p$ of the form $F_q(x, y) = (x, q(x) + y)$, where q has the properties outlined in Lemma 2.13 (smooth, non-decreasing on $[0, 2\pi)$ with $q(0) = 0$). This implies the lower bound for the full class Ω_p. Without loss of generality and with a slight abuse of notation, we may restrict our argument to an evaluation set that samples q at a dense set of points $\{\hat{x}_j\}_{j=1}^\infty \subset (0, 2\pi)$. Suppose, for a contradiction, that $\{\Gamma_n\}$ is an SPGA for $\{\Xi_p, \Omega_p, \mathcal{M}_{\rm H}, \Lambda_{[0,2\pi]^2_{\rm per}}\}$ with the property that

$$\inf_{F_q\in\Omega_p}\mathbb{P}\left(\lim_{n\to\infty}\Gamma_n(\tilde{q}) = {\rm Sp}_{\rm pp}(\mathcal{K}_{F_q})\right) = \epsilon > 1/2. \tag{14}$$

Here, $\tilde{q}$ represents inexact input for q. That is, an admissible $\tilde{q}$ is of the form $\{f_{j,n}(q) : j, n \in \mathbb{N}\}$, where $|f_{j,n}(q) - q(\hat{x}_j)| \le 2^{-n}$. We will construct a function q with inexact input $\tilde{q}$ so that the bound in Equation (14) cannot hold. The function q will be a pointwise limit of functions $q^{(k)}$. Throughout, $q^{(k)}$ will be assumed to satisfy the properties outlined in Lemma 2.13 and $F_{q^{(k)}} \in \Omega_p$, even if not explicitly stated.

Base case: We begin with a bijection $q^{(1)}$ such that $(q^{(1)})'(x) > 0$ for all $x \in [0, 2\pi)$. Case (a) of Lemma 2.13 implies that $\Xi_p(F_{q^{(1)}}) = \{1\}$. Let $\tilde{q}^{(1)}$ be an arbitrary set of Δ_1-information for $q^{(1)}$. Applying the covering lemma (Lemma 1.20), there exists $n_1 \in \mathbb{N}$, a finite set $\mathcal{I}_1 \subset \mathbb{N}^2$ and $r_1 \in (0, 2\pi)$ such that the following two conditions hold. First, letting J_1 be the set of j for which there exists n with $(j, n) \in \mathcal{I}_1$, it holds that $\hat{x}_j < r_1$ whenever $j \in J_1$. Second,

$$\mathbb{P}\left({\rm dist}(-1, \Gamma_{n_1}(\tilde{q}^{(1)})) \ge 1 \text{ and } C_1(\tilde{q}^{(1)})\right) > 1/2,$$

where $C_1(\tilde{q})$ is the event that Γ_{n_1} samples from $\{f_{j,n}(q) : (j, n) \in \mathcal{I}_1\}$ and outputs $\Gamma_{n_1}(\tilde{q}) \neq$ NH. This completes the base case.

Inductive step: Suppose $q^{(k)}$, $\tilde{q}^{(k)} = \{f_{j,n}(q^{(k)}) : j, n \in \mathbb{N}\}$, $n_k \in \mathbb{N}$ and $r_k \in (0, 2\pi)$ are defined. We consider two cases, depending on the parity of k.

If k is odd, we choose $q^{(k+1)}$ so that $q^{(k+1)}(x) = q^{(k)}(x)$ for $x \in (0, r_k]$ and $q^{(k+1)}$ is constant on an open interval containing $r_k/2 + \pi$, where it takes a value c_{k+1} with $c_{k+1}/\pi \notin \mathbb{Q}$. We define $\tilde{q}^{(k+1)}$, the Δ_1-information for $q^{(k+1)}$, as follows. If $\hat{x}_j \le r_k$, then $f_{j,n}(q^{(k+1)}) = f_{j,n}(q^{(k)})$, otherwise $f_{j,n}(q^{(k+1)}) = q^{(k+1)}(\hat{x}_j)$. Case (b) of Lemma 2.13 implies that

$\Xi_p(F_{q^{(k+1)}}) = \mathbb{T}$. Applying the covering lemma (Lemma 1.20), there exists $n_{k+1} \in \mathbb{N}$ with $n_{k+1} \geq n_k$, a finite set $\mathcal{I}_{k+1} \subset \mathbb{N}^2$ with $\mathcal{I}_k \subset \mathcal{I}_{k+1}$, and $r_{k+1} \in (r_k/2 + \pi, 2\pi)$ such that the following two conditions hold. First, letting J_{k+1} be the set of j for which there exists n with $(j,n) \in \mathcal{I}_{k+1}$, it holds that $\hat{x}_j < r_{k+1}$ whenever $j \in J_{k+1}$. Second,

$$\mathbb{P}\left(\mathrm{dist}(-1, \Gamma_{n_{k+1}}(\tilde{q}^{(k+1)})) \leq 1/2 \text{ and } C_{k+1}(\tilde{q}^{(k+1)})\right) > 1/2,$$

where $C_{k+1}(\tilde{q})$ is the event that $\Gamma_{n_{k+1}}$ samples from $\{f_{j,n}(q) : (j,n) \in \mathcal{I}_{k+1}\}$ and outputs $\Gamma_{n_{k+1}}(\tilde{q}) \neq \mathrm{NH}$.

If k is even, we can choose $q^{(k+1)}$ with $(q^{(k+1)})'(x) > 0$ for all $x \in [0, 2\pi)$ and Δ_1-information $\tilde{q}^{(k+1)} = \{f_{j,n}(q^{(k+1)}) : j,n \in \mathbb{N}\}$ so that

$$f_{j,n}(q^{(k+1)}) = f_{j,n}(q^{(k)}) \quad \forall (j,n) \in \mathcal{I}_k, \quad q^{(k+1)}(x) = q^{(k)}(x) = q^{(k-1)}(x) \quad \forall x \in (0, r_{k-1}].$$

The first of these conditions can be achieved since $f_{j,n}(q^{(k)}) = q^{(k)}(\hat{x}_j)$ if $\hat{x}_j > r_{k-1}$ and we may slightly perturb $q^{(k)}$ to achieve a strictly increasing function, whilst ensuring $f_{j,n}(q^{(k+1)})$ is still admissible for $q^{(k+1)}$. The second condition is consistent with how we selected the functions in the inductive step for odd k. Case (a) of Lemma 2.13 implies that $\Xi_p(F_{q^{(k+1)}}) = \{1\}$. Applying the covering lemma (Lemma 1.20), there exists $n_{k+1} \in \mathbb{N}$ with $n_{k+1} \geq n_k$, a finite set $\mathcal{I}_{k+1} \subset \mathbb{N}^2$ with $\mathcal{I}_k \subset \mathcal{I}_{k+1}$, and $r_{k+1} \in (r_k/2 + \pi, 2\pi)$ such that the following two conditions hold. First, letting J_{k+1} be the set of j for which there exists n with $(j,n) \in \mathcal{I}_{k+1}$, it holds that $\hat{x}_j < r_{k+1}$ whenever $j \in J_{k+1}$. Second,

$$\mathbb{P}\left(\mathrm{dist}(-1, \Gamma_{n_{k+1}}(\tilde{q}^{(k+1)})) \geq 1 \text{ and } C_{k+1}(\tilde{q}^{(k+1)})\right) > 1/2,$$

where $C_{k+1}(\tilde{q})$ is the event that $\Gamma_{n_{k+1}}$ samples from $\{f_{j,n}(q) : (j,n) \in \mathcal{I}_{k+1}\}$ and outputs $\Gamma_{n_{k+1}}(\tilde{q}) \neq \mathrm{NH}$. This completes the inductive step.

Limit argument: Since $q^{(2m+1)}(x) = q^{(2m)}(x) = q^{(2m-1)}(x)$ for $x \in (0, r_{2m-1}]$ for all $m \in \mathbb{N}$ and $\lim_{m\to\infty} r_{2m-1} = 2\pi$, the following pointwise limit exists:

$$q(x) = \lim_{m\to\infty} q^{(2m+1)}(x).$$

The function q satisfies the conditions in Lemma 2.13. Furthermore, we can perform the construction so that $F_q \in \Omega_p$. We define $\tilde{q}$ by setting $f_{j,n}(q) = f_{j,n}(q^{(k)})$ if $(j,n) \in \mathcal{I}_k$ for $k = 1, 2, \ldots$ and $f_{j,n}(q) = q(\hat{x}_j)$ if $(j,n) \notin \cup_{k=1}^{\infty} \mathcal{I}_k$. For each $k \in \mathbb{N}$, let B_k be the event

$$\mathrm{dist}(-1, \Gamma_{n_k}(\tilde{q})) \begin{cases} \geq 1, & \text{if } k \text{ is odd}, \\ \leq 1/2, & \text{if } k \text{ is even}. \end{cases}$$

Due to our construction and the consistency of probabilistic general algorithms, we have $\mathbb{P}(B_k) > 1/2$ for all k. We now argue as in the proof of Theorem 2.9 to obtain the required contradiction. ☐

2.5 Spectra of discrete-space systems

We now consider the state space $\mathcal{X} = \mathbb{N}$, equipped with the usual counting measure $\omega = \sum_{j=1}^{\infty} \delta_j$, and consider the dynamical systems governed by a function $F : \mathbb{N} \to \mathbb{N}$. We define the Koopman operator on sequences $x : \mathbb{N} \to \mathbb{C}$ via

$$[\mathcal{K}_F x](j) = x(F(j)).$$

We consider $\mathcal{K}_F$ as an operator on $L^2(\mathbb{N}, \omega) \cong l^2(\mathbb{N})$, and assume that it is bounded. Our input class and evaluation sets are

$$\Omega_{\mathbb{N}} = \{F \text{ s.t. } F : \mathbb{N} \to \mathbb{N} \text{ and } \mathcal{K}_F \text{ is bounded}\}, \quad \Lambda_{\mathbb{N}} = \{F \mapsto F(j) : j \in \mathbb{N}\}.$$

We are interested in computing $\mathrm{Sp}_{\mathrm{ap}}(\mathcal{K}_F)$.

Theorem 2.15. *Given the above setup, we have the following classifications:*

$$\Delta_3 \not\ni \{\Xi_{\mathrm{Sp}_{\mathrm{ap}}}, \Omega_{\mathbb{N}}, \mathcal{M}_{\mathrm{H}}, \Lambda_{\mathbb{N}}\} \in \Pi_3.$$

In other words, we cannot compute the approximate point spectrum in two limits via any algorithm. However, we can obtain an arithmetic Π_3-tower.

To prove Theorem 2.15, our strategy will be to embed a certain combinatorial problem into the spectral problem of interest. This problem will have a known lower bound complexity, allowing us to prove the lower bound in Theorem 2.15. Specifically, let Ω_{Mat} be the collection of all infinite matrices $a = \{a_{m_1,m_2}\}_{m_1,m_2 \in \mathbb{N}}$ with entries $a_{m_1,m_2} \in \{0,1\}$ and Λ_{Mat} be the set of component-wise evaluation functions. We consider the formula

$$Q(a) = \begin{cases} 1, & \text{if for all but a finite number of } i,\ \forall j\ \exists n > j \text{ s.t. } a_{n,i} = 1, \\ 0, & \text{otherwise}. \end{cases}$$

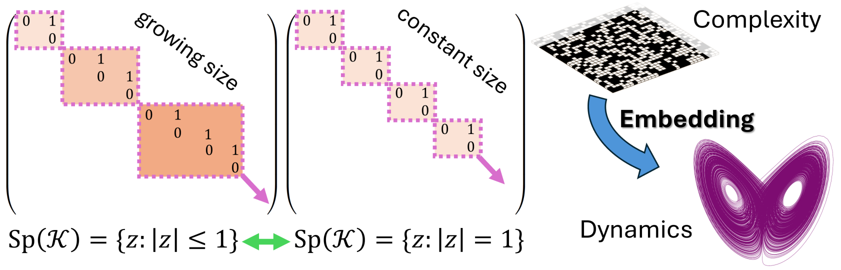

Supplementary Figure 3: Outline of the proof of the lower bound in Theorem 2.15. We embed a combinatorial problem involving matrices of 0s and 1s such that the spectrum fundamentally changes depending on the solution. This construction is analogous to symbolic dynamics, where dynamical systems are studied by encoding orbits as sequences of symbols from a finite alphabet. Many chaotic systems are semi-conjugate or conjugate to a shift on such sequences.

In other words, Q decides whether the matrix has only finitely many columns with only finitely many 1's. It was proven in [21] that $\{Q, \Omega_{\mathrm{Mat}}, [0,1], \Lambda_{\mathrm{Mat}}\} \notin \Delta_3$. Note that the metric space is not $\mathcal{M}_{\mathrm{dec}}$, but $[0,1]$ with the usual metric. We shall also need the following technical lemma and the idea is summarized in Supplementary Figure 3.

Lemma 2.16. *For $n \in \mathbb{N}$, define the perturbed shift matrix*

$$B_n = \begin{pmatrix} 1 & & & \\ 1 & 0 & & \\ & \ddots & \ddots & \\ & & 1 & 0 \end{pmatrix} \in \mathbb{C}^{n \times n}$$

and let B_∞ denote the corresponding limit operator on $l^2(\mathbb{N})$. Then $\mathrm{Sp}_{\mathrm{ap}}(B_\infty) = \mathbb{T}$. For a sequence $\{l_r\}_{r=1}^\infty \subset \mathbb{N}$, define $B(\{l_r\}) = \bigoplus_{r=1}^\infty B_{l_r}$. If $\{l_r\}$ is bounded, $\mathrm{Sp}_{\mathrm{ap}}(B(\{l_r\})) \subset \{0,1\}$, otherwise $\mathrm{Sp}_{\mathrm{ap}}(B(\{l_r\})) = \{z \in \mathbb{C} : |z| \leq 1\}$.

Proof. For the first part, recall that $\mathrm{Sp}_{\mathrm{ap}}(B_\infty)$ is the set of points z for which there exists a unit-norm sequence $\{u_n\} \subset l^2(\mathbb{N})$ with $\lim_{n\to\infty} \|(B_\infty - zI)u_n\| = 0$. Let S_∞ denote the operator on $l^2(\mathbb{N})$ defined by $e_j \mapsto e_{j+1}$. By considering the action of B_∞ on $\mathrm{span}\{e_1\}^\perp$, we see that $\mathrm{Sp}_{\mathrm{ap}}(S_\infty) \subset \mathrm{Sp}_{\mathrm{ap}}(B_\infty)$. Since S_∞ is a non-invertible isometry, $\mathrm{Sp}_{\mathrm{ap}}(S_\infty) = \mathbb{T}$. Now suppose that $z \notin \mathbb{T}$. Then for any unit-norm $u \in l^2(\mathbb{N})$

$$\|(S_\infty - zI)u\| \leq \|(B_\infty - zI)u\| + \|u_1 e_1\| \leq \|(B_\infty - zI)u\| + \frac{1}{|1-z|}\|(B_\infty - zI)u\|.$$

Since $z \notin \mathrm{Sp}_{\mathrm{ap}}(S_\infty)$, $\|(S_\infty - zI)u\|$ is bounded below independently of the choice of u and hence so is $\|(B_\infty - zI)u\|$. It follows that $z \notin \mathrm{Sp}_{\mathrm{ap}}(B_\infty)$.

Suppose that the sequence $\{l_r\}$ is bounded and that $z \notin \{0,1\}$. Then $(B_{l_r} - zI)^{-1}$ exists for all r and is uniformly bounded. Hence, $z \notin \mathrm{Sp}_{\mathrm{ap}}(B(\{l_r\}))$.

For the final case, suppose that the sequence $\{l_r\}$ is unbounded. Let S_n be the matrix B_n, but with the top-left entry set to zero, and define $S(\{l_r\}) = \bigoplus_{r=1}^\infty S_{l_r}$. By arguing as in the first part of the proof, if $z \neq 1$ and $u \in l^2(\mathbb{N})$, then

$$\|(S(\{l_r\}) - zI)u\| \leq \|(B(\{l_r\}) - zI)u\| + \frac{1}{|1-z|}\|(B(\{l_r\}) - zI)u\|.$$

Hence, $\mathrm{Sp}_{\mathrm{ap}}(B(\{l_r\})) \subset \mathrm{Sp}_{\mathrm{ap}}(S(\{l_r\})) \cup \{1\} \subset \{z \in \mathbb{C} : |z| \leq 1\}$. Let $z \in \mathbb{C}$ with $|z| < 1$ and define $u_z = e_n + z e_{n-1} + \cdots + z^{n-1} e_1 \in \mathbb{C}^n$. Then, $(B_n - zI)u_z = (1-z)z^{n-1}e_1$ and hence $\lim_{n\to\infty} \|(B_n - zI)u_z\|/\|u_z\| = 0$. By considering u_z for n corresponding to an increasing subsequence of $\{l_r\}$, we see that $z \in \mathrm{Sp}_{\mathrm{ap}}(B(\{l_r\}))$. Hence, $\mathrm{Sp}_{\mathrm{ap}}(B(\{l_r\})) = \{z \in \mathbb{C} : |z| \leq 1\}$. ☐

Proof of Theorem 2.15. **Step 1: Upper bound.** As an orthonormal basis of $L^2(\mathbb{N}, \omega)$, we consider $\{\phi_n\}_{n=1}^\infty$, where $\phi_n(j) = \delta_{n,j}$. Given $F \in \Omega_{\mathbb{N}}$, let A_F be the matrix representing $\mathcal{K}_F$ with respect to this basis. We can evaluate any entry of A_F using finitely many evaluations from $\Lambda_{\mathbb{N}}$. The upper bound now follows from the Π_3 towers of algorithms in [7].

Step 2: Lower bound. First, given a sequence $\{c_i\}_{i\in\mathbb{N}}$ with each $c_i \in \{0,1\}$, we define the bounded operator $C(\{c_i\})$ acting on $l^2(\mathbb{N})$ via its matrix entries:

$$[C(\{c_i\})]_{k,l} = \begin{cases} 1, & l < k, c_l = c_k = 1, \text{ and } c_n = 0 \text{ for all } l < n < k, \\ 1, & l = k, c_k = 1, \text{ and } c_n = 0 \text{ for all } n < k, \\ 1, & l = k \text{ and } c_k = 0, \\ 0, & \text{otherwise.} \end{cases}$$

Then $C(\{c_i\})$ acts as $B_{\sum_i c_i}$ on the closure of the span of $\{e_i : c_i = 1\}$ and the identity on the orthogonal complement of this subspace. Given $a \in \Omega_{\mathrm{Mat}}$, we consider the family of sequences

$$c_i^{(j)} = c_i^{(j)}(a) = \begin{cases} 1, & i \leq j, \\ a_{i+1-j,j}, & \text{otherwise.} \end{cases}$$

We then consider the following operator acting on $\oplus_{j=1}^{\infty} l^2(\mathbb{N})$: $A(a) = \oplus_{j=1}^{\infty} C(\{c_i^{(j)}\})$. Lemma 2.16 shows that if $Q(a) = 1$, then $\mathrm{Sp}_{\mathrm{ap}}(A(a)) \subset \{0\} \cup \mathbb{T}$. Otherwise, $\mathrm{Sp}_{\mathrm{ap}}(A(a)) = \{z \in \mathbb{C} : |z| \leq 1\}$.

Suppose for a contradiction that $\{\Gamma_{n_2,n_1}\}$ is a height-two tower of general algorithms for $\{\Xi_{\mathrm{Sp}_{\mathrm{ap}}}, \Omega_{\mathbb{N}}, \mathcal{M}_{\mathrm{H}}, \Lambda_{\mathbb{N}}\}$. Given $a \in \Omega_{\mathrm{Mat}}$, we consider the function $F = F_a$ defined as follows. We can identify $\oplus_{j=1}^{\infty} l^2(\mathbb{N})$ with $l^2(\mathbb{N})$ via a computable bijection between the relevant index sets, and, hence, consider $A(a)$ as a bounded operator on $l^2(\mathbb{N})$. When viewed as an infinite matrix, this matrix has exactly one 1 in each row, and every other entry is 0. Moreover, the operator is bounded. By considering this matrix acting with respect to the basis $\{\phi_n\}_{n=1}^{\infty}$, this matrix induces F_a and a corresponding Koopman operator $\mathcal{K}_{F_a}$. Since we know that each row of $A(a)$ has exactly one non-zero entry, any evaluation $F_a(j)$ of F_a can be evaluated in finitely many calls to Λ_{Mat} with input a. It follows that $\tilde{\Gamma}_{n_2,n_1}(a) = \min\{2 \times \mathrm{dist}(1/2, \Gamma_{n_2,n_1}(F_a)), 1\}$ defines general algorithms that map into $[0,1]$ and use the evaluation set Λ_{Mat}. Moreover, the above discussion shows that $\lim_{n_2\to\infty} \lim_{n_1\to\infty} \tilde{\Gamma}_{n_2,n_1}(a) = Q(a)$, the required contradiction. □

3 Pseudocode for algorithms

Here, we provide pseudocode for the algorithms that form the upper bounds in our classifications. The proofs of convergence can be found in the proofs of Theorem 2.3. Each algorithm uses a dictionary $\{g_j\}_{j=1}^{\infty}$. For simplicity, we have given pseudocode for when these basis functions are orthonormal (to be consistent with the proofs), but they can be implemented for non-orthonormal systems.

3.1 Algorithms for the approximate point spectrum and pseudospectrum

We begin by computing the approximate point spectrum and pseudospectrum of Koopman operators. Recall from Section 2.1 that a modulus of continuity α for F allows us to approximate the integrals $\langle \mathcal{K}_F g_j, g_i \rangle$ and $\langle \mathcal{K}_F g_j, \mathcal{K}_F g_i \rangle$ with error bounds using snapshot data. By considering the pencil

$$z \mapsto (\mathcal{K}_F - zI)^*(\mathcal{K}_F - zI),$$

we can use these approximations of inner products to compute

$$h_n(z,F) = \sqrt{\sigma_{\inf}(\mathcal{P}_n(\mathcal{K}_F - zI)^*(\mathcal{K}_F - zI)\mathcal{P}_n^*)}, \tag{15}$$

where $\mathcal{P}_n$ denotes the orthogonal projection onto $\mathrm{span}\{g_1, \ldots, g_n\}$. The functions $h_n(z,F)$ converge locally uniformly (i.e., uniformly on any compact subset of $\mathbb{C}$) to $\sigma_{\inf}(\mathcal{K}_F - zI)$. One always has

$$\sigma_{\inf}(\mathcal{K}_F - zI) \leq \mathrm{dist}(z, \mathrm{Sp}_{\mathrm{ap}}(\mathcal{K}_F)).$$

To compute $\mathrm{Sp}_{\mathrm{ap}}(\mathcal{K}_F)$, we need to compute an upper bound on $\mathrm{dist}(z, \mathrm{Sp}_{\mathrm{ap}}(\mathcal{K}_F))$, so we require an inequality that is the 'other way round'. If the system is measure-preserving, then $\sigma_{\inf}(\mathcal{K}_F - zI) = \mathrm{dist}(z, \mathrm{Sp}_{\mathrm{ap}}(\mathcal{K}_F))$. We can generalize this as follows.

Definition 3.1. Let $\mathfrak{G} : \mathbb{R}_{\geq 0} \to \mathbb{R}_{\geq 0}$ be a strictly increasing function with $\mathfrak{G}(x) \geq x$ and $\lim_{x\to\infty} \mathfrak{G}(x) = \infty$. We say that $\mathcal{K}_F$ has its resolvent bounded by $\mathfrak{G}$ if

$$\mathrm{dist}(z, \mathrm{Sp}_{\mathrm{ap}}(\mathcal{K}_F)) \leq \mathfrak{G}(\sigma_{\inf}(\mathcal{K}_F - zI)) \quad \forall z \in \mathbb{C}. \tag{16}$$

The set of all such systems with modulus of continuity α is denoted by $\Omega_{\mathcal{X}}^{\alpha,\mathfrak{G}}$.

A simple compactness argument shows that, for any bounded $\mathcal{K}_F$, a suitable $\mathfrak{G}$ always exists so that $\mathcal{K}_F$ has resolvent bounded by $\mathfrak{G}$. However, given $\mathcal{K}_F$, we may not know a suitable $\mathfrak{G}$. As with the modulus of continuity α, this uncertainty underlies some of our impossibility results. Typically, we want an algorithm that can handle a whole class of systems, such as $\Omega_{\mathcal{X}}^{\alpha,\mathfrak{G}}$, rather than a single system. Beyond measure-preserving systems, there are dissipative systems where $\mathfrak{G}$ is known [35] and for the systems in this paper, one can take $\mathfrak{G}(x) = cx$ for a constant c that depends on the non-normality of the operator (approximated through pseudospectra).

Supplementary Algorithm 1 A Σ_1-tower of algorithms for computing the spectrum of $\mathcal{K}_F$ for $F \in \Omega_{\mathcal{X}}^{\alpha,\mathfrak{G}}$. We have written the code for an orthonormal dictionary of observables, but the algorithm is easily adapted to the non-orthonormal case.

Input: Function F (in the form of snapshots $\{F(\hat{x}_j) : j = 1, 2, \ldots\}$), dictionary of functions $\{g_j\}_{j=1}^{\infty}$, modulus of continuity α, resolvent bounding function $\mathfrak{G}$, $n \in \mathbb{N}$.

1: Compute the $n \times n$ matrices

$$A_{i,j} = \langle \mathcal{K}_F g_j, g_i \rangle = \int_{\mathcal{X}} g_j(F(x))\overline{g_i(x)}\, \mathrm{d}\omega(x), \quad L_{i,j} = \langle \mathcal{K}_F g_j, \mathcal{K}_F g_i \rangle = \int_{\mathcal{X}} g_j(F(x))\overline{g_i(F(x))}\, \mathrm{d}\omega(x), \quad i, j = 1, \ldots n,$$

using quadrature (evaluation of F at snapshots) and α to bound quadrature errors (see proof of Theorem 2.3).

2: Define the finite grid

$$\mathrm{Grid}(n) = \left\{ z \in \frac{1}{n}\mathbb{Z} + \frac{i}{n}\mathbb{Z} : |z| \leq n \right\}.$$

3: For each $z \in \mathrm{Grid}(n)$, compute

$$h_n(z, F) = \sqrt{\sigma_{\inf}(L - \overline{z}A - zA^* + |z|^2 I)}, \quad d_{n,z} = \mathfrak{G}\left(h_n(z, F)\right).$$

Compute approximate eigenfunctions (corresponding right-singular vectors) if desired.

4: For each $z \in \mathrm{Grid}(n)$, set $\Upsilon_{n,z} = \{w \in \mathrm{Grid}(n) : |z - w| \leq d_{n,z}\}$. If $h_n(z, F) \leq 1/2$, compute the set of local minimizers

$$M_z = \left\{ w \in \Upsilon_{n,z} : h_n(w, F) = \min_{v \in \Upsilon_{n,z}} h_n(v, F) \right\}.$$

Otherwise, set $M_z = \emptyset$.

5: Set $\Gamma_n(F) = \cup_{z \in \mathrm{Grid}(n)} M_z$ and $E_n(w) = d_{n,w}$ for $w \in \Gamma_n(F)$.

Output: $\Gamma_n(F)$, an approximation of $\mathrm{Sp}_{\mathrm{ap}}(\mathcal{K}_F)$, and the error function E_n.

Supplementary Algorithm 1 shows the Σ_1^A-tower of algorithms for the approximate point spectrum of systems in $\Omega_{\mathcal{X}}^{\alpha,\mathfrak{G}}$. The idea is to search for local minimizers of $h_n(z, F)$, where the bound

$$d_{n,z} = \mathfrak{G}\left(h_n(z, F)\right) \geq \mathfrak{G}\left(\sigma_{\inf}(\mathcal{K}_F - zI)\right) \geq \mathrm{dist}(z, \mathrm{Sp}_{\mathrm{ap}}(\mathcal{K}_F))$$

provides a local search radius and the sets M_z are our best estimate of the approximate point spectrum locally near the point $z \in \mathrm{Grid}(n)$. In addition to providing error bounds, the algorithm is local, trivially parallelizable (owing to the separate computation for different z points), and stable.

Without the function $\mathfrak{G}$, we can still compute the approximate point pseudospectrum with error control. This is due to the fact that

$$\mathrm{Sp}_{\mathrm{ap},\epsilon}(\mathcal{K}_F) = \{z \in \mathbb{C} : \sigma_{\inf}(\mathcal{K}_F - zI) \leq \epsilon\}$$

is defined directly in terms of $\sigma_{\inf}(\mathcal{K}_F - zI)$. The process is summarized in Supplementary Algorithm 2 and is a generalization of the ResDMD algorithm in [27]. By taking $\epsilon = 1/n_2$, we obtain a Π_2-tower for the approximate point spectrum, summarized in Supplementary Algorithm 3.

To compute the approximate point pseudospectrum of systems in $\Omega_{\mathcal{X}}$, we can no longer approximate the inner products $\langle \mathcal{K}_F g_j, g_i \rangle$ and $\langle \mathcal{K}_F g_j, \mathcal{K}_F g_i \rangle$ with explicit error bounds (or rates) but can compute them at the cost of a limit. This then requires an alteration, as laid out in the proof of Theorem 2.3, and summarized in Supplementary Algorithm 4, to ensure convergence. Again, we may take $\epsilon = 1/n_3$ to obtain a Π_3-tower of algorithms for the approximate point spectrum, summarized in Supplementary Algorithm 5.

3.2 Algorithms for spectral type and pure point spectrum

To compute the pure point part of the spectral measure, we apply the method of Theorem 2.12, which can be generalized to unitary Koopman operators on arbitrary $\mathcal{X}$. The method is based on the RAGE theorem (Theorem 2.14). Recall that $\{\mathcal{P}_n\}_{n \in \mathbb{N}}$ is a sequence of increasing finite-rank orthogonal projections such that $\mathcal{P}_n^* \mathcal{P}_n$ converges strongly to the identity. For example, we can take $\mathcal{P}_n$ to be the orthogonal projection onto $\mathrm{span}\{g_1, \ldots, g_n\}$ (as in the previous subsection). Supplementary Algorithm 6 summarises the procedure, where we consider the pure point part of the spectral measure of an observable g measured on an open set U. In practice, the indicator function $\chi_U(\mathcal{K}_F)$ is computed in steps 2 and 3 using the measure-preserving EDMD algorithm [22], which provides a unitary Galerkin approximation of $\mathcal{K}_F$. The approximation of the functional calculus is outlined in [22, Section 5]. To decide whether the pure point spectrum of $\mathcal{K}_F$ (away from 1) is empty,

Supplementary Algorithm 2 A Σ_1-tower of algorithms for computing the pseudospectrum of $\mathcal{K}_F$ for $F \in \Omega_{\mathcal{X}}^{\alpha}$. We have written the code for an orthonormal dictionary of observables, but the algorithm is easily adapted to the non-orthonormal case.

Input: Function F (in the form of snapshots $\{F(\hat{x}_j) : j = 1, 2, \ldots\}$), dictionary of functions $\{g_j\}_{j=1}^{\infty}$, modulus of continuity α, $n \in \mathbb{N}$, $\epsilon > 0$.

1: Compute the $n \times n$ matrices

$$A_{i,j} = \langle \mathcal{K}_F g_j, g_i \rangle = \int_{\mathcal{X}} g_j(F(x))\overline{g_i(x)}\, d\omega(x), \quad L_{i,j} = \langle \mathcal{K}_F g_j, \mathcal{K}_F g_i \rangle = \int_{\mathcal{X}} g_j(F(x))\overline{g_i(F(x))}\, d\omega(x), \quad i, j = 1, \ldots n,$$

using quadrature (evaluation of F at snapshots) and α to bound quadrature errors.

2: Define the finite grid

$$\mathrm{Grid}(n) = \left\{ z \in \frac{1}{n}\mathbb{Z} + \frac{i}{n}\mathbb{Z} : |z| \le n \right\}.$$

3: For each $z \in \mathrm{Grid}(n)$, compute $h_n(z, F) = \sqrt{\sigma_{\inf}(L - \overline{z}A - zA^* + |z|^2 I)}$.

4: Set $\Gamma_n^{\epsilon}(F) = \{z \in \mathrm{Grid}(n) : h_n(z, F) < \epsilon\}$.

Output: $\Gamma_n^{\epsilon}(F)$, an approximation of $\mathrm{Sp}_{\mathrm{ap},\epsilon}(\mathcal{K}_F)$.

Supplementary Algorithm 3 A Π_2-tower of algorithms for computing the spectrum of $\mathcal{K}_F$ for $F \in \Omega_{\mathcal{X}}^{\alpha}$. We have written the code for an orthonormal dictionary of observables, but the algorithm is easily adapted to the non-orthonormal case.

Input: Function F (in the form of snapshots $\{F(\hat{x}_j) : j = 1, 2, \ldots\}$), dictionary of functions $\{g_j\}_{j=1}^{\infty}$, modulus of continuity α, $n_1, n_2 \in \mathbb{N}$.

1: Set $\Gamma_{n_2,n_1}(F)$ to be the output of Supplementary Algorithm 2 with $\epsilon = 1/n_2$ and $n = n_1$.

Output: $\Gamma_{n_2,n_1}(F)$, an approximation of $\mathrm{Sp}_{\mathrm{ap}}(\mathcal{K}_F)$.

Supplementary Algorithm 4 A Σ_2-tower of algorithms for computing the pseudospectrum of $\mathcal{K}_F$ for $F \in \Omega_{\mathcal{X}}$. We have written the code for an orthonormal dictionary of observables, but the algorithm is easily adapted to the non-orthonormal case.

Input: Function F (in the form of snapshots $\{F(\hat{x}_j) : j = 1, 2, \ldots\}$), dictionary of functions $\{g_j\}_{j=1}^{\infty}$, $n_1, n_2 \in \mathbb{N}$, $\epsilon > 0$.

1: Let A' and L' be n_1-point quadrature approximations of the $n_2 \times n_2$ matrices

$$A_{i,j} = \langle \mathcal{K}_F g_j, g_i \rangle = \int_{\mathcal{X}} g_j(F(x))\overline{g_i(x)}\, d\omega(x), \quad L_{i,j} = \langle \mathcal{K}_F g_j, \mathcal{K}_F g_i \rangle = \int_{\mathcal{X}} g_j(F(x))\overline{g_i(F(x))}\, d\omega(x), \quad i, j = 1, \ldots n_2,$$

using evaluation of F at snapshots.

2: Define the finite grid

$$\mathrm{Grid}(n_2) = \left\{ z \in \frac{1}{n_2}\mathbb{Z} + \frac{i}{n_2}\mathbb{Z} : |z| \le n_2 \right\}.$$

3: For each $z \in \mathrm{Grid}(n_2)$, compute $h_{n_2,n_1}(z, F) = \sqrt{\sigma_{\inf}(L' - \overline{z}A' - zA'^* + |z|^2 I)}$.

4: Consider the separated intervals

$$I_{n_2}^1(\epsilon) = [0, \epsilon - 1/n_2], \quad I_{n_2}^2(\epsilon) = [\epsilon + 1/(2n_2), \infty).$$

Given $h_{n_2,j}(z, F)$ for $j = 1, \ldots, n_1$, let k be the largest such j with $h_{n_2,j}(z, F) \in I_{n_2}^1(\epsilon) \cup I_{n_2}^2(\epsilon)$. If such a k exists with $h_{n_2,k}(z, F) \in I_{n_2}^1(\epsilon)$, then $z \in \Gamma_{n_2,n_1}^{\epsilon}(F)$. Otherwise, $z \notin \Gamma_{n_2,n_1}^{\epsilon}(F)$.

Output: $\Gamma_{n_2,n_1}^{\epsilon}(F)$, an approximation of $\mathrm{Sp}_{\mathrm{ap},\epsilon}(\mathcal{K}_F)$.

Supplementary Algorithm 5 A Π_3-tower of algorithms for computing the spectrum of $\mathcal{K}_F$ for $F \in \Omega_{\mathcal{X}}$. We have written the code for an orthonormal dictionary of observables, but the algorithm is easily adapted to the non-orthonormal case.

Input: Function F (in the form of snapshots $\{F(\hat{x}_j) : j = 1, 2, \ldots\}$), dictionary of functions $\{g_j\}_{j=1}^{\infty}$, $n_1, n_2, n_3 \in \mathbb{N}$.

1: Set $\Gamma_{n_3,n_2,n_1}(F)$ to be the output of Supplementary Algorithm 4 with $\epsilon = 1/n_3$.

Output: $\Gamma_{n_3,n_2,n_1}(F)$, an approximation of $\mathrm{Sp}_{\mathrm{ap}}(\mathcal{K}_F)$.

we alter Supplementary Algorithm 6 as outlined in the proof of Theorem 2.12. The idea is to set $U = \mathbb{T}\backslash\{1\}$ and consider a set $\{g_k\}_{k=1}^{\infty}$ that form a dense subset of $L^2(\mathcal{X}, \omega)$. The process is summarized in Supplementary Algorithm 7.

Supplementary Algorithm 6 A Σ_2-tower of algorithms for computing the pure point part of spectral measures of a general unitary Koopman operator $\mathcal{K}_F$.

Input: Function F (in the form of snapshots $\{F(\hat{x}_j) : j = 1, 2, \ldots\}$), dictionary of functions $\{g_j\}_{j=1}^{\infty}$, observable $g \in L^2(\mathcal{X}, \omega)$, open set $U \subset \mathbb{T}$, $n_1, n_2 \in \mathbb{N}$.

Compute the $n_2 \times n_2$ matrix

$$A_{i,j} = \langle \mathcal{K}_F g_j, g_i \rangle = \int_{\mathcal{X}} g_j(F(x)) \overline{g_i(x)} \, d\omega(x), \quad i, j = 1, \ldots, n_2.$$

Compute an SVD of $A^* = U_1 \Sigma U_2^*$.

Compute the eigendecomposition $U_2 U_1^* = V \Lambda V^*$ and let Λ_U and V_U be the subset of eigenpairs with eigenvalues in U.

Compute $m_k = \|\Lambda_U^k V_U^* g\|^2 / (2n_1 + 1)$ for $k = -n_1, \ldots, n_1$.

Set $\Gamma_{n_2,n_1}(\mathcal{K}_F, g, U) = \min\left\{\sum_{k=-n_1}^{n_1} m_k, \|g\|^2\right\}$.

Output: $\Gamma_{n_2,n_1}(\mathcal{K}_F, g, U)$, an approximation of $\mu_g^{(\mathrm{pp})}(U)$.

Supplementary Algorithm 7 A Σ_2-tower of algorithms for the decision problem $\Xi_p^{\mathrm{dec}}(F)$, which decides whether the pure point spectrum of a unitary Koopman operator $\mathcal{K}_F$ is larger than $\{1\}$.

Input: Function F (in the form of snapshots $\{F(\hat{x}_j) : j = 1, 2, \ldots\}$), dense set $\{g_k\}_{k=1}^{\infty}$ of observables, $n_1, n_2 \in \mathbb{N}$.

For $k = 1, \ldots, n_2$, let $W_{n_2,n_1}(g_k)$ be the output of Supplementary Algorithm 6 with $g = g_k$ and $U = \mathbb{T} \backslash \{1\}$.

Set

$$a_{n_2,n_1}(F) = \max_{1 \le k \le n_2} W_{n_2,n_1}(g_k).$$

Define the two separated intervals $I_1 = [0, 1/4]$ and $I_2 = [1/2, \infty)$.

Set $\Gamma_{n_2,n_1}(F) = 0$ if the largest $l = 1, \ldots, n_1$ with $a_{n_2,l} \in I_1 \cup I_2$ has $a_{n_2,l} \in I_1$. If no such l exists, or $a_{n_2,l} \in I_2$, set $\Gamma_{n_2,n_1}(F) = 1$.

Output: $\Gamma_{n_2,n_1}(F)$, an approximation of $\Xi_p^{\mathrm{dec}}(F)$.

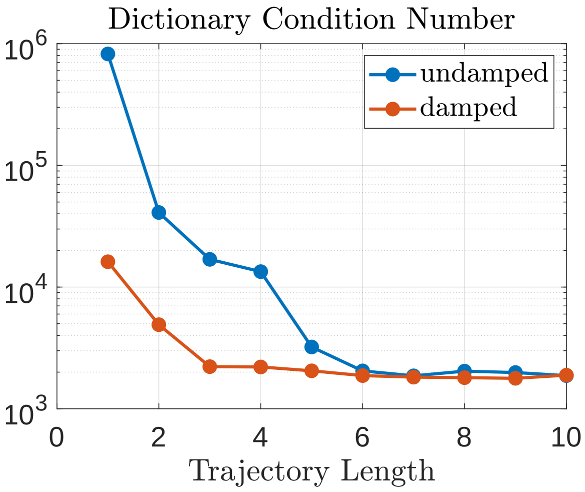

Supplementary Figure 4: Conditioning of the dictionary for $N = 500$ and various trajectory lengths for the first set of data used to find the centers of the radial basis functions. The choice of trajectory length 5 produces a well-conditioned dictionary for the examples in this paper.

4 Further experimental diagnostics

4.1 Further analysis for Duffing oscillator

Supplementary Figure 4 shows the condition number of the dictionary for the Duffing oscillator example, computed using k-means clustering for different initial trajectory lengths (shown on the horizontal axis). The condition number is defined as follows: we evaluate the dictionary on a second, independent set of data points for Monte Carlo estimation, yielding an $M \times N$ matrix. The condition number of this matrix approximates how well-conditioned the dictionary is. A trajectory length of 5 produces a well-conditioned basis and is used for all examples in the paper.

Supplementary Figure 5 illustrates convergence for the Duffing oscillator example. The left panel shows the convergence

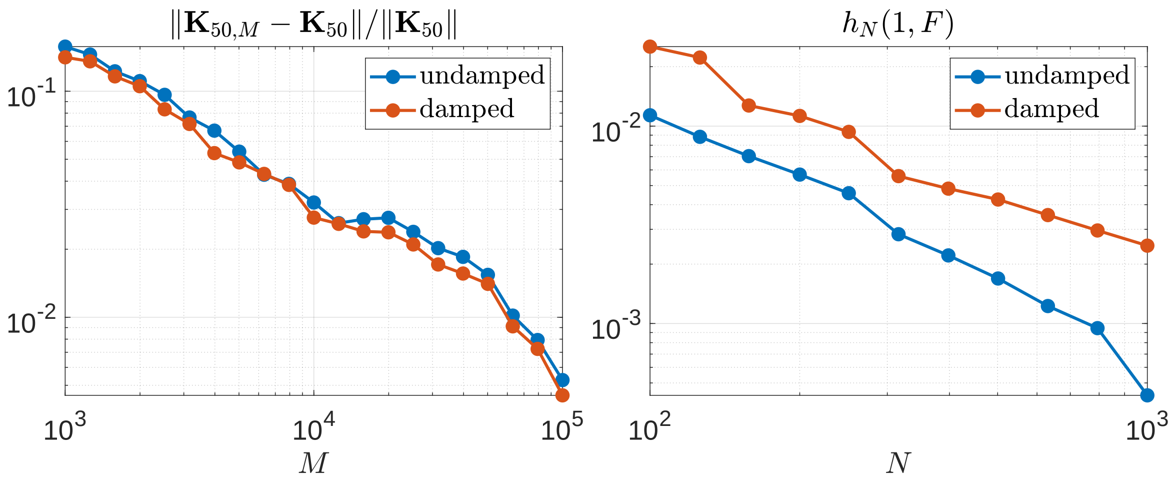

Supplementary Figure 5: Left: Convergence of the EDMD matrix to its large-data limit, with relative error measured in the Frobenius norm. The observed convergence rate is approximately $\mathcal{O}(M^{-1/2})$. Right: Convergence of the spectral distance function $h_N(z, F)$ at $z = 1$, a representative spectral point. The results confirm the theoretical convergence guarantees of our method.

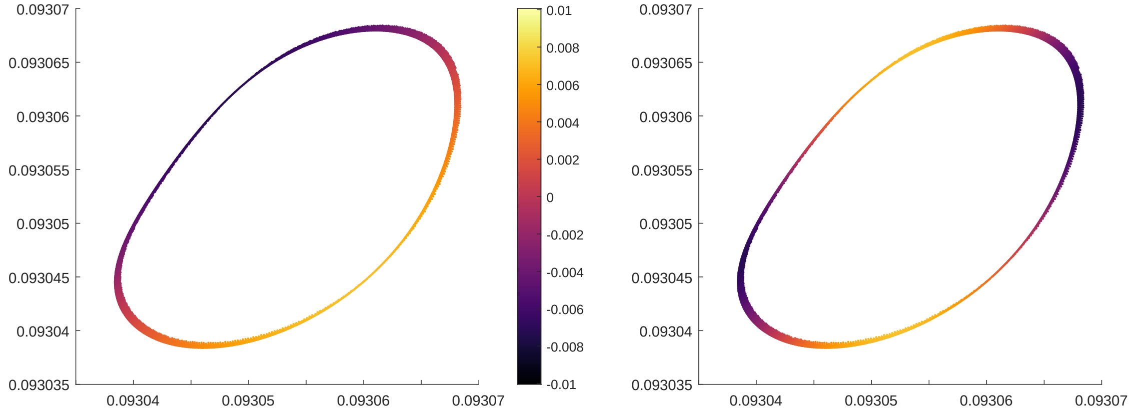

Supplementary Figure 6: Limit cycle of the cavity flow at $Re = 13,000$. The horizontal axis represents the kinetic energy, and the vertical axis represents the time-delayed kinetic energy. The plot shows the real part of the eigenfunction corresponding to the fundamental eigenvalue λ (left) and its square λ^2 (right).

of the EDMD matrix $\mathbf{K}_{N,M}$ (computed as outlined in the main text with a dictionary of N observables and M snapshot data points) to its large-data limit (after taking $M \to \infty$ so that corresponding correlations have converged) $\mathbf{K}_N$ for $N = 50$. The error is measured as the relative Frobenius norm, the square root of the sum of the squared absolute values of the matrix entries. The convergence rate is approximately the Monte Carlo rate $\mathcal{O}(M^{-1/2})$ [14]. The right panel shows the convergence of the function $h_N(z, F)$ (see Equation (15)) for $z = 1$, which is chosen to demonstrate convergence since it lies in the spectrum.

4.2 Further analysis for cavity flow

Here, we plot the extracted eigenfunctions for the cavity flow, illustrating how their structure is related to the geometry of the attractor at different Reynolds numbers. To display the attractor, we use the total kinetic energy as one coordinate and the delayed kinetic energy with a time delay of 1 second. For $Re = 13,000$, the attractor forms a limit cycle, so we use one time delay (i.e., the attractor in a two-dimensional state space). For larger Reynolds numbers, we use two time delay coordinates (i.e., the attractor in a three-dimensional state space).

To extract the eigenfunctions, we begin with a 20-dimensional subspace of observables, constructed from time delays of the kinetic energy as described in the Methods section of the main text for this example. We then apply Supplementary Algorithm 6 (the algorithmic realization of the RAGE theorem) to project these onto the eigenspaces of the Koopman operator, effectively removing any continuous spectral components. Next, we run Supplementary Algorithm 1 to compute the associated spectrum on this subspace, which yields the eigenvalues and eigenfunctions. We use the full $M = 20000$ snapshots in all cases.

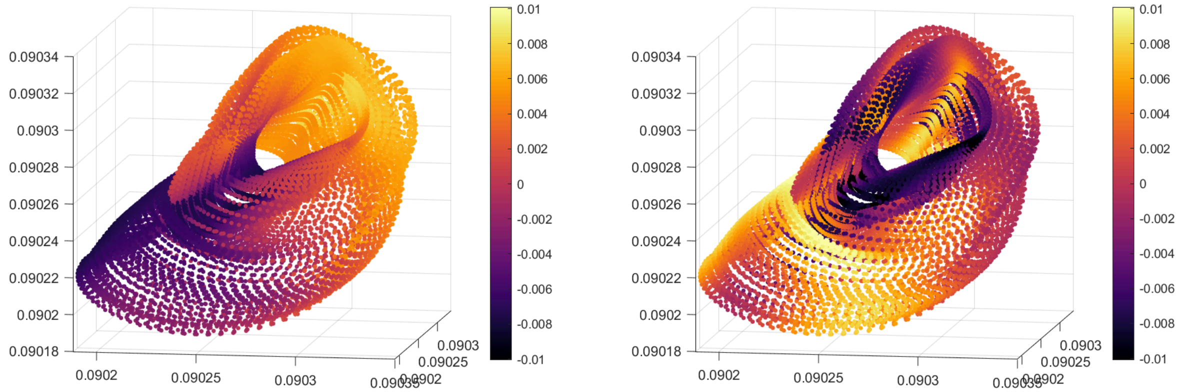

Supplementary Figure 7: Torus attractor of the cavity flow at $Re = 16,000$. The axes represent the kinetic energy and two time-delayed kinetic energies. The plot shows the real part of the eigenfunctions corresponding to the fundamental eigenvalues λ and μ.

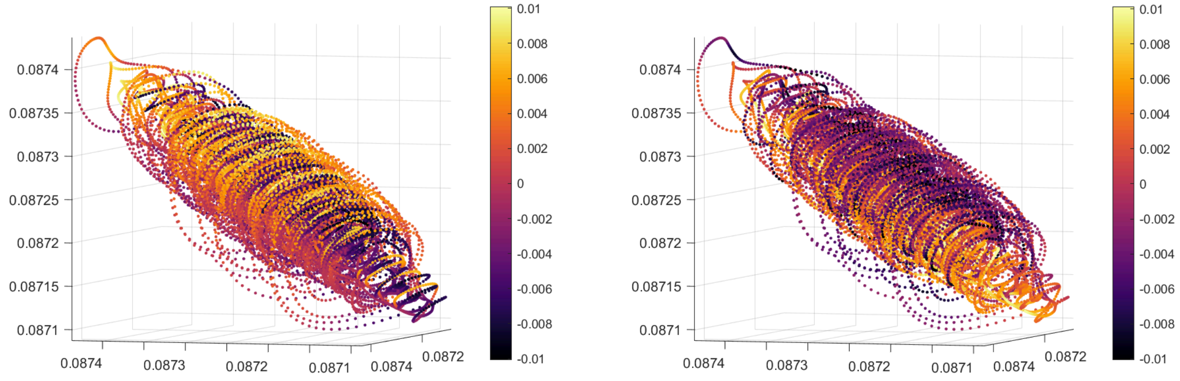

Supplementary Figure 8: Skew-periodic attractor of the cavity flow at $Re = 19,000$. The axes represent the kinetic energy and two time-delayed kinetic energies. The plot shows the real part of the eigenfunctions corresponding to the two basic frequencies of the discrete part of the spectrum.

The results are shown in Supplementary Figures 6 to 8 for various Reynolds numbers. For $Re = 13,000$, the attractor is a limit cycle. The plot displays the eigenfunction corresponding to the fundamental eigenvalue λ (left) and its square λ^2 (right). The color represents the real part of the eigenfunction at each point. The doubling of frequency from left to right is consistent with a Koopman spectrum consisting of powers $\{\lambda^n\}$. For $Re = 16,000$, the attractor is a torus, corresponding to two fundamental eigenvalues, λ and μ (quasiperiodic dynamics). The Koopman eigenfunctions reveal the directions on the torus where the evolution is linear and periodic. For $Re = 19,000$, the attractor is skew-periodic, with a strong discrete spectrum (in the energy sense) consisting of two basic frequencies and a relatively weak continuous component. The corresponding eigenfunctions are plotted as functions of the kinetic energy and two time delays, as before. These eigenfunctions provide geometric slices on which the motion is purely quasiperiodic.

4.3 Further analysis for Arctic sea ice

Supplementary Figure 9 shows the time evolution of the hidden decaying modes from the main text computed over the first and second halves of the dataset. The magnitude decays approximately exponentially at approximately the rate expected from the eigenvalue λ (dashed line), suggesting a connection to long-term sea-ice decline associated with climate change. This connection is likely nuanced: dissipative Koopman eigenfunctions exhibit near-monotonic reduction as time increases, whereas observed sea-ice loss rates over recent decades are known to be non-monotonic. Since the full sea-ice state is expressed as a sum of modes, the near-monotonic behavior of ϕ_{decay} does not contradict the non-monotonic trend patterns seen in the data. In particular, the second half of the dataset produces an eigenfunction with a slightly faster rate of decay than the first, although the slopes are broadly consistent with those obtained from the full-interval analysis. The oscillations of the eigenfunctions follow the period expected from the complex argument of the associated eigenvalues.

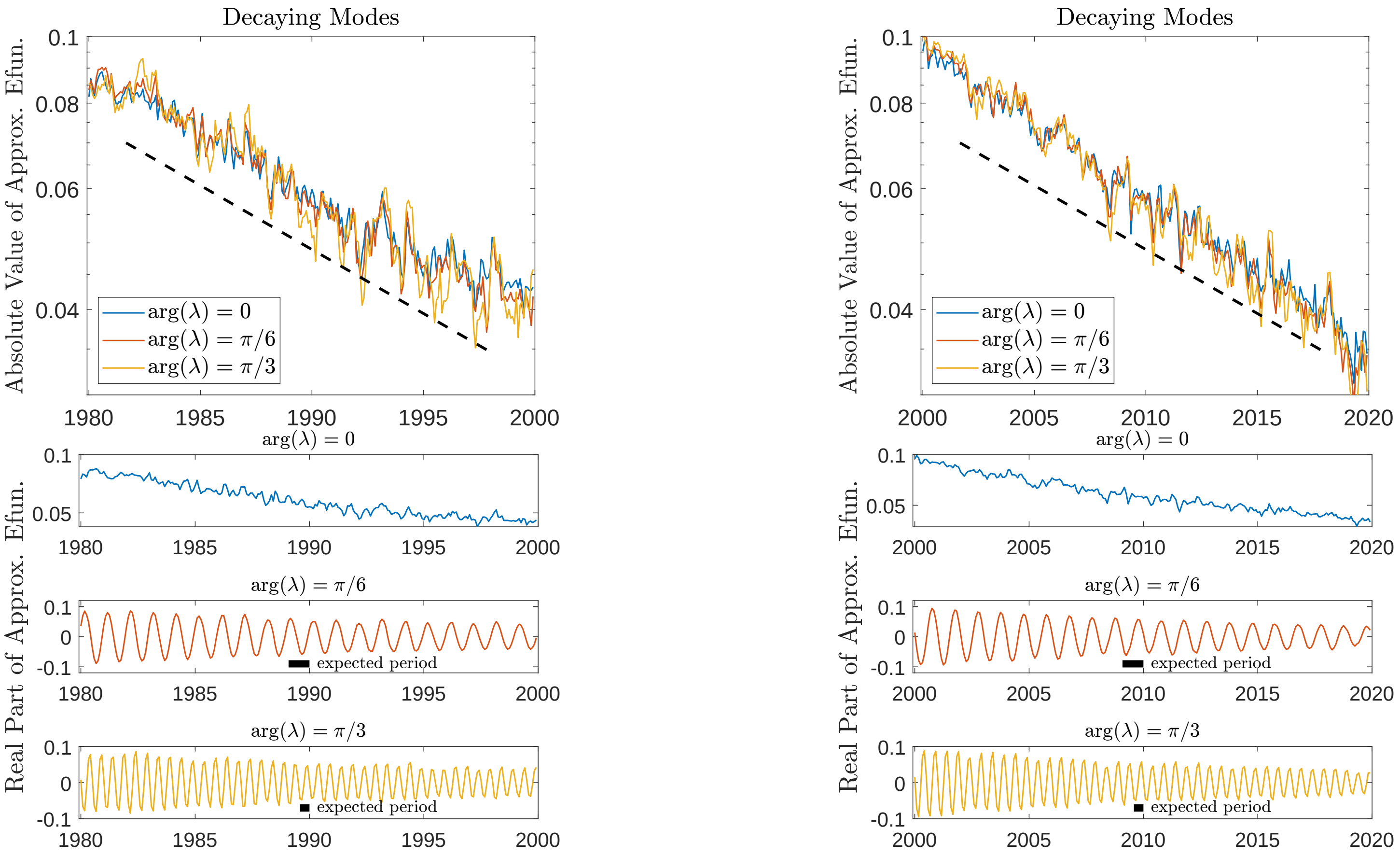

Supplementary Figure 9: Time evolution of the hidden decaying Koopman eigenfunction ϕ_{decay} computed separately over the first and second halves of the dataset. We only show those whose eigenvalue have non-negative complex arguments due to complex-conjugate symmetry. The top row shows the absolute value $|\phi_{\text{decay}}|$, and the bottom row shows the real part $\text{Re}(\phi_{\text{decay}})$. The left and right columns correspond to the first and second halves of the dataset, respectively. The dashed lines indicate the exponential decay rate predicted by the associated eigenvalue λ. Both halves exhibit near-monotonic decay with broadly consistent slopes, indicating robustness of the estimated long-term decay mode across the analysis interval.

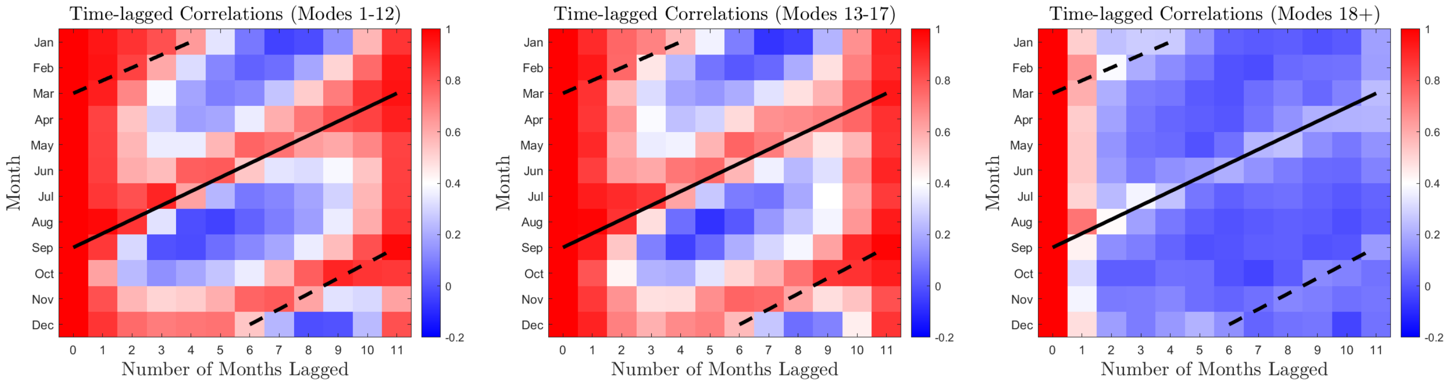

Supplementary Figure 10: Time-lagged pattern correlations of sea ice anomalies. Solid lines indicate months with increased correlation due to melt-to-growth reemergence, while dashed lines indicate increased correlation due to growth-to-melt reemergence. The modes are ordered by their approximation errors: the leftmost corresponds to the dominant annual variation, the middle group contains the five hidden decaying modes, and the rightmost includes the remaining modes.

Supplementary Figure 10 shows the autocorrelation functions of sea ice anomalies reconstructed from individual Koopman modes. Correlations are computed by treating the sea ice concentration at each grid point as a component of a vector. Notably, the hidden modes capture seasonally modulated reemergence of correlations, whereby sea ice anomalies that develop during the growth season reappear in the following melt season, despite a loss of correlation during the intervening winter months [10]. This behavior highlights the Koopman decomposition's ability to capture meaningful long-term memory in sea ice

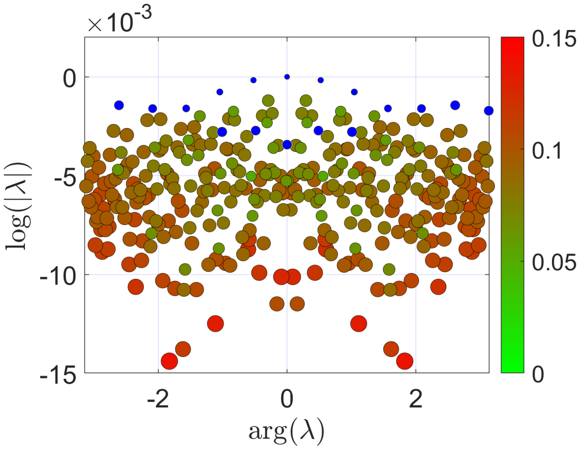

Supplementary Figure 11: Koopman spectrum for 2012–2021 Arctic sea ice data. The spectral features and reconstruction errors are similar to those obtained from the full dataset.

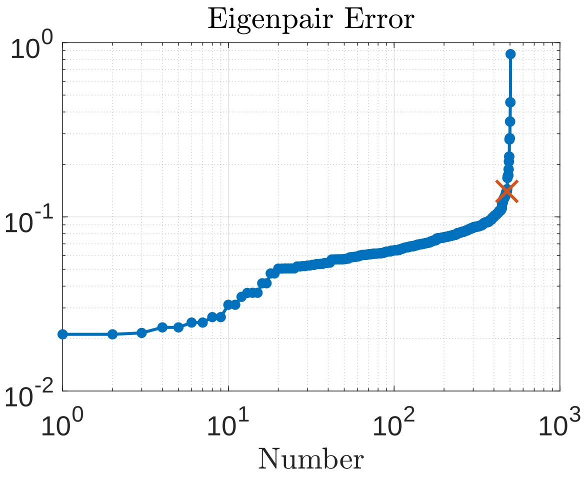

Supplementary Figure 12: Eigenpair errors for the Arctic sea ice example computed using the method outlined in the proof of Theorem 2.3. We have ordered the pairs according to their errors and the red cross shows a typical elbow point of the curve, which we use for optimal truncations in the main text.

dynamics.

Supplementary Figure 11 shows the Koopman spectrum computed using data from 2012–2021 (and the same methods used to produce Figure 2 in the main text), the period used in the binary accuracy forecasting experiment. The resulting spectra and associated errors are broadly similar to those obtained from the full dataset. However, the precise locations of the hidden modes vary, and the error metric is generally higher when using a smaller training dataset, as expected.

We now plot the errors of the EDMD eigenvalues in Figure 2 of the main text. Supplementary Figure 12 presents the results, with eigenpairs ordered by their computed errors. To mitigate spurious eigenpairs in Koopman mode expansions, we truncate at the elbow of the curve, marked by the red cross. This provides a principled way to choose the truncation parameter ε_0 in the Koopman mode decomposition described in the Methods section.

4.4 Other dynamical systems

We now provide further details of the additional experiments in the main text, which we reproduce in Supplementary Figure 13 for the convenience of the reader. In each case, we specify the dynamical system, how the data were collected, and the choice of observables. This information fully specifies the input required for the EDMD algorithm (see main text) and our convergent algorithms, both of which use the same data and dictionary for each system.

4.4.1 Flow past a cylinder

We consider flow past a circular cylinder of diameter $D = 1$ with Reynolds number $Re = 100$, which exceeds the critical Reynolds number at which the flow undergoes a supercritical Hopf bifurcation, resulting in laminar vortex shedding [37, 67].

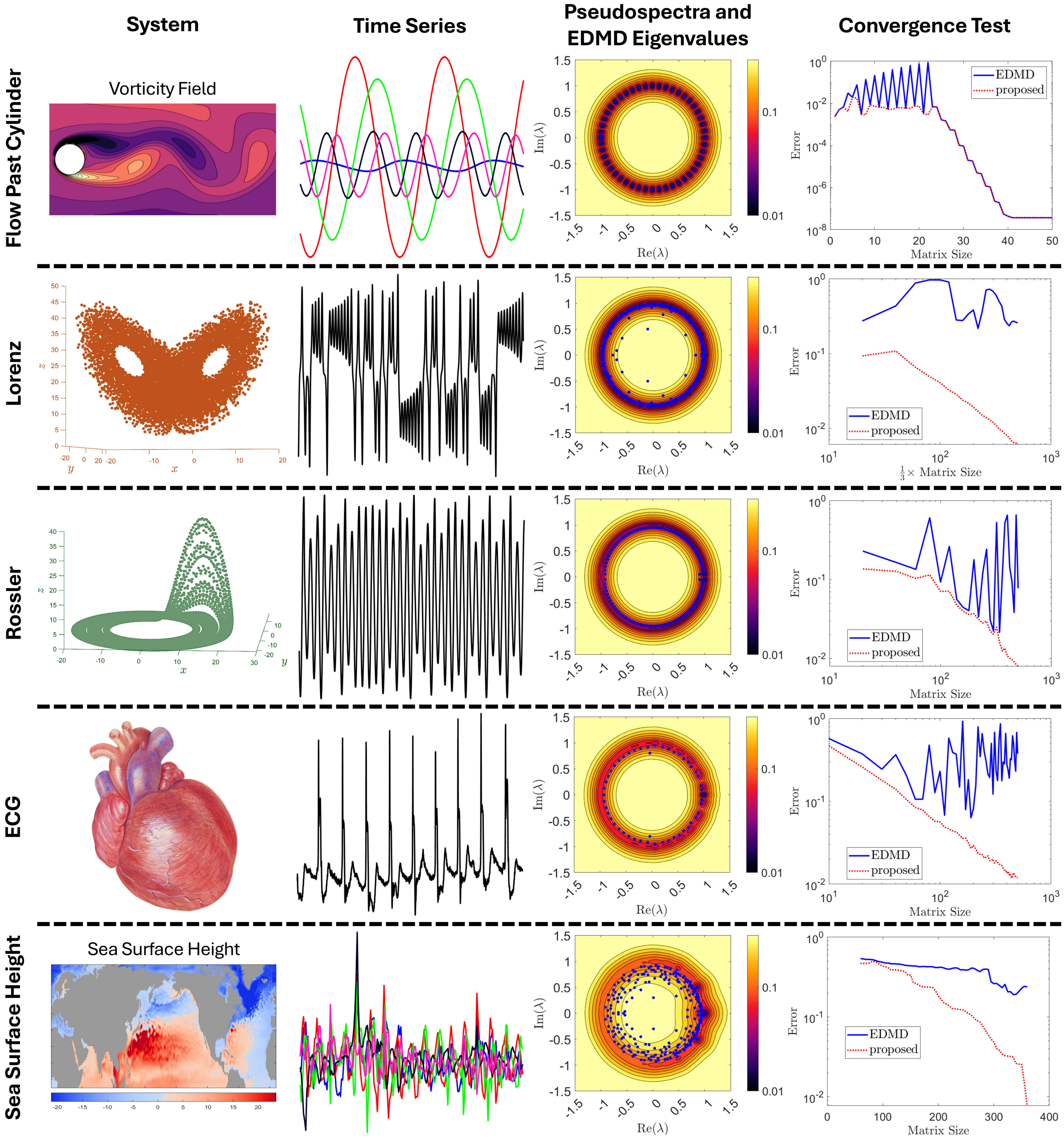

Supplementary Figure 13: Spectral analysis of a range of analytic and real-world dynamical systems using our proposed algorithms. Each row corresponds to a different system: (i) periodic flow past a cylinder, (ii) Lorenz system, (iii) Rössler system, (iv) electrocardiogram (ECG) data (Image: *Heart* by H. G. Wetselaar, Leiden University Libraries / Europeana, Public Domain), and (v) monthly mean sea surface height in the Northern Hemisphere (1950–present). Columns show: the system or dataset, a representative time series of observables, the computed pseudospectra (the sublevel sets of ε for ε-approximate eigenfunctions) overlaid with EDMD eigenvalues (blue dots), and a convergence comparison between EDMD (blue) and our method. Except for the periodic flow, EDMD yields spurious eigenvalues and lacks convergence. In contrast, our approach produces qualitatively accurate and convergent spectral approximations. Notably, the pseudospectra reveal distinct spectral structures across systems: continuous spectra for chaotic systems (Lorenz, Rössler), spectral clustering near $\lambda = 1$ for ECG, and non-normal features and seasonal modes around $\lambda \approx \exp(2\pi i/12)$ in sea surface height data.

The flow has a stable limit cycle representative of the three-dimensional flow [55, 54]. Due to its simplicity and relevance in engineering, this is one of the most studied examples in modal-analysis techniques [60, Table 3], [16, 64]. The Koopman operator of the post-transient flow is unitary with eigenvalues $\{\lambda_0^n : n \in \mathbb{Z}\}$ on the unit circle [4]. We compute the vorticity field using an incompressible, two-dimensional lattice-Boltzmann solver [39, 63], with a time step so that approximately 24 snapshots of the flow field correspond to the period of vortex shedding. The computational domain is $18D \times 5D$, with a 800×200 grid resolution and ambient state-space dimension $d = 158624$. This is less than 800×200 due to the presence of the cylinder, which is positioned $2D$ downstream of the inlet at the mid-height of the domain. The cylinder side walls are bounce-back and no-slip, with a parabolic velocity profile at the inlet of the domain and non-reflecting outflow at the outlet. After simulations converge to statistically stationary periodic vortex shedding, we collect $M = 120$ snapshots.

Let $\mathbf{X} \in \mathbb{R}^{d\times 120}$ be the data matrix of the vorticity field at the snapshots and $\mathbf{Y} \in \mathbb{R}^{d\times 120}$ the corresponding matrix after one time step. We compute a truncated SVD of the data matrix $\mathbf{X} \approx \mathbf{U\Sigma V}^*$, $\mathbf{U} \in \mathbb{R}^{d\times N}$, $\mathbf{\Sigma} \in \mathbb{R}^{N\times N}$, $\mathbf{V} \in \mathbb{R}^{M\times N}$. The columns of $\mathbf{U}$ and $\mathbf{V}$ are orthonormal and $\mathbf{\Sigma}$ is diagonal. As our dictionary, we use the N first left singular vectors of the data matrix of the vorticity field (POD modes [8]). The jth row of the POD matrix $\mathbf{U}^*\mathbf{X}$ is an affine function evaluated at the snapshot data. The pseudospectra plot was computed using $N = 50$ observables. The trajectory data plotted in the figure in the paper (Supplementary Figure 13 in S.I.) correspond to the first five POD modes (note the periodic behavior).

4.4.2 Lorenz system

The famous Lorenz (63) system [44] is the following three coupled ordinary differential equations:

$$\dot{u}_1 = \sigma\left(u_2 - u_1\right), \quad \dot{u}_2 = u_1\left(\rho - u_3\right) - u_2, \quad \dot{u}_3 = u_1 u_2 - \beta u_3.$$

The system describes a truncated model of Rayleigh–Bénard convection, where the parameters σ, ρ, and β are proportional to the Prandtl number, Rayleigh number, and the physical proportions of the fluid, respectively. We take the standard values $\sigma = 10$, $\rho = 28$, and $\beta = 8/3$ and consider the dynamics of $x = (u_1, u_2, u_3)$ on the Lorenz attractor $\mathcal{X} \subset \mathbb{R}^3$. We consider a discrete-time dynamical system by sampling with a time-step $\Delta t = 0.05$. Hence, $F(x)$ is the solution at $t = 0.05$ to the above equations with initial value x. This system has a unique SRB measure ω on $\mathcal{X}$ [65], meaning that for Lebesgue-almost every initial condition x_0 in the basin of attraction of $\mathcal{X}$ and for every compactly supported continuous function $g : \mathbb{R}^3 \to \mathbb{C}$,

$$\lim_{M\to\infty} \frac{1}{M} \sum_{m=0}^{M-1} \left[\mathcal{K}^m g\right](x_0) = \int_{\mathcal{X}} g(x)\, \mathrm{d}\omega(x).$$

We use a time-delay embedding dictionary. Specifically, for $j = 1, 2, 3$, we define the observables

$$g_{j,1}(x) = u_j, \quad g_{j,k+1} = \mathcal{K} g_{j,k}, \quad k = 1, \ldots, N-1.$$

We consider M snapshots $x^{(1)} = x_0$ and $x^{(m+1)} = F(x^{(m)})$ drawn from a single trajectory with quadrature weights $w_m = 1/M$. Since $g_{j,k}(x^{(m)}) = g_{j,1}(x^{(m+k-1)})$, we can evaluate the dictionary from the single trajectory. We use the `ode45` command in MATLAB to collect the data after an initial 'burn-in' time to ensure that the initial point x_0 is (approximately) on the Lorenz attractor. The system is chaotic, so we cannot hope to numerically integrate accurately for long periods. However, convergence is still obtained as $M \to \infty$ due to an effect known as shadowing [57]. The pseudospectra plot was computed using $N = 100$ (300 observables) and $M = 10^4$ snapshots, and the shown trajectory data is for u_1 (first coordinate).

4.4.3 Rössler system

The Rössler system [59] is defined by the following three coupled ordinary differential equations:

$$\dot{u}_1 = -u_2 - u_3, \quad \dot{u}_2 = u_1 + 0.1u_2, \quad \dot{u}_3 = 0.1 + u_3(u_1 - 14).$$

We study the dynamics of $x = (u_1, u_2, u_3)$ on the Rössler attractor. Although often regarded as a simplified analogue of the Lorenz (63) system, the Rössler system—despite its relatively simple form—exhibits rich and complex dynamical behavior. The setup for data collection and dictionary construction is the same as in the Lorenz example, with one key difference: to illustrate the use of alternative dictionaries, we define the observables as

$$g_1(x) = u_1, \quad g_{k+1} = \mathcal{K} g_k, \quad k = 1, \ldots, N-1,$$

for our dictionary. The pseudospectra plot was computed using $N = 100$ observables and $M = 10^4$ snapshots, and the shown trajectory data is for u_1 (first coordinate).

4.4.4 Electrocardiogram (ECG)

An electrocardiogram (ECG) measures the electrical activity of the heart, producing the characteristic spiking pulses associated with each heartbeat. In this analysis, we use the ECG signal qtdb/sel102 from [40] with $M = 44499$ snapshots. We construct a dictionary consisting of $N-1$ time delays, yielding N observables in total, following the same approach described in the Methods section for the Arctic sea ice example. This type of time-delay dictionary is commonly used in the analysis of ECG recordings [62, 58]. The pseudospectra plot was computed using $N = 50$ observables.

4.4.5 Northern Hemisphere sea surface height

This final example examines the dynamics of the monthly mean sea surface height in the Northern Hemisphere. We use the OFES simulation of hindcast data from 1950 to the present day ($M = 359$ snapshots), which was conducted on the Earth Simulator with the support of JAMSTEC [38]. The dataset spans latitudes from 74.95°S to 74.95°N in intervals of 0.1°(excluding Arctic regions), and longitudes from 0.05°to 359.95°, also in intervals 0.1°. Since the Northern and Southern Hemispheres exhibit different dynamics, we restrict our analysis to latitudes between 0.05°N and 74.95°N. At each longitude-latitude point, the dataset provides monthly mean sea surface height, and we exclude any regions that are not ocean-covered.

We construct the dictionary using a kernelized version of the POD dictionary used for the cylinder flow. The description is given in [56], but amounts to kernel basis functions of the form

$$\left(1 + \mathbf{x}^\top \mathbf{x}^{(m)}\right)^{20} \exp(-\|\mathbf{x} - \mathbf{x}^{(m)}\|_{l^2}/\sigma),$$

where the scaling parameter σ is set to the average l^2-norm of the snapshot data after centering it to have zero mean. The pseudospectra plot was computed using $N = 250$ observables. The trajectory data plotted in the figure in the paper (Supplementary Figure 13 in S.I.) correspond to the first five functions.

Supplementary References

[1] M. Aizenman and S. Warzel. *Random Operators*, volume 168 of *Graduate Studies in Mathematics*. American Mathematical Society, 2015.

[2] W. O. Amrein and V. Georgescu. On the characterization of bound states and scattering states in quantum mechanics. *Helvetica Physica Acta. Physica Theoretica. Societatis Physicae Helveticae Commentaria Publica*, 46(5):635–658, 1973.

[3] S. Arora and B. Barak. *Computational complexity: A modern approach*. Cambridge University Press, 2009.

[4] S. Bagheri. Koopman-mode decomposition of the cylinder wake. *Journal of Fluid Mechanics*, 726:596–623, June 2013.

[5] A. Bastounis, A. C. Hansen, and V. Vlačić. The extended Smale's 9th problem – On computational barriers and paradoxes in estimation, regularisation, computer-assisted proofs and learning. *arXiv preprint arXiv:2110.15734*, 2021.

[6] S. Becker and A. Hansen. Computing solutions of schrödinger equations on unbounded domains-on the brink of numerical algorithms. *arXiv preprint arXiv:2010.16347*, 2020.

[7] J. Ben-Artzi, M. J. Colbrook, A. C. Hansen, O. Nevanlinna, and M. Seidel. Computing spectra - On the solvability complexity index hierarchy and towers of algorithms. *arXiv*, 2020.

[8] G. Berkooz, P. Holmes, and J. L. Lumley. The proper orthogonal decomposition in the analysis of turbulent flows. *Annual Review of Fluid Mechanics*, 25(1):539–575, Jan. 1993.

[9] T. Berry, D. Giannakis, and J. Harlim. Nonparametric forecasting of low-dimensional dynamical systems. *Physical Review E*, 91(3):032915, Mar. 2015.

[10] E. Blanchard-Wrigglesworth, K. Armour, C. Bitz, and E. DeWeaver. Persistence and inherent predictability of Arctic sea ice in a GCM ensemble and observations. *J. Climate*, 24(1):231–250, 2011.

[11] L. Blum, F. Cucker, M. Shub, and S. Smale. *Complexity and Real Computation*. Springer New York, Secaucus, NJ, USA, 1998.

[12] A. Brown and M. Halperin. On certain area-preserving maps. *Annals of Mathematics*, pages 833–837, 1935.

[13] M. Budišić, R. Mohr, and I. Mezić. Applied Koopmanism. *Chaos: An Interdisciplinary Journal of Nonlinear Science*, 22(4):047510, Dec. 2012.

[14] R. E. Caflisch. Monte Carlo and quasi-Monte Carlo methods. *Acta Numerica*, 7:1–49, Jan. 1998.

[15] S. N. Chandler-Wilde, R. Chonchaiya, and M. Lindner. On spectral inclusion sets and computing the spectra and pseudospectra of bounded linear operators. *Journal of Spectral Theory*, 14(2):719–804, 2024.

[16] K. K. Chen, J. H. Tu, and C. W. Rowley. Variants of dynamic mode decomposition: Boundary condition, Koopman, and Fourier analyses. *Journal of Nonlinear Science*, 22(6):887–915, Apr. 2012.

[17] D. L. Cohn. *Measure theory*, volume 5. Springer, 2013.

[18] M. J. Colbrook. *The foundations of infinite-dimensional spectral computations*. PhD thesis, University of Cambridge, 2020.

[19] M. J. Colbrook. Computing spectral measures and spectral types. *Communications in Mathematical Physics*, 384(1):433–501, Apr. 2021.

[20] M. J. Colbrook. Computing semigroups with error control. *SIAM Journal on Numerical Analysis*, 60(1):396–422, 2022.

[21] M. J. Colbrook. On the computation of geometric features of spectra of linear operators on Hilbert spaces. *Foundations of Computational Mathematics*, pages 1–82, Dec. 2022.

[22] M. J. Colbrook. The mpEDMD algorithm for data-driven computations of measure-preserving dynamical systems. *SIAM Journal on Numerical Analysis*, 61(3):1585–1608, June 2023.

[23] M. J. Colbrook, V. Antun, and A. C. Hansen. The difficulty of computing stable and accurate neural networks: On the barriers of deep learning and Smale's 18th problem. *Proceedings of the National Academy of Sciences*, 119(12):e2107151119, 2022.

[24] M. J. Colbrook, L. J. Ayton, and M. Szőke. Residual dynamic mode decomposition: Robust and verified Koopmanism. *Journal of Fluid Mechanics*, 955:A21, Jan. 2023.

[25] M. J. Colbrook and A. C. Hansen. The foundations of spectral computations via the solvability complexity index hierarchy. *Journal of the European Mathematical Society*, 25(12):4639–4728, Nov. 2022.

[26] M. J. Colbrook, B. Roman, and A. C. Hansen. How to compute spectra with error control. *Physical Review Letters*, 122(25):250201, 2019.

[27] M. J. Colbrook and A. Townsend. Rigorous data-driven computation of spectral properties of Koopman operators for dynamical systems. *Communications on Pure and Applied Mathematics*, 77(1):221–283, July 2023.

[28] J. B. Conway. *A Course in Functional Analysis*, volume 96. Springer New York, 1985.

[29] S. Das, D. Giannakis, and J. Slawinska. Reproducing kernel Hilbert space compactification of unitary evolution groups. *Applied and Computational Harmonic Analysis*, 54:75–136, Sept. 2021.

[30] T. Eisner, B. Farkas, M. Haase, and R. Nagel. *Operator theoretic aspects of ergodic theory*, volume 272. Springer International Publishing, 2015.

[31] V. Enss. Asymptotic completeness for quantum mechanical potential scattering. I. Short range potentials. *Communications in Mathematical Physics*, 61(3):285–291, 1978.

[32] J. Fillman and D. C. Ong. Purely singular continuous spectrum for limit-periodic CMV operators with applications to quantum walks. *Journal of Functional Analysis*, 272(12):5107–5143, 2017.

[33] G. B. Folland. *Real Analysis: Modern Techniques and their Applications*, volume 40 of *Pure and applied mathematics*. Wiley, New York, 2. ed. edition, 1999.

[34] D. Giannakis and C. Valva. Consistent spectral approximation of Koopman operators using resolvent compactification. *Nonlinearity*, 37(7):075021, 2024.

[35] M. I. Gil'. *Operator Functions and Localization of Spectra*. Springer Berlin Heidelberg, 2003.

[36] A. C. Hansen. On the solvability complexity index, the n-pseudospectrum and approximations of spectra of operators. *Journal of the American Mathematical Society*, 24(1):81–124, July 2011.

[37] C. P. Jackson. A finite-element study of the onset of vortex shedding in flow past variously shaped bodies. *Journal of Fluid Mechanics*, 182(1):23–45, Sept. 1987.

[38] JAMSTEC. JAMSTEC OFES (Ocean General Circulation Model for the Earth Simulator) Dataset, 2009. `https://doi.org/10.17596/0002029`.

[39] T. Józsa, M. Szőke, T.-R. Teschner, L. Z. Könözsy, and I. Moulitsas. Validation and verification of a 2D lattice Boltzmann solver for incompressible fluid flow. In *Proceedings of the VII European Congress on Computational Methods in Applied Sciences and Engineering (ECCOMAS Congress 2016)*, ECCOMAS Congress 2016. Institute of Structural Analysis and Antiseismic Research School of Civil Engineering National Technical University of Athens (NTUA) Greece, 2016.

[40] E. Keogh, J. Lin, and A. Fu. Hot sax: Efficiently finding the most unusual time series subsequence. In *Fifth IEEE International Conference on Data Mining (ICDM'05)*, pages 8–pp. Ieee, 2005.

[41] B. O. Koopman. Hamiltonian systems and transformation in Hilbert space. *Proceedings of the National Academy of Sciences*, 17(5):315–318, May 1931.

[42] B. O. Koopman and J. von Neumann. Dynamical systems of continuous spectra. *Proceedings of the National Academy of Sciences*, 18(3):255–263, Mar. 1932.

[43] G. Levitt. Feuilletages des surfaces. In *Annales de l'institut Fourier*, volume 32, pages 179–217, 1982.

[44] E. N. Lorenz. Deterministic nonperiodic flow. *Journal of the Atmospheric Sciences*, 20(2):130–141, Mar. 1963.

[45] S. Luzzatto, I. Melbourne, and F. Paccaut. The Lorenz attractor is mixing. *Communications in Mathematical Physics*, 260(2):393–401, Aug. 2005.

[46] A. Mauroy and I. Mezić. Global stability analysis using the eigenfunctions of the Koopman operator. *IEEE Transactions on Automatic Control*, 61(11):3356–3369, Nov. 2016.

[47] A. Mauroy, I. Mezić, and J. Moehlis. Isostables, isochrons, and Koopman spectrum for the action–angle representation of stable fixed point dynamics. *Physica D: Nonlinear Phenomena*, 261:19–30, Oct. 2013.

[48] I. Mezić. *On the geometrical and statistical properties of dynamical systems: theory and applications*. PhD thesis, 1994.

[49] I. Mezić. On applications of the spectral theory of the Koopman operator in dynamical systems and control theory. In *2015 54th IEEE Conference on Decision and Control (CDC)*, pages 7034–7041. IEEE, Dec. 2015.

[50] I. Mezić. Spectrum of the Koopman operator, spectral expansions in functional spaces, and state-space geometry. *Journal of Nonlinear Science*, 30(5):2091–2145, Dec. 2020.

[51] I. Mezić. Koopman operator, geometry, and learning of dynamical systems. *Notices of the American Mathematical Society*, 68(07):1, Aug. 2021.

[52] I. Mezić. On numerical approximations of the Koopman operator. *Mathematics*, 10(7):1180, Apr. 2022.

[53] B. S. Nagy, C. Foias, H. Bercovici, and L. Kérchy. *Harmonic Analysis of Operators on Hilbert Space*. Springer Science & Business Media, 2010.

[54] B. R. Noack, K. Afanasiev, M. Morzynski, G. Tadmor, and F. Thiele. A hierarchy of low-dimensional models for the transient and post-transient cylinder wake. *Journal of Fluid Mechanics*, 497:335–363, Dec. 2003.

[55] B. R. Noack and H. Eckelmann. A global stability analysis of the steady and periodic cylinder wake. *Journal of Fluid Mechanics*, 270:297–330, July 1994.

[56] M. O. Williams, C. W. Rowley, and I. G. Kevrekidis. A kernel-based method for data-driven Koopman spectral analysis. *Journal of Computational Dynamics*, 2(2):247–265, 2015.

[57] S. Y. Pilyugin. *Shadowing in Dynamical Systems.* Number v.1706 in Lecture Notes in Mathematics Ser. Springer Berlin / Heidelberg, Berlin, Heidelberg, 1999.

[58] M. Richter and T. Schreiber. Phase space embedding of electrocardiograms. *Physical Review E*, 58(5):6392, 1998.

[59] O. E. Rössler. An equation for continuous chaos. *Physics Letters A*, 57(5):397–398, July 1976.

[60] C. W. Rowley and S. T. M. Dawson. Model reduction for flow analysis and control. *Annual Review of Fluid Mechanics*, 49(1):387–417, Jan. 2017.

[61] D. Ruelle. A remark on bound states in potential-scattering theory. *Il Nuovo Cimento A*, 61(4):655–662, 1969.

[62] T. Schreiber and D. T. Kaplan. Nonlinear noise reduction for electrocardiograms. *Chaos: An Interdisciplinary Journal of Nonlinear Science*, 6(1):87–92, 1996.

[63] M. Szőke, T. I. Jozsa, Á. Koleszár, I. Moulitsas, and L. Könözsy. Performance evaluation of a two-dimensional lattice Boltzmann solver using CUDA and PGAS UPC based parallelisation. *ACM Transactions on Mathematical Software*, 44(1):1–22, July 2017.

[64] K. Taira, M. S. Hemati, S. L. Brunton, Y. Sun, K. Duraisamy, S. Bagheri, S. T. M. Dawson, and C.-A. Yeh. Modal analysis of fluid flows: Applications and outlook. *AIAA Journal*, 58(3):998–1022, Mar. 2020.

[65] W. Tucker. A rigorous ODE solver and Smale's 14th problem. *Foundations of Computational Mathematics*, 2(1):53–117, Jan 2002.

[66] A. M. Turing. On computable numbers, with an application to the Entscheidungsproblem. *Proceedings of the London Mathematical Society*, s2-42(1):230–265, 1937.

[67] A. Zebib. Stability of viscous flow past a circular cylinder. *Journal of Engineering Mathematics*, 21(2):155–165, 1987.
