# Supplementary material for: Adversarial dynamical systems characterize when data-driven learning succeeds or fails
Source: Nat Commun. 2026 Jul 14;17:5397. doi: 10.1038/s41467-026-74220-8 (PMC13369498; doi:10.1038/s41467-026-74220-8)
Supplement: Supplementary file 2 — Transparent Peer Review file [file 41467_2026_74220_MOESM2_ESM.pdf]

nature portfolio

Peer Review File

Adversarial dynamical systems characterize when data-driven learning succeeds or fails

Corresponding Author: Dr Matthew Colbrook

This file contains all reviewer reports in order by version, followed by all author rebuttals in order by version.

Attachments originally included by the reviewers as part of their assessment can be found at the end of this file.

Version 0:

Reviewer comments:

Reviewer #1

(Remarks to the Author)
Please see attached PDF.

(Remarks on code availability)

Reviewer #2

(Remarks to the Author)
My overall feeling is that the authors have tried to merge a mathematics paper and a methods paper into a format for Nature Communications and the result is less than the sum of the parts. There are many claims that I would say are on the "over-the-top" side, and that are not substantiated upon reading the manuscript. It is often unclear what the new results are. Theory is mostly vaguely stated with little detail (in some cases, without citation) and is not well-linked to specific theorems in the supplementary material. The methods section is very brief, to the point where it would be impossible to reproduce some of the results. The comparison with EDMD could be made much clearer and convincing with greater detail. The sea-ice example lacked detail in the methods section and in the climate interpretation.

In summary, the manuscript was long and (to me) confusing. I struggled to learn much from the manuscript. If the mathematics is new, it would be better to publish it in a specialist journal for an audience that can understand the mathematics. Similarly, the applied components need much greater detail on the methods and interpretation and could also be submitted to a specialist dynamics journal. I do not recommend publication in Nature Communications.

Detailed points:

0. Figure 1 serves no scientific purpose because it contains 16 complicated, small subimages, which are not understandable from the caption.

1. In the abstract an "optimal algorithm" is mentioned but I could not find it. This algorithm should "overcome key limitations of standard methods", but it was not clear to me what these key limitations were or how a presented algorithm overcame them.

2. The abstract and p2 claims "first general guarantees of convergence". Perhaps the devil is in the word "general", but I think this is a misleading statement given the substantial work on eigenvector convergence of operators.

3. The abstract states that the sea-ice example "illustrates how AI models using Koopman operators can deliver superior performance at a fraction of the computational cost". I could not find anything in the manuscript illustrating this.

4. Figure 2(c) is supposed to show that the "new method converges". It is unclear what the error is measuring, and the y-axis range for the error for the "new method" did not dip below 0.01. Similar to figure 1, most panels were not able to be comprehended from the caption.

5. The claim on p3 that "our work contributes to understanding the challenge of hallucination in LLMs" lacks evidence.

6. The arctic sea ice example was mostly unclear. Figure 3 has no color scale and I have no idea what the colors mean in the spatial plots. If these are Koopman modes, aren't they complex? How are the complex modes represented in the figures, and how are complex modes linked with ice melt? The same questions exist for Figure 7. On p7 it was stated that decaying (complex) modes "reflect dissipative dynamics, i.e. sea ice melting", but no reasoning was provided for this claim.

7. The italic result on the second column of p8 is not particularly informative. For example, (i) the cause of the failure is not described, (ii) examples of failure or indications of the size of the class of maps that fail are missing. If the cause is exotic, or the class of failures is tiny, this is not a particularly interesting result.

8. Similarly, also on p8 "...there is no universal rule for determining N....that guarantees convergence... these limits are combined". Presumably this means there is some 'bad class', which is not identified here.

9. The final paragraph prior to the section heading "Classifications of learning" on p9: the paragraph sounds interesting, but is so vague it cannot be understood (even though the paragraph claims the necessity of using two limits is "intuitive").

10. p11, 4 paragraphs prior to Discussion heading: this is supposed to "establish" and "illustrate" a result about needing three successive limits, but what follows is a brief set of statements with no reasoning (or illustration).

11. The Discussion opens with "We have developed a powerful technique...", but no technique has been presented yet. Similarly, in the Discussion: "we designed new optimal algorithms", "introduced multi-data-limit algorithms", "This work represents a rare instance in machine learning...". None of these items have been defined in the manuscript so far.

12. On p13, an algorithm is described, but it is not clear if this is a new algorithm appearing for the first time in this manuscript or not. The lack of citation strongly suggests it must be completely new, but in that case, the authors should introduce it in far more detail. I note also that as a data-driven method, this approach seems to require 3 limits, which the authors railed against toward the beginning of the manuscript.

13. The experimental details on pp13 and 14 for three complicated dynamical systems were inadequate. If this is a new approach, one could spend an entire paper analyzing it and describing strengths/weaknesses through detailed and thorough examples. The Duffing example did not explain (this is not a complete list): the choice of trajectory length 5, why k-means clustering was used, the value of L. The EDMD description, which is meant to be the main comparator, was confusing. I could not find an algorithm in SuppSection2.1 as claimed.
Fluid flow example: why is Fig 11 interesting? Why is the pure point spectrum in Fig 2 interesting?
Sea ice example: the whole example is lacking in detail. Details of embedding are lacking.
How do you know your embedding is good?

(Remarks on code availability)

Version 1:

Reviewer comments:

Reviewer #1

(Remarks to the Author)
Please see attached PDF.

(Remarks on code availability)

Version 2:

Reviewer comments:

Reviewer #1

(Remarks to the Author)
The authors have addressed the remaining review comments.

I have no further comments and I recommend publication of the manuscript in its current version.

(Remarks on code availability)

Open Access This Peer Review File is licensed under a Creative Commons Attribution 4.0 International License, which permits use, sharing, adaptation, distribution and reproduction in any medium or format, as long as you give appropriate credit to the original author(s) and the source, provide a link to the Creative Commons license, and indicate if changes were made.
In cases where reviewers are anonymous, credit should be given to 'Anonymous Referee' and the source.
The images or other third party material in this Peer Review File are included in the article’s Creative Commons license, unless indicated otherwise in a credit line to the material. If material is not included in the article’s Creative Commons license and your intended use is not permitted by statutory regulation or exceeds the permitted use, you will need to obtain permission directly from the copyright holder.
To view a copy of this license, visit https://creativecommons.org/licenses/by/4.0/

Global comments for both referees

We are grateful to all the referees for their thoughtful and constructive feedback, which has led to substantial improvements in both the clarity and content of the manuscript. We enclose our detailed responses to each comment, alongside the revised manuscript and Supplementary Information.

Changes in the paper are marked in red. All other changes consist of grammatical and spelling corrections, as well as adjustments to wording for simplification, improved clarity, and economy. Notably, we have added a new figure (Figure 9) showcasing spectral analyses of a wide range of analytical and real-world dynamical systems using our proposed algorithms, following Referee 2's comments.

We would like to highlight several major updates made in response to Referee 2's general comments (with further detail provided in the response document):

- The manuscript has been restructured to better integrate theory, methods, and applications. To support this revised format, we have included in the response a list of recent *Nature Communications* articles on data-driven dynamical systems that integrate mathematics, methods, and applications, and adopt a similar multidisciplinary structure and style to our revised paper.
- In the revised manuscript, we have carefully reduced jargon and kept the main text accessible without requiring advanced mathematics. The only mathematical concepts now involved are eigenvalues and eigenfunctions, separation of variables, limits, and functions—all of which are standard in undergraduate physical science curricula and are briefly explained where they appear.
- All major claims are now clearly stated and substantiated. The main theoretical results are explicitly explained and directly linked to corresponding theorems in the Supplementary Information, while written in the paper in a manner suitable for a general scientific audience. The algorithms are also linked to corresponding pseudocode in the Supplementary Information.
- The Methods section—including the sea ice example—has been much expanded and now provides comprehensive detail on all aspects of implementation. We believe that it is now more in line with *Nature Communications*'s standards, which we view as a strength of the revised manuscript.
- Our discussion of EDMD has been clarified (including the explicit matrix it constructs), and we now include a bullet-point summary of the climate-related interpretations below in our response. The applied components have been revised to include clearer interpretation and more complete methodological detail.

In addition, all of this has been done whilst making the paper shorter and more focused. We sincerely thank the referee for these suggestions, which have helped us improve the manuscript.

Response to Referee 1

Comments and suggestions made by the referee are addressed below.
(*referee comments in italics*, our response in normal font, *quotes from revised paper in red*)

This paper presents a study on algorithmic barriers to data-driven approximation (learning) of dynamical systems. The focus is on learning via spectral approximation of Koopman operators on L^p spaces. Building on a program developed by the authors on the Solvability Complexity Index (SCI), a number of theoretical results are derived on the impossibility of approximating the spectrum of the Koopman operator for a class of dynamical systems using a single sequence of algorithms, as well as learnability via multi-parameter families of algorithms using iterated limits. Based on these results, an SCI index is introduced characterizing the efficiency of a given family of algorithms in terms of the number of iterated limits it requires (compared to the minimum bound for the given system class). The paper also includes a suite of numerical examples involving the Duffing oscillator, a cavity flow, and Arctic sea ice dynamics.

In my assessment this is an interesting and well-written paper that represents a significant contribution in the field of data-driven approximation of dynamical systems that is suitable for publication in Nature Communications. I do believe, however, that aspects of the manuscript, particularly aspects related to its positioning in the broader context of learning methodologies, would benefit from some adjustment/clarification. I also have some comments on the sea ice application.

We thank the referee for their thoughtful comments, which have been very helpful in improving the clarity and quality of our manuscript, as well as providing further climate interpretations of the sea ice example. We greatly appreciate the time and care taken in reviewing our work. In what follows, we have addressed each of the referee's points in detail and have made corresponding revisions to the manuscript where appropriate.

Before describing my comments in detail, I will note that there is an arXiv preprint by the authors entitled "Limits and powers of Koopman learning" (arXiv:2407.06312) that appears to be closely related to this manuscript but is not cited in the bibliography. If the preprint is being considered as a peer-reviewed journal article or proceedings paper it should be cited and its relationship with the present manuscript should be commented on.

The mentioned arXiv preprint is indeed an earlier version of the current manuscript. We posted it in advance of a seminar presentation in order to have a stable reference for attendees. That preprint has not been, and will not be, submitted as a separate article to any peer-reviewed journal or proceedings. Rather, it served as a preliminary version of the present work and has since been substantially revised and expanded into the manuscript under review. When revisions are complete, we will update the arXiv version accordingly

Comments on abstract and sections until "Three examples"

1. *In my initial reading of the abstract and first few paragraphs, my impression had been that the paper would address general learning techniques. I am referring to statements such as "reliable learning is impossible, regardless of algorithm choice" in the abstract and "first examples of problems in data-driven dynamical systems that no sequence of algorithms, probabilistic or otherwise, can solve" in the second paragraph of the right-hand column of p. 1. However, later on it becomes clear that the focus is on learning with Koopman/transfer operator techniques, and in particular methods that utilize the spectrum of the Koopman operator in L^2 spaces. On the face of it this is a significantly narrower class of algorithms that the wording mentioned above suggests. Is it obvious that the impossibility results for algorithms utilizing approximation of the Koopman spectrum spectrum in L^2 extend to arbitrary algorithms (again, as the wording suggests)? If this is true, I would suggest adding an explanation in a prominent location in the manuscript. Either way, I believe that it is important to clarify in the abstract and early on in the main text that the bulk of the analysis will be on the specific class of Koopman spectral approximation techniques, even if the scope of the results can be extended further.*

   We thank the referee for pointing out how we can improve the clarity of the abstract. The "first examples" we refer to are indeed the first impossibility results established in the broader context of data-driven dynamical systems, here illustrated through the spectral approximation of Koopman operators. Furthermore, the broader framework we introduce—namely, the construction of adversarial

dynamical systems that obstruct learning and the SCI—has wider applicability for general learning techniques. In particular, it can be adapted to other classes of data-driven learning problems, including PDE learning (for example, the third author is writing a manuscript applied to PINNs). Indeed, the first author currently has a PhD student working on extending these ideas to hyperbolic PDEs, where similar impossibility results are being proved. We briefly mention this direction in the Discussion section (see the example quotation below).

Regarding the statement "reliable learning is impossible, regardless of algorithm choice," the impossibility results we prove—for the problem of computing spectral properties of Koopman operators—hold for any type of algorithm. This follows from our use of general algorithms in the proofs, which encompass all computational procedures. It is important to emphasize that once the computational problem is fixed, the impossibility results apply universally, independent of the algorithm used.

While we had indicated on page two that our analysis focuses on Koopman operators and their spectral approximation as an example, we now realise that this may come too late. To address this, we have revised both the abstract and the opening section of the introduction (see red text on page 1), emphasising that (though the framework of upper and lower bounds (in particular, the method of adversarial dynamical systems) is general) the main example of our paper is on spectral methods for Koopman and transfer operators. We have also moved the discussion of further methods to the Discussion section, for example, we write *"Our approach extends beyond Koopman operators, provided appropriate domain-specific "sudden change" lemmas can be established (see Figure 11). In each case, an analog of the "ball of learnability" and the classifications in Figures 7 and 8 applies. This perspective is relevant to a range of methods—including SINDy [10], neural ODEs [89], Fourier neural operators [90], LSTMs [91], and PDE-net [92]—as well as to other areas of scientific computing with ML. For example, recent work has shown that linear elliptic PDEs can be learned from input-output pairs [93], analogous to snapshot-based learning. Whether similar approaches extend to hyperbolic or nonlinear PDEs remains open, but our proof techniques may offer insight into this challenge."*

2. *The second-to-last paragraph on p. 2 mentions that the paper introduces the "first general- purpose, convergent methods for Koopman learning". The first paragraph caption of Figure 2 also refers to a "new method" without providing an explicit reference. I am sorry if I missed this somewhere in the details, but what is the new method introduced in this paper? Is this related to the RAGE theorem discussed in the Methods section? As far as I could tell the numerical methods make significant use of approaches previously developed by the authors (e.g., residual methods involving $\mathcal{K}_F^*\mathcal{K}_F$). In my opinion the new aspects of the Koopman approximation methodology introduced in the paper should be more strongly emphasized. A pseudocode that can be referenced (e.g., in Figure 2) would also be helpful.*

   Thank you for the helpful suggestion of pseudocode and pointing out how we can make the connections with algorithms stronger. The new methods we refer to are developed in the convergence proofs (Supplementary Information). These methods establish the first general-purpose, provably convergent algorithms for Koopman spectral learning in the presence of continuous spectra. While the analysis builds on tools we have developed, such as $\mathcal{K}_F^*\mathcal{K}_F$, and makes use of the RAGE theorem (a well-known result in mathematical physics) to handle spectral delocalisation, the overall methodology and convergence framework introduced here are new.

   For example, our Σ_1 algorithm for spectra (Algorithm 1 in the Supplementary Information) uses an adaptive procedure to search for local minimisers of estimates of $\sigma_{\mathrm{inf}}(\mathcal{K}-zI)$. In contrast, previous residual methods approximate pseudospectra. The only previously known algorithm that we use was an early version of Algorithm 2, which has since been significantly generalised and now incorporates adaptive quadrature procedures. We also point out in the Methods section that the algorithms based on the RAGE theorem are completely different to other techniques that partition the full spectral measure using ergodicity (we do not need to assume ergodicity for our algorithms).

   To improve clarity, we have added a dedicated section to the Supplementary Information that includes pseudocode for the algorithms, making the contribution easier to understand and reference. We have also expanded the Methods section accordingly and now explicitly refer to the pseudocode in the caption of Figure 2 (now Figure 1), as you suggested.

3. *Figure 2: What is being plotted in the Error axis in panel (c)? Please update the label and/or include*

the information in the caption.

We thank the referee for pointing out how to make Figure 2 clearer. In panel (c) of Figure 2 (now Figure 1), we plot the maximum of (an approximation of) $\sigma_{\mathrm{inf}}(\mathcal{K} - zI)$ (where σ_{inf} denotes the smallest singular value, also called injection modulus) over the output as a measure of error, averaged over 10 independent realisations of the experiment (since the snapshot data are sampled randomly). This quantity reflects how close the computed spectrum is to the true spectrum of the Koopman operator. Our approximation of the function $z \mapsto \sigma_{\mathrm{inf}}(\mathcal{K} - zI)$ converges (from above) uniformly on compact subsets of $\mathbb{C}$ as the matrix size increases.

In the Hamiltonian setting, we have the identity $\sigma_{\mathrm{inf}}(\mathcal{K} - zI) = \mathrm{dist}(z, \mathrm{Spap}(\mathcal{K}))$, so this error is exact. In the dissipative case, the inequality $\sigma_{\mathrm{inf}}(\mathcal{K} - zI) \leq \mathrm{dist}(z, \mathrm{Sp}_{\mathrm{ap}}(\mathcal{K}))$ holds, and while this may underestimate the true distance, it still allows us to detect non-convergence—for instance, the plateau in the EDMD error curve remains indicative of failure to recover the spectrum. Moreover, one can show that $\mathrm{dist}(z, \mathrm{Sp}_{\mathrm{ap}}(\mathcal{K})) = \mathcal{O}(\sigma_{\mathrm{inf}}(\mathcal{K} - zI))$ in this case, so convergence of the plotted error to zero for the new method provides rigorous evidence of spectral convergence.

In response to the referee's comment, we have now clarified the vertical axis label in Figure 2(c) (now Figure 1(c)), updated the figure caption to explain this choice of error metric, and expanded upon the interpretation in the Methods section to guide the reader more clearly. The same error metric is also included in Figure 9 of the main text, which shows similar results for a range of dynamical systems.

4. *The statement in the top-right of p. 6 that "many practitioners simply believe in the limitless power of such approaches" seems overly speculative/subjective. I suggest rewording that sentence.*

   We agree that the original phrasing was too subjective and have revised the sentence accordingly. The updated version avoids subjective language while retaining the intended message, namely that these methods are often applied under broad assumptions that may not always be justified. We hope this resolves the concern.

Comments on the sea ice application

1. *It has been known through work dating back at least to the early 2010s [1] that Arctic sea ice exhibits a seasonally-modulated reemergence of correlations, whereby sea ice anomalies occurring during the growth season (in the fall) reemerge in the following melt season (spring) despite a loss of correlation in the intervening winter months. This phenomenon, as well as a related melt-to- growth reemergence was originally revealed using conventional correlation techniques and it was physically interpreted through various mechanisms involving the dynamic/thermodynamic coupling between sea ice, the upper ocean, and atmosphere. In [3] (and earlier papers referenced therein) we studied sea ice reemergence using a kernel eigendecomposition technique (NLSA) that in many ways behaves similarly to Singular Spectrum Analysis or Hankel DMD. A finding of this work was the correlation structure of sea ice reemergence can be reconstructed using low-dimensional families of modes consisting of a low-frequency (interannual to decadal) mode as well as pairs of modes given by products between the low-frequency mode and the harmonics of the annual cycle. See, e.g., Fig. 2 in [4] for representative time series that broadly correspond to real and imaginary parts of Koopman eigenfunctions obtained through DMD-type methods. I suspect that the "hidden" decaying sea ice modes shown in Figure 3 of this paper are related to those seasonally modulated patterns. This is both because of the fact that that the family of blue dots in the spectrum of Figure 3 consists of a single eigenfunction of zero/low-frequency together with its corresponding annual and semiannual modulated pairs, as well as the spatial structure of the SIC anomalies (featuring, e.g., anomalies of opposite sign in the Bering and Beaufort seas) which is characteristic of the aforementioned sea ice reemergence behavior. A way to verify this would be to (i) compare the moduli of eigenfunction time series in the "hidden" family and test whether the modulus of the zero-frequency eigenfunction in that family correlates significantly with the moduli of the other eigenfunctions in the family; (ii) compute time-correlation functions of reconstructed sea ice concentration/extent based on Koopman eigenfunctions and modes, and look for characteristic reemergence "branches" in the correlation structure (e.g., Figs. 1, 2 in [3]). Either way, I suggest commenting on previous work related to sea ice reemergence as it appears to be at least potentially related. I would also suggest to adjust text such as "undetected by conventional approaches" in the abstract,*

unless there is significant evidence that the hidden modes in Figure 3 are not related to reemergence. If this turns out to be the case the authors may wish to investigate whether sea ice reemergence modes are present deeper in the spectrum since they are a recognized source of coherent sea ice variability.

Thank you for suggesting to look at this phenomenon – it has lead to new interesting physical interpretations in our Arctic sea ice example and interesting connections with the wider literature. If we compare, as suggested by the referee in (i), the moduli of eigenfunction time series in the "hidden" family and test whether the modulus of the zero-frequency eigenfunction in that family correlates significantly with the moduli of the other eigenfunctions in the family, we obtain canonical correlations along the time series of 0.9807 and 0.9798 for the modes with argument $\pi/6$ and $\pi/3$, respectively.

Below we show the time-correlation functions of reconstructed sea ice concentration/extent based on Koopman eigenfunctions and modes:

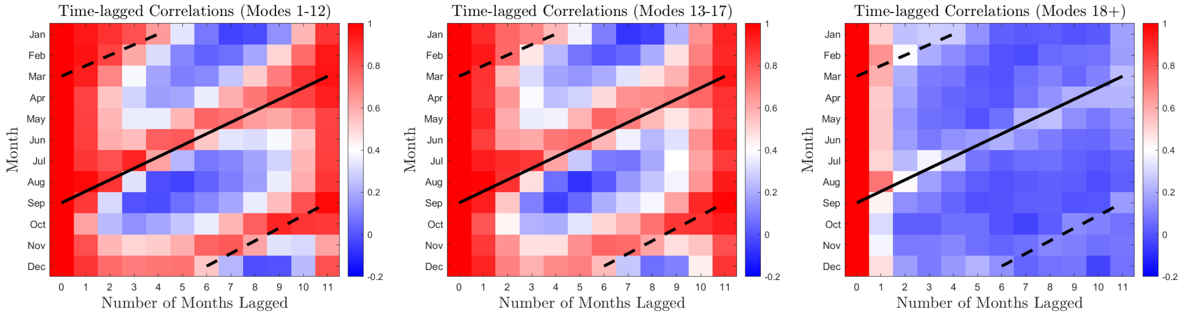

The modes in these figures are ordered by their errors. So the leftmost corresponds to the annual variation, the middle the five hidden modes, and the rightmost the remaining modes. The annual variation and decaying modes do indeed seem to capture reemergence.

We wish to point out that, without the error bounds, this pattern would be hard to spot behind a wall of spurious modes. Moreover, it is not immediately clear to us how to directly link the dissipative modes we have found to those suggested by the referee. In the papers on sea ice reemergence discussed by the referee, there is a focus on families of modes associated with patterns such as the North Pacific Gyre Oscillation that are studied systematically in both climate models (with no climate change) and observations. In contrast (see our reply to point 3 below), there is strong evidence that the modes we have found are associated with climate change trends. Hence, we have discussed sea ice reemergence as a related phenomenon (with additional figures in the Supplementary Information) and adjusted the text in the abstract accordingly. We believe pursuing this link further would be of great interest in future work, but an extensive study is beyond the scope of the current manuscript.

2. *In the climate science literature, such seasonally modulated product modes are sometimes referred to as "combination modes" [8], and are found in different contexts such as the El Niño Southern Oscillation and Antarctic sea ice variability; see e.g., the generator spectrum in Fig. 3 of [6] where a number of such families can be found.*

   Thank you for providing other examples of the multiplicative nature of the Koopman spectrum in climatology. We have now discussed this (and referenced the papers) when talking about the hidden family.

3. *In the second paragraph of p. 7 the decay constant $-1/\log|\lambda|$ of the hidden modes is connected with decay in the Antarctic region (I am referring specifically to the phrase "consistent with decay times observed in the Antarctic region"). Did you mean Arctic here as opposed to Antarctic?*

   We meant Antarctic, and are referring here to a decay that was not observed in the Arctic in the cited study, but was observed in the Antarctic, owing to the use of DMD (as opposed to EDMD). We have now made this clearer.

   And what does "decay" refer to in this context; is it decline of sea ice extent? If so, I couldn't see how the decay constant based on λ (which measures decay of correlations of observables under the approximate Koopman operator) is directly connected to characteristic timescales of sea ice loss. Moreover, I don't

believe that concentration of anomalies in the Barents and Kara Seas is indicative of sea ice reduction in and of itself—these regions have significant natural variability due to the presence of marginal ice zones. For a more definitive test, I would suggest examining the corresponding Koopman eigenfunction time series—if that time series has an appreciable trend component over the analysis interval this would provide significantly stronger evidence of a connection with sea ice loss due to climate change. In that case, the hidden modes with nonzero $\arg(\lambda)$ *could be interpretable as modulations of the seasonal cycle by the trend (see Fig. 8 in [7] for an example involving precipitation). However, it is entirely possible that the zero-frequency mode in the hidden family is related to natural low-frequency climate patterns rather than trends. Either scenario would be interesting in my opinion.*

Thank you for this point, which has allowed us to relate these modes with sea ice loss and also clarify a query of the other referee. Following the referee's suggestion, we have computed the Koopman eigenfunction times series (below we only show those with non-negative arguments due to complex-conjugate symmetry, absolute value on the left and real part on the right with the expected periods of oscillation from λ):

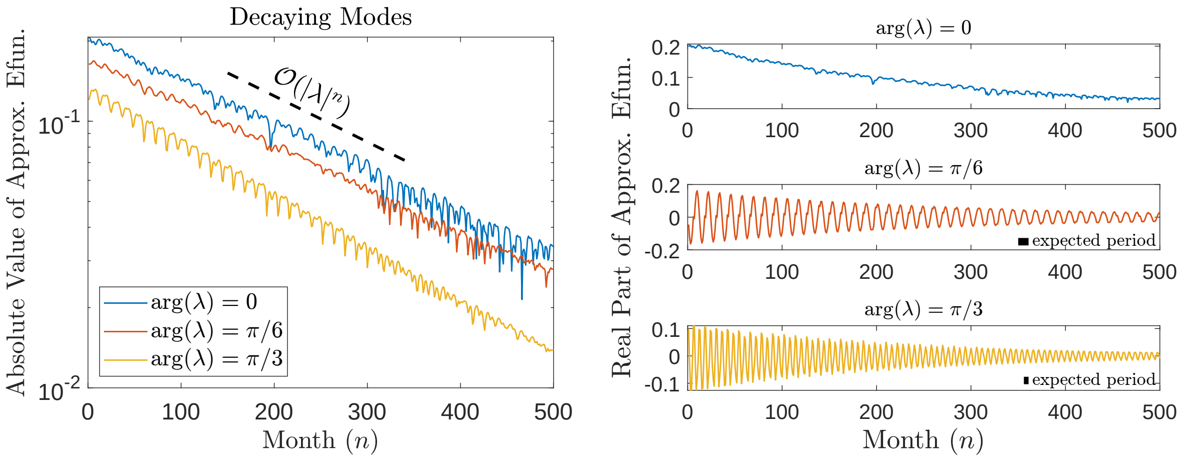

There is a trend consistent with the eigenvalue and the expected modulation. We have now discussed this point in the paper (and added this plot to the Supplementary Information), including the interpretation as modulations of the seasonal cycle by the trend. We have also made the meaning of "decay" much clearer throughout the manuscript.

4. *Figure 4 and related text:*

   (a) *Could you provide the definition of "entire sea ice concentration"? Is this the average sea ice concentration (a scalar between 0 and 1) summed or averaged over all gridpoints? If so I wonder why the authors made that choice of observable instead of sea ice extent (which is shown in Figure 7). As far as I know, sea ice extent is a standard choice for pan-Arctic sea ice forecasting.*

   This is a good point and has allowed us to make it clearer that we are considering a harder forecasting test. We meant the entire dataset—specifically, the reconstruction of sea ice concentration at every grid point. So there is no summation or average over the gridpoints. We have now clarified this in the text. We chose to focus on this observable (rather than sea ice extent) because reconstructing the full spatial field is a more challenging task, and thus provides a stricter test of the methods.

   (b) *How is the relative mean squared error computed? Specifically, what is the normalization factor used to compute relative errors; e.g., is it based on the standard deviation of the full sea ice concentration or the standard deviation of anomalies relative to monthly climatology (average of all Januaries, Februaries, etc. over a reference period such as the training period)? The latter would be more appropriate since the seasonal cycle is a dominant, but trivially predictable, component of the signal. If predicting sea ice extent, the authors could consider reporting un-normalized RMSEs in units of area.*

We have now provided a formula for the error metric. Following the referee's suggestion (see point (c) below as well), we have measured the reconstruction error of the anomalies.

(c) *The figure should include forecast errors by simple baseline models for comparison. Two standard choices are the persistence model and a periodic model that predicts by monthly climatology. More elaborate models are oftentimes assessed through the additional forecast skill they provide beyond these baseline approaches; e.g. [2].*

We tested both methods and found that the periodic model considerably outperformed the persistence model. Consequently, we have used the periodic model as the baseline for comparison. This has also been done for the binary accuracy figure.

(d) *I am concerned that the 4-year verification interval (2016–2020) is too short to draw statisti- cally robust conclusions about forecast skill given various sources of interannual and decadal climate variability. Since there are data available from 1979–2021 why not use the first half for training and the second half for verification? Another option might be to use the same verification interval (2012–2020) as in Figure 5. In this way the results in Example 1.3 would be organized around two Koopman models (one for the spectral computations in Figure 3 and another one for forecasting) as opposed to three different models in the current setup.*

Thank you for this suggestion - it has allowed us to demonstrate statistical robustness of our results. We agree with the concern that a short verification interval may not be sufficient for this plot, especially in the presence of interannual and decadal climate variability. In response, we have revised the figure and now use the error metric suggested earlier by the referee. To address the concern about sample size and robustness, we have expanded the verification period considerably.

Specifically, we now consider forecasts initialized at every month from 2005 to 2010, each with a 10-year forecast horizon. In each case, we use data from 1979 onwards as the training data. This gives a total of 72 distinct forecast trajectories, allowing us to average errors across different initial conditions and seasons. This change provides a significantly larger basis for evaluating forecast performance and enables more statistically meaningful comparisons between methods.

5. *In general, do the forecast models utilizing shorter training intervals have Koopman spectra of similar structure/quality to the spectrum depicted in Figure 3 (e.g., in terms of eigenvalue error)? Do these models also have hidden decaying sea ice modes? Since spectral approximation is placed at the center-stage of learning dynamical systems it would be helpful to include that information somewhere; e.g., include plots of representative spectra in the Supplementary Information.*

The corresponding spectra and errors are indeed similar. Though, the exact location of the hidden modes varies and the error metric is typically worse for smaller sized training data (as expected). Here is the spectrum for data from 2012-2021 (where we perform the binary accuracy experiment):

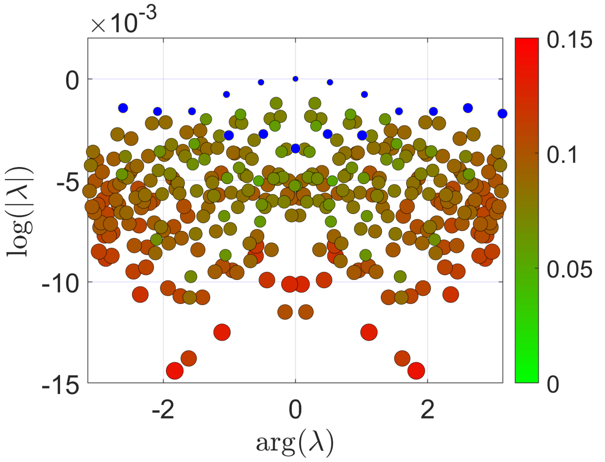

We have now discussed this in the Supplementary Information.

6. *I couldn't find information about the number of time-delays used in the various sea ice experiments. Please include that or highlight it more strongly if already present. Also, please confirm that in the prediction experiments delay-embedding is always "backward"? In other words the initial conditions do not contain information from the future.*

   We have now highlighted this more strongly (6 delays to to help capture semiannual patterns - we also found other choices ≥ 3 up to about 60 (5 years) worked well). We also confirm that, in all prediction experiments, the delay embeddings are constructed in a strictly backward manner. That is, the delay-coordinate vectors used for forecasting are built from past observations only; no future information is used in either the construction of the embeddings or in the initial conditions provided to the forecasting models. This ensures that the evaluation is entirely out-of-sample and causal, in line with standard practice in forecasting and time series analysis. We have made the description in the Methods section of delay embedding clearer accordingly.

7. *Similarly to comment 4(c), it would be useful to include results from persistence and/or climatology forecasts in Figure 5. It's not obvious what is the binary accuracy that one can expect from simple models.*

   Thank you for this helpful suggestion to compare against further benchmarks. As noted in our response above, we have now carried out a comparison with both the persistence and periodic models. Across all error metrics considered—including binary accuracy—the periodic model consistently outperformed the persistence model, often by a significant margin. For this reason, we have chosen to include only the periodic model in Figure 5 as a representative simple baseline.

Comments on Results section

1. *As mentioned above, the bulk of the analysis in this section is on learning algorithms for dynamical systems that involve approximating the approximate spectrum (no pun intended) $\mathrm{Sp}_{\mathrm{ap}}(\mathcal{K}_F)$ of the Koopman operator on L^2 spaces. A general question I had is to what extent are the barriers to learning derived for this class of algorithms representative of general learning algorithms, including operator-theoretic algorithms that do not perform spectral computations, or even nonlinear approximation algorithms that do not employ Koopman/transfer operators?*

   This is a great question. Spectral computations are typically harder than, for example, obtaining approximations that converge in the strong operator topology. Hence, we expect that they sit lower in the SCI than spectral computations. Table 1 in the paper includes a few upper bounds for the action of $\mathcal{K}$ on observables (strong operator convergence). However, we do not have any corresponding lower bounds. We fully expect that the adversarial dynamical systems approach will extend to this scenario (see also this paper: https://onlinelibrary.wiley.com/doi/abs/10.1002/nla.2531 that looks at impossibility result in the context of queries of finite matrices, for which it may be possible to combine with our techniques). We have now mentioned this in the Discussion section of the paper.

   For a variety of prediction applications, learning the Koopman operator in the strong operator topology (or even weak topology) without approximating its spectrum seems adequate, is that not the case? I am thinking of basic examples—for instance consider the class of continuous maps $F : X \to X$ on closed, smooth submanifolds X of Euclidean space $\mathbb{R}^d$ for any dimension d. There exist various graph-theoretic algorithms for approximation of heat operators (e.g., [5]) such that given i.i.d. samples $x_1, \ldots, x_M \in X$ drawn from an arbitrary fully supported probability measure μ on X with smooth density relative to the Riemannian volume measure, they produce one-parameter families of kernel functions $p_M \in C(X \times X)$ (oftentimes, by normalization of Gaussian kernels on $\mathbb{R}^d$ with a bandwidth parameter ϵ_M decreasing to 0 as $M \to \infty$ at an appropriate rate) whose corresponding integral operators $P_M : C(X) \to C(X)$,

$$P_M f = \int_X p_M(\cdot, x) f(x) d\mu_M(x) \equiv \frac{1}{M} \sum_{m=1}^{M} p_M(\cdot, x_m) f(x_m),$$

   converge strongly to the identity as $M \to \infty$. (Here, $\mu_M = \frac{1}{M}\sum_{m=1}^{M} \delta_{x_m}$ is the empirical sampling

measure.) Define $\mathcal{K}_{F,M} : C(X) \to \infty C(X)$ *as* $\mathcal{K}_{F,M} = P_M \mathcal{K}_F$, *where*

$$\mathcal{K}_{F,M} f = \frac{1}{M} \sum_{m=1}^{M} p_M(\cdot, x_m) f(x_{m+1})$$

Then, as $M \to \infty$, $\mathcal{K}_{F,M}$ *converges strongly to the Koopman operator* $\mathcal{K}_F$ *on* $C(X)$. *In particular, given* $f \in C(X)$ *and samples* $f(x_2), \ldots, f(x_{M+1})$ *for training,* $\mathcal{K}_{F,M} f$ *is an empirically computable continuous function that converges uniformly to* $\mathcal{K}_F f$ *in a single limit of* $M \to \infty$ *with probability 1. Doesn't this constitute some form of "learning" of the dynamical system generated by* F *that is compatible with Definition 11 in the Supplementary Information?*

Indeed it would, and this is exactly the kind of example that we had in mind when stating that we expect these kinds of problems may sit lower in the SCI hierarchy in the paragraph above.

Applying this procedure componentwise to the components $(F_1, \ldots, F_d)$ *of the map using snapshot data for training would lead to a consistent approximation of* F *in a single limit. However, the impossibility result stated in the paper for the class* $\Omega_{[0,1]}$ *would suggest that algorithms using* $\mathrm{Sp}_{\mathrm{ap}}(\mathcal{K}_F)$ *as part of the computation would need at least two limits. Assuming there is no loophole somewhere in my argument (e.g., the fact that* X *has no boundary makes this class of systems intrinsically different from* $\Omega_{[0,1]}$*), I believe it should be mentioned that algorithms bypassing spectral computations may be not subject to the same impossibility result.*

There are, however, some subtleties in the reasoning above. First, the SCI can depend on the norm in which approximations are measured (indeed, the first author is studying this phenomenon in a different project in the context of solving parabolic PDEs). This is particularly relevant because, for pointwise forecasting, one ideally seeks convergence in the L^∞ norm, whereas many existing results (including some involving EDMD) are formulated in L^2. Second, although the referee's reasoning (as far as we can tell) does not require a measure-preserving assumption, this assumption plays a crucial role in some of our impossibility results. There may therefore be important distinctions between measure-preserving and non-measure-preserving systems. In line with the referee's helpful suggestion, we have now clarified in the Discussion section (see the Fourth point of future work): *"Fourth, it is natural to seek lower bounds complementing the upper bounds in Table 1 for problems that do not rely on spectral computations. These may have different lower bounds than those we established, but we anticipate that the adversarial dynamical systems framework can be extended to cover them."* We believe that a discussion of the related topologies and norms beyond this paragraph is outside the scope of this paper, but warrants future work.

I would like to emphasize that this is by no means a criticism of the theoretical results presented in the paper—as mentioned in the opening of my report I believe they provide deep insights about aspects of learning dynamical systems. However, in my opinion statements such as "These impossibility results are universal, applying to any type of algorithm, regardless of what n represents" on p. 8 could benefit from some additional quantification if the example given above is valid. A modest rewording in a few instances, e.g., say something like "These impossibility results are universal among operator-theoretic algorithms utilizing computation of approximate Koopman spectra..." I believe would suffice. Even if the example given above is not valid, it might still be worthwhile to comment on it as I imagine I'm not the only one who would think of kernel smoothing as a simple way of learning continuous maps on manifolds.

We thank the referee for bringing up this example that has allowed us to improve the breadth of the Discussion section whilst making the abstract clearer. Regarding the abstract, our original wording remains logically correct, since once the computational problem is fixed—in our case, computing Koopman spectra—the universal impossibility result does indeed hold independently of the algorithm used. I.e., the impossibility result is for that problem. The referee's suggestion essentially corresponds to changing the computational problem itself, which is certainly valid but represents a distinct type of statement. We believe a more precise alteration is to adjust the Discussion as we outline above. We have also altered the abstract to make clear that we are talking about problems, and then the impossibility of algorithms for those problems.

2. *Page 2 of the Supplementary Information explains that well-definition of the Koopman operator* $\mathcal{K}_F$ *on*

$L^2(X,\omega)$ relies on the map F being nonsingular with respect to the measure ω. I think that mentioning this requirement in the main text as well would be worthwhile. In particular, the definition of the class $\Omega_{\mathcal{X}}$ on p. 8 ("... the broader class of systems where F is continuous, without any additional assumption") should be narrowed down if the intent is to define a Koopman operator on $L^2(X,\omega)$ for every element of this class. Besides well-definition, do you need closability of $\mathcal{K}_F$ to make various spectral computations meaningful?

This is a good point. While we have aimed to keep the technical prerequisites in the main text to a minimum, we agree that additional precision is warranted here. To strike a balance, we now mention this condition in the Methods section when discussing the setup of L^2 spaces. Although closability is certainly required in general, we focus throughout the paper on bounded Koopman operators (we also now state this in the Methods section), so this issue does not arise in our setting.

3. *While I believe that Table 1 provides a nice bird's-eye view of various algorithms in the literature, I do believe that certain important algorithmic nuances could be more strongly emphasized, particularly for the benefit of readers who are not specialists in the field. I can speak more closely about references [17, 60, 10] in Table 1, where I was a coauthor. In that line of work, a common theme was to simultaneously learn a well-conditioned dictionary (orthonormal basis) and solve the spectral/pointwise approximation problem for the operator in question (typically the generator of continuous-time systems) using its representation in that dictionary. For the class of systems under study (measure-preserving ergodic flows on manifolds embedded in Euclidean data spaces) this approach led to a family of algorithms which, as I understand, meet the requirements for a general algorithm in Definition 2 of the Supplementary Information. In particular, for a given set of inputs (snapshot data) and choice of approximation parameters (e.g., number of basis functions N in the notation of this manuscript) there was a concrete computational procedure producing an output such as a spectrum or matrix representation of an operator. On the other hand, looking at the original EDMD [41] and generator EDMD [79] papers, it appears that the choice of dictionary is largely left to the user. In fact, the abstract of [41] states "The method requires a data set of snapshot pairs and a dictionary of scalar observables...". Without this important input, the (generator) EDMD methods as formulated in [41, 79] do not seem to be complete computational algorithms in the sense of Definition 2, so how was their SCI determined? Is there a canonical dictionary construction that was used for systems in each class Ω? I believe that the construction of the dictionary should be taken into account if it has implications on the SCI, or an explanation should be added if it does not. In other words, I agree that minimizing the number of iterated limits required is an important consideration (as discussed, e.g., in the last paragraph of p. 9), but if certain approaches achieve part of this efficiency by relegating an important design decision to the user, namely the construction of dictionaries/bases for large classes of systems, directly comparing them with methods that offer a solution to this problem feels somewhat like an apples-to-oranges comparison.*

   This is a good point. We completely agree with the referee (and in fact our upper bounds also require a dictionary that we describe how to construct - typically it does not alter the SCI). Often the upper bounds in the table only rely on a dictionary whose corresponding projections converge strongly to the identity (with some weak assumptions about the domain of the Koopman generator in the continuous time case). We have now added a remark about this point in the caption of the table. Moreover, we have added a more technical remark in the Supplementary Information after the corresponding proof that discusses the point raised by the referee.

4. *My final comments are on the radial basis function construction described in the second paragraph in on p. 14.*

   Thank you for these points, which have enabled us to discuss the generality of our setup and the very weak assumptions needed for a sequence of dictionaries.

   (a) *I wasn't sure how k-means clustering fits in the scheme of the iterated data size ($M \to \infty$) and dictionary size ($N \to \infty$) limits. Is it obvious that k-means is sufficiently well-behaved so as to ensure that output of the algorithm converges as $N \to \infty$ at fixed M? Could one run into issues with k-means failing to converge or producing cluster centers that depend sensitively on the choice of initial condition in the k-means iteration?*

   Indeed, one must increase the number of centers (and data points to compute them) adaptively

as N increases. We have not found this an issue in practice for the range of parameters we considered, and the only requirement is that the corresponding projections onto the span of the dictionary converge strongly to the identity. The initial first dataset of length 5 is chosen to get good clustering. We have now explicitly described this in the Methods (and included an additional figure in the Supplementary Information). It is possible that k-means fails to converge outside the parameter range we consider, but a convergence analysis of k-means is likely very hard (if not impossible). As discussed in the next point, we could use a different dictionary if desired.

(b) *Independently of the above, if the bandwidth of the Gaussian RBFs is held fixed then as $M \to \infty$ the basis elements will become increasingly colinear. For a computational algorithm that operates with finite arithmetic precision, does that create stability/conditioning issues? Would that necessitate the use of some regularization procedure for sufficiently large M (or adaptive bandwidth tuning), potentially with its own parameters leading to an increased "practical" SCI?*

We select the bandwidth dependent on the data in a manner that is now fully specified in the Methods section. In practice, we did not find an increased SCI. It is possible that one would have to adjust the procedure for very large N, but this is beyond the scope of the present paper. In that scenario, one could simply construct (by hand) a dictionary of $L^2(\mathbb{R}^2)$ (in the case of the Duffing oscillator) that does not have this issue. The Duffing oscillator example is there to demonstrate the failure of EDMD to converge to spectra (and the need to use alternative methods). Whilst important, the dictionary choice is not the main highlight of that example, and, as discussed, one can change the dictionary to see similar results (the new Figure 9 shows the same phenomenon across a range of different dictionaries and systems).

Response to Referee 2

Comments and suggestions made by the referee are addressed below.
(*referee comments in italics*, our response in normal font, *quotes from revised paper in red*)

We thank the referee for their detailed feedback. Below we respond to the points in the referee's summary, and we describe the concrete steps we have taken to address them. We believe that the manuscript is now significantly improved in structure and clarity as a result. We hope that the revised version addresses the referee's concerns and conveys more effectively both the substance and scope of our work.

My overall feeling is that the authors have tried to merge a mathematics paper and a methods paper into a format for Nature Communications and the result is less than the sum of the parts.

We appreciate the referee's comments that have helped us ensure such integration enhances rather than detracts from the paper's clarity. We thank them for their feedback on how to address this by improving the clarity of our narrative, improving certain stylistic features, and their specific points below. We believe the revision now presents a more cohesive study on the capabilities and limits of data-driven learning, with both the theoretical and methodological aspects accessible to a broad audience, and each enhancing the other.

Our aim is to present significant theoretical advances alongside their direct practical applicability, yielding state-of-the-art results in data-driven dynamical systems (e.g., forecasting). The integration of mathematics and methods for applications is a crucial part of this endeavour. One can see this in our paper as follows:

- Adversarial dynamical systems (***theory***) reveal the challenges of data-driven learning: see the challenges **(C1)** and **(C2)** that are now added in the main paper to make this clearer and discussed below. By linking dynamical systems theory with computational complexity, we have established a framework clarifying the limits of using finite data to analyze complex dynamics. This provides a unifying understanding of when learning is possible versus impossible;
- These challenges guide the algorithmic design (***practice***), e.g., our new Algorithm 1 (see the added pseudocode and the quotation in red that discuses this when addressing point 7 below) and all of the algorithms that lead to classifications shown in Figures 7 and 8: these are the first general purpose convergent algorithms for data-driven spectral analysis of Koopman operators. We have also demonstrated these results on several new systems in the revised paper, in addition to the examples that were already present;
- These new techniques can be used effectively on real-world systems (***results and applications***), such as sea ice forecasting where we perform better than current benchmarks. This is discussed in reply to the referee's point 3 below: we perform better than state-of-the-art machine learning methods in terms of accuracy, number of parameters and computation time. Specifically, we use two orders of magnitude fewer parameters, several orders of magnitude less computation time, and achieve greater accuracy.

Hence, theory, state-of-the-art methods, and results and applications are intertwined. We believe the multidisciplinary combination of rigorous theory, algorithmic development, and scientific application makes this a strong fit for an interdisciplinary journal. These three components cannot be separated and we view their integration as a strength of the revised paper.

In addition to the point by point response to the referee's detailed comments below, we have undertaken several substantial revisions to address this concern of the referee directly:

- **Organization of Results:** We have reorganized the Results section to follow a clearer progression. The section begins with a roadmap paragraph that outlines what will be presented:

  "We first present three illustrative examples that demonstrate the reliability, advantages, and broad applicability of our general-purpose learning algorithms: (i) a classic oscillator where previous methods diverge but ours converges, (ii) a fluid flow with continuous spectrum where we extract meaningful eigenvalues, and (iii) an Arctic sea ice dataset where we uncover physically interpretable modes with error bounds and achieve state-of-the-art forecasts. We then examine the underlying theoretical foundations,

showing that our algorithms optimally match fundamental limits revealed by adversarial dynamical systems (Figure 7). Indeed, analyzing the reasons behind these fundamental limitations enabled us to pinpoint the essential algorithmic properties required for reliable convergence. Finally, we provide a unified classification of the complexity of these learning problems, clarifying precisely when learning is possible and when it is fundamentally impossible."

This explicit mapping ensures the reader knows exactly what to expect and sets the narrative right from the start. Starting with illustrative examples (the order of examples remains the same: a simple oscillator through to a complex real-world data example), followed by the construction of adversarial systems and the challenges they reveal (e.g., see the added challenges **(C1)** and **(C2)**), and culminating in the development of new algorithms and their classification. The now-combined section ***"Unified complexity classifications of learning"*** ties everything together into a framework. It also ends with with a narrative emphasis: *"In short, we now have a complete characterization of when data-driven spectral learning can succeed and when it cannot."* By the time Results concludes, the reader has seen tangible evidence, theoretical explanation, and a big-picture classification – a coherent progression.

Moreover, the theoretical results are consistently linked back to the examples, so that the examples motivate and illustrate the theory, while the theory explains their behavior. We have also revised the headings for each example to clarify their respective contributions—Duffing: ***"Overcoming lack of convergence in current methods"***; Flow: ***"Eigenvalue extraction in a high-dimensional chaotic flow"***; Sea ice: ***"Physical modes and state-of-the-art forecasting"***.

- **Integrated Storyline:** The content is now organized so that practical examples lead naturally into theoretical insights, which then inform the development of new methods. This creates a feedback loop between application and theory rather than a stark division. The Results section exemplifies this integration: after demonstrating the problem (standard methods failing) and the solution (our algorithm working) in Examples 1.1–1.3, the text turns to why this is happening, introducing the theoretical limits (impossibility results). Then, rather than leaving the theory abstract, it circles back to practice by showing how those theoretical insights guided the creation of optimal algorithms and a classification framework. By the time we reach the Discussion, we explicitly celebrate this synergy: *"By combining a new theoretical complexity framework with practical algorithmic advances, our work provides a rare instance in ML of a synthesis between rigorous theory and application."* This statement is backed by the content, as the reader has seen the theory and application interwoven.
- **Emphasis on scientific findings:** We have revised the paper to emphasize scientific findings over technical details, which are now deferred to the Methods (e.g., definitions like "perfect measurement device" and inner products for EDMD) or the Supplementary Material where appropriate. The focus is now on key insights—such as the discovery of hidden modes in Arctic sea ice and their role in long-term predictability, or the reasons why adversarial systems occur. Any mathematics is distilled into its key findings and intuition in the main text, preventing it from overwhelming the narrative. Similarly, the methods aspects are mostly in the Methods section at the end, with brief summaries in the examples to guide understanding and interpretation. Combined with cross-references connecting them, this ensures that each part contributes to the story without derailing it. More broadly, our framework offers a unified perspective (spanning both the mathematical and practical aspects) on when learning is fundamentally possible or impossible, representing a significant conceptual contribution. The Discussion also unifies the narrative by revisiting how adversarial examples led to trustworthy algorithms, and how both together advance the goal of reliable ML in applications. In short, the motivation and implications are foregrounded, with technicalities serving to support them.
- **Theory suitable for general scientists:** We have simplified the theoretical content by removing unnecessary or heavy notation and eliminating any reliance on advanced mathematical concepts. Formal theorems are now presented in the Supplementary Information, while the main text adopts an accessible, explanatory tone that references them (yet remains precise in the statement of results). This shift ensures that the results—one of the key strengths of our study—are understandable to a broad scientific audience without requiring any mathematics beyond what might be expected of an undergraduate in any of the physical sciences. Throughout, we have eliminated jargon and avoided any stylistic elements that resemble a mathematics paper. Key ideas, including the impossibility results and the

SCI classification, are now explained conceptually and with intuitive examples, making the narrative clearer and more approachable.

- **Expanded Methods section:** We have carefully separated and substantially expanded the Methods section (including additional examples and experiments, some also in the Supplementary Information), as requested by the referee. Any content that reads like step-by-step algorithms, derivations, or proofs is now either in this Methods section or the Supplementary Information. For example, the derivation of the $h_N(z, F)$ function is now in the Methods, while the Results provide accessible explanations for general readers where needed. This clarifies the distinction between high-level findings (in Results/Discussion) and technical validation (in Methods/Supplementary Information).
- **Introduction and Discussion:** We have tightened the Introduction and Discussion to clearly convey the paper's motivation, central question, and overarching narrative. See for example the big question in the abstract/intro: *"When can we reliably learn system behaviors from data, and when is it impossible?"* This framing is now echoed throughout the manuscript. For example, after the three examples we state: *"These practical advantages motivate a return to our central question: When can we reliably learn system behaviors from data, and when is it impossible? To address this, we present our theoretical results, with full proofs provided in the S.I."* We highlight the broad interdisciplinary relevance of our unified framework for trustworthy data-driven learning—spanning applications from climate forecasting to safe AI and control—and ensure that theoretical advances, algorithms, and methodological details are appropriately referenced. The Discussion explicitly frames the significance of the work, including its resolution of a longstanding open problem for Koopman operators and demonstration of real-world significance (e.g., insights into Arctic sea ice decline and forecasting improvements) and the generality of the approach (applicability to other methods and areas of scientific computing). This is followed by a forward-looking section outlining open problems and future directions in a concise way. The Discussion section ends with a statement on the importance of knowing the impossible as well as the possible (and how these aspects combine): *"With this surge of interest and innovation, it is crucial for the community to grasp not only what is possible but also what is fundamentally impossible. This prevents the pursuit of unattainable algorithms or methods, safeguards against potentially catastrophic errors, and reveals the conditions under which learning is feasible: upper and lower bounds inform and sharpen one another. This process led us to our convergent algorithms. Such classifications are essential if we are to fully harness the power of ML in dynamical systems."*
- **Logical transitions:** Throughout, we have linked the three aspects above (Theory, State-of-the-art Methods, Results and Applications) and bridged sections to make the paper easier to read. For example, we have added the above text at the start of the Results section, the transitions discussed in the previous bullet point, and linked the theory back to the examples:
  - *"Example 1.1 demonstrated the convergence of this algorithm applied to the Duffing oscillator, while Figure 9 confirms its performance on other systems. Moreover, the error bounds achieved by the algorithm enabled improved sea ice forecasts in Example 1.3 (e.g., Figure 5)."*
  - *"Example 1.2 applied this approach to a complex fluid flow, see also the Methods. This two-limit approach is optimal, as no single limit method can solve these problems. Figure 8 summarises the SCI classifications of computing spectral type."*

  At the start of *"Adversarial systems reveal challenges"* we have stated: *"We first construct adversarial dynamical systems that expose two fundamental challenges in computing Koopman spectra. These show lower bounds on problem complexity. Even with infinite data, no algorithm can guarantee learning certain system behaviors, a first example of inherent limits in data-driven system learning."* At the start of *"A universal algorithm with learning guarantees"* we have stated: *"Learning from the above results, we now show that Koopman spectra can be computed from trajectory data, provided two key conditions are satisfied. Together with the other algorithms discussed below, these show upper bounds on problem complexity. This result also resolves the fundamental open problem of data-driven computation of* $\mathrm{Sp}_{\mathrm{ap}}(\mathcal{K}_F)$*."* There are many other connections made throughout the manuscript.

We believe these improvements better highlight how our results and innovations collectively represent a significant advance in data-driven dynamical systems (as justified in our Results, e.g., see the discussion of

the referee's point 3 below, as well as the remarks of Referee 1, which substantiate this claim), addressing the referee's concern that the paper should be more than the sum of its parts.

Finally, we would like to point out that there are numerous applied mathematics papers in *Nature Communications* (see list below for a small sample) that combine mathematics and new methods in a similar way to the structure and flavour of how our paper has now been revised, following the helpful suggestions by the referee. Once again, we thank them for helping us improve the presentation and narrative of our paper.

There are many claims that I would say are on the "over-the-top" side, and that are not substantiated upon reading the manuscript.

We thank the referee for pointing out how we can improve the clarity and strengthen the connection between our claims and the supporting evidence. It was certainly not our intention to make any "over-the-top" statements. We believe this concern stems from a lack of clarity in how some results were presented, rather than from unsupported claims, and we have now revised the manuscript to address this. We also explicitly tie each example to the broader claims – for instance, noting that Example 1.1 illustrates the robustness of their method where EDMD fails, and Example 1.3 achieves state-of-the-art forecasts compared to deep learning methods (with references to figures showing the results).

Brief overview of support of claims: Below, in response to the referee's detailed comments, we explain how the claims in question are supported by specific theoretical results, algorithmic developments, and empirical findings. We have improved the exposition to make these connections more explicit.

For now, here is a brief overview:

- The claim of "first general guarantees of convergence" for spectra is referring to Koopman operators. To our knowledge, and based on a comprehensive understanding of the literature and experience built up over two decades working in Koopman spectral methods and operator learning more broadly, no general-purpose convergent algorithms currently exist for this setting (see also the summary of the literature in Table 1). To avoid any ambiguity, we have revised the abstract and introduction to explicitly state "for Koopman operators".
- The claim of "illustrates how AI models using Koopman operators can deliver superior performance at a fraction of the computational cost" is backed up by both the comparative figure with IceNet and our discussion of parameter counts and training time. As detailed in our response to the referee's point 3 below, we have clarified this in the revised manuscript by expanding the discussion, presenting it in a dedicated paragraph, and referencing it explicitly in the figure caption.
- The claim "our work contributes to understanding the challenge of hallucination in LLMs" has now been expanded beyond the reference and discussion we originally provided, connecting it with Koopman operators, chaos, and continuous spectra. We have added references that explicitly discuss this connection with Koopman operators and challenges of their spectra.

Each of these points are discussed in detail below in response to the referee's specific comments.

Additionally, we have carefully reviewed the manuscript and revised the language throughout to soften or qualify statements that could be interpreted as overstated. We have ensured that all key claims are now explicitly tied to supporting results, with clear references to theorems, citations, or numerical evidence. We are grateful for the opportunity to clarify these points and hope the revised version more clearly reflects the strength and scope of the contributions without overstating them.

It is often unclear what the new results are.

We thank the referee for pointing out how we can be more specific about what our results are. To address this, we have refined the presentation of contributions in the Abstract, Introduction, and Discussion sections. In particular, the revised first paragraph of the Discussion section also makes the contributions clear, following the feedback provided by the referee on those points:

"We have developed a powerful technique—namely, the construction of adversarial dynamical systems (see

*§Adversarial systems and Figure 11)—to establish the first impossibility re- sults in the field of data-driven dynamical systems. We identified conditions (**(C1)** and **(C2)**) for which no sequence of randomized algorithms (e.g., randomly sampling trajectories) can succeed with probability greater than 50%. These failures are not rare, but represent fundamental barriers. These barriers provided insights, which we used to develop a suite of new algorithms (Algorithms 1–7 in the S.I.) that achieve optimal performance and are provably convergent. These algorithms produce trustworthy, verifiable outputs, and include what we term 'multi-data-limit' methods (see Figures 7 and 8). By linking dynamical systems theory with the foundations of computation, we established a computational complexity framework for data-driven dynamical systems. This framework clarifies the inherent limitations of using finite data to analyze complex dynamics while also highlighting new algorithmic possibilities with broad applications. By combining a new theoretical complexity framework with practical algorithmic advances, our work provides a rare instance in ML of a synthesis between rigorous theory and application."*

Throughout the manuscript, we explicitly highlight theoretical, algorithmic, and applied advances, consistently referencing relevant theorems, algorithms, and examples. We have also placed the three examples of our algorithms in action in the Results section. This revision (and further changes to the Results section as above) should help readers more readily distinguish foundational background material from methodological innovations and illustrative examples.

Theory is mostly vaguely stated with little detail (in some cases, without citation) and is not well-linked to specific theorems in the supplementary material.

The statements of the theoretical results are now clearly articulated. We have revised the main text to more clearly identify and link each theoretical statement to specific theorems in the Supplementary Information or the Methods (e.g., for implementation). In particular, when we refer to convergence guarantees or spectral properties, we now include either a direct citation to the relevant result or an inline reference such as "(see Theorem X in Supplementary Information)." All other theory has been referenced whenever appropriate. This balance ensures the main text stays focused, yet a motivated reader can verify every claim in detail. Importantly, we do not leave new concepts unexplained waiting in the Supplement – we give at least a high-level explanation in the main text and point to the S.I. for rigor.

The methods section is very brief, to the point where it would be impossible to reproduce some of the results.

We thank the referee - this helpful and constructive suggestion has helped us substantially expand and improve the Methods section (see also our reply to detailed comment 13 below). We now:

- Provide a more precise description of the new algorithms, including implementation details such as data sampling and dictionary choices. Any parameters used in the experiments are either given or clearly referenced.
- Include pseudocode for the key steps of the algorithm in the Supplementary Information, along with parameter settings for all experiments.
- Added additional experimental details to the Supplementary Information, following the referee's comment 13 below. For example, various choices of sampling for the Duffing oscillator, and other example systems with data collection and dictionary choices discussed.
- Add schematic illustrations (including one from the earlier Figure 1, now moved here) to clarify how the proof idea works.

We have structured the Methods into logical sub-sections that mirror the flow of the Results:

- **Computing Koopman modes and EDMD:** This first sub-section concisely reviews the standard EDMD approach and how Koopman modes are obtained. It provides equations for the correlation matrix estimates and describes the computation of modes via a least-squares approach. This ensures that a reader can implement this method and understand the baseline. The inclusion of these details addresses reproducibility for comparing against EDMD.

- **Upper bounds: A suite of convergent algorithms:** This subsection summarizes the new algorithms. Rather than simply referencing pseudocode in S.I., we walk through each algorithm's core steps in prose. For example, we explain how to construct three matrices of inner products that allow us to compute error bounds. The text also explicitly notes the assumptions needed for each algorithm.
- The Methods then has separate labeled subsections for the **Duffing oscillator**, **Further examples comparing EDMD and algorithms**, **High Reynolds number fluid flows** and **Arctic sea ice**. Each of these provides concrete details for full reproducibility.
- **Lower bounds: The method of adversarial systems:** The Methods concludes with a subsection on the construction of adversarial examples. This indicates how the proofs work, satisfying those curious about the construction without overwhelming others.

Our aim is that these changes make the paper more transparent and reproducible. In line with *Nature Communications* practice, we will provide code and data to enable full reproduction of the results.

The comparison with EDMD could be made much clearer and convincing with greater detail.

We thank the referee for prompting us to make the comparison with EDMD clearer and more convincing. We are now fully explicit in our implementation and EDMD's role in the comparisons. Many of these changes are described below in our response to the referee's specific point. They include more detail in the initial description of the example, much greater detail in the Methods section (and additional examples of the comparison), with corresponding further plots in the Supplementary Information.

One can think of EDMD as producing a finite matrix approximation of the Koopman operator (which one may think of as an infinite matrix acting on observables). In essence, EDMD parallels finite element methods for differential operators [1], with the mass and stiffness correlation matrices $G_{ij} = \langle g_j, g_i \rangle$ and $A_{ij} = \langle \mathcal{K} g_j, g_i \rangle$ $(i, j = 1, \ldots, N)$ now estimated from snapshot data (using $\mathcal{K} g_j(x^{(m)}) = g_j(y^{(m)})$):

$$G_{ij} \approx \hat{G}_{ij} = \frac{1}{M} \sum_{m=1}^{M} g_j(x^{(m)}) g_i(x^{(m)}), \quad A_{ij} \approx \hat{A}_{ij} = \frac{1}{M} \sum_{m=1}^{M} g_j(y^{(m)}) g_i(x^{(m)}).$$

The resulting EDMD approximation is the $N \times N$ matrix $\hat{G}^{-1}\hat{A}$ and corresponds to a projection of the Koopman operator onto the space spanned by the finite number of observables $g_1, \ldots, g_N$.

We have also explained why EDMD fails – it effectively performs a finite-dimensional projection of an infinite-dimensional operator, which can introduce spurious eigenvalues and divergence as the basis size increases. Making this definition of EDMD explicit (we now do so in the Methods section) has also helped us address some of the referee's detailed comments below.

The sea-ice example lacked detail in the methods section and in the climate interpretation.

We wish to thank the referee for prompting us to clarify these points since we believe that this example is now one of highlights of the revised manuscript with numerous climate interpretations. We have also expanded the details of this example in the Methods section. All details such as the source of the data and processing, forecasting experimental setup, periodic baseline, EDMD with delay embedding, comparison with benchmarks etc. are all explicitly given. Changes have also been made following suggestions of the other referee.

Climate interpretations: There are now several details on climate interpretation (not a complete list):

- We substantially expanded the interpretation of Koopman modes, as suggested by the referee below (see point 6). This includes a discussion of how the modulus and argument of each eigenvalue have physical meaning, and how the associated Koopman mode highlights geographically significant regions. For example, an eigenvalue $\lambda = 1$ for the annual mean, a complex pair around $e^{\pi i/6}$ corresponding to the annual cycle (monthly harmonic), and several decaying modes with $|\lambda| < 1$ representing long-term decline trends.

- We now explicitly link the hidden decaying modes to dissipative dynamics, particularly long-term sea ice decline (see trend plot in the Supplementary Information), providing strong evidence of a connection to climate-driven sea ice loss. These hidden modes, with nonzero $\arg(\lambda)$ represent seasonal patterns modulated by long-term decline. We also highlight how these modes combine with annual variation modes, supported by canonical correlation analysis.
- We describe how the hidden mode is spatially concentrated in the Barents and Kara Seas, and its potential links to extreme weather patterns. We also discuss why such modes have been overlooked in Arctic data, unlike analogous Antarctic trends.
- We address the credibility of the identified modes by discussing the error bounds shown in the plots. This ensures that the modes are real and not artefacts (in contrast to many spurious modes produced by EDMD), and illustrates how these verified modes improve forecasting.
- We describe how the modes capture seasonally modulated reemergence of correlations (see Supplementary Information), revealing memory effects in the climate system. Specifically, anomalies in the growth season reappear in the melt season, even when correlation is lost during the intervening winter.
- We discuss how these modes could influence large-scale climate patterns (e.g., ocean circulation changes), and their practical implications for shipping routes and climate resilience. We also note potential connections to tipping points, such as increased Barents–Atlantic mixing and shifts in AMOC behavior.
- We examine how the modes change over time (e.g., in ten-year windows from 1980–2019). The mean mode shows a persistent decline in sea ice and reduced winter extent. The annual cycle modes show shifting geographic patterns, with marginal seas (Beaufort, Kara, etc.) exhibiting increased amplitude—suggesting growing seasonal contrast. This is consistent with the presence of a slow decaying mode in the spectrum shown in Figure 2 of the main text.
- We found no clear link between prediction accuracy and El Niño events. Forecast skill drops during summer months due to the well-known "spring predictability barrier," but these months also show the largest gains over SEAS5.

In summary, we believe the revised manuscript now offers many rich and detailed physical interpretations of the climate results.

We would also like to note that Referee 1 identified substantial climate interpretation in our sea ice example, which we believe may have been overlooked by Referee 2 due to the issues raised in detailed comment 6 below. Several of the interpretations and remarks discussed above have been enhanced in the revision following Referee 1's report. As we explain further below (point 6), Referee 2's comment on the Koopman modes highlighted areas where we had not conveyed these points as clearly as we should have. This has now been addressed, and we sincerely appreciate the referee for prompting this important clarification.

In summary, the manuscript was long and (to me) confusing. I struggled to learn much from the manuscript. If the mathematics is new, it would be better to publish it in a specialist journal for an audience that can understand the mathematics. Similarly, the applied components need much greater detail on the methods and interpretation and could also be submitted to a specialist dynamics journal. I do not recommend publication in Nature Communications.

We thank the referee for their feedback, which has helped us balance mathematical innovation with practical relevance, combining new theoretical results, algorithmic development, and real-world applications in a way that is broadly accessible. In response to this and other comments, we have substantially revised the manuscript for improved clarity and exposition. As outlined in our response to the detailed comments below, we now provide clearer motivation for each result (e.g., linking the challenges revealed by adversarial dynamical systems to new algorithmic developments) and significantly expand the methodological and interpretive components, particularly for the Duffing oscillator and sea ice forecasting examples. We are grateful to the referee for their detailed comments, which have helped us address these issues.

Length: We have taken the referee's feedback and carefully revised the manuscript to tighten the exposition and eliminate unnecessary material or complexity. As a result, and despite incorporating new content (such

as additional experiments, a longer Methods section, and the new Figure 9) in response to both referees' comments, the revised manuscript is now half a page shorter than the original submission, with the text up to the Methods section trimmed by more than 2 pages. We also note that *Nature Communications* has published many papers of comparable or greater length in this area.

Mathematics: We thank the referee for pointing out how we can ensure the mathematics in the manuscript is suitable for a general scientific audience. In the revision, we have made the main text self-contained and presented the key insights in an accessible manner, without requiring an advanced mathematical background. Familiarity with the standard concepts of functions, separation of variables, eigenvalues, and derivatives—typically covered in any undergraduate course in the physical sciences—suffices, including for understanding the Methods section. Since we are submitting to the physics and mathematics track at *Nature Communications*, we believe it is fair to assume this background. Moreover, we have explained terms intuitively throughout.[1] Key equations (including those in the Methods) that illustrate the approach are accompanied with plain-language descriptions. For example, the caption of Figure 9 gives a clear intuition behind the plotted level sets of the approximate eigenfunction error ε.

As discussed above, the revised version now more clearly articulates the central message: that the new mathematical results (e.g., impossibility results through adversarial dynamical systems) directly enable provably convergent spectral algorithms for complex systems, outperforming existing approaches such as EDMD and deep learning methods (illustrated in Figures 1 and 9 for EDMD nonconvergence, and Figures 5 and 6 where we outperform deep learning approaches). This connection is central to the broader impact of the work. Our submission to *Nature Communications* is motivated by the broader insight that new theoretical tools can unlock practical advances in spectral analysis and forecasting of complex systems — especially those for which standard methods fail. The mathematics we develop is deliberately structured to yield algorithms that are robust, interpretable, and directly applicable to systems with continuous or mixed spectra — a setting of high relevance in physics, climate science, and engineering. We believe this interdisciplinary approach, combining rigorous theory with algorithmic and scientific relevance, aligns well with the journal's scope.

We would also note that *Nature Communications* has published numerous mathematically sophisticated papers in data-driven dynamical systems and related fields (see the list below). As *Nature Communications* states in its guide for authors: *"Papers published by the journal represent important advances of significance to specialists within each field."* In this spirit, we believe that a paper that is understandable to a reader from the physical sciences with a modest background in calculus — as we have worked hard to ensure following the referee's helpful feedback — is well-suited to the journal.

Broader relevance of the framework: The mathematical framework underlying our construction of adversarial dynamical systems is of broad and interdisciplinary interest. As outlined in the Discussion, it has implications for a wide range of fields, including partial differential equation (PDE) learning, the identification of latent or unobserved dynamics, control theory, and the analysis and training of neural ordinary differential equations (neural ODEs), which are themselves formulated as dynamical systems:

"This perspective is relevant to a range of methods—including SINDy [10], neural ODEs [89], Fourier neural operators [90], LSTMs [91], and PDE-net [92]—as well as to other areas of scientific computing with ML. For example, recent work has shown that linear elliptic PDEs can be learned from input-output pairs [93], analogous to snapshot-based learning. Whether similar approaches extend to hyperbolic or nonlinear PDEs remains open, but our proof techniques may offer insight into this challenge."

[1] The Supplementary Information contains full proofs, using standard undergraduate calculus and probability tools. These are not required to understand the results. Importantly, we now ensure that each result in the main text is presented with clear motivation and interpretation, and we include high-level intuition behind the constructions and proofs to help guide the reader. Following the referee's point about our initial Figure (point 0 below), we have taken the subfigures and placed them in the Supplementary Information to aid with intuition behind the proofs. Furthermore, we have now ensured that the mathematical results/statements presented in the main paper can be understood without reference to the proofs. We have also explained the mechanisms at play in all of the results, so that a reader can gain this insight without reading the proofs. We would like to thank the referee for their remarks below about explaining the mechanisms.

These connections underscore the relevance of our approach beyond the immediate context of spectral learning and Koopman theory, offering new insights into the fundamental limits and capabilities of data-driven modelling across scientific disciplines. We believe the vibrant community interested in these types of results will appreciate the novel insights presented.

Applied components: We have made several concrete improvements to the applied components (see the lists above and also below). We now provide detailed implementation information, including algorithmic steps, parameter settings, and data preprocessing choices. We have added pseudocode and numerical diagnostics to the Supplementary Information to enhance transparency and reproducibility. We have extended the discussion of the sea ice case study to better connect the computed Koopman modes with known climate dynamics, providing a clearer interpretation of the results.

Examples of *Nature Communication* papers in this field with a similar style: Many impactful *Nature Communications* papers in the mathematics of data-driven dynamical systems reflect a similar spirit and structure to the revised version of our paper—for example:

https://www.nature.com/articles/s41467-021-26357-x

https://www.nature.com/articles/s41467-021-21331-z

https://www.nature.com/articles/s41467-022-28518-y

https://www.nature.com/articles/s41467-018-07210-0

https://www.nature.com/articles/s41467-017-00030-8

https://www.nature.com/articles/s41467-024-48033-6

https://www.nature.com/articles/s41467-020-15582-5

https://www.nature.com/articles/s41467-021-23479-0

https://www.nature.com/articles/s41467-024-46598-w

https://www.nature.com/articles/s41467-024-45323-x

https://www.nature.com/articles/s41467-022-30628-6

https://www.nature.com/articles/s41467-021-21554-0

We hope that the revised manuscript now more clearly demonstrates the value of the theoretical contributions, the strength of the algorithms, and their relevance to challenging real-world applications—especially in contexts where existing methods fall short. Notably, *Nature Communications* welcomes high-quality research in all scientific disciplines, including mathematical and applied sciences.

Once again, we thank the referee for their time and for prompting improvements in content, clarity, and accessibility that have, in our view, substantially strengthened the manuscript.

Detailed points:

0. *Figure 1 serves no scientific purpose because it contains 16 complicated, small subimages, which are not understandable from the caption.*

   We thank the referee for prompting us to improve how these visual elements are presented in the paper. We understand the concern that Figure 1, in its original form, may have appeared visually dense and difficult to interpret without sufficient context early in the paper. Our original intention was to provide visual intuition for the key mechanisms behind several of the theorems and proofs. In earlier discussions, colleagues—including those not directly involved in the project—found the figure helpful in understanding these arguments. However, we now recognize that what works well in an interactive setting (e.g., on a whiteboard or in a seminar) can become overwhelming in the compressed and static

format of a manuscript, particularly near the beginning and without sufficient narrative support.

In response to the referee's comment, we have:

- Selected a single, representative subfigure from the original set and moved it to the Methods section, where we now walk through the associated mechanism in detail,
- Relocated the remaining subfigures to the Supplementary Information, where they serve as optional visual support for readers interested in the technical details.

1. *In the abstract an "optimal algorithm" is mentioned but I could not find it. This algorithm should "overcome key limitations of standard methods", but it was not clear to me what these key limitations were or how a presented algorithm overcame them.*

   We are grateful to the referee for prompting us to strengthen this connection between the abstract, theoretical results, and practical contributions. The "optimal algorithms" mentioned in the abstract refer to the new class of methods we develop and analyse in the convergence theorems, which are now presented in the Methods section along with pseudocode in the Supplementary Information.

   Limitations of standard methods: The main issue we address is non-convergence: widely used methods such as EDMD often fail to produce reliable spectral estimates. This is demonstrated in our Duffing oscillator example, where EDMD yields spurious convergence to incorrect eigenvalues. In contrast, our algorithms return verified approximations with provable convergence bounds. We now highlight this contrast more clearly in the Results section and have added an additional figure with additional example systems (Figure 9 of the main text, also discussed below). These examples demonstrate the convergence of our approach (we have a proof that it always works) and the typical non-convergence of EDMD.

   Further examples: These limitations also manifest in other ways. For instance, in the Arctic sea ice example, our algorithm achieves more accurate long-range forecasts and captures physically meaningful Koopman modes, with quantifiable error bounds—something standard approaches do not provide.

   To address the referee's concerns, we have made the following revisions:

   - Described each algorithm in the Methods section;
   - Added a dedicated section in the Supplementary Information summarising the algorithmic framework and including pseudocode for clarity and ease of reference;
   - Explicitly referenced this section throughout the main text and in the caption of Figure 1 (previously Figure 2), which illustrates the algorithm in action;
   - Clarified in the abstract that the key limitation of standard methods we overcome is their lack of convergence guarantees. This is also highlighted in the title of the Duffing oscillator example: ***"Overcoming lack of convergence in current methods"***;
   - Clarified in the section "Unified complexity classifications of learning" why they are optimal;
   - Discussed how these algorithms realise the bounds of what is possible (i.e., tight optimal upper bounds) in the captions of Figures 7 and 8.

   We believe these revisions clarify both the nature of the "optimal algorithms" and the core limitations they are designed to overcome.

2. *The abstract and p2 claims "first general guarantees of convergence". Perhaps the devil is in the word "general", but I think this is a misleading statement given the substantial work on eigenvector convergence of operators.*

   We thank the referee for this helpful comment on how to clarify this claim. We are not asserting the first convergence results for eigenvectors of operators in general. As the referee rightly points out, there is an extensive literature on this topic, including the classical Babuška–Osborn theory [2] for finite element approximations of eigenvalues and eigenfunctions of elliptic operators. These results, however, typically apply to self-adjoint (i.e., Hermitian, meaning that the operator is equal to its conjugate transpose) or compact operators (or those with compact resolvent), where the spectrum consists solely of isolated eigenvalues with finite multiplicity. (See also Chatelin's textbook [3] which treats regions of discrete spectra for a wide range of operators.)

In contrast, Koopman operators are often non-self-adjoint and may possess continuous or mixed spectra. In such settings, standard operator-theoretic results (such as Babuška–Osborn theory or the methods in Chatelin's textbook) do not apply, and commonly used data-driven methods such as EDMD are known to fail or produce misleading results. To our knowledge, our work provides the first general-purpose, provably convergent algorithms for Koopman spectral estimation in the presence of continuous or mixed spectra. The table included in the paper summarises the landscape of existing algorithms, most of which either assume specific structural properties (e.g., discrete spectra or linear dynamics), or focus on related but distinct computational tasks.

To avoid any ambiguity, we have revised the abstract as:

> *"Our methods provide the first general convergence guarantees for Koopman operators..."*

Similarly, the introduction now states:

> *"In particular, Koopman operators are generally non-self-adjoint (non-Hermitian) and often have a continuous rather than discrete spectrum, so much of the classical toolkit for computing spectra [33,34] does not apply."*

and then later:

> *"We overcome its limitations by introducing the first general-purpose, convergent methods for learning Koopman spectral properties (Algorithms 1–5 in the S.I., see top panel of Figure 1)..."*

so that the meaning of our claim is clear. We thank the referee again for prompting this important clarification.

3. *The abstract states that the sea-ice example "illustrates how AI models using Koopman operators can deliver superior performance at a fraction of the computational cost". I could not find anything in the manuscript illustrating this.*

We thank the referee for prompting us to make this statement clearer. The comparison referenced in the abstract is illustrated in Figures 5 and 6, which present the forecast performance of our Koopman-based method relative to leading machine learning models, including IceNet.

Previously, we had written in the main text:

> *"Moreover, this is achieved at a fraction of the computational cost and with significantly fewer parameters than deep learning approaches (IceNet uses 4.4×10^7 trainable weights, while the Koopman approach relies on an observable space of at most 510 dimensions corresponding to 260100 parameters). Koopman models are optimal as they capture the minimal number of components (pseudoeigenfunctions) necessary to represent the system's dynamics."*

For context, IceNet demonstrated significant performance improvements over SEAS5, which contributed to its publication in *Nature Communications*. Our method achieves comparable—and in several metrics, superior—forecast accuracy relative to IceNet, while using orders of magnitude fewer trainable parameters and substantially less computational cost.

In response to the referee's feedback, we have now made these comparisons more explicit in the revised manuscript and signposted them in several places:

- When discussing this aspect at various places in the paper, we have referenced the relevant figures. E.g., on page two of the introduction we now write: *"we achieve state-of-the-art Arctic sea ice forecasts (Figures 5 and 6)"*;
- In the caption of Figure 5 we have added: *"Moreover, this is achieved using orders of magnitude fewer trainable parameters and substantially less computational cost."*
- We have updated the above text so that it is now given as an expanded stand alone paragraph: *"This improvement is achieved at a fraction of the computational cost and with significantly fewer parameters than deep learning approaches. IceNet uses 4.4×10^7 trainable weights and requires over a day to train on a Nvidia Quadro P4000 GPU, while the Koopman approach relies on an observable space of at most 510 dimensions (260100 parameters) and is trained on a laptop in under a second."*

We hope this clarification makes the basis for the abstract claim more transparent, and we thank the referee again for prompting this improvement.

4. *Figure 2(c) is supposed to show that the "new method converges". It is unclear what the error is measuring, and the y-axis range for the error for the "new method" did not dip below 0.01. Similar to figure 1, most panels were not able to be comprehended from the caption.*

We thank the referee again for helping us improve the clarity and presentation of this figure and its accompanying explanation. We have now clarified both the definition of the error metric used in Figure 2(c) (now Figure 1(c)) and its interpretation.

The error plotted corresponds to a (convergent) approximation of $\sup_z \sigma_{\text{inf}}(\mathcal{K} - zI)$ over an algorithm's output (ours or EDMD), where σ_{inf} denotes the smallest singular value and I is the identity operator. This quantity captures how closely the computed spectrum approximates the true spectrum of the Koopman operator. In the conservative (i.e., measure-preserving) setting, this approximation becomes exact:

$$\sigma_{\text{inf}}(\mathcal{K} - zI) = \text{dist}(z, \text{Sp}_{\text{ap}}(\mathcal{K}))$$

In the dissipative case, it remains a lower bound so it still provides a reliable signal of spectral accuracy or non-convergence.[2] For instance, the plateau in the EDMD error curve remains indicative of failure to recover the spectrum. We have added a more detailed explanation of this in the Methods section.

To connect this error with the theory developed in the main text, we also now explain how it relates to the parameter ε appearing in our definition of approximate eigenfunctions and temporal coherency. This connection clarifies why the level sets of the plotted error identify regions of near-eigenvalue behavior.

In response to the referee's comment, we have made the following changes:

- Clarified the vertical axis label in Figure 1(c),
- Expanded the figure caption to explain the meaning and relevance of the error metric,
- Improved the caption and presentation of all panels in Figure 1 for clarity, including the top and bottom panels (see also our response to Comment 13),
- Expanded the description in the Methods section to aid interpretation.

Regarding the comment on the error not dipping below 0.01: The convergence rate of our algorithm is problem-dependent and influenced by factors such as spectral type and sampling scheme (which the user often has no control over in applications). One can in fact prove that it is generally impossible to establish a convergence rate for *any* algorithm. Nonetheless, a final error of 0.01 is generally considered excellent in data-driven spectral approximation (and machine learning in general). The key point in this figure is not the absolute error value but the observed convergence trend of the new method, in contrast to the stagnation of EDMD. Additional examples (see new Figure 9) illustrate similar trends and support this interpretation (and have corresponding different values on the y-axis - some systems are inherently harder than others). For this example, one can associate the error with a timescale of coherency (this is discussed after the equation $\phi_\varepsilon(x_n) = \lambda^n \phi_\varepsilon(x_0) + \mathcal{O}(n\varepsilon)$) and it corresponds to a worst case timescale of about 30 seconds (which is very good for this system).

5. *The claim on p3 that "our work contributes to understanding the challenge of hallucination in LLMs" lacks evidence.*

We thank the referee for the opportunity to clarify our intent and to provide further references supporting this link. We do not claim to solve the hallucination problem in LLMs, but rather to point out how effects such as continuous spectra and adversarial dynamics may contribute to understanding this phenomenon.

The original sentence in question followed a discussion including a reference supporting the connections between LLM architectures and dynamical systems with continuous spectra. In the revision, we have expanded this discussion and added two further references:

[2]For the examples in our paper, one can show that $\text{dist}(z, \text{Sp}_{\text{ap}}(\mathcal{K})) = \mathcal{O}(\sigma_{\text{inf}}(\mathcal{K} - zI))$, so convergence of the plotted error to zero for the new method provides rigorous evidence of spectral convergence.

(a) a study linking pathological behavior in LLMs to Markov chains (whose operators are Koopman operators);

(b) a DeepMind paper which makes connections with Koopman operators explicit, including language models, and uses it to study architectures.

The relevant paragraph now reads:

"The adversarial dynamics we construct may also shed light on hallucinations in LLMs. These models generate trajectories (sentences) over a state space (words) via one-step-ahead prediction, a process linked to Koopman operators [25,44,45]. The adversarial systems we construct that impede learning often have Koopman operators with a continuous spread of frequencies, characteristic of chaotic dynamics. This enables short-term accuracy but causes long-term unpredictability due to sensitivity to initial conditions, mirroring how small prompt changes in LLMs can cause divergent outputs. Our results may therefore help explain inherent limitations of autoregressive architectures in maintaining long-term accuracy."

We believe this expanded explanation and supporting literature now make our intended message clear. The dynamical setup of our paper parallels the architecture of LLMs (as described in the referenced papers). Difficulty in computing Koopman spectra corresponds to difficulty in developing coherence estimates (see our discussion of approximate eigenfunctions in the paper), which corresponds to an instability in long-term accuracy of autoregressive architectures.

6. *The arctic sea ice example was mostly unclear. Figure 3 has no color scale and I have no idea what the colors mean in the spatial plots. If these are Koopman modes, aren't they complex? How are the complex modes represented in the figures, and how are complex modes linked with ice melt? The same questions exist for Figure 7. On p7 it was stated that decaying (complex) modes "reflect dissipative dynamics, i.e. sea ice melting", but no reasoning was provided for this claim.*

We thank the referee for this comment: it has prompted significant improvements to both the clarity of our figures and the explanation of how Koopman modes relate to the physical interpretation of sea ice dynamics.

Complex Koopman modes and physical interpretation: The referee is absolutely right that Koopman modes are generally complex. In the original submission, we displayed the real part in one figure and the absolute value in another—both of which are common in Koopman and modal analyses (see, e.g., Taira et al., AIAA J. (2017) for fluid flows, or papers on acoustics). However, we agree that the inconsistency was confusing. We have now:

– Used only the absolute value of the Koopman modes in all figures,

– Added consistent colorbars across all spatial plots,

– Explained this choice in the main text.

We have also clarified in the manuscript how complex Koopman modes relate to physical interpretation. Since the input data are real-valued, the eigenvalues, eigenfunctions, and modes occur in complex-conjugate pairs. Nevertheless, their combined effect is real. In particular, take the first KMD described in the section on separation of variables in the main text (for real-valued observables $\mathbf{g}$), and consider one of the terms in the summation in terms of these complex conjugate pairs:

$$\lambda_1^n \phi_1(x_0)\mathbf{g}_1 + \overline{\lambda_1}^n \overline{\phi_1(x_0)}\overline{\mathbf{g}_1}.$$

Write in modulus argument form:

$$\lambda_1 = re^{i\theta}, \quad \phi_1(x_0)\mathbf{g}_1 = \mathbf{R}e^{i\mathbf{\Theta}}.$$

Then

$$\lambda_1^n \phi_1(x_0)\mathbf{g}_1 + \overline{\lambda_1}^n \overline{\phi_1(x_0)}\overline{\mathbf{g}_1} = \mathbf{R}r^n[\exp(in\theta + i\mathbf{\Theta}) + \exp(-in\theta - i\mathbf{\Theta})] = 2\mathbf{R}r^n\cos(n\theta + \mathbf{\Theta}).$$

It follows that r captures growth or decay in time through the power r^n, whereas θ captures oscillation. Hence, eigenvalues (which may be complex) with $|\lambda| < 1$ correspond to dissipative dynamics, such as

long-term sea ice decline, while θ determines the timescale of periodic behavior. The Koopman mode (e.g., via $\mathbf{R}$) encodes spatial structure. In this case, it corresponds to geographically significant regions for the dynamics described by λ. This gives an interpretable way to disentangle complex nonlinear dynamics.[3]

Sea ice decline: Regarding the specific link to sea ice decline, we have added a new plot (in the Supplementary Information) showing the time evolution of the eigenfunctions in question associated with a Koopman eigenvalue λ_j, that satisfy $\phi_j(x_n) \approx \lambda_j^n \phi_j(x_0)$. We only show those with non-negative arguments due to complex-conjugate symmetry, absolute value on the left and real part on the right with the expected periods of oscillation from λ:

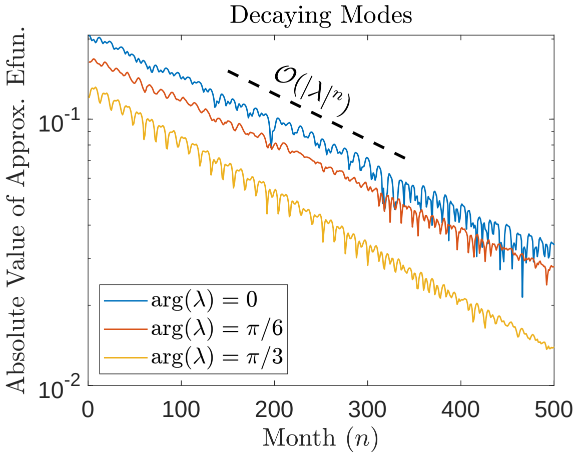

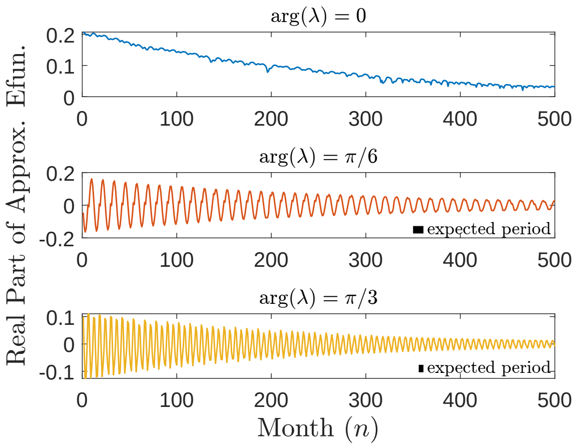

There is a trend consistent with the eigenvalue (in particular $|\lambda| < 1$) and the expected modulation. We have added this plot to the Supplementary Information. We now include this discussion in the main text, which also aligns with a helpful suggestion from Referee 1. This is further supported by the spatial structure of the mode, which highlights geographic regions where the decay occurs. In general, each Koopman mode identifies geographic regions where sea ice concentration exhibits oscillatory, growing, or decaying behavior, as determined by the associated eigenvalue. For instance:

- Modes with eigenvalues near 1 correspond to persistent structures (e.g., long-term means),
- Modes with $|\lambda| < 1$ identify slowly decaying structures (e.g., long-term decline patterns),
- Modes with $\theta \approx \pi/6$ highlight seasonal oscillations.

We also now clarify that, while the data are bounded by 100%, Koopman modes can take on larger values. This is a known feature of non-orthogonal decompositions. For instance, even a simple vector like $(0, 1)$ can be decomposed as a sum of two vectors with larger norm. Because Koopman modes are projections onto eigenfunctions (which need not be orthogonal), the resulting values are not constrained to the input range.

In summary, we have:

- Clarified the role and meaning of complex Koopman modes,
- Ensured consistency in all spatial plots,
- Explained mode evolution (this is also referenced in the caption of Figure 3),
- Expanded the explanation of how different modes relate to physical processes such as decline and seasonal variation.

[3] We have drawn analogies with diagonalizing a matrix and separation of variables in the main text. This decomposition is also analogous to Fourier series (e.g., expressing $\cos(n\theta)$ via exponentials $\exp(in\theta)$ and $\exp(-in\theta)$), but differs in two key ways. First, the eigenvalues λ may have $|\lambda| = r \neq 1$ (for Fourier series, they have modulus 1). If $r < 1$, the above analysis shows that the corresponding component of the signal is decaying in time (i.e., dissipative dynamics if $\mathbf{g}$ corresponds to the state - as it does in our sea ice example where $\mathbf{g}$ is the sea ice concentration over all the grid points). Second, we typically expand a vector of observables (rather than just a single function (observable) as in separation of variables) and hence the coefficients $\mathbf{g}_1$ are vector-valued.

We thank the referee again for this valuable feedback, which has significantly improved both the exposition and interpretability of the sea ice example.

7. *The italic result on the second column of p8 is not particularly informative. For example, (i) the cause of the failure is not described, (ii) examples of failure or indications of the size of the class of maps that fail are missing. If the cause is exotic, or the class of failures is tiny, this is not a particularly interesting result.*

We thank the referee for these excellent questions, which have significantly improved the scope and discussion surrounding our adversarial systems. In response, we have revised the manuscript to directly address these concerns, providing a clearer explanation of both the causes and scope of the failure mechanisms. The relevant theorems are now explicitly referenced in the main text, and we have added new discussion to highlight the significance of the result.

Cause of failure: The failure highlighted on page 8 (of original submission, now in text on page 7) stems from two distinct mechanisms that arise in broad classes of dynamical systems:

- For systems in the class $\Omega_{\mathbb{D}}$, the issue is that there is no a priori way to determine, for a given observable g, whether the available data is sufficient to approximate the Koopman operator acting on that observable. This reflects a well-known feature, e.g., of ergodic systems—namely, that convergence rates are problem-specific and no universal rate exists when sampling relevant correlations [4, 5] (see also our response to detailed comment number 9 below, and the description of the EDMD algorithm now given in the Methods). We refer to this limitation as Challenge **(C1)** in the revised text. Consequently, one cannot design a general-purpose algorithm that adaptively guarantees convergence for arbitrary measure-preserving dynamics—even when data is abundant.
- For systems in $\Omega_{[0,1]}$, the problem arises from non-normality—the lack of commutativity between $\mathcal{K}$ and $\mathcal{K}^*$ (i.e., non-orthogonality of eigenfunctions)—which complicates the separation of transient from asymptotic behavior [6]. This is labelled Challenge **(C2)**.

Both mechanisms highlight fundamental challenges in extracting long-time spectral information from finite trajectory data. They are also encountered in problems beyond Koopman operators.

Are these failures rare or exotic? No—they occur in standard, well-studied classes of dynamical systems. Our constructions use classical examples such as smooth Hamiltonian flows and smooth interval maps, and the same mechanisms can be embedded in high-dimensional systems, other state spaces, or even when we are allowed to sample the derivatives of F etc. The impossibility results (in particular the proof technique) holds for *any class of systems* that has either **(C1)** or **(C2)**. These are not pathological edge cases, but rather illustrative of pervasive challenges in data-driven spectral learning. Importantly, they show that even with large datasets or randomized sampling, no black-box algorithm can universally guarantee success without additional assumptions. We have now made this clearer in the manuscript by highlighting this. Furthermore, based on our extensive experience computing Koopman operators—and consistent with the broader literature, as summarized in Table 1—such issues are common and arise frequently in practical applications.

Why this matters: Our result formalizes key limitations of current approaches: the inability to detect failure without prior system knowledge. This directly motivates the design of new algorithms that succeed under clearly stated assumptions. Indeed, these theoretical insights underpin the provably convergent algorithms we present later in the paper. Such motivation, coupled with a clear objective for its resolution, is essential; without it, the development of new algorithms is exceedingly difficult.

For example, we now write after discussing the algorithm in the section *A universal algorithm with learning guarantees*:

*"Our analysis of **(C1)** and **(C2)** directly leads to this provably convergent algorithm, in contrast to EDMD, which does not converge. The core idea of the algorithm (see Methods) is to use the modulus of continuity to adaptively select the dictionary size N based on the available data M in an averaging procedure similar to Equation (1). Unlike EDMD, however, we do not compute eigenvalues of a finite matrix. Instead, we use this adaptive strategy to compute a correlation matrix that allows us to compute the error metric shown in Figure 1. We search for local minimizers of this function and exploit the measure-preserving property of the system to relate this metric to the distance between a point $z \in \mathbb{C}$ and*

the spectrum. This distance is computed alongside an associated approximate eigenfunction satisfying the coherency condition Equation (5) with ε equal to this distance."

On the "size" of the failure class: Quantifying the "size" of the failure class would require fixing an algorithm and defining a probability measure on the space of dynamical systems—neither of which is natural or canonical in this setting. Our approach is instead aligned with standard practice in complexity theory and numerical analysis: we define the problem class first and then ask whether a general-purpose algorithm exists that performs well across it. This is more informative than algorithm-specific empirical failure rates (as demonstrated with our EDMD experiments - non-convergence of standard techniques is ubiquitous), and allows us to rigorously characterize the limits of learnability. We have written in the text:

"While it is sometimes possible to design algorithms that work for a single known system, this does not reflect the goals of general-purpose learning or complexity classification. Instead, our focus is on identifying the minimal assumptions under which an algorithm can reliably learn across a broad class of systems, a core aim in both computational complexity and ML. "

We now also emphasize that, for the systems in our class, no algorithm can succeed with probability greater than 50%, regardless of how large the dataset is or how it is sampled. This impossibility result is constructive: it not only highlights failure modes but clarifies what assumptions are necessary for successful spectral learning.

In summary, we have clarified both the mechanism and the generality of the failure result. These insights are far from pathological; they guide the development of robust methods and reveal the conditions under which learning is provably possible. We thank the referee for prompting us to sharpen this narrative, which we now make more explicit in the relevant section.

8. *Similarly, also on p8 "...there is no universal rule for determining N... that guarantees convergence... these limits are combined". Presumably this means there is some 'bad class', which is not identified here.*

We thank the referee for raising this important point, which has helped us clarify the distinction between systems where convergence rates are possible and those where it is not, and improve our explanation of the challenges in determining a universal $N(M)$ strategy.

The core issue is that, in general, there is no universal rule for selecting N (dictionary size) as a function of M (data size) that guarantees convergence across all dynamical systems in any class of systems that suffers from **(C1)** outlined above. The reason lies in the system-dependent nature of convergence rates for time-averaged quantities such as correlation functions. For example:

- On periodic or typical quasi-periodic attractors, one typically observes convergence rates of $\mathcal{O}(M^{-1})$ [7].
- For strongly mixing systems, this slows to $\mathcal{O}(M^{-1/2})$ [4].
- But for general ergodic systems, no universal rate exists [5, page 14]. This lack of a uniform rate is a key example of the "bad class" referred to in the text.

More broadly, identifying this "bad class" can be challenging, as it depends on many aspects such as the regularity of the dynamics, geometry of the state space, and the observables. The example in [5], for instance, is technical and non-constructive, which reflects the difficulty of formalising the boundary between systems where convergence can be guaranteed and those where it cannot.

There are a few highly structured settings in which one can rigorously relate N and M to guarantee convergence. A notable example is the recent work of Bandtlow and colleagues [8] on Ruelle resonances for analytic expanding maps on the circle. In this case, the analytic structure allows the two limits—$M \to \infty$ and $N \to \infty$—to be combined into a single limit. However, such examples are non-generic and rely heavily on specific assumptions (e.g., analyticity and spectral gaps).

Nevertheless, it is possible to delineate precisely when convergence becomes possible or impossible:

- Our impossibility result shows that, unless one can control the variability or regularity of F, there is no general-purpose strategy for selecting N as a function of M that ensures convergence. This is formalized in our discussion of Challenge **(C1)** (see previous comment), which we now explicitly

link to this part of the manuscript.

– Conversely, when such control over F is available, convergence becomes feasible. This is demonstrated by the upper bounds and provably convergent algorithms we construct (we consider the setup of a modulus of continuity).

We have revised the text to make this distinction clearer:

*"For instance, for EDMD (see Methods) without the modulus of continuity, one must first take the number of data samples M to infinity to approximate correlations and only then the number of observables N to infinity (even then, EDMD may fail to converge). The convergence rate of time-averaged quantities such as correlations depends sensitively on the system's mixing properties and the regularity of the observable. While polynomial rates hold for certain strongly mixing systems [68,72], Birkhoff's theorem guarantees only convergence without any uniform rate and time averages can converge arbitrarily slowly [69, Chapter 2]. This non-uniformity prevents the formulation of a universal convergence rate or a general- purpose $N(M)$ strategy, unless we can control **(C1)**."*

and

"As another example, Bandtlow et al. [71] construct an EDMD-based method that provably converges with $N = N(M)$ for analytic expanding maps, using analyticity and spectral gaps to unify the two limits."

9. *The final paragraph prior to the section heading "Classifications of learning" on p9: the paragraph sounds interesting, but is so vague it cannot be understood (even though the paragraph claims the necessity of using two limits is "intuitive").*

We thank the referee for highlighting the need for greater clarity, which has allowed us to explicitly explain the necessity of using two successive limits and refine our discussion of the underlying challenges in convergence, greatly improving the precision and motivation of this section.

The key issue is that for systems in the class $\Omega_{\mathcal{X}}^m$, the convergence of time averages does not occur at a uniform rate across different observables, initial conditions, or system parameters. This makes it impossible, in general, to combine the two fundamental limits (e.g., in EDMD)—data size ($M \to \infty$) and model complexity (e.g., dictionary size $N \to \infty$)—into a single limit that guarantees convergence.

To illustrate this more concretely, consider an irrational rotation on the unit circle:

$$F_\beta(x) = x + \beta \mod 1, \quad \text{with } \beta \in \mathbb{R} \setminus \mathbb{Q}.$$

Let $g(x) = \chi_{[0,1/2)}(x)$, the indicator function of the first half of the circle. The Birkhoff time averages,

$$A_n(x) := \frac{1}{n} \sum_{k=0}^{n-1} g(F_\beta^k(x)),$$

converge to the space average (1/2) for almost every initial condition. However, there is no uniform bound on the rate of convergence, and for some β (e.g., Liouville numbers, which are real numbers that can be approximated extremely closely, i.e., at an exponential rate, by rational numbers - in fact the set of bad β has measure 1 so is typical), convergence can be arbitrarily slow [9, Chapter 2] (this is the primary motivating example throughout chapters 1 and 2 of that book, convergence rates are the focus of Chapter 2). This lack of uniformity persists even in this simple highly regular system and illustrates the need to decouple the data limit and the model complexity limit.

This is the underlying reason why one must first take the number of data points to infinity (to ensure accurate statistical averages) before increasing the model complexity (e.g., via dictionary size N) to capture richer spectral features. This phenomenon also occurs in Ulam's method or spectral approximations of transfer operators (see the discussion connected to Table 1 of the paper).

We now make this connection explicit in a revised paragraph. In fact, following the suggestion of the referee for enhanced clarity, we have now gone through each of the classes in turn:

"First, let $\Omega_{\mathcal{X}}^\alpha$ denote systems whose dynamics have a known smoothness quantified by a modulus of continuity α. For these systems, there exist learning algorithms that depend on two parameters, n_1

and n_2, and reliably use snapshot data to approximate the dynamics. These algorithms converge in two successive data limits ($n_1 \to \infty$ and then $n_2 \to \infty$) to the spectrum $\mathrm{Sp}_{\mathrm{ap}}(\mathcal{K}_F)$ for all systems F in $\Omega_{\mathcal{X}}^{\alpha}$."

*"Second, let $\Omega_{\mathcal{X}}^{m}$ denote measure-preserving systems with continuous dynamics. Again, algorithms exist that achieve convergence to $\mathrm{Sp}_{\mathrm{ap}}(\mathcal{K}_F)$ in two successive data limits for all systems in this class. The necessity of using two successive limits arises naturally. In Equations (7) and (8), challenge **(C1)** implies that convergence rates of the underlying sums cannot be uniformly controlled [69], a fact well known for mixing systems, among others. Thus, one must first take the large-data limit ($M \to \infty$), then the limit of dictionary size ($N \to \infty$); these limits cannot, in general, be combined or reversed. An implication of our analysis is that, for any algorithm (not just EDMD), no universal rule linking M and N guarantees convergence when both grow together."*

*"Finally, consider the most general scenario, $\Omega_{\mathcal{X}}$, consisting of all continuous systems without further assumptions. Remarkably, reliable algorithms still exist, but they require three successive data limits to convergence to the spectrum $\mathrm{Sp}_{\mathrm{ap}}(\mathcal{K}_F)$ for all $\Omega_{\mathcal{X}}$: increasing the number of snapshots ($M \to \infty$), expanding the dimensionality of the subspaces ($N \to \infty$), and refining coherence estimates (take the regularization parameter $\varepsilon \downarrow 0$ in Equation (5)) into spectra. Without assumptions on the underlying system, no simpler two-step algorithm can achieve guaranteed convergence (Theorem 2.9 of the S.I.)). **(C1)** and **(C2)** each cost a limit, illustrating the intrinsic difficulty of learning general dynamical systems."*

This is also supported by the now-explicit description of the EDMD algorithm in the Methods, our revisions in response to point 8 of the referee, and the example of estimating the average energy E of an oscillating system from discrete measurements of instantaneous energy e at time n.

We are grateful to the referee for prompting this clarification, which has improved both the precision and motivation of this section.

10. *p11, 4 paragraphs prior to Discussion heading: this is supposed to "establish" and "illustrate" a result about needing three successive limits, but what follows is a brief set of statements with no reasoning (or illustration).*

    We thank the referee for highlighting the need for a clearer illustration, which has led us to better clarify the reasoning behind the three successive limits and directly reference the relevant theorem from the Supplementary Information, improving the explanation and its integration into the main text.

    The result referred to is proven in the Supplementary Information and shows that, in the absence of structural assumptions on the dynamical system, three limits are necessary to compute the spectrum of the Koopman operator from data. These limits arise from the need to overcome the two key challenges **(C1)** and **(C2)** identified earlier. Each of these imposes a cost in terms of limiting procedures.

    As an illustration, for a fixed regularization parameter $\varepsilon > 0$—interpreted as a temporal coherency threshold—we can compute the ε-pseudospectrum:

$$\mathrm{Sp}_{\mathrm{ap},\varepsilon}(\mathcal{K}) = \{\lambda \in \mathbb{C} : \text{there exists a normalised observable } g \text{ with } \|\mathcal{K}g - \lambda g\| \leq \varepsilon\}$$

    in two limits, even in the presence of **(C1)** (as now outlined in the Methods section). However, to recover the true spectrum $\mathrm{Sp}_{\mathrm{ap}}(\mathcal{K})$, one must also take the limit $\varepsilon \downarrow 0$. Without overcoming **(C2)**, it is impossible to obtain a convergence rate of $\mathrm{Sp}_{\mathrm{ap},\varepsilon}(\mathcal{K})$ to $\mathrm{Sp}_{\mathrm{ap}}(\mathcal{K})$ as $\varepsilon \downarrow 0$. Hence, three limits are required.

    We have clarified this explanation in the main text, now placed earlier in the section "To Infinity and Beyond," and have removed the previous standalone subsection on the impossibility result. We have also provided a more detailed reasoning in the Methods section where we discuss the algorithms. The relevant theorem from the Supplementary Information is now referenced directly to support this claim. This can be seen in the paragraph in red in the response to comment number 9.

11. *The Discussion opens with "We have developed a powerful technique...", but no technique has been presented yet. Similarly, in the Discussion: "we designed new optimal algorithms", "introduced multi-data-limit algorithms", "This work represents a rare instance in machine learning...". None of these items have been defined in the manuscript so far.*

We thank the referee for pointing out how to ensure that the Discussion best summarizes the main contributions and their broader significance. In light of the revisions described above, these contributions are now more clearly defined and signposted throughout the manuscript. Specifically:

- "We have developed a powerful technique..." refers to the construction of adversarial dynamical systems that yield lower bounds on the difficulty of learning spectral quantities from data. The construction is introduced in the *Introduction* and developed in detail in the section titled *Adversarial systems* (see the discussion of **(C1)** and **(C2)**). This technique underpins the first impossibility results in data-driven dynamical systems, and is illustrated in Figure 11, now explicitly referenced in the *Methods* section.
- "We designed new optimal algorithms": Following suggestions from both referees, we have included detailed pseudocode in the Supplementary Information and clarified the description of these algorithms in the *Results* and *Methods* sections. These algorithms, introduced in the section *A universal algorithm with learning guarantees*, provably compute the spectrum of the Koopman operator from snapshot data and achieve convergence under mild assumptions. They match the lower bounds revealed by the adversarial systems and achieve convergence with verification under mild conditions.
- "Introduced multi-data-limit algorithms": This refers to the same family of algorithms, which are specifically constructed to handle multiple successive data limits. We have clarified this terminology in the revised manuscript and linked it more explicitly to the classification results.
- "This work represents a rare instance in machine learning": This statement refers to the classification results discussed in the Results section, where we provide both upper and lower bounds on problem difficulty in the Solvability Complexity Index (SCI) hierarchy.

To avoid ambiguity, we have now clarified these links in the revised Discussion section and ensured that the terminology used there aligns more explicitly with definitions and section headings in the main text:

*"We have developed a powerful technique—namely, the construction of adversarial dynamical systems (see §Adversarial systems and Figure 11)—to establish the first impossibility re- sults in the field of data-driven dynamical systems. We identified conditions (**(C1)** and **(C2)**) for which no sequence of randomized algorithms (e.g., randomly sampling trajectories) can succeed with probability greater than 50%. These failures are not rare, but represent fundamental barriers. These barriers provided insights, which we used to develop a suite of new algorithms (Algorithms 1–7 in the S.I.) that achieve optimal performance and are provably convergent. These algorithms produce trustworthy, verifiable outputs, and include what we term 'multi-data-limit' methods (see Figures 7 and 8). By linking dynamical systems theory with the foundations of computation, we established a computational complexity framework for data-driven dynamical systems. This framework clarifies the inherent limitations of using finite data to analyze complex dynamics while also highlighting new algorithmic possibilities with broad applications. By combining a new theoretical complexity framework with practical algorithmic advances, our work provides a rare instance in ML of a synthesis between rigorous theory and application."*

All of these points are addressed in the Results section, and Figures 7 and 8 summarize the classification results. We have updated the captions of Figures 7 and 8 to clarify that each classification includes both an explicit algorithm (upper bound) and an impossibility result via adversarial dynamical systems (lower bound on difficulty). We are grateful to the referee for prompting this clarification.

12. *On p13, an algorithm is described, but it is not clear if this is a new algorithm appearing for the first time in this manuscript or not. The lack of citation strongly suggests it must be completely new, but in that case, the authors should introduce it in far more detail. I note also that as a data-driven method, this approach seems to require 3 limits, which the authors railed against toward the beginning of the manuscript.*

We thank the referee for these helpful suggestions on how to make the algorithm advances much clearer. The algorithm described is indeed new, as are several others introduced in this work. Each algorithm corresponds to an upper bound for one of the dynamical system classes summarized in Figure 7, which outlines the computational complexity landscape. To signpost them and address the referee's concern,

we now provide detailed pseudocode for all algorithms in the Supplementary Information. In addition, the Methods section has been revised to present each class alongside its associated algorithm, and a high-level summary is now included in the Results section.

Regarding the number of limits: The three-limit requirement applies only to the most general class $\Omega_{\mathcal{X}}$. For more structured subclasses, fewer limits suffice. For example, in $\Omega_{\mathcal{X}}^{\alpha,m}$—where system regularity and Koopman non-normality are controlled—only a single limit is needed, and our algorithms provide explicit error bounds even with finite dictionaries. We have clarified this point in the revised Methods section, and when discussing the general class $\Omega_{\mathcal{X}}$.

13. *The experimental details on pp 13 and 14 for three complicated dynamical systems were inadequate. If this is a new approach, one could spend an entire paper analyzing it and describing strengths/weaknesses through detailed and thorough examples. The Duffing example did not explain (this is not a complete list): the choice of trajectory length 5, why k-means clustering was used, the value of L. The EDMD description, which is meant to be the main comparator, was confusing. I could not find an algorithm in Supp Section 2.1 as claimed. Fluid flow example: why is Fig 11 interesting? Why is the pure point spectrum in Fig 2 interesting? Sea ice example: the whole example is lacking in detail. Details of embedding are lacking. How do you know your embedding is good?*

We thank the referee for these helpful suggestions that have helped us greatly improve the experimental details. We have substantially expanded the description of all examples, added missing details, and provided new diagnostic figures and results. The Supplementary Information now contains explicit pseudocode for the algorithms, and the Methods now contains data generation protocols, and dictionary construction procedures, ensuring full reproducibility. We also emphasize that all results are supported by rigorous convergence proofs, and our algorithms come with a posteriori error bounds that validate their empirical performance across diverse examples.

Choice of systems: While some of the examples are inherently complex (e.g., Arctic sea ice example), many were chosen for their conceptual simplicity and pedagogical value. For instance, any Hamiltonian system must be at least two-dimensional, and the Duffing oscillator (with $\gamma = 0$) is among the simplest such examples. Since continuous dynamical systems cannot exhibit chaos in two dimensions, the added examples of the Lorenz and Rössler systems (see the additional figure below) in three dimensions are natural choices, as they are two of the most elementary (and indeed famous) examples of chaotic behavior. The flow past a cylinder provides a textbook example of periodic behavior, while the cavity flow demonstrates a transition from steady to chaotic dynamics. Our goal is to balance theoretical examples with real-world systems to showcase the breadth and robustness of the approach.

Duffing oscillator: We now include a full description of both EDMD and our algorithm, along with their data generation and dictionary construction. The parameter L is not used in this example—it refers to a different algorithm based on the RAGE theorem applied in the cavity flow example. We have clarified this in the revised text and signposted algorithm references clearly throughout. In that algorithm L is a parameter taken to infinity as an algorithm converges, so there is no choice of L required (our results proved that this eigenvalue extraction problem cannot be solved in fewer limits).

We have collected data and used k-means clustering in an analogous way to the original EDMD paper. This is a standard method to choose centers for radial basis functions based on data. The idea is that the clustering ensures the centers are not too highly concentrated and that the resulting dictionary provides a good representation of the dynamics over the data points. The choice of 5 (used to find the centers) is based on reducing the condition number of the basis. We have now described this process in the Supplementary Information and provided the following figure which demonstrates it:

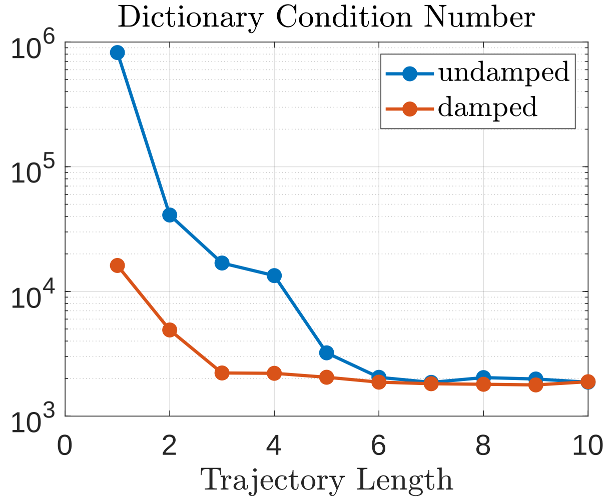

In the Methods section, we have now explicitly described the process for constructing this choice of dictionary (including the bandwidth selection for radial basis functions). The only requirement of the dictionary is that, as we use more basis functions, the projections onto the span of the dictionary converge in the so-called strong operator topology. This amounts to the projections acting on any fixed observable converging to that observable. This is a very weak condition that is satisfied for all of the examples in this paper (even if it did not hold, we would still be able to use our error bounds in Algorithm 1 to bound the distance to the spectrum).

We have also added figures in the Supplementary Information to demonstrate the convergence of our algorithm as more data is collected and as the size of the dictionary is increased. The corresponding figures are:

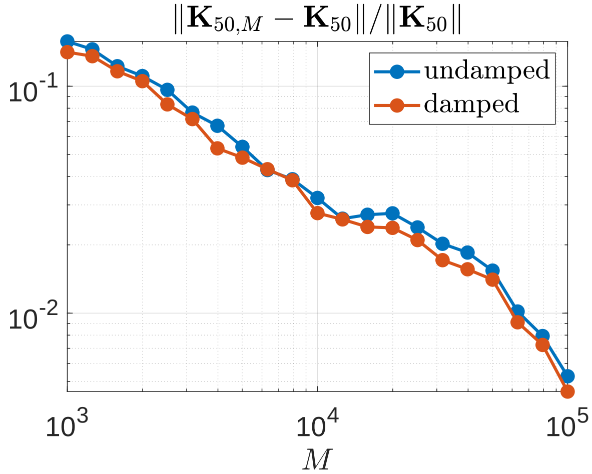

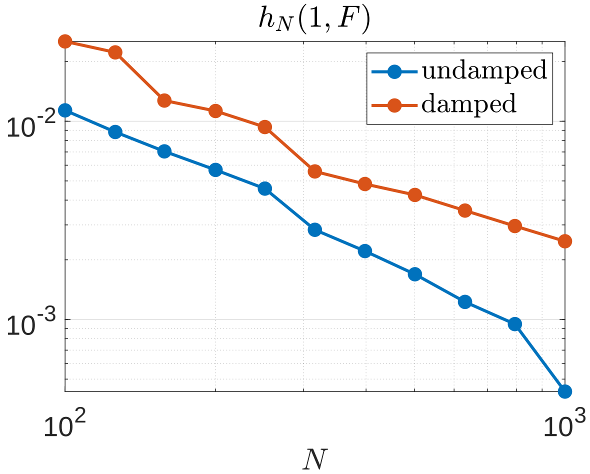

The left plot shows convergence of the EDMD matrix approximations (in the matrix Frobenius norm) as more data is collected. The right plot shows the convergence of functions that are used to approximate spectral distances (see the description in the Methods). As discussed above, these are good convergence results (and small errors) for data-driven spectral problems.

Other dynamical systems: To demonstrate the generality and broad applicability of our results (including across many different types of dynamical systems and qualitatively different trajectory data), we have also provided a figure that shows EDMD and our algorithms applied to several further dynamical systems:

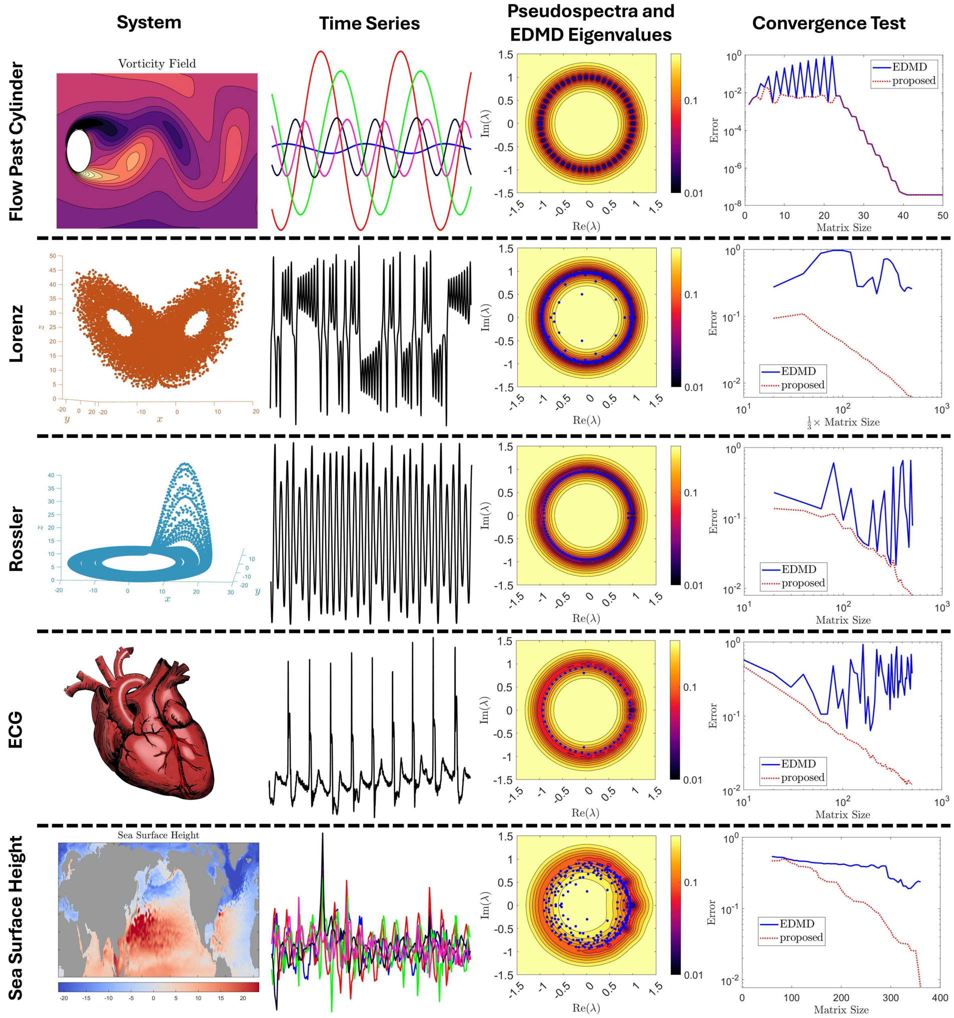

The information required to reproduce our results consists of three parts: the dynamical system, the data collection procedure, and the dictionary of observables used. These are explicitly given in the Supplementary Information. In all cases, our algorithms and EDMD use the same data and the same dictionary to ensure a fair comparison. The figure demonstrates a wide range of dictionary choices, data collections, and types of dynamical systems. The cylinder flow and Lorenz are further used in Figure 1 to make the bottom panel clearer.

Cavity flow and pure point spectrum: This is an excellent question and discerning eigenvalues versus continuous spectrum is a fascinating topic. We have now added a paragraph at the start of the section "Learning eigenpairs and latent spaces is hard" explaining why the pure point spectrum is of general interest:

"As a final problem, we consider the complexity of determining the spectral type, i.e., eigenvalues versus continuous components (see bottom panel of Figure 1). Spectral types distinguish between recurrent patterns (periodic or quasiperiodic oscillations) and more chaotic or mixing behavior, based on how the system spreads energy over frequencies [83, page 45]. They play a key role in applications such as fluid mechanics [84], anomalous transport [85], and analysis of trajectory invariants and exponents [86], and are particularly important in reduced-order modeling [7, 87]. We discussed their role in the context of our cavity flow problem in Example 1.2."

Spectral types distinguish between recurrent patterns (like periodic or quasiperiodic oscillations) and more chaotic or mixing behavior, based on how the system spreads energy over frequencies pure point spectra corresponds to quasiperiodic motion, in contrast to the continuous spectrum, which is associated

with decay of correlations, and it plays a fundamental role in many applications that we now list. For example, the so-called RAGE theorem that we use is a fundamental result in quantum mechanics that lies at the heart of phenomena such as Anderson localization. See, for example, the book *Random Operators: Disorder Effects on Quantum Spectra and Dynamics* by Aizenman and Warzel.

If the Koopman operator $\mathcal{K}$ admits an orthonormal basis of eigenfunctions $\{\phi_j\}$ with eigenvalues $\{\lambda_j\}$ (i.e., only eigenvalues and no continuous spectra), then the evolution of any observable g can be described by

$$g(x_n) = [\mathcal{K}^n g](x_0) = \sum_j \lambda_j^n \langle g, \phi_j \rangle \phi_j(x_0).$$

Each eigenvalue can be written as $\lambda_j = \exp(i\theta_j)$ for $\theta_j \in [0, 2\pi)$. The dynamics of g are *periodic* if $\{\theta_j/(2\pi) : \langle g, \phi_j \rangle \neq 0\}$ is a finite subset of $\mathbb{Q}$ (i.e., there is a fundamental frequency). Otherwise, the motion is called *quasiperiodic*. More generally, systems may exhibit continuous spectra (as occurs in our cavity flow example at high Reynolds number) which correspond to integrals over a continuum of frequencies [10]. Isolating the pure point spectrum allows us to disentangle the quasiperiodic structure from mixing behavior and provides insight into the asymptotic dynamics in the system's state space by inspecting the Koopman spectrum. When introducing approximate eigenfunctions, we have also provided some further use of eigenfunctions:

"Approximate eigenfunctions also encode key dynamical features of the system, such as the global stability of equilibria. The contours (or level sets) of these functions highlight key structures in the system's dynamics, such as regions that behave independently over long times, pathways along which the system evolves, and surfaces where states settle into long-termbehavior at the same rate [20, 49]."

In fluid flows, discrete Koopman eigenvalues typically indicate quasiperiodic motion, while a broadband spectrum is often interpreted as a signature of chaotic dynamics [11, 12, 13]. This structure of the spectrum can also be used to study sudden qualitative changes in the system's behaviour, such as the geometry of the attractor, as the Reynolds number increases. For example, in the main text we now describe (in the sea ice example) how the Koopman eigenvalues and eigenfunctions have a multiplicative structure: if $\mathcal{K}g_1 = \lambda_1 g_1$ and $\mathcal{K}g_2 = \lambda_2 g_2$, then $\mathcal{K}(g_1 g_2) = \lambda_1 \lambda_2 g_1 g_2$. This structure (in particular, the pure point spectrum) describes the geometry of the attractor. For a limit cycle (a stable repeating loop), there is a single fundamental eigenvalue λ and the spectrum consists of its integer powers λ^n. This structure is visible, for instance, in the spectrum of the flow past a cylinder (see new Figure 9), which corresponds to low Reynolds number. For a torus (three-dimensional ring-shaped) attractor, there are two basic eigenvalues λ and μ and the spectrum consists of products $\lambda^m \mu^n$. In this way, the geometry of the attractor in state space (e.g., whether the attractor is shaped like a limit cycle or a torus in the state space) can be inferred from the structure of the spectrum. In systems with mixed spectra, such as our cavity flow at higher Reynolds numbers, the Koopman spectrum contains both discrete (quasiperiodic) and continuous (chaotic) components. This type of attractor is referred to as skew-periodic in the dynamical systems literature [14], which we have also intuitively explained in the main text.

The evolution of the Koopman spectrum in Figure 10 of the main text (for the cavity flow) reveals a sequence of bifurcations (sudden qualitative changes in the system's behaviour) as the Reynolds number increases. For $Re \leq 10000$, the cavity flow, driven by a regularized lid velocity, converges to a steady laminar solution, corresponding to a fixed point in the state space of the flow. Just above $Re = 10000$, this steady solution becomes unstable, and the system transitions to a time-periodic flow, which remains stable up to $Re = 15000$. The fundamental frequency of the periodic flow decreases with increasing Reynolds number. At $Re \geq 15000$, a second bifurcation occurs, and the flow becomes quasiperiodic. The basic frequencies of the quasiperiodic motion also decrease with Reynolds number, until around $Re = 18000$, where a third bifurcation is observed: the portion of kinetic energy associated with the continuous spectrum rises sharply to a few percent. This continuous component continues to grow, and by $Re \geq 22000$, the state space dynamics exhibit no quasiperiodic structure: the Koopman spectrum consists solely of continuous components, indicating fully chaotic dynamics.

Physically, the evolution of the Koopman spectrum in Figure 10 provides a picture of the transition to chaos that is consistent with the theory of Ruelle and Takens [15]. According to this theory,

a chaotic state can emerge after one or two bifurcations from an initially stable steady flow. The Koopman spectrum serves as a generalized spectral tool for the study of bifurcations: it enables clear quantification of energy contributions from both truly periodic components and the continuous spectrum in deterministic flows. Moreover, it connects the discrete part of the spectrum to the geometry of the state space and the structure of the flow domain.

In addition to the general paragraph in “Learning eigenpairs and latent spaces is hard”, we have now added this physical description in Example 1.2, and commented on the transitions as Re is increased in Figure 10 in the Methods section. We have also made the bottom panel of Figure 1 a lot clearer to articulate the importance of spectral type.

To demonstrate the different spectra type structure and the attractors, in the Supplementary Information, we also plot the extracted Koopman eigenfunctions on the attractor in state space, with full details provided in the relevant section. For $Re = 13000$, the attractor is a limit cycle. The plot below shows the eigenfunction corresponding to the fundamental eigenvalue λ (left) and its square λ^2 (right). The horizontal axis represents the kinetic energy and the vertical axis the time-delayed kinetic energy. This provides a coordinate system in which to represent the limit cycle:

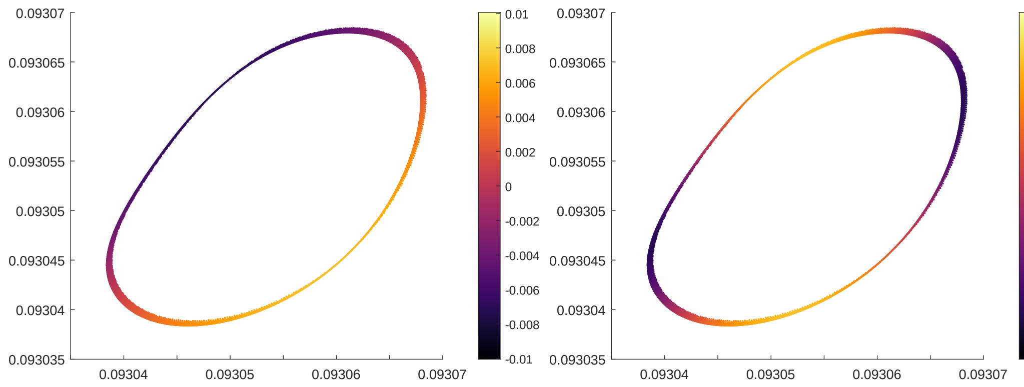

The color represents the real part of the eigenfunction at each point of this statespace. The doubling in frequency from left to right is consistent with a Koopman spectrum consisting of powers $\{\lambda^n\}$. For $Re = 16000$, the attractor is a torus corresponding to two fundamental eigenvalues, λ and μ (quasiperiodic dynamics). We plot the corresponding eigenfunctions in three dimensions, with coordinates given by the kinetic energy and two time-delayed kinetic energies:

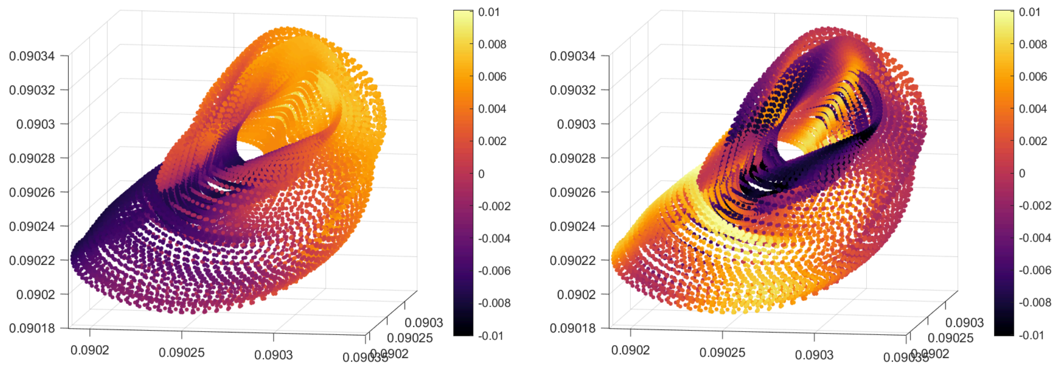

The Koopman eigenfunctions show the directions on the torus where the evolution is linear and periodic. For $Re = 16000$, the attractor is skew-periodic, with a strong discrete spectrum (in the energy sense) consisting of two basic frequencies and a relatively weak continuous component. The corresponding eigenfunctions are plotted as functions of the kinetic energy and two time delays, as before. These eigenfunctions provide geometric slices on which the motion is purely quasiperiodic:

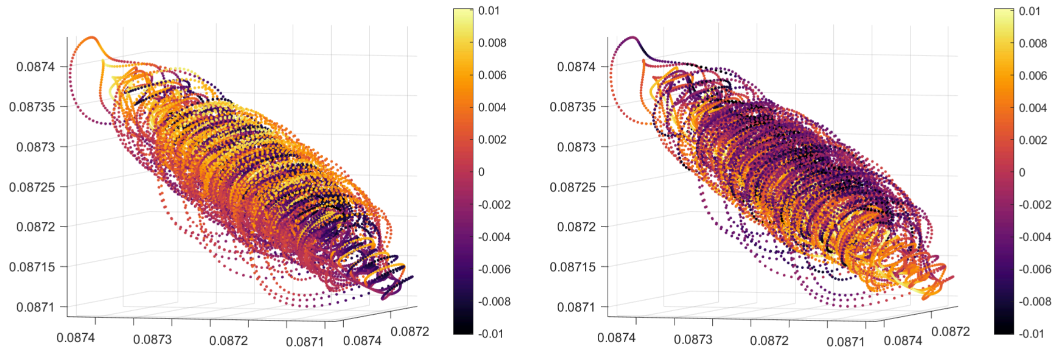

Arctic sea ice: When describing the example, we have now provided much more detail, including how the Koopman modes provide physically relevant information, what error metrics we use, and the interpretations for Arctic sea ice (see the list above near the start of our response to Referee 2). In the Methods section, we have provided a much clearer description of all the details, including how the data is sourced, processed, organized with delay embedding and an explicit description of how the dictionary is constructed, along with the forecasts, benchmarks, error metrics, and EDMD. The revised Methods section now describes each step explicitly.

The question of how we know the embedding is an excellent one and we thank the referee for raising it. A key strength of our algorithm is that it comes with rigorous error bounds (show for Duffing and the range of other examples above), which allow us to test a posteriori whether a given choice of dictionary performs well—without requiring comparison to held-out data or forecasts. This is extremely useful. We found that our chosen dictionary produces small errors when approximating the Koopman spectrum. It would be interesting future work to combine our error bounds with other methods of learning a dictionary (e.g., neural networks) but this is beyond the scope of the present manuscript (that we now briefly mention). We have now discussed this a posteriori dictionary verification in the Methods:

"A key strength of our algorithm is its rigorous error bounds (e.g., Figures 1 and 9), which enable direct evaluation of dictionary performance by tracking the error—without needing held-out data or forecasts. This allows us to verify that our chosen dictionary yields small errors when approximating the Koopman spectrum. Future work will explore integrating neural network embeddings [29] with our error-bound framework."

References

[1] D. Boffi. Finite element approximation of eigenvalue problems. *Acta Numerica*, 19:1–120, 2010.

[2] Ivo Babuška and John Osborn. Eigenvalue problems. In *Handbook of Numerical Analysis*, volume 2, pages 641–787. Elsevier, 1991.

[3] Françoise Chatelin. *Spectral Approximation of Linear Operators*. Society for Industrial and Applied Mathematics, 2011.

[4] A. Kachurovskii. The rate of convergence in ergodic theorems. *Russian Mathematical Surveys*, 51(4):653–703, 1996.

[5] U. Krengel. *Ergodic theorems*. Walter de Gruyter, 2011.

[6] L. N. Trefethen and M. Embree. *Spectra and Pseudospectra: The Behavior of Nonnormal Matrices and Operators*. PUP, 2005.

[7] Igor Mezić and Fotis Sotiropoulos. Ergodic theory and experimental visualization of invariant sets in chaotically advected flows. *Physics of Fluids*, 14(7):2235–2243, 2002.

[8] Oscar F. Bandtlow, Wolfram Just, and Julia Slipantschuk. EDMD for expanding circle maps and their complex perturbations. *Appl. Comput. Harmon. Anal.*, to appear.

[9] Lauwerens Kuipers and Harald Niederreiter. *Uniform distribution of sequences.* Courier Corporation, 2012.

[10] I. Mezić. Spectral properties of dynamical systems, model reduction and decompositions. *Nonlinear Dyn.*, 41(1-3):309–325, 2005.

[11] H. Swinney and J. Gollub. The transition to turbulence. *Physics today*, 31(8):41–49, 1978.

[12] Doyne Farmer, James Crutchfield, Harold Froehling, Norman Packard, and Robert Shaw. Power spectra and mixing properties of strange attractors. *Annals of the New York Academy of Sciences*, 357(1):453–471, 1980.

[13] Jérémy B. et al. Experimental investigation of global structures in an incompressible cavity flow using time-resolved PIV. *Experiments in Fluids*, 50(4):905–918, 2011.

[14] H. Broer and F. Takens. Mixed spectra and rotational symmetry. *Archive for rational mechanics and analysis*, 124:13–42, 1993.

[15] D. Ruelle and F. Takens. On the nature of turbulence. *Les rencontres physiciens-mathématiciens de Strasbourg-RCP25*, 12:1–44, 1971.

Response to Referee

Changes in the paper are marked in red. We have also tweaked the title and simplified panel (d) of figure 1, as outlined in our cover letter. All other changes consist of grammatical and spelling corrections.

Comments and suggestions made by the referee are addressed below.
(*referee comments in italics*, our response in normal font)

I would like to thank the authors for their thorough responses to the Referee comments and associated revisions of the manuscript. In my opinion, the review comments have been well-addressed and the manuscript is publishable in Nature Communications after minor revision. I only have a few remaining comments.

We thank the referee for their thoughtful comments, which have been very helpful in improving the clarity and quality of our manuscript. We greatly appreciate the time and care taken in reviewing our work. In what follows, we have addressed each of the referee's points in detail and have made corresponding revisions to the manuscript where appropriate.

1. *SI Figure 9*

   - *I would suggest using calendar dates (similarly to Figure 3 in the main text) in the x-axes rather than month numbers.*

     Thank you for this suggestion, we have now done this.

   - *From the point of view of making connections with climate change, I found it somewhat counter-intuitive that the* ϕ_{decay} *time series exhibits a near-monotonic reduction of negative slope as time (n) increases. I understand that this behavior is consistent with the corresponding Koopman eigenvalue, but as far as I know sea ice loss has been slower in the 1970s and 1980s, then underwent acceleration in the 1990s to early 2000s, and then entered a period of general slowdown or "hiatus" lasting through the present. This non-monotonic trend behavior is to some extent visible in the moving mean curve in Figure 3. The revised manuscript states that the time series of* ϕ_{decay} *provides "strong evidence of a connection to sea ice loss from climate change", but it is not clear how to reconcile the near-monotonic behavior of* ϕ_{decay} *with the non-monotonic rates of sea ice loss observed over the past few decades. In summary, while I have no doubt that there is a connection between* ϕ_{decay} *and climate change, the details of that connection may be quite nuanced. I would therefore be hesitant to call the evidence strong without further justification of the differences between* ϕ_{decay} *with known trend patterns as noted above.*

     We have softened the wording and referred to a new figure addressing the sub-bullet point below. Since the full state is expressed as a sum of modes, the near-monotonic behaviour of (the absolute values of) ϕ_{decay} does not contradict the non-monotonic rates of sea-ice loss observed over the past few decades. As the referee points out, this connection may indeed be quite nuanced. We have also clarified this point in the main text.

   - *It might be worthwhile examining the behavior of* ϕ_{decay} *computed separately using the first half and second half of the 1979–2021 analysis interval. Would the eigenfunction time series slopes be mutually consistent between these calculations and the one from the full analysis interval?*

     Thank you for this suggestion. We have now replaced the original figure with precisely this since we think it presents a clearer picture. We note that there appears to be a slight variation in the slope (consistent with the results of Figure 3), but that the slopes are largely consistent with the full time interval analysis.

2. *p. 5, "Hidden modes with nonzero* $\arg(\lambda)$ *can be interpreted as seasonal patterns modulated by this*

long-term trend.": I believe a reference to [28] (who made a similar observation for eigenfunctions computed from SST data) would be appropriate here; see, e.g., subsection "Response of seasonal cycle of precipitation to climate change" where these modes are referred to as "trend combination modes".

Thank you for this suggestion. We have now added this reference.

3. *In their response to comment 4(c) in my review the authors mention that the periodic baseline model considerably outperforms the persistence model. I wasn't sure how to fully interpret this statement since, by construction, the persistence model has zero error at the forecast initialization time, and would thus outperform all models shown in Figures 4 and 5. Presumably, the persistence forecast loses skill more rapidly than other models, but including it as a means of gauging improvement in rate of skill loss that these models offer could still be helpful. Did the authors mean that the persistence forecast considerably underperforms the other models even for lead times as short as 1 month? If so it would be useful to include that information.*

   Yes, we meant for lead times as short as one month. To include this information, we have now added the (monthly) persistence model to Figure 4, and altered that experiment according to our reply to the referee's next set of comments.

4. *Thank you for clarifying that "entire sea ice concentration" corresponds to sea ice concentration fields (comment 4(a) in my review) and the error metric used to assess forecast skill in Figure 4 is given by*

$$\text{Error} = \frac{\|\mathbf{x} - \mathbf{x}_{\text{rec}}\|^2_{l^2}}{\|\mathbf{x} - \mathbf{x}_{\text{per}}\|^2_{l^2}}.$$

   The results in Figure 4 indicate that this error metric does not saturate to the baseline climatological forecast over prediction horizons extending out to more than five years (with either DMD or the proposed method). This is considerably longer than predictability limits expected for sea ice dynamics, where, for example, skillful forecasts of September pan-Arctic sea ice area to a few months in advance (let alone several years in advance) would be considered a success. I am therefore concerned that the error metric in (1) does not clearly reflect when loss of skill has taken place.

   - *I presume that the l^2 norms in (1) correspond to integration over spatial gridpoints, is that right? If so, can the authors confirm that these norms are approximations of area integrals over the Arctic spatial domain? The main text states that each gridpoint has a fixed area of 625 km^2 but as far as I could tell (apologies if I missed the relevant details) there is no information provided about the meridional extent of the domain or the longitude–latitude sampling employed.*
   - *It is not clear how Error from (1) is averaged to produce the results in Figure 4. Is Error averaged over the initial conditions, or the numerator and denominator are averaged separately before computing the ratio?*
   - *Assuming that $\|\cdot\|_{l^2}$ corresponds to area integrals, wouldn't it be more interpretable to compute σ^2 as the time average of $\|\mathbf{x} - \mathbf{x}_{\text{per}}\|^2_{l^2}$ at lead time 0 over the training dataset, and show time averages of*

$$\text{Error}_{\text{rec}} = \frac{\|\mathbf{x} - \mathbf{x}_{\text{rec}}\|^2_{l^2}}{\sigma^2}, \quad \text{Error}_{\text{per}} = \frac{\|\mathbf{x} - \mathbf{x}_{\text{per}}\|^2_{l^2}}{\sigma^2}$$

     over initial conditions in the verification dataset? In this way it would be clear (i) how the errors of both "rec" and "per" forecasts compare to the climatological variance σ^2 relative to the seasonal cycle; and (ii) when the error of $\mathbf{x}_{\text{rec}}$ has saturated to that of $\mathbf{x}_{\text{per}}$. In the current Figure 4, (i) is missing.
   - *A typical pan-Arctic domain will contain gridpoints where predictability of sea ice concentration is trivial over certain months of the year (e.g., winter months at a sufficiently high latitude or summer months at a sufficiently low latitude). Given that, it may be more informative to report errors for the aggregated pan-Arctic sea ice area or extent (e.g., using a modification of (2)) rather*

than spatially averaging pointwise errors. Pan-Arctic sea ice area or extent are also commonly used target variables in predictability studies which would facilitate comparison of the results in this paper with other works in the literature.

We thank the referee for these helpful comments. To address these concerns, we have made the following changes.

First, when computing the squared l^2 norm between the sea ice concentration and a given reconstruction, we restrict attention to an active grid-cell region (defined separately for each calendar month), which shrinks in summer and expands in winter according to the maximum observed sea ice extent for that month. These active regions are identical to those used in [63]. We also confirm that these norms approximate area integrals over the Arctic spatial domain.

Next, we computed σ^2 as the average of $\|\mathbf{x} - \mathbf{x}_{\text{per}}\|_{l^2}^2$ (evaluated over the active regions) at a lead time of one month over the period 2005–2015 (we use a one-month lead time to avoid the zero-error baseline at initialization). All errors were then normalised by σ^2, and Figure 4 now shows the average value of $\|\mathbf{x} - \mathbf{x}_{\text{rec}}\|_{l^2}^2/\sigma^2$ for each reconstruction method, averaged over initial conditions during 2005–2015. We also include the (monthly) persistence model as a baseline for comparison. Since Figure 5 already reports a complementary binary classification metric, we chose to retain the spatially integrated l^2 error here. We thank the referee for these suggestions, which have substantially improved the clarity and interpretability of this experiment. With this revised normalisation, the reconstruction errors saturate on interannual timescales, consistent with known predictability limits of sea ice dynamics.

Adversarial Dynamical Systems Reveal Limits and Rules for Trustworthy Data-Driven Learning

Review

This paper presents a study on algorithmic barriers to data-driven approximation (learning) of dynamical systems. The focus is on learning via spectral approximation of Koopman operators on L^p spaces. Building on a program developed by the authors on the Solvability Complexity Index (SCI), a number of theoretical results are derived on the impossibility of approximating the spectrum of the Koopman operator for a class of dynamical systems using a single sequence of algorithms, as well as learnability via multi-parameter families of algorithms using iterated limits. Based on these results, an SCI index is introduced characterizing the efficiency of a given family of algorithms in terms of the number of iterated limits it requires (compared to the minimum bound for the given system class). The paper also includes a suite of numerical examples involving the Duffing oscillator, a cavity flow, and Arctic sea ice dynamics.

In my assessment this is an interesting and well-written paper that represents a significant contribution in the field of data-driven approximation of dynamical systems that is suitable for publication in *Nature Communications*. I do believe, however, that aspects of the manuscript, particularly aspects related to its positioning in the broader context of learning methodologies, would benefit from some adjustment/clarification. I also have some comments on the sea ice application.

Before describing my comments in detail, I will note that there is an *arXiv* preprint by the authors entitled "Limits and powers of Koopman learning" (arXiv:2407.06312) that appears to be closely related to this manuscript but is not cited in the bibliography. If the preprint is being considered as a peer-reviewed journal article or proceedings paper it should be cited and its relationship with the present manuscript should be commented on.

Dimitris Giannakis

Comments on abstract and sections until "Three examples"

1. In my initial reading of the abstract and first few paragraphs, my impression had been that the paper would address general learning techniques. I am referring to statements such as "reliable learning is impossible, regardless of algorithm choice" in the abstract and "first examples of problems in data-driven dynamical systems that no sequence of algorithms, probabilistic or otherwise, can solve" in the second paragraph of the right-hand column of p. 1. However, later on it becomes clear that the focus is on learning with Koopman/transfer operator techniques, and in particular methods that utilize the spectrum of the Koopman operator in L^2 spaces. On the face of it this is a significantly narrower class of algorithms that the wording mentioned above suggests. Is it obvious that the impossibility results for algorithms utilizing approximation of the Koopman spectrum

spectrum in L^2 extend to arbitrary algorithms (again, as the wording suggests)? If this is true, I would suggest adding an explanation in a prominent location in the manuscript. Either way, I believe that it is important to clarify in the abstract and early on in the main text that the bulk of the analysis will be on the specific class of Koopman spectral approximation techniques, even if the scope of the results can be extended further.

2. The second-to-last paragraph on p. 2 mentions that the paper introduces the "first general-purpose, convergent methods for Koopman learning". The first paragraph caption of Figure 2 also refers to a "new method" without providing an explicit reference. I am sorry if I missed this somewhere in the details, but what is the new method introduced in this paper? Is this related to the RAGE theorem discussed in the Methods section? As far as I could tell the numerical methods make significant use of approaches previously developed by the authors (e.g., residual methods involving $\mathcal{K}_F^* \mathcal{K}_F$). In my opinion the new aspects of the Koopman approximation methodology introduced in the paper should be more strongly emphasized. A pseudocode that can be referenced (e.g., in Figure 2) would also be helpful.

3. Figure 2: What is being plotted in the Error axis in panel (c)? Please update the label and/or include the information in the caption.

4. The statement in the top-right of p. 6 that "many practitioners simply believe in the limitless power of such approaches" seems overly speculative/subjective. I suggest rewording that sentence.

Comments on the sea ice application

1. It has been known through work dating back at least to the early 2010s [1] that Arctic sea ice exhibits a seasonally-modulated reemergence of correlations, whereby sea ice anomalies occurring during the growth season (in the fall) reemerge in the following melt season (spring) despite a loss of correlation in the intervening winter months. This phenomenon, as well as a related melt-to-growth reemergence was originally revealed using conventional correlation techniques and it was physically interpreted through various mechanisms involving the dynamic/thermodynamic coupling between sea ice, the upper ocean, and atmosphere. In [3] (and earlier papers referenced therein) we studied sea ice reemergence using a kernel eigendecomposition technique (NLSA) that in many ways behaves similarly to Singular Spectrum Analysis or Hankel DMD. A finding of this work was the correlation structure of sea ice reemergence can be reconstructed using low-dimensional families of modes consisting of a low-frequency (interannual to decadal) mode as well as pairs of modes given by products between the low-frequency mode and the harmonics of the annual cycle. See, e.g., Fig. 2 in [4] for representative time series that broadly correspond to real and imaginary parts of Koopman eigenfunctions obtained through DMD-type methods.

I suspect that the "hidden" decaying sea ice modes shown in Figure 3 of this paper are related to those seasonally modulated patterns. This is both because of the fact that that the family of blue dots in the spectrum of Figure 3 consists of a single eigenfunction of zero/low-frequency together with its corresponding annual and semiannual modulated pairs, as well as the spatial structure of the SIC anomalies (featuring, e.g., anomalies of opposite sign in the Bering and Beaufort seas) which is characteristic of the aforementioned sea ice reemergence behavior. A way to verify this would be to (i) compare the moduli of eigenfunction time series in the "hidden" family and test whether the modulus of the zero-frequency eigenfunction in that family correlates significantly with

the moduli of the other eigenfunctions in the family; (ii) compute time-correlation functions of reconstructed sea ice concentration/extent based on Koopman eigenfunctions and modes, and look for characteristic reemergence "branches" in the correlation structure (e.g., Figs. 1, 2 in [3]).

Either way, I suggest commenting on previous work related to sea ice reemergence as it appears to be at least potentially related. I would also suggest to adjust text such as "undetected by conventional approaches" in the abstract, unless there is significant evidence that the hidden modes in Figure 3 are not related to reemergence. If this turns out to be the case the authors may wish to investigate whether sea ice reemergence modes are present deeper in the spectrum since they are a recognized source of coherent sea ice variability.

2. In the climate science literature, such seasonally modulated product modes are sometimes referred to as "combination modes" [8], and are found in different contexts such as the El Niño Southern Oscillation and Antarctic sea ice variability; see e.g., the generator spectrum in Fig. 3 of [6] where a number of such families can be found.

3. In the second paragraph of p. 7 the decay constant $-1/\log|\lambda|$ of the hidden modes is connected with decay in the Antarctic region (I am referring specifically to the phrase "consistent with decay times observed in the Antarctic region"). Did you mean Arctic here as opposed to Antarctic? And what does "decay" refer to in this context; is it decline of sea ice extent? If so, I couldn't see how the decay constant based on λ (which measures decay of correlations of observables under the approximate Koopman operator) is directly connected to characteristic timescales of sea ice loss. Moreover, I don't believe that concentration of anomalies in the Barents and Kara Seas is indicative of sea ice reduction in and of itself—these regions have significant natural variability due to the presence of marginal ice zones. For a more definitive test, I would suggest examining the corresponding Koopman eigenfunction time series—if that time series has an appreciable trend component over the analysis interval this would provide significantly stronger evidence of a connection with sea ice loss due to climate change. In that case, the hidden modes with nonzero $\arg(\lambda)$ could be interpretable as modulations of the seasonal cycle by the trend (see Fig. 8 in [7] for an example involving precipitation). However, it is entirely possible that the zero-frequency mode in the hidden family is related to natural low-frequency climate patterns rather than trends. Either scenario would be interesting in my opinion.

4. Figure 4 and related text:

(a) Could you provide the definition of "entire sea ice concentration"? Is this the average sea ice concentration (a scalar between 0 and 1) summed or averaged over all gridpoints? If so I wonder why the authors made that choice of observable instead of sea ice extent (which is shown in Figure 7). As far as I know, sea ice extent is a standard choice for pan-Arctic sea ice forecasting.

(b) How is the relative mean squared error computed? Specifically, what is the normalization factor used to compute relative errors; e.g., is it based on the standard deviation of the full sea ice concentration or the standard deviation of anomalies relative to monthly climatology (average of all Januaries, Februaries, etc. over a reference period such as the training period)? The latter would be more appropriate since the seasonal cycle is a dominant, but trivially predictable, component of the signal. If predicting sea ice extent, the authors could consider reporting un-normalized RMSEs in units of area.

(c) The figure should include forecast errors by simple baseline models for comparison. Two standard choices are the persistence model and a periodic model that predicts by monthly climatology. More elaborate models are oftentimes assessed through the additional forecast skill they provide beyond these baseline approaches; e.g. [2].

(d) I am concerned that the 4-year verification interval (2016–2020) is too short to draw statistically robust conclusions about forecast skill given various sources of interannual and decadal climate variability. Since there are data available from 1979–2021 why not use the first half for training and the second half for verification? Another option might be to use the same verification interval (2012–2020) as in Figure 5. In this way the results in Example 1.3 would be organized around two Koopman models (one for the spectral computations in Figure 3 and another one for forecasting) as opposed to three different models in the current setup.

5. In general, do the forecast models utilizing shorter training intervals have Koopman spectra of similar structure/quality to the spectrum depicted in Figure 3 (e.g., in terms of eigenvalue error)? Do these models also have hidden decaying sea ice modes? Since spectral approximation is placed at the center-stage of learning dynamical systems it would be helpful to include that information somewhere; e.g., include plots of representative spectra in the Supplementary Information.

6. I couldn't find information about the number of time-delays used in the various sea ice experiments. Please include that or highlight it more strongly if already present. Also, please confirm that in the prediction experiments delay-embedding is always "backward"? In other words the initial conditions do not contain information from the future.

7. Similarly to comment 4(c), it would be useful to include results from persistence and/or climatology forecasts in Figure 5. It's not obvious what is the binary accuracy that one can expect from simple models.

Comments on Results section

1. As mentioned above, the bulk of the analysis in this section is on learning algorithms for dynamical systems that involve approximating the approximate spectrum (no pun intended) $\mathrm{Sp}_{\mathrm{ap}}(\mathcal{K}_F)$ of the Koopman operator on L^2 spaces. A general question I had is to what extent are the barriers to learning derived for this class of algorithms representative of general learning algorithms, including operator-theoretic algorithms that do not perform spectral computations, or even nonlinear approximation algorithms that do not employ Koopman/transfer operators?

For a variety of prediction applications, learning the Koopman operator in the strong operator topology (or even weak topology) without approximating its spectrum seems adequate, is that not the case? I am thinking of basic examples—for instance consider the class of continuous maps $F \colon X \to X$ on closed, smooth submanifolds X of Euclidean space $\mathbb{R}^d$ for any dimension d. There exist various graph-theoretic algorithms for approximation of heat operators (e.g., [5]) such that given i.i.d. samples $x_1, \dots, x_M \in X$ drawn from an arbitrary fully supported probability measure μ on X with smooth density relative to the Riemannian volume measure, they produce one-parameter families of kernel functions $p_M \in C(X \times X)$ (oftentimes, by normalization of Gaussian kernels on $\mathbb{R}^d$ with a bandwidth parameter ϵ_M decreasing to 0 as $M \to \infty$ at an appropriate rate) whose

corresponding integral operators $P_M \colon C(X) \to C(X)$,

$$P_M f = \int_X p_M(\cdot, x) f(x)\, d\mu_M(x) \equiv \frac{1}{M} \sum_{m=1}^{M} p_M(\cdot, x_m) f(x_m),$$

converge strongly to the identity as $M \to \infty$. (Here, $\mu_M = \frac{1}{M} \sum_{m=1}^{M} \delta_{x_m}$ is the empirical sampling measure.) Define $\mathcal{K}_{F,M} \colon C(X) \to C(X)$ as $K_{F,M} = P_M \mathcal{K}_F$, where

$$K_{F,M} f = \frac{1}{M} \sum_{m=1}^{M} p_M(\cdot, x_m) f(x_{m+1}).$$

Then, as $M \to \infty$ $K_{F,M}$ converges strongly to the Koopman operator $\mathcal{K}_F$ on $C(X)$. In particular, given $f \in C(X)$ and samples $f(x_2), \dots, f(x_{M+1})$ for training, $\mathcal{K}_{F,M} f$ is an empirically computable continuous function that converges uniformly to $\mathcal{K}_F f$ in a single limit of $M \to \infty$ with probability 1. Doesn't this constitute some form of "learning" of the dynamical system generated by F that is compatible with Definition 11 in the Supplementary Information?

Applying this procedure componentwise to the components $(F_1, \dots, F_d)$ of the map using snapshot data for training would lead to a consistent approximation of F in a single limit. However, the impossibility result stated in the paper for the class $\Omega_{[0,1]}$ would suggest that algorithms using $\mathrm{Sp}_{\mathrm{ap}}(\mathcal{K}_F)$ as part of the computation would need at least two limits. Assuming there is no loophole somewhere in my argument (e.g., the fact that X has no boundary makes this class of systems intrinsically different from $\Omega_{[0,1]}$), I believe it should be mentioned that algorithms bypassing spectral computations may be not subject to the same impossibility result.

I would like to emphasize that this is by no means a criticism of the theoretical results presented in the paper—as mentioned in the opening of my report I believe they provide deep insights about aspects of learning dynamical systems. However, in my opinion statements such as "These impossibility results are *universal*, applying to any type of algorithm, regardless of what n represents" on p. 8 could benefit from some additional quantification if the example given above is valid. A modest rewording in a few instances, e.g., say something like "These impossibility results are *universal* among operator-theoretic algorithms utilizing computation of approximate Koopman spectra..." I believe would suffice. Even if the example given above is not valid, it might still be worthwhile to comment on it as I imagine I'm not the only one who would think of kernel smoothing as a simple way of learning continuous maps on manifolds.

2. Page 2 of the Supplementary Information explains that well-definition of the Koopman operator $\mathcal{K}_F$ on $L^2(X, \omega)$ relies on the map F being nonsingular with respect to the measure ω. I think that mentioning this requirement in the main text as well would be worthwhile. In particular, the definition of the class $\Omega_{\mathcal{X}}$ on p. 8 ("...the broader class of systems where F is continuous, without any additional assumption") should be narrowed down if the intent is to define a Koopman operator on $L^2(\mathcal{X}, \omega)$ for every element of this class. Besides well-definition, do you need closability of $\mathcal{K}_F$ to make various spectral computations meaningful?

3. While I believe that Table 1 provides a nice bird's-eye view of various algorithms in the literature, I do believe that certain important algorithmic nuances could be more strongly emphasized, particularly for the benefit of readers who are not specialists in the field. I can speak more closely about references [17, 60, 10] in Table 1, where I was a coauthor. In that line of work, a common

theme was to simultaneously learn a well-conditioned dictionary (orthonormal basis) *and* solve the spectral/pointwise approximation problem for the operator in question (typically the generator of continuous-time systems) using its representation in that dictionary. For the class of systems under study (measure-preserving ergodic flows on manifolds embedded in Euclidean data spaces) this approach led to a family of algorithms which, as I understand, meet the requirements for a general algorithm in Definition 2 of the Supplementary Information. In particular, for a given set of inputs (snapshot data) and choice of approximation parameters (e.g., number of basis functions N in the notation of this manuscript) there was a concrete computational procedure producing an output such as a spectrum or matrix representation of an operator. On the other hand, looking at the original EDMD [41] and generator EDMD [79] papers, it appears that the choice of dictionary is largely left to the user. In fact, the abstract of [41] states "The method requires a data set of snapshot pairs and a dictionary of scalar observables. . . ". Without this important input, the (generator) EDMD methods as formulated in [41, 79] do not seem to be complete computational algorithms in the sense of Definition 2, so how was their SCI determined? Is there a canonical dictionary construction that was used for systems in each class Ω? I believe that the construction of the dictionary should be taken into account if it has implications on the SCI, or an explanation should be added if it does not.

In other words, I agree that minimizing the number of iterated limits required is an important consideration (as discussed, e.g., in the last paragraph of p. 9), but if certain approaches achieve part of this efficiency by relegating an important design decision to the user, namely the construction of dictionaries/bases for large classes of systems, directly comparing them with methods that offer a solution to this problem feels somewhat like an apples-to-oranges comparison.

4. My final comments are on the radial basis function construction described in the second paragraph in on p. 14.

(a) I wasn't sure how k-means clustering fits in the scheme of the iterated data size ($M \to \infty$) and dictionary size ($N \to \infty$) limits. Is it obvious that k-means is sufficiently well-behaved so as to ensure that output of the algorithm converges as $N \to \infty$ at fixed M? Could one run into issues with k-means failing to converge or producing cluster centers that depend sensitively on the choice of initial condition in the k-means iteration?

(b) Independently of the above, if the bandwidth of the Gaussian RBFs is held fixed then as $M \to \infty$ the basis elements will become increasingly colinear. For a computational algorithm that operates with finite arithmetic precision, does that create stability/conditioning issues? Would that necessitate the use of some regularization procedure for sufficiently large M (or adaptive bandwidth tuning), potentially with its own parameters leading to an increased "practical" SCI?

References

[1] E. Blanchard-Wrigglesworth, K. C. Armour, C. M. Bitz, and E. DeWeaver. Persistence and inherent predictability of Arctic sea ice in a GCM ensemble and observations. *J. Climate*, 24: 231–250, 2011. doi:10.1175/2010jcli3775.1.

[2] E. Blanchard-Wrigglesworth, R. I. Cullather, W. Wang, J. Zhang, and C. M. Bitz. Model forecast skill and sensitivity to initial conditions in the seasonal Sea Ice Outlook. *Geophys. Res. Lett.*, 42(19):8042–8048, 2015. doi:10.1002/2015GL065860.

[3] M. Bushuk and D. Giannakis. The seasonality and interannual variability of Arctic sea-ice reemergence. *J. Climate*, 30:4657–4676, 2017. doi:10.1175/JCLI-D-16-0549.1.

[4] M. Bushuk, D. Giannakis, and A. J. Majda. Reemergence mechanisms for North Pacific sea ice revealed through nonlinear Laplacian spectral analysis. *J. Climate*, 27:6265–6287, 2014. doi:10.1175/jcli-d-13-00256.1.

[5] D. B. Dunson, H.-T. Wu, and N. Wu. Spectral convergence of graph Laplacian and heat kernel reconstruction in L^∞ from random samples. *Appl. Comput. Harmon. Anal.*, 55(NN):282–336, 2021. doi:10.1016/j.acha.2021.06.002.

[6] G. Froyland, D. Giannakis, B. Lintner, M. Pike, and J. Slawinska. Spectral analysis of climate dynamics with operator-theoretic approaches. *Nat. Commun.*, 12:6570, 2021. doi:10.1038/s41467-021-26357-x.

[7] G. Froyland, D. Giannakis, E. Luna, and J. Slawinska. Revealing trends and persistent cycles of non-autonomous systems with operator-theoretic techniques: Applications to past and present climate dynamics. *Nature Commun.*, 15:4268, 2024. doi:10.1038/s41467-024-48033-6.

[8] M. F. Stuecker, F. F. Jin, and A. Timmermann. El Niño–Southern Oscillation frequency cascade. *Proc. Natl. Acad. Sci.*, 112(44):13490–13495, 2015. doi:10.1073/pnas.1508622112.

Adversarial Dynamical Systems Reveal Limits and Rules for Trustworthy Data-Driven Learning

Review of Revision 1

I would like to thank the authors for their thorough responses to the Referee comments and associated revisions of the manuscript. In my opinion, the review comments have been well-addressed and the manuscript is publishable in *Nature Communications* after minor revision. I only have a few remaining comments.

1. SI Figure 9:

   - I would suggest using calendar dates (similarly to Figure 3 in the main text) in the x-axes rather than month numbers.
   - From the point of view of making connections with climate change, I found it somewhat counter-intuitive that the ϕ_{decay} time series exhibits a near-monotonic reduction of negative slope as time (n) increases. I understand that this behavior is consistent with the corresponding Koopman eigenvalue, but as far as I know sea ice loss has been slower in the 1970s and 1980s, then underwent acceleration in the 1990s to early 2000s, and then entered a period of general slowdown or "hiatus" lasting through the present. This non-monotonic trend behavior is to some extent visible in the moving mean curve in Figure 3. The revised manuscript states that the time series of ϕ_{decay} provides "strong evidence of a connection to sea ice loss from climate change", but it is not clear how to reconcile the near-monotonic behavior of ϕ_{decay} with the non-monotonic rates of sea ice loss observed over the past few decades.

     In summary, while I have no doubt that there is a connection between ϕ_{decay} and climate change, the details of that connection may be quite nuanced. I would therefore be hesitant to call the evidence strong without further justification of the differences between ϕ_{decay} with known trend patterns as noted above.
   - It might be worthwhile examining the behavior of ϕ_{decay} computed separately using the first half and second half of the 1979–2021 analysis interval. Would the eigenfunction time series slopes be mutually consistent between these calculations and the one from the full analysis interval?

2. p. 5, "Hidden modes with nonzero arg(λ) can be interpreted as seasonal patterns modulated by this long-term trend.":

   I believe a reference to [28] (who made a similar observation for eigenfunctions computed from SST data) would be appropriate here; see, e.g., subsection "Response of seasonal cycle of precipitation to climate change" where these modes are referred to as "trend combination modes".

3. In their response to comment 4(c) in my review the authors mention that the periodic baseline model considerably outperforms the persistence model. I wasn't sure how to fully interpret this statement since, by construction, the persistence model has zero error at the forecast initialization time, and would thus outperform all models shown in Figures 4 and 5. Presumably, the persistence forecast loses skill more rapidly than other models, but including it as a means of gauging improvement in rate of skill loss that these models offer could still be helpful.

   Did the authors mean that the persistence forecast considerably underperforms the other models even for lead times as short as 1 month? If so it would be useful to include that information.

4. Thank you for clarifying that "entire sea ice concentration" corresponds to sea ice concentration fields (comment 4(a) in my review) and the error metric used to assess forecast skill in Figure 4 is given by

$$\text{Error} = \frac{\|\boldsymbol{x} - \boldsymbol{x}_{\text{rec}}\|_{l^2}^2}{\|\boldsymbol{x} - \boldsymbol{x}_{\text{per}}\|_{l^2}^2}. \tag{1}$$

   The results in Figure 4 indicate that this error metric does not saturate to the baseline climatological forecast over prediction horizons extending out to more than five years (with either DMD or the proposed method). This is considerably longer than predictability limits expected for sea ice dynamics, where, for example, skillful forecasts of September pan-Arctic sea ice area to a few months in advance (let alone several years in advance) would be considered a success. I am therefore concerned that the error metric in (1) does not clearly reflect when loss of skill has taken place.

   - I presume that the l^2 norms in (1) correspond to integration over spatial gridpoints, is that right? If so, can the authors confirm that these norms are approximations of area integrals over the Arctic spatial domain? The main text states that each gridpoint has a fixed area of 625 km^2 but as far as I could tell (apologies if I missed the relevant details) there is no information provided about the meridional extent of the domain or the longitude–latitude sampling employed.
   - It is not clear how Error from (1) is averaged to produce the results in Figure 4. Is Error averaged over the initial conditions, or the numerator and denominator are averaged separately before computing the ratio?
   - Assuming that $\|\cdot\|_{l^2}$ corresponds to area integrals, wouldn't it be more interpretable to compute σ^2 as the time average of $\|\boldsymbol{x} - \boldsymbol{x}_{\text{per}}\|_{l^2}^2$ at lead time 0 over the training dataset, and show time averages of

$$\text{Error}_{\text{rec}} = \frac{\|\boldsymbol{x} - \boldsymbol{x}_{\text{rec}}\|_{l^2}^2}{\sigma^2}, \quad \text{Error}_{\text{per}} = \frac{\|\boldsymbol{x} - \boldsymbol{x}_{\text{per}}\|_{l^2}^2}{\sigma^2} \tag{2}$$

     over initial conditions in the verification dataset? In this way it would be clear (i) how the errors of both "rec" and "per" forecasts compare to the climatological variance σ^2 relative to the seasonal cycle; and (ii) when the error of $\boldsymbol{x}_{\text{rec}}$ has saturated to that of $\boldsymbol{x}_{\text{per}}$. In the current Figure 4, (i) is missing.
   - A typical pan-Arctic domain will contain gridpoints where predictability of sea ice concentration is trivial over certain months of the year (e.g., winter months at a sufficiently

high latitude or summer months at a sufficiently low latitude). Given that, it may be more informative to report errors for the aggregated pan-Arctic sea ice area or extent (e.g., using a modification of (2)) rather than spatially averaging pointwise errors. Pan-Arctic sea ice area or extent are also commonly used target variables in predictability studies which would facilitate comparison of the results in this paper with other works in the literature.
